# Modular co-option of cardiopharyngeal genes during non-embryonic myogenesis

**DOI:** 10.1186/s13227-019-0116-7

**Published:** 2019-03-05

**Authors:** Maria Mandela Prünster, Lorenzo Ricci, Federico D. Brown, Stefano Tiozzo

**Affiliations:** 1 Laboratoire de Biologie du Développement de Villefranche-sur-mer (LBDV), CNRS, Sorbonne Université, 06230 Villefranche sur Mer, France; 2 Department of Organismic and Evolutionary Biology, Harvard University, 52 Oxford Street, Cambridge, MA 02138 USA; 3 Departamento de Zoologia, Instituto Biociências, Universidade de São Paulo, São Paulo, SP CEP 05508-090 Brazil; 4 Centro de Biologia Marinha (CEBIMar), Universidade de São Paulo, São Sebastião, SP CEP 11612-109 Brazil

**Keywords:** Blastogenesis, Ascidians, *Botryllus schlosseri*, Budding, Muscle, Regeneration

## Abstract

**Background:**

In chordates, cardiac and body muscles arise from different embryonic origins. In addition, myogenesis can be triggered in adult organisms, during asexual development or regeneration. In non-vertebrate chordates like ascidians, muscles originate from embryonic precursors regulated by a conserved set of genes that orchestrate cell behavior and dynamics during development. In colonial ascidians, besides embryogenesis and metamorphosis, an adult can propagate asexually via blastogenesis, skipping embryo and larval stages, and form anew the adult body, including the complete body musculature.

**Results:**

To investigate the cellular origin and mechanisms that trigger non-embryonic myogenesis, we followed the expression of ascidian myogenic genes during *Botryllus schlosseri* blastogenesis and reconstructed the dynamics of muscle precursors. Based on the expression dynamics of *Tbx1/10*, *Ebf, Mrf, Myh3* for body wall and of *FoxF, Tbx1/10, Nk4, Myh2* for heart development, we show that the embryonic factors regulating myogenesis are only partially co-opted in blastogenesis, and that markers for muscle precursors are expressed in two separate domains: the dorsal tube and the ventral mesenchyma.

**Conclusions:**

Regardless of the developmental pathway, non-embryonic myogenesis shares a similar molecular and anatomical setup as embryonic myogenesis, but implements a co-option and loss of molecular modules. We then propose that the cellular precursors contributing to heart and body muscles may have different origins and may be coordinated by different developmental pathways.

**Electronic supplementary material:**

The online version of this article (10.1186/s13227-019-0116-7) contains supplementary material, which is available to authorized users.

## Background

Musculature is a tissue specialized in contraction shared among all eumetazoans. Its cellular components contain molecular structures based on actomyosin and an array of accessory proteins which allows contractility [1]. Myogenesis operates through a progressive activation of transcription factors organized in a hierarchical and modular network that drive cell fate specification, and is followed by specific cell behaviors that lead to muscle differentiation and organization [2]. For example, trunk skeletal muscles of vertebrates develop from somites and are determined by the expression of the paired box transcription factor *Pax3* [3], whereas cardiac, pharyngeal [4], and possibly also esophagus striated muscles [5] originate from cardiopharyngeal mesoderm and are determined by T-Box1. A common origin of heart and pharyngeal muscles from the cardiopharyngeal field predates vertebrate evolution [4, 6–8] and has been hypothesized to be a chordate synapomorphy [9, 10].

In metazoans, muscles can develop during embryogenesis and during non-embryonic development of adult organisms, i.e., regeneration and asexual development. The myogenic process may be triggered by populations of multi- or unipotent stem cells [11–14]. Post-embryonic myogenesis also occurs in species with indirect development and complex life cycles [15]. These animals undergo drastic changes between their larval and adult bauplan, such as during metamorphosis, and the musculature can radically change architecture within the same organism between different life stages [16, 17].

With their biphasic life history and asexually reproducing colonial species, ascidians (Tunicata) offer great opportunities to study the development of different muscle architectures in different life stages [18]. From the fertilized egg, a stereotyped development and largely determinative embryogenesis [19] leads to the formation of a planktonic larval body. In the larval tail, bands of mononucleated myocytes are arranged in a striated fashion [20] and express a myosin heavy chain, specific of embryonic muscles [21, 22]. When the larva settles on a substrate and metamorphoses into a sessile filter-feeding adult, the sarcomeric arranged musculature gets reabsorbed along with the tail [23] and non-striated circular and longitudinal body muscles form along the mantle of the organism, together with the cardiac muscles [20, 24]. The adult body wall muscle are described as smooth (non-striated) muscles, which apparently evolved due to a loss of sarcomeric organization, probably in order to cope with a sessile lifestyle, which requires slow contractions for the fine-tuning of water inflow [20]. The body and heart muscles express two specific, post-metamorphic myosins: *myosin heavy chain 3* and *myosin heavy chain 2* [22], respectively.

During embryonic development of solitary ascidians, maternal deposition of the zinc finger family member Zic-r.a (Macho-1) is essential for early muscle specification [25]. Zic-r.a activates the expression of the T-box transcription factor *Tbx6*, necessary to induce the tail muscles [26]. Zic-r.a and Tbx6 together with beta-catenin define the cardiopharyngeal field by activating the bHLH regulatory gene *mesodermal posterior* (*Mesp)* [27]. The cardiopharyngeal field, a.k.a. the trunk ventral cells (TVCs) in *Ciona,* gives rise to both the heart [28] and part of the adult ascidian body musculature. The expression of the homeobox transcription factor *Nk4*, ortholog to tinman/Nkx2-5, in TVCs antagonizes *Tbx1/10* and promotes the cardiac muscle fate through the activation of *Gata 4/5/6* [29]. The heart muscle will continue to differentiate and express myosin heavy chain 2 [22]. On the other hand, a subset of the TVCs express the transcription factor *Tbx1/10*, which promotes the expression of *Ebf* (COE, Collier/OLF/Ebf) and orchestrates the transition to the myogenic program by the activation of myogenic regulatory factor (Mrf), eventually giving rise to the longitudinal muscle and the muscles around the atrial siphon [29]. Another set of body muscle, the oral siphon muscles, derive from a different population of cells named trunk lateral cells (TLCs) in *Ciona*. TLCs follow a different fate but are partially regulated by the same transcription factors (TFs) involved in muscle development of TVCs [30, 31]. After metamorphosis, all the body muscles continue to differentiate by expressing the same myosin heavy chain 3 [22].

**Fig. 1:**
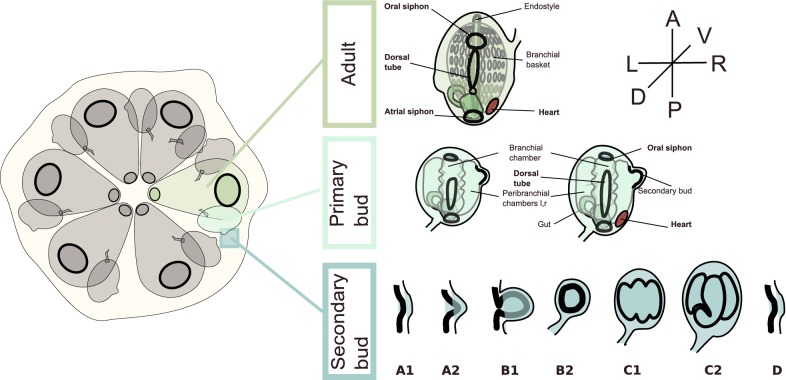
Development and staging of a *Botryllus schlosseri* colony. The staging of the animals was performed after Lauzon [63]. The secondary bud develops as thickening of the peribranchial epithelium and the epidermis (stages A1, A2), which evaginates and closes forming a double monolayered vesicle (stages B1, B2). The inner vesicle undergoes morphogenesis and is subdivided into three chambers (stages C1, C2). During “takeover” (stage D), the adult degenerate and get resorbed, the primary buds become adult, the secondary buds become the primary buds and start to bud again a new secondary bud, so a new blastogenetic cycle begins for the next secondary bud (A1) [32, 34]

In colonial species of ascidians, such as *Botryllus schlosseri,* the post-metamorphic individual, the oozooid, begins asexual budding from undifferentiated cells, in a process named blastogenesis, which leads to the development of an adult zooid, the blastozooid (Fig. 1). Therefore, the life cycle of a colonial ascidian is characterized by three different body plans: the larva displaying most chordate features, the post-metamorphic oozooid, and the asexually propagated blastozooid. By producing multiple buds, in a cyclic manner, blastogenesis eventually leads to the formation of colonies composed of several genetically identical blastozooids. Regardless of their different ontogenetic origins, the overall anatomy of the oozooid and the blastozooid is similar, including the general organization of their musculature [20, 24, 32]. However, in blastogenesis the process of development is direct; namely, it skips the determinative steps of embryonic development [33] and passes neither through a larval stage nor metamorphosis.

In order to investigate the mechanisms underlying clonal replication of muscular systems in colonial ascidians and infer the origin of muscle precursors during blastogenesis, we selected genes involved in embryonic and metamorphic myogenesis in the well-studied solitary ascidian *Ciona*, and followed their expression during blastogenesis of *B. schlosseri*. With the aim to reconstruct the dynamics of muscle formation in ascidians, we took advantage of the extensive literature on myogenesis in solitary ascidians, as well as of the broad and detailed descriptions of *Botryllus* ontogeny [34] and we established a relationship between the spatiotemporal pattern of expression of a robust, conserved cascade of myogenic-specific genes and the detailed morphological descriptions of *Botryllus schlosseri* blastogenesis. Our study revealed that during myogenesis of an asexually derived zooid the embryonic and post-metamorphic myogenic genes are only partially co-opted, reflecting a lack of maternal signals and the absence of the larval stage.

## Results

### Changes in muscle architecture during metamorphosis and blastogenesis

To understand whether the molecular structure of the differentiated muscles in colonial ascidians reflects the expression scenario described in solitary species [22], and in order to compare the molecular structure of the muscle fibers between oozooid and blastozooid, we searched for members of the muscle type class two myosin heavy chains (MYH) in the transcriptomes of multiple stages in *Botryllus schlosseri* [35, 36]. Four paralogues were identified in *Botryllus schlosseri*, including three muscle specific (MYH1, MYH2, and MYH3) and one non-muscle specific (MYH 9/10/11/14). The three muscle-specific MYH clustered together with their corresponding homologous sequences of other solitary ascidians belonging to the order Phlebobranchia or Stolidobranchia (Additional file 1: Fig. 1). MYH3 subfamily constituted the sister group to the cluster of ascidian MYH1, MYH2, and their corresponding vertebrate paralogues. Within the ascidian paralogues, MYH1 and MYH2 clustered as the sister group and are more closely related to the vertebrate muscle-specific MYH sequences (Additional file 1: Fig. 1).

**Fig. 2:**
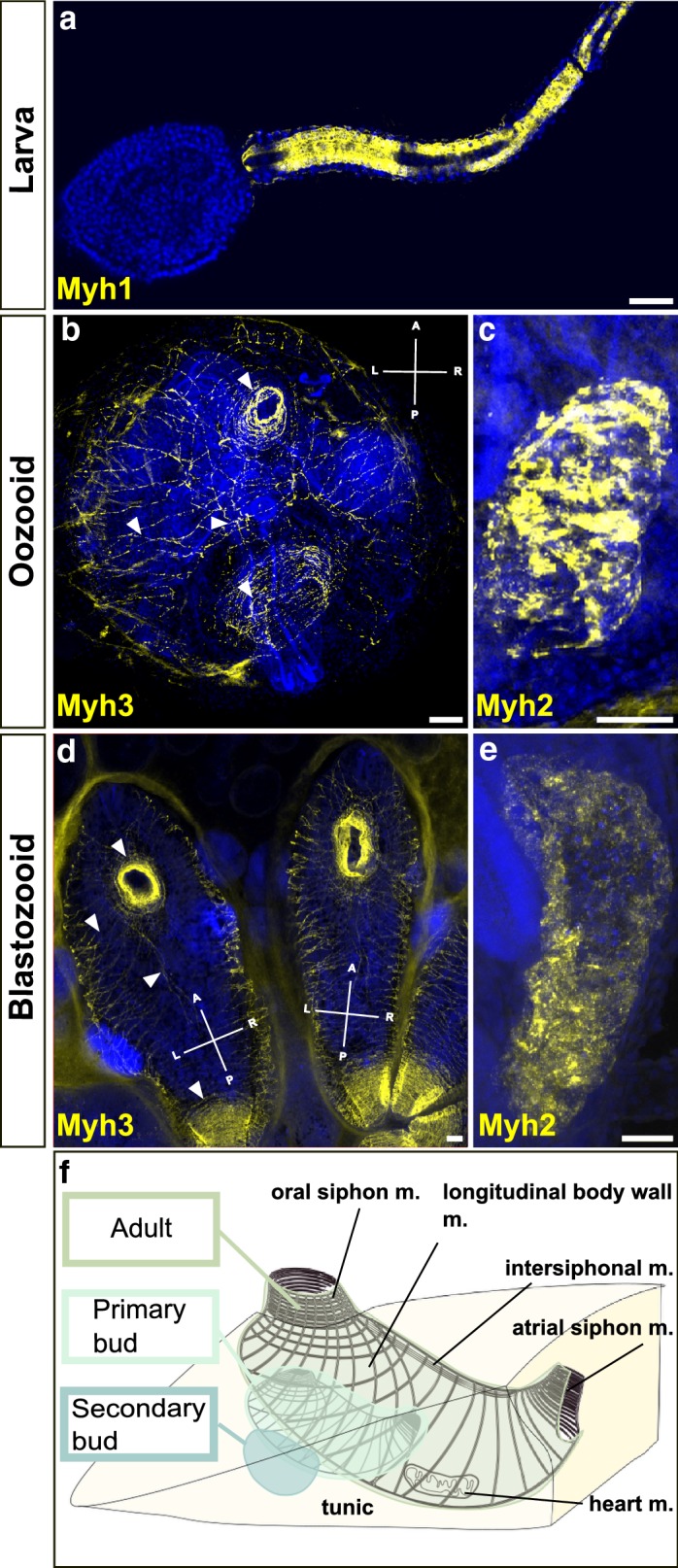
Fluorescent in situ hybridization showing the expression of *myosin heavy chain* mRNAs at different stages of *Botryllus schlosseri* life cycle. **a** Expression of *Myh1* (yellow) along the striated muscle in the larval tail. **b** *Myh3* expression along muscle circular (oral and atrial siphons) and longitudinal muscle fibers in the oozooid body wall. **c** Expression of *Myh2* in the heart of the adult oozooid. **d** *Myh3* expression in the body wall of a blastozooid: circular and longitudinal fibers. **b**–**d** Arrowheads indicating the circular muscles around the oral siphon, the longitudinal body muscles and the intersiphonal muscles. **e** *Myh2* expression in the heart of an adult blastozooid. Gene names are indicated in the lower left corner of the pictures in yellow. Arrowheads point circular and longitudinal fibers. Blue nuclei: Hoechst staining. Scale bar: 50 μm. **f** Schematic drawing of the body musculature of a colony in stage C1. Adult, a primary bud, and a secondary bud are embedded in common tunic. At stage C1 adult and primary bud, the muscle fibers are developed and both are able to contract. The body wall musculature consists of circular muscles around the oral and atrial siphon, longitudinal muscles, and a band of intersiphonal muscles, connecting the two siphons. The heart lies to the ventral and right side of the zooid body

In situ hybridization revealed that the three mRNAs coding for the muscle relevant MYH proteins are expressed in different regions and at different time points of *B.schlosseri* development. Embryonic *Myh* (*Myh1*) was exclusively expressed in the larval stage in tail muscles (Fig. 2a).

The caudal musculature of the swimming larva consists of cylindrical mononucleated cells in longitudinal rows flanking the notochord. During resorption of the larval tail, in metamorphosis, muscle cells lose their cohesion and are pushed into the body cavity, where they become surrounded by phagocytes [37]. The process of tail resorption in *Botryllus schlosseri* (Additional file 2: Fig. 2) occurs within 10 min approximately. *Myh1* was expressed in the swimming larval tail in six rows of muscle cells (Additional file 2: Fig. 2. A1–A6). Within cells of the larval tail muscle, the RNA expression was localized at the anterior and posteriormost tips of the cells and surrounding the nucleus (Additional file 2: Fig. 2. A5).When regression of the tail was almost complete, the RNA expression became restricted to more anterior sites of the tail (Additional file 2: Fig. 2.B1–2,6) and subsequently formed two separate fields, in the posterior larval trunk (Additional file 2: Fig. 2.B3–4, 7). Muscle cell connections were lost and cells became roundish, but myofibrillar striation remained (Additional file 2: Fig. 2.B5). Single cells of embryonic musculature were still found within the mantle of the young oozooid (Additional file 2: Fig. 2.C1–4).

The muscle architecture of the zooid, both oozooid and blastozooid, heavily differed from the larval tail musculature. The adult body musculature is characterized by the expression of *Myh*3. In *Botryllus,* the first expression of *Myh3* was only observed during metamorphosis, at the anterior end of the young oozooid, and where the future intersiphonal muscles set place (Additional file 3: Fig. 3.A1–5, B.1–3, C). In the early oozooid, single larval muscle cells were still present in the mantle of the animal (Additional file 2: Fig. 2.C3, Additional file 3: Fig. 3.A3–6, C), but they do not express *Myh3*. The late oozooid, which already carries a bud in stage C2, showed clear dorsal expression of *Myh3* around the oral and atrial siphons, the intersiphonal muscle bands and the body wall musculature (Additional file 3: Fig. 3.D1–4, F), but not on the ventral side (Additional file 3: Fig. 3.E1–4). The fully developed post-metamorphic oozooids expressed *Myh3* in their entire body wall, including the dorsal and ventral musculature, highlighting the fibrous structure of adult muscles (Fig. 2b), i.e., the circular muscles around the oral and atrial siphons, the longitudinal muscles, and the intersiphonal muscle (Fig. 2f). The blastozooid (zooid developed by budding) expressed *Myh3* from the primary bud at stage A1 until the fully differentiated zooid (Fig. 2d). Expression of *Myh3* was detected in circular, longitudinal, and intersiphonal muscles, mimicking the patterns observed in the oozooid.

The heart in ascidians consists of a simple two-layered tube (myo- and pericard) that beats hemolymph in a reversible orientation through an open circulatory system [28, 38] and is characterized by the expression of *Myh2*. The heart of solitary ascidians does not fully differentiate or begin contractions before metamorphosis, whereas the heart of some colonial ascidians may become fully functional before settlement [39]. In *Botryllus*, *Myh2* expression was first observed at the ventral side of the swimming larva (Additional file 4: Fig. 4.A1–5). The field displays already a sac-like form. In the early oozooid, *Myh2* expression was observed on the beating heart at the left side of the body (Additional file 4: Fig. 4.B1–5). In the fully developed oozooid heart, the muscle fibers of the heart expressed *Myh2* (Fig. 2c). Comparably, *Myh2* expression in blastozooids was localized to the heart of primary buds, and expression was maintained throughout development and in the fully differentiated zooid (Fig. 2e).

### Partial redeployment of embryonic myogenic motifs during blastogenesis

To assay if myogenic motifs expressed during embryonic development and metamorphoses have been co-opted during blastogenesis, we focused on the expression of 17 candidate genes well characterized during the myogenesis of solitary species [31, 40]. We first described the embryonic development of the *B. schlosseri* to assay if the cleavage pattern is conserved between solitary and colonial species. We observed that the early cleavage patterns are comparable to the stereotypical patterns observed in solitary ascidians, including the extensively studied *C. robusta* [33, 41, 42]. However, the timing between each of the cleavages is longer, and the time from the first cleavage and the larval hatching is around 5 days (Additional file 5: Fig. 5). The expression of *Zic*-*r.a*, *Tbx6, Ebf,* and *Tbx1/10* also confirmed the temporal and spatial pattern of expression observed in solitary species: The mRNA coding for the zinc finger transcription factor *Zic-r.a* was localized vegetally at the tip of the two blastomeres of the two-cell stage embryo (Additional file 6: Fig. 6.A). At a 110-cell stage, the expression localized in two posterior blastomeres (Additional file 6: Fig. 6.B). *Tbx6* mRNAs were expressed in a bilateral fashion (Additional file 6: Fig. 6.C–D) in the presumptive myoplasm and future larval tail muscles (Additional file 6: Fig. 6.E). Bilateral *Ebf* expression has been observed in a group of four single cells at the anterior trunk in an early tailbud stage (Additional file 6: Fig. 6.F).

Next, we carried an initial assessment for the presence and relative abundance of gene expression using the transcriptomes obtained from entire non-fertile colonies, including adult zooid, primary and secondary buds, at seven blastogenetic stages [35]. While these transcriptomic datasets cannot be used to observe differential expression during the development of a single bud, these analyses showed the presence of only a subset of candidate genes, whereas others genes important for muscle development, typically expressed early in the embryonic development of *Ciona,* were not found to be expressed during blastogenesis (Table 1, Additional file 7: Fig. 7). The absence of expression of early myogenic genes was also supported by RT-PCR (Additional file 6: Fig. 6.G) and FISH (data not shown). The transcripts that were found in a negligibly low copy number or absent from the transcriptomes belong to myogenic transcription factors *ZicL, LIM (Lhx3), Tbx6, Hand*-*r,* and *Mesp,* whereas *Nk4, Tbx1/10, FoxF, Islet, Myh2*, Gata4/5/6, Mrf*, Myh1,* and *Zic-r.a* (*Macho*-*1*) were expressed at low levels. In contrast, *Myh3, Ebf,* and *Ets* were myogenic factors expressed at high levels throughout the seven stages of *Botryllus* blastogenesis.

**Table 1:** Presence of candidate myogenic motif genes in the transcriptomes of entire colonies at different blastogenetic stages

RPM 0–1	PRM 1–100	RPM > 100
*Hand*-*r*	*Ebf*	*bHLH*-*tun*
*LIM*	*FoxF*	*Ets*
*Mesp*	*Gata4/5/6*	*Myh3*
*Tbx6a*	*Islet*	
*Tbx6b*	*Mrf*	
*ZicL*	*Myh1*	
	*Myh2*	
	*Nk4*	
	*Tbx1/10*	
	*Zic.r*-*a*	

Data are subdivided in three categories: “very low” expression (RPM < 1), “low” expression (RPM 1 > 100), and “high” expression (RPM > 100)

### Development of the body wall musculature during blastogenesis

**Fig. 3:**
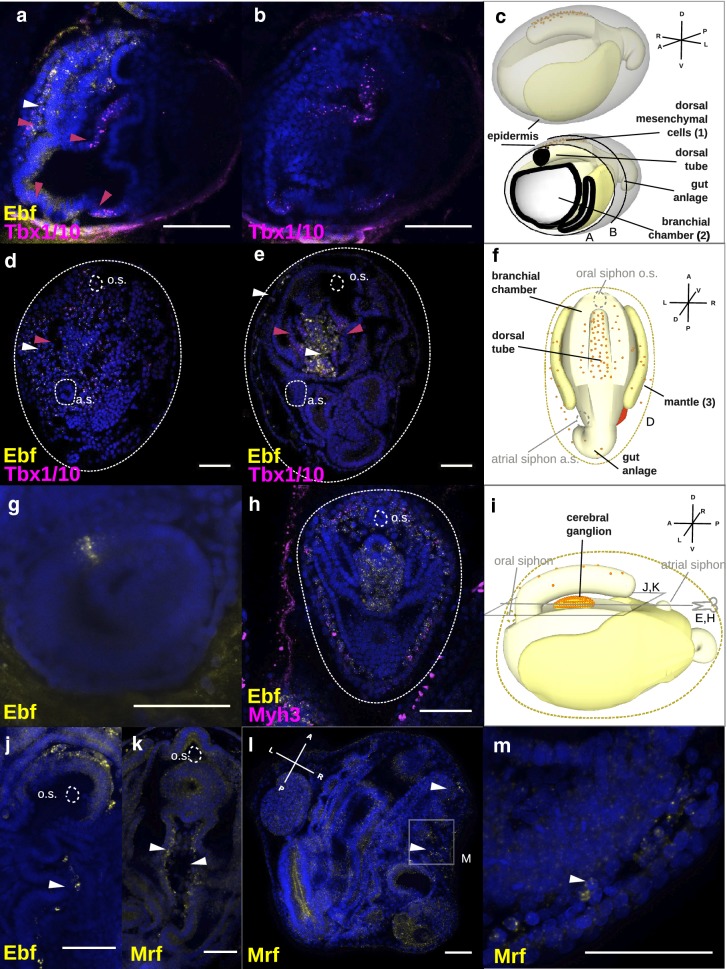
Fluorescent in situ hybridization (FISH) showing expression of body muscle myogenic TFs during blastogenetic development of *Botryllus schlosseri*. **a**–**e** Double ISH showing the expression of *Tbx1/10* (magenta) and *Ebf* (yellow). **a**, **b** *FISH* performed on the same animal in stage C2-D. **a** Magenta arrowheads point to the expression of *Tbx1/10* in the dorsal mesenchyme, the dorsal region of the branchial basket, and the ventral region of the branchial basket lateral to the endostyle, and white arrowheads point to the expression of Ebf in the dorsal mesenchyme. **b** Posterior and transversal section of the same sample showing the expression of *Tbx1/10* in the dorsal and ventral branchial basket. **c** Schematic representation of a developing *Botryllus* secondary bud in stage C2-D, showing the dorsal tube the branchial chamber, the gut, and the epidermis. **d**–**e** FISH performed in stage A1. **d** Magenta arrowheads point to the expression of *Tbx1/10* and white arrowheads to the expression of Ebf in the mantle. O.s: oral siphon, a.s.: atrial siphon. **e** Magenta arrowheads point to the bilateral expression of *Tbx1/10* in the intersiphonal region and white arrowheads to the expression of Ebf in the mantle and the forming cerebral ganglion. **f** Schematic representation of a developing *Botryllus* primary bud in stage A1. **g** Earliest expression of *Ebf* in few single cells of the vesicle-like secondary bud in stage B2, oriented to the primary bud and hence the future dorsal side. **h** Double ISH showing the expression of *Ebf* (yellow) and *Myh3* (magenta), *Ebf* is expressed in mantle together with *Myh3*, in the cerebral ganglion and in the dorsal tube. **i** Schematic representation of a developing *Botryllus* primary bud in stage A, sagittal view. **j** *Ebf* expression in the region of the intersiphonal muscle. **k** Max projection showing the expression of *Mrf* in the region of the intersiphonal muscle. **l**, **m** *Mrf* expression in the mantle of the primary bud. Blue nuclei: Hoechst staining, dashed line: outline of primary bud, scale bar: 50 μm. Asterisk: unspecific staining in either tunic, gonads, or insufficiently bleached auto-fluorescent cells

In order to understand where the body wall muscles originate and develop during *Botryllus* blastogenesis, we investigated the expression of *Tbx1/10*, *Ebf*, and *Mrf*. These three TFs are expressed in one of the two muscle precursor fields in the *Ciona* larval head, specifically in the trunk ventral cells (TVCs). In contrast to descriptions during ascidian embryogenesis, we observed three main regions of expression of these genes in *Botryllus* blastogenesis: (1) a mesenchymal region between the dorsal tube and the dorsal epidermis, (2) the dorsal side of the branchial chamber where the future intersiphonal muscle will develop, and (3) the mantle or body wall, which corresponds to a membrane beneath the tunic made of epithelial tissue, connective tissue, musculature, blood vessels, and nerves (Fig. 3c, f, i).

*Tbx1/10* expression began in the secondary bud at stage C2, in mesenchymal cells between the dorsal tube and the epidermis (Fig. 3a, Additional file 8: Fig. 8.B, F, Additional file 9: Movie 1). In the early primary bud (between stages D–A1), *Tbx1/10* was expressed in mesenchymal cells distributed underneath the epidermis along the entire body of the zooid, i.e., the mantle (Fig. 3d). At stage C2, the secondary bud started to express *Tbx1/10* ventrally in the branchial chamber flanking the forming endostyle as well as in its medio-dorsal region (Fig. 3a, b). This second domain of expression appeared to concentrate laterally in the epithelium and into the cerebral ganglion region in stage D/A1 of the primary bud (Fig. 3e) and was maintained until A2, as it began to appear more diffuse. *Tbx1/10* was also expressed on the ventral side of the bud within the region of the forming heart.

The earliest expression of *Ebf* was in a domain composed of 3 to 5 cells in the secondary bud at stage B2 (Fig. 3g). A second domain of expression appeared in the secondary bud on the dorsal tube [34] at stage C1 and in mesenchymal cells between the dorsal tube and the overlying epidermis, where it was co-expressed with *Tbx1/10* (Fig. 3a, Additional file 8: Fig. 8F). In the primary bud, at stage D-A1 *Ebf* was co-expressed with *Tbx1/10* in mesenchymal cells along the mantle of the zooid (Fig. 3d, Additional file 8: Fig. 8.D, E). From stage B1/B2, *Ebf* was expressed ubiquitously in the cerebral ganglion, in scattered cells around the forming neural gland, and it was co-expressed with the body wall muscle marker *Myh3* in the mesenchymal cells along the mantle, where the future muscle fibers formed (Fig. 3h, Additional file 10: Movie 2). In a C2 primary bud, *Ebf* was expressed in few scattered cells along the intersiphonal region (Fig. 3j).

The transcriptomic data we analyzed showed a low expression of *Mrf* in all blastogenetic stages (Fig. 3h). In addition, FISH showed feeble expression of *Mrf* only in primary buds at stage A2, in cells of the mantle (Fig. 3l, m), as well as in a dorsal domain of the branchial chamber, possibly where *Tbx1/10* was also expressed (Fig. 3k). However, due to the low expression of *Mrf* RNA, it was not possible to perform double FISH for *Mrf* and *Tbx1/10* simultaneously.

### Presence of putative muscle stem cells in the zooid

In *Ciona,* the atrial and oral siphon muscles have been shown to maintain a population of putative muscle stem cells, that is, *Mrf*-*/bHLH*-*tun *+ [31, 43]. While *Mrf* is expressed in differentiating muscle cells, the tunicate-specific helix–loop–helix transcription factor *bHLH*-*tun* is expressed in muscle stem cells [31, 43]. In the *Botryllus* oozooid, the *bHLH*-*tun* mRNA was expressed in a ring around the two siphons (Additional file 11: Fig. 9.A1-5, B.1-4), which probably corresponds to the inner muscle population of the siphon, as was previously suggested in *Ciona*. In addition, *bHLH*-*tun* was expressed in a few cells of the lateral body wall (Additional file 11: Fig. 9.A2) and in a few cells on top of the dorsal tube (Additional file 11: Fig. 9.C1–4). In the *Botryllus* blastozooid, such spatial pattern of expression was not been detected; however, transcriptome analyses showed that *bHLH*-*tun* is in fact present during different stages of blastogenesis (Additional file 7: Fig. 7).

### Heart development during blastogenesis

**Fig. 4:**
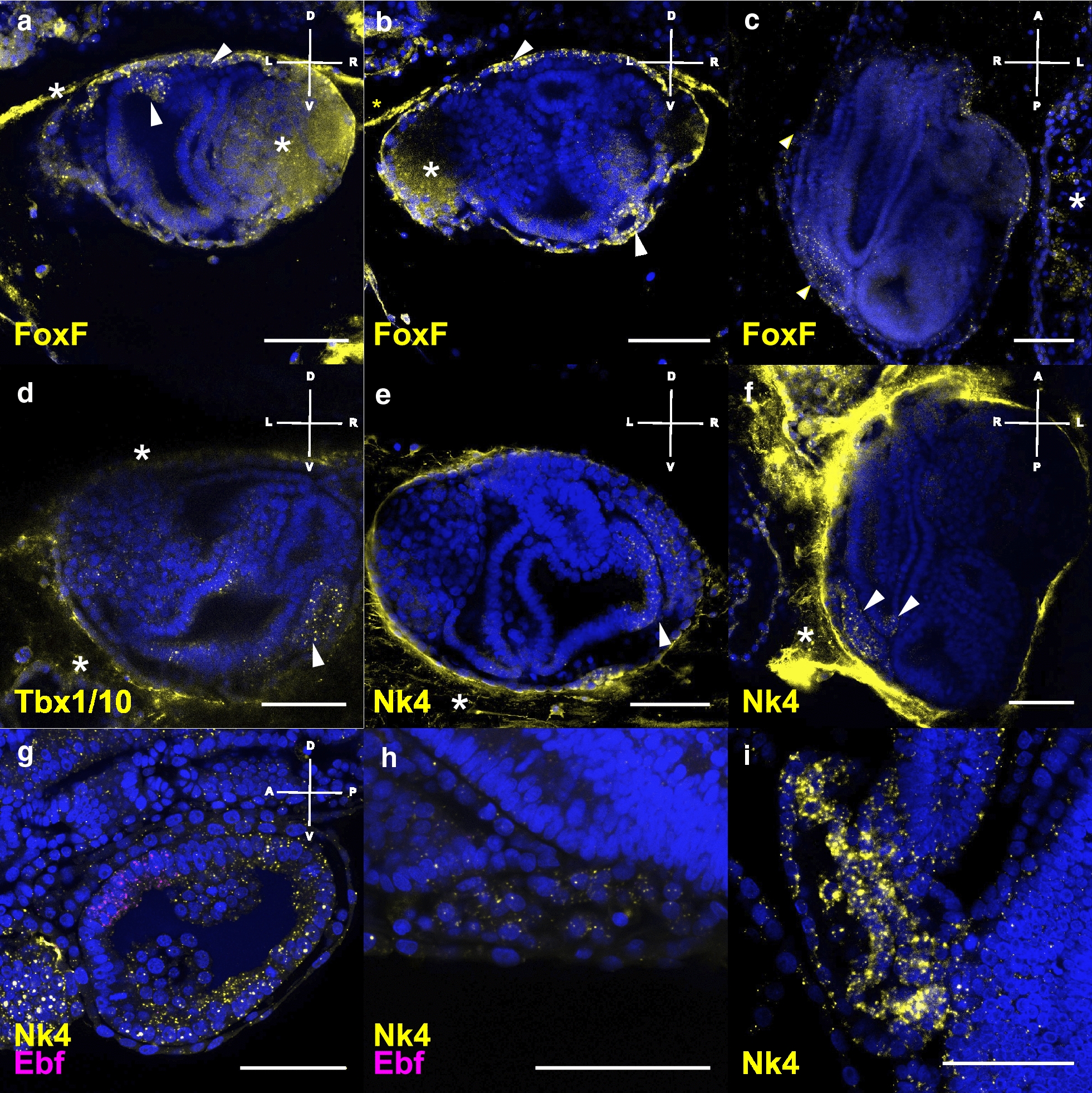
Expression of TFs heart development-related revealed by FISH. **a**–**c** Expression of *FoxF* in secondary bud at stage D: **a** in the dorsal branchial chamber and epidermis, **b** in the forming heart vesicle, and **c** *FoxF* expression in the heart and epidermis in a ventral view shown by a max projection of 13 slides, stage A2. **d** *Tbx1/10* expression in stage D. **e** Asymmetric *Nk4* expression in stage C2, over the entire right peribranchial chamber (white arrowhead) and the right side of the branchial chamber. **f** *Nk4* expression in a ventral view of a primary bud in stage B1. **g**, **h** Double FISH with *Nk4* and *Ebf* in the same B2-C1 colony **g** in the secondary bud and **h** the heart of the primary bud. *Nk4* restricting to the myocardium in late primary bud. Probes are indicated in the lower left corner of the pictures in the same color of the expression. Blue nuclei: Hoechst staining, dashed line: outline of primary bud, scale bar: 50 μm. Asterisk: unspecific staining in either tunic, gonads, or insufficiently bleached auto-fluorescent cells

In order to investigate the formation of heart musculature during blastogenesis, we followed the expression of the TFs *FoxF, Tbx1/10,* and *Nk4*, which characterize the TVC embryonic lineage that gives rise to the heart muscles in *Ciona* [29, 40] (Fig. 4). In contrast, *Botryllus FoxF* mRNA was expressed in cells of the bud epidermis throughout blastogenesis (Fig. 4a–c) and more specifically on the dorsal side of the branchial chamber of the secondary bud on stage D (Fig. 4a). In the *Botryllus* primary bud, *FoxF* seemed to concentrate at sites lateral to the cerebral ganglion (data not shown). Last, we detected expression of *FoxF* in a cluster of cells in the left ventral side of the mantle where the heart formed (Fig. 4b) and in the heart of primary buds (Fig. 4c).

*Tbx1/10* transcripts showed similar patterns of expression to those described above for *FoxF*, but in addition *Tbx1/10* showed expression on the secondary bud (stage D/A1) in a cluster of cells on the left ventral side of the mantle where the heart presumably will form (Fig. 4d).

Heart field determination in *Ciona* is mainly characterized by the expression of *Nk4*, which antagonizes *Tbx1/10* and *Ebf* [29]. In the *Botryllus* secondary bud at stage C2, *Nk4* was expressed asymmetrically on the left side of the branchial chamber epithelium and in the whole left peribranchial chamber (Fig. 4e). At stage B1, *Nk4* expression was observed in the forming heart (Fig. 4f). Expression of *Nk4* in the myocardium remained high during the complete development of the heart until the myocardium was separated from the pericardium (Fig. 4i).

To test whether *Ebf* and *Nk4* expression domains are mutually exclusive, we performed double ISH (Fig. 4g, h) on a single colony. *Nk4* was expressed all over the secondary bud at stage B2–C1 except for the region of *Ebf* expression, which corresponds to the site where the dorsal tube will form (Fig. 4g). We never found *Ebf* expressed at any stage of ascidian heart development (Fig. 4h).

## Discussion

In this study, we characterized mRNA expression patterns of myogenesis-related genes during the two different ontogeneses of the colonial ascidian *Botryllus schlosseri*. By following the sequential expression patterns of myogenic TFs throughout a complete series of developmental stages, we inferred the putative cellular precursors for body and heart muscles. We show that, within a single chordate species, the myogenic transcriptional motifs are only partially co-opted and cellular origin and transcriptional regulation that lead to adult muscles are coordinated differently during embryonic and non-embryonic developmental processes.

### Colonial and solitary embryogenesis/metamorphosis share myogenetic motifs

Comparative analyses of expression patterns of myogenic TFs during embryogenesis showed that several genes are conserved between *Botryllus* and the solitary species studied so far [44–47], suggesting that core elements of the myogenic regulatory cascade of solitary species are conserved during the sexual development of colonial species. For instance, the maternal expression of the posterior determinant and muscle specifier *Zic*-*r.a* (*Macho*-*1*) [44, 48] in *B. schlosseri,* as well as its target *Tbx6*, indicates that the upstream determinants governing ascidian muscle development share the same expression as that reported for solitary forms. *Ebf* is also expressed during embryogenesis in *B. schlosseri*, and *Mesp* showed some expression despite the fact that it was not detectable by ISH, which we attribute to a temporal restriction of *Mesp*. Our data support the observation of Ricci et al. [33], showing that the early cleavage pattern seems to be conserved between solitary and colonial ascidians, at least for *B. schlosseri*. These data confirm the robustness of the ascidian expression and cleavage patterns across solitary and colonial species despite large variation in egg size and strongly suggest that the developmental mechanisms responsible of larval and post-metamorphic myogenesis might be well conserved in the whole class.

### Differentiated muscle cells share the same molecular architecture into oozooid and blastozooid

In *Botryllus*, the body and heart muscles are formed anew, starting from the post-metamorphic oozooid and in every adult blastozooid during each blastogenetic cycle [20, 24]. Regardless their different cellular origins and their divergent ontogenies, the oozooid and the blastozooid of *Botryllus schlosseri* present a similar arrangement of body structures, tissues, and cell types [32]. A few differences among the two zooid types include differences in body size (the oozooid generally being bigger than the blastozooid), differences related to the architecture of the branchial basket [34, 49], and the organization of the musculature, e.g., the number of muscle fibers varies as well as their arrangement in the atrial siphon [24]. Despite the observed variation in muscle organization, both oozooid and blastozooid muscle cells expressed the same myosins in their fully differentiated fibers, *i.e*., *Myh3* in the entire body wall musculature and *Myh2* in the heart. These results confirm that muscles of solitary and colonial ascidians express the same genes during the final differentiation of muscle cells. Furthermore, within distinct zooids of the same colonial species, different developmental trajectories lead to a similar differentiation process and similar types of muscles.

### Myogenic regulatory factors are only partially co-opted during blastogenesis

The transcriptomic profiles of the colony across different blastogenic stages showed the presence of only a subset of the myogenic TFs engaged during ascidian embryogenesis. With the exception of *Zic*-*r.a*, upstream regulators of embryonic myogenesis, such as *Tbx6*, *LIM (Lhx3), ZicL, Mesp*, and *Hand*-*r,* are not expressed in blastogenesis. During *Botryllus* embryogenesis, *Zic-r.a* is expressed in the neural plate, and during blastogenesis it is expressed in the dorsal tube (Additional file 12: Fig. 10). *Zic*-*r.a* expression in blastogenesis suggests a neurogenic rather than a myogenic function of *Zic*-*r.a* [50]. For instance, its expression does not seem linked to *Tbx6*, one of its downstream myogenic targets, which is absent in blastogenesis. This neurogenic role has been described in *Ciona* where, besides its function in early myogenesis, *Zic*-*r.a* is also zygotically expressed during neurogenesis, as suppressor of notochord fate [51]. The direct development of the bud, which lacks a larval stage with a notochord and tail musculature, may explain the absence of the early muscle transcriptional module. However, a lack of myogenic upstream regulators did not prevent the expression of late myogenic TFs, which are co-opted during blastogenesis. Precisely the same way that TFs are expressed in the TVCs of the *Ciona* larva throughout cardiopharyngeal development [40]. These results suggest a degree of plasticity in the regulation of myogenic transcriptional modules in ascidians, which can be decoupled from the control of maternal determinants and early zygotic transcriptional regulators.

### De novo origin of musculature in blastogenesis

In blastogenesis, the body muscle fate seems to be regulated by a kernel of genes that are expressed in the dorsal domain of the developing bud. In particular, *Tbx1/10 *+ and *Ebf *+ cells are localized in a set of mesenchymal cells between the dorsal tube and the epidermis. These cells have been previously described in *Botryllus* and *Diplosoma* as neural precursors, which migrate and cluster forming the cerebral ganglion [52, 53]. However, the sequential expression—both temporal and spatial—of *Tbx1/10, Ebf,* and *Mrf* during bud development suggests that these cells migrate from the dorsal tube toward the lateral mantle, aligning where the future muscle fibers form. There precursors might end up in the circular musculature of both siphons, as well as in the longitudinal muscle of the mantle. Therefore, in addition to center of neurogenesis, the dorsal tube could also have an additional role in myogenesis. In *Ciona,* the oral siphon muscles do not derive from TVCs but from a population of TLCs, where *Ebf* is first expressed regulating downstream expression of *Tbx1/10* [30, 31]. Without proper functional tests to dissect the interactions between *Ebf* and *Tbx1/10,* we cannot conclude whether the oral siphon musculature network of solitary ascidians is retained during blastogenesis. The presence of muscle stem cells cannot be completely ruled out due to the lack of detection of *Mrf*-*/bHLH*-*tun *+cells by FISH in the blastozooids, because the colony transcriptomes revealed an expression of *bHLH*-*tun*. It remains unclear whether a population of muscle precursors exists, and if such a population is renewed every blastogenic cycle or can persist over blastogenetic generations.

Another domain of expression of *Tbx1/10*–*Ebf*–*Mrf* is located in a dorsal part of the branchial basket epithelium. In this domain, the temporal sequence of gene expression is different. *Eb*f is detected in the overhead mesenchymal cells during later developmental stages, and surprisingly *Mrf* is expressed before *Ebf*. The deviating patterns of expression of these genes as a result of heterochrony are suggestive of cell-independent behaviors that occur at the site where the intersiphonal muscles will form. The intersiphonal muscles are the last muscles to form in the *Botryllus* blastozooid and connect longitudinally to the two siphons [24]. The expression of *Tbx1/10, Ebf,* and *Mrf* suggests a putative myogenic source in the dorsal epithelial domain, likely linked to the specification of intersiphonal muscles. However, without live tracking the dynamic nature of muscle formation cannot be fully understood, and we cannot rule out that the *Mrf *+ cells originate from other domains. Because muscle cell fates from these precursors have not been reported in *Ciona*, nor could we detect any of such muscle fates in other closely related styelid species [54] (Additional file 13: Fig. 11), the evidence so far suggests a single origin of myogenic precursor cells in the dorsal domain of buds as a synapomorphy of the Botryllidae.

### During blastogenesis heart muscle origin is possibly uncoupled from body muscles

During blastogenesis, no evidence showed any morphological recapitulation of embryonic heart development, i.e., no ventral fusion of bilaterally located heart progenitors [28]. Instead, the heart either originates from mesenchymal precursors that cluster in the ventrolateral side of the forming zooid [20, 55] or arises in the same area from the evagination of the branchial chamber [56]. The heart lineage markers, *Foxf, Nk4, Gata4/5/6* [33] as well as TFs *Tbx1/10* are expressed in the ventrolateral portion of the branchial chamber, where the cardiac muscle forms. Particularly the late *Nk4* expression is restricted exclusively to the myocardium. While it remains difficult to live-track heart precursor cells and to find their origin, a correlation between the hierarchy of TFs expression and blastozooid organogenesis suggests that the heart is specified in situ in the ventrolateral heart domain. The early expression of *Tbx1/10* together with *Nk4* that leads to *Nk4* and *Gata4/5/6* expression in the heart primordium resembles the formation of the second heart field [40]. However, the lack of functional relationships between TFs and the dramatically different ontogeneses suggests again a potential reshuffling of the embryonic cardiac module.

**Fig. 5:**
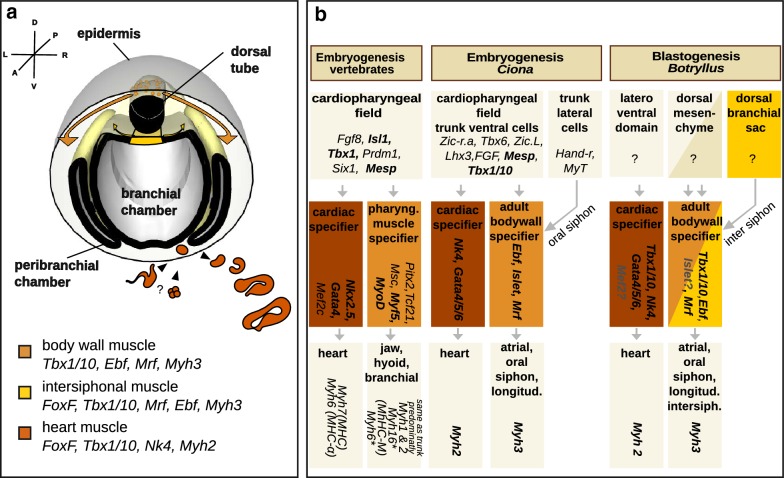
Proposed model showing a modular nature of muscle development. **a** Proposed model for *Botryllus schlosseri* muscle development. Three myogenic regions are depicted: the body wall muscles are derived from mesenchymal cells that evaginated from the dorsal tube, the intersiphonal muscles from the posterior branchial chamber, and the heart formed by either evagination or clustering of mesenchymal cells in the ventrolateral region. The myocardium is formed by invagination; hence, the two-layered tubular heart of the adult is put in place. **b** Comparison of the cardiopharyngeal muscle network in chordate development. The expression of genes has been assayed in multiple vertebrates [3, 64–67], *Ciona intestinalis* [31, 40] and *Botryllus schlosseri*. A number of similarities characterize the inductive signals specifying the cardiopharyngeal field in vertebrate and ascidian embryogenesis, while in blastogenesis a common origin of body wall muscles and heart muscles is unlikely. The cardiac lineage expresses *Nk2.5* and *Gata4* in the forming vertebrate hearts as well as their orthologs in both ascidian species. For what concern *Mef2* expression no information is currently available in ascidians [68]. An ortholog to vertebrate *Mef2* is present in the transcriptomic dataset of blastogenesis. Pharyngeal muscle starts differentiation by activating the paralogues *Myf5* and *MyoD*; only one ortholog, *Mrf*, is found in ascidians. To activate such in ascidians, *Ebf* is expressed in the body muscle lineage. Two myosin heavy chain forms characterize the vertebrate heart. Pharyngeal muscles do not express the same isoforms in all vertebrates: ruminants and rodents express the same myosins as in trunk and limb, namely, *Myh1* and Myh*2*; in other animals *Myh6* and *Myh16* can be expressed in addition. In ascidians, *Myh*2 is expressed in the heart, and *Myh3* in the body wall. Bold text indicates conserved expression within at least two species, gray only transcriptomic data, asterisk only in some species

Based on the correlation between patterns of expression and morphogenesis presented here, we propose a model which implies the presence of three putative myogenic domains during *Botryllus* blastogenesis (Fig. 5a): (1) muscle precursors delaminate from the dorsal tube and migrate along the mantle to form the circular muscles of the siphons and body wall muscles; (2) intersiphonal muscles originate from a dorsal portion of the branchial chamber and seem to be regulated in a different way from the other body wall muscles; and (3) the heart is formed from another population of founder cells localized in a ventrolateral domain of the bud, which remains poorly characterized. While the origin of muscle cells in *B. schlosseri* blastogenesis is still elusive, specifiers of cardiac and adult body wall muscles appear partially co-opted from embryogenesis (Fig. 5b).

### Methods

#### Animal husbandry

*Botryllus schlosseri* colonies were raised on a 50 × 70 × 1 mm glass slides as described previously [57]. A *Botryllus* colony consists of three coexisting asexual generations: the adult filtering zooids, their buds, called primary buds, and the secondary buds (or budlets), sprouting from the primary buds (Fig. 1). Budding (blastogenesis) was staged according to Lauzon et al. (2002) [58]. First, a budlet appears as thickening of the peribranchial chamber and overlying epidermis of the adult zooid (stage A) thus closes off forming a double vesicle connected with the parental zooid by the epidermis (stage B). Then, organogenesis begins and the inner vesicle separates into three chambers: one central branchial and two lateral peribranchial chambers (stage C). Staging of the development of the embryo has been done according to Conklin [59].

#### Embryo harvesting and dechorionation

Embryos were harvested at different developmental stages from the colony by opening the *Botryllus* adults with a syringe. Dechorionation was performed in fertilized eggs by shaking the eggs at 60RPM at room temperature in 0.2% trypsin and 20 mM TAPS and pH 8.2 in seawater for 1.5–2 h followed by several seawater washes. To calculate the cleavage time, non-dechorionated fertilized eggs have been harvested and kept in filtered seawater at 17 °C.

#### Gene identification and phylogenetic analyses

RNA sequences were retrieved by tblastn from the *Botryllus schlosseri* transcriptome database http://octopus.obs-lfr.fr/public/botryllus/blast_botryllus.php, full-length sequences of proteins were retrieved from Aniseed (https://www.aniseed.cnrs.fr/) and from NCBI. Alignments have been compiled using MAFFT, and sequence was trimmed by the TrimAl Gappyout method. Maximum likelihood trees were compiled using PhyML [60] (Additional files 14, 15, 16, 17: Fig. 12–15, Additional file 18: Supp Text 1).

#### *Fluorescent* in situ *hybridization (FISH)*

Primers for antisense mRNA probes were designed in the translated region of each gene (Additional file 19: Table 1) FISH was carried out as previously described in Ricci et al. (2016) with the following modifications: 1% dextran sulfate was added to the hybridization buffer and the revelation solution. The anti-digoxigenin antibody (HRP) (Roche, 11207733910) was pre-adsorbed for 1 h in hybridization solution with a mix of fixed colonies at different stages. When the tunic was exhibiting a very strong background, the animals were manually removed from x the tunic after rehydration, post-fixed in 4% PFA for 1 h, and transferred into washing baskets in 24-well plates. DIG probe detection was performed with bench-made FITC-tyramide by 3 h incubation. For double FISH, the hybridization of DIG-labeled and fluorescein-labeled probes was performed at the same time; fluorescein probes were detected with Cy3-tyramide. Single FISH experiments were conducted at least 3 times using different genotypes. For each experiment, an average of 15 zooids for each developmental stage has been screened in order to assess the consistency of the signals. For each experiment, negative controls (using sense probe) and positive controls have been performed. The ISH on embryos was performed after Christiaen et al. [61].

#### Transcriptional data analysis

Raw RNA-seq reads of seven stages of one unfertile colony of *B. schlosseri* SB802d were obtained from Rodriguez et al. [35] and quantified by pseudo-alignment via Kallisto [62] to the mixed-stage transcriptome database http://octopus.obs-fr.fr/public/botryllus/blast_botryllus.php. A heat map was generated using R; a cutoff for not expressed genes was chosen < 1 RPM, lowly expressed 1–100, and high expressed > 100 RPM (Additional file 7: Fig. 7, Additional file 20: Table 2). Orthologs of muscle determinants have been selected by reciprocal blast with published data from NCBI. For some candidate genes, the orthology has been also assessed by phylogenetic analyses (Additional files 14, 15, 16, 17: Fig. 12–15, Additional file 18: Text 1).

#### Imaging

Imaging of the NBT/BCIP ISH has been acquired with Zeiss Axio Imager A2 with a 20 × magnification, DIC, and color camera. Confocal stacks and movies (Additional files 9, 10: Movie 1, 2) were acquired a Leica SP8 (20 × or 40 ×/1.1 Water WD 0.6 HCX PL APO CS2) and processed with ImageJ and Inkscape. While the FISH protocol adopted has been shown to produce low background noise, the analyses of potential non-specific signals have been performed by saturating the output signals via both increasing the “gain” and the power of the exciting laser (Additional file 21: Figure 16).

## Additional files

**Additional file 1.** **Figure 1:** *Botryllus schlosseri* Myosin heavy chain gene tree (maximum likelihood).

**Additional file 2.** **Figure 2:** *Myh1* expression in larva and during metamorphosis.

**Additional file 3.** **Figure 3:** Myh3 expression in *B. schlosseri* oozooid.

**Additional file 4.** **Figure 4:** *Myh2* expression in swimming larva and oozooid.

**Additional file 5.** **Figure 5:** Embryonic development *Botryllus schlosseri*.

**Additional file 6.** **Figure 6:** Expression of myogenic factors in early embryo.

**Additional file 7.** **Figure 7:** Components of myogenic motif in the transcriptomes of entire colonies at different blastogenetic stages.

**Additional file 8.** **Figure 8:** Detailed expression pattern of *Ebf* and *Tbx1/10*.

**Additional file 9.** **Movie 1:** Expression of *Tbx1/10* in stage C2 secondary bud.

**Additional file 10.** **Movie 2:** Colocalization of *Ebf* (magenta) and *Myh3* (yellow) expression.

**Additional file 11.** **Figure 9:** Expression of *bHLH-tun* in oozooid.

**Additional file 12.** **Figure 10:** *Zic-r.a* expression in embryo, oozooid, and blastozooid.

**Additional file 13.** **Figure 11:** Phalloidin in *Polyandrocarpa zorritensis* (Styelidae) adult, showing the absence of intersiphonal muscle bands.

**Additional file 14.** **Figure 12:** ML tree of Nk4.

**Additional file 15.** **Figure 13:** ML tree of MRF.

**Additional file 16.** **Figure 14:** ML tree of Mesp.

**Additional file 17.** **Figure 15:** ML tree of Ebf.

**Additional file 18.** **Text 1:** Sequences used for phylogeny.

**Additional file 19.** **Table 1:** Primer sequences.

**Additional file 20.** **Table 2:** Correlations coefficient of transcriptomic data for candidate genes > 1.

**Additional file 21.** **Fig. 16:** Examples of recurring background.
