# Supplementary figures and images for: Modular co-option of cardiopharyngeal genes during non-embryonic myogenesis
Source: EvoDevo. 2019 Mar 5;10:3. doi: 10.1186/s13227-019-0116-7 (PMC6399929; doi:10.1186/s13227-019-0116-7)

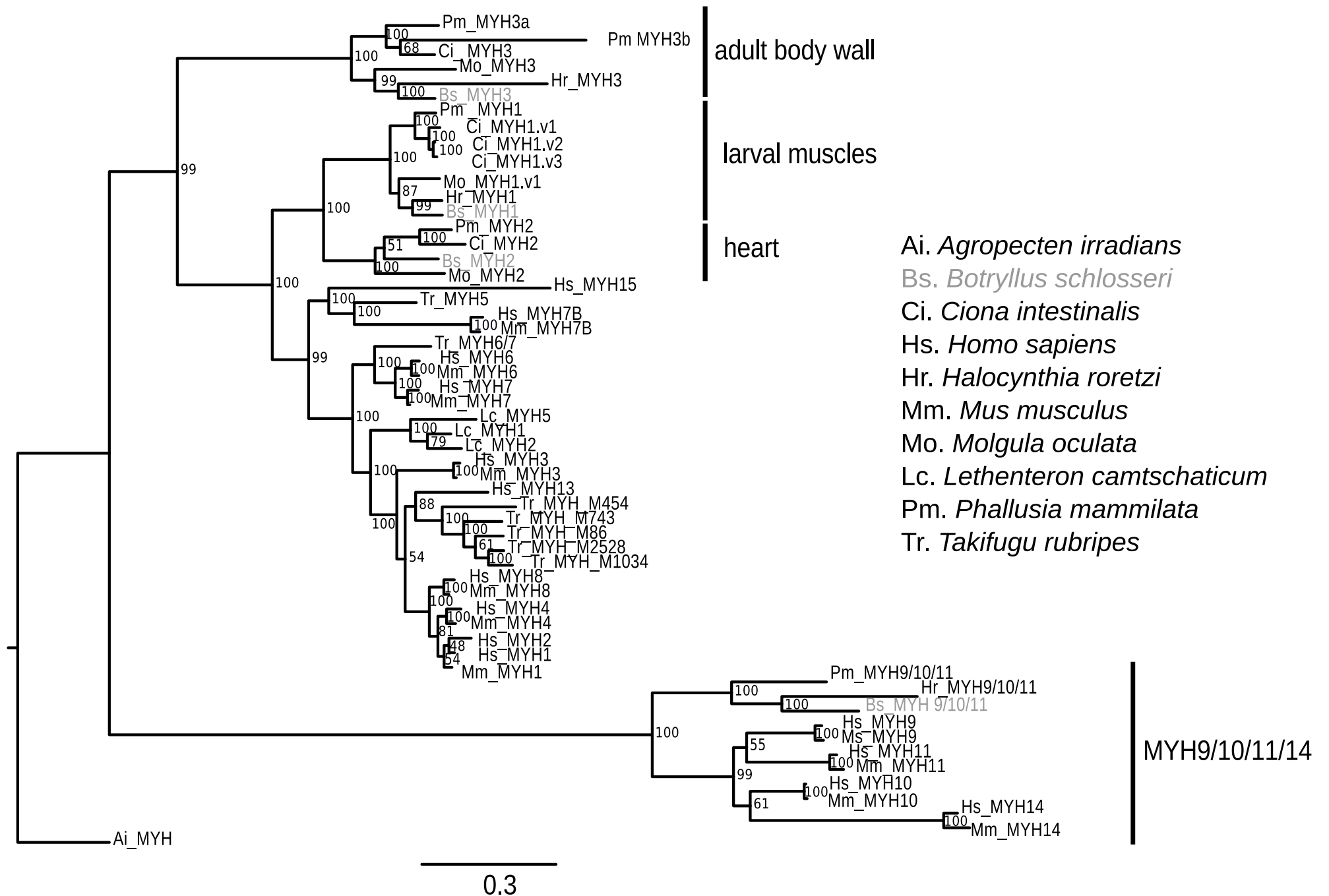

ML tree, LG, 100 bootstrap

Supplement: Supplementary file 1 — Additional file 1. Figure 1: Botryllus schlosseri Myosin heavy chain gene tree (maximum likelihood). [file 13227_2019_116_MOESM1_ESM.pdf]

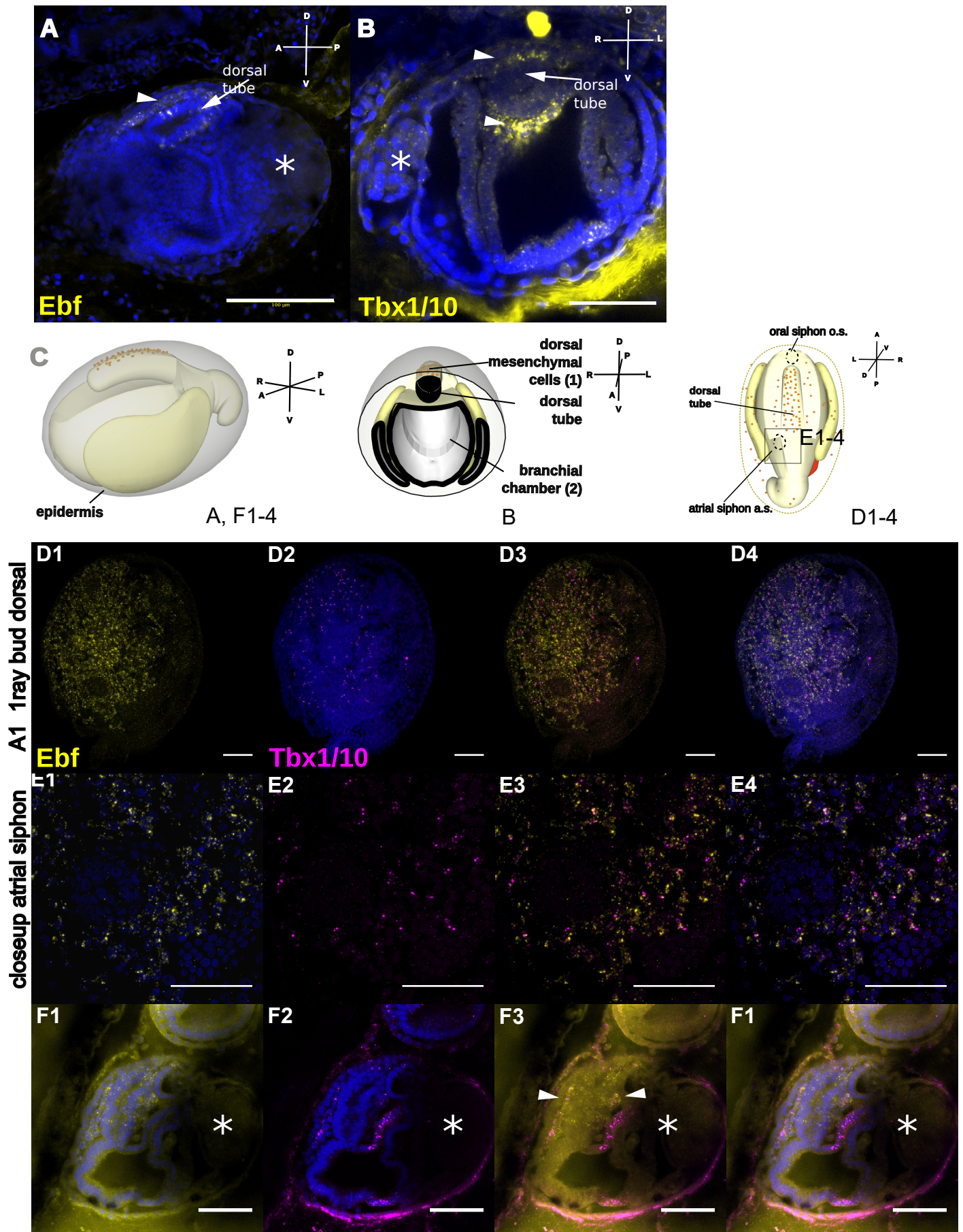

Supplement: Supplementary file 8 — Additional file 8. Figure 8: Detailed expression pattern of Ebf and Tbx1/10. [file 13227_2019_116_MOESM8_ESM.pdf]
