# Supplementary material for: Modular co-option of cardiopharyngeal genes during non-embryonic myogenesis
Source: EvoDevo. 2019 Mar 5;10:3. doi: 10.1186/s13227-019-0116-7 (PMC6399929; doi:10.1186/s13227-019-0116-7)
Supplement: Supplementary file 2 — Additional file 2. Figure 2: Myh1 expression in larva and during metamorphosis. [file 13227_2019_116_MOESM2_ESM.pdf]

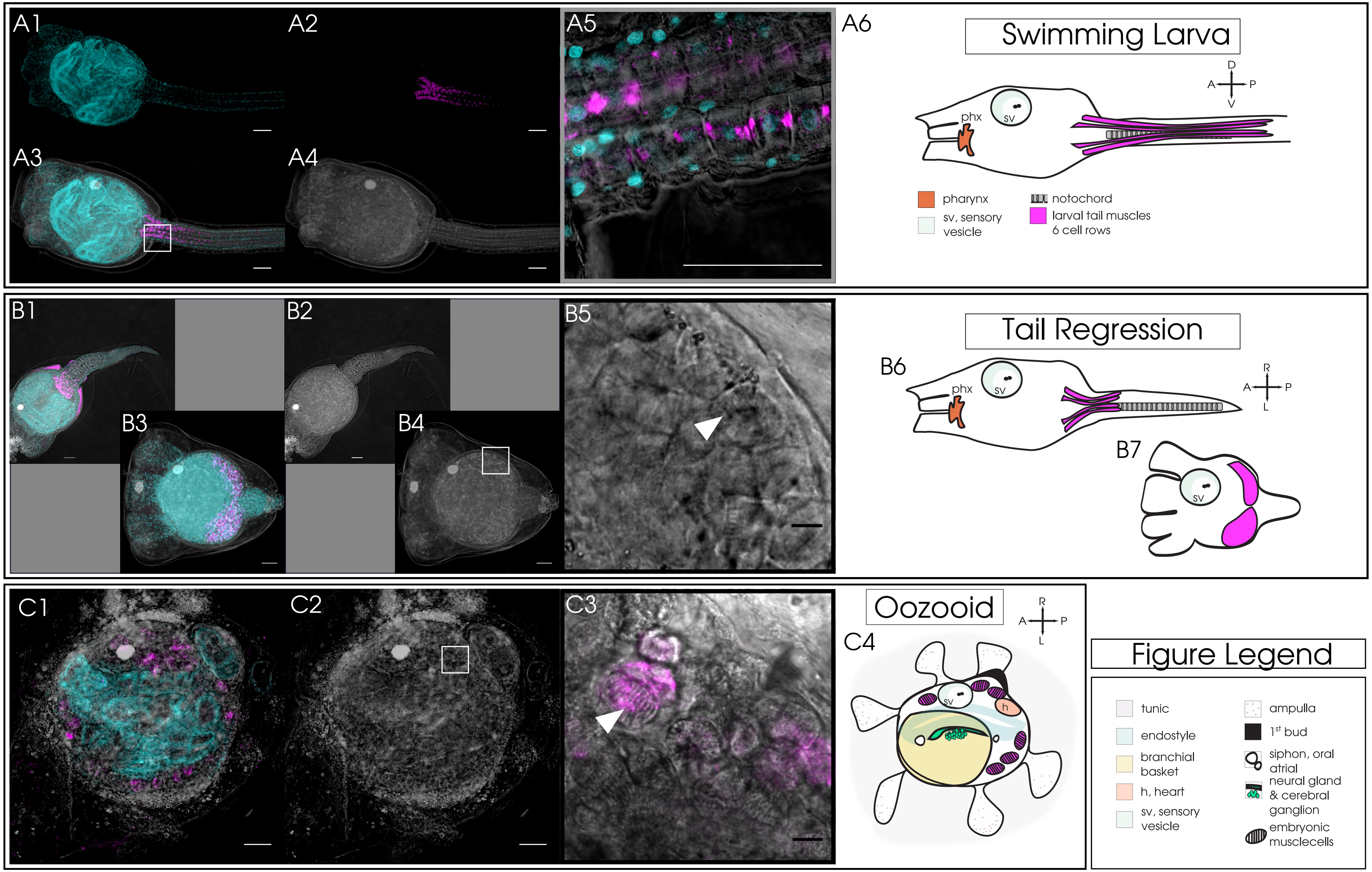

Supp. Fig. 2. Expression of *Myh1* in (A1-A6) swimming larvae, (B1-B5) during tail regression, and (C1-C4) in the metamorphosized sexually developped young oozooid. (B5, C3) Arrowheads pointing to example of embryonic muscle cell that express *Myh1* untill early oozooid. Hoechst (cyan), *Myh1* (magenta), bright field (grey). Scale bar 50 micron.
