# Supplementary material for: Modular co-option of cardiopharyngeal genes during non-embryonic myogenesis
Source: EvoDevo. 2019 Mar 5;10:3. doi: 10.1186/s13227-019-0116-7 (PMC6399929; doi:10.1186/s13227-019-0116-7)

Early Oozooid

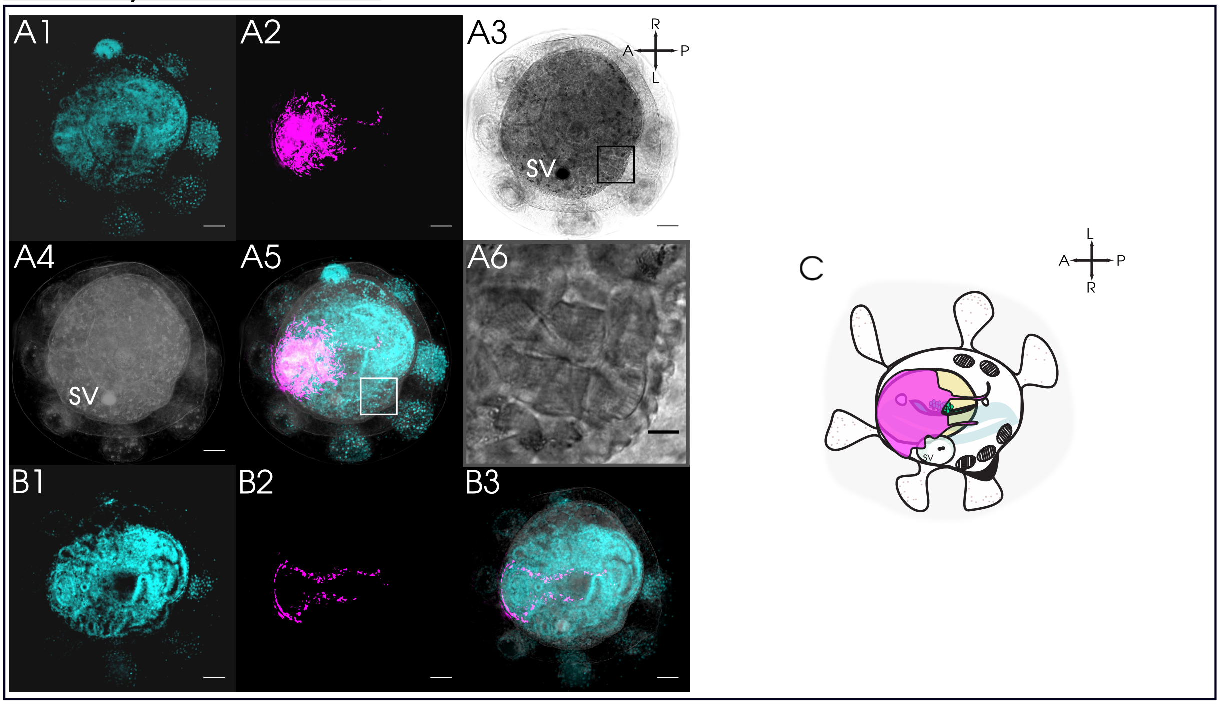

Late Oozooid

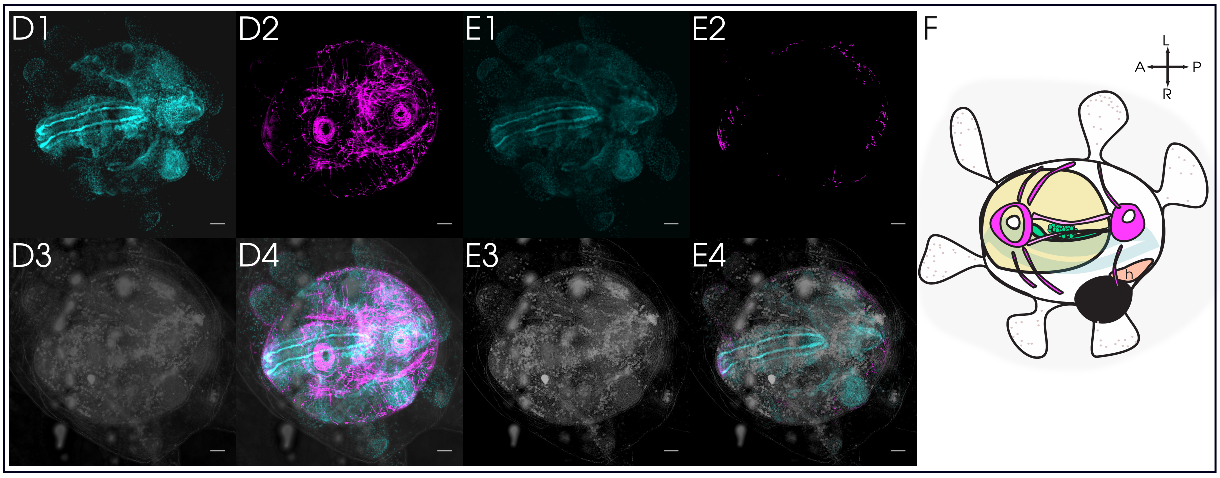

Supp. Fig. 3. Expression of Myh3 in the oozooid. (A1-A5) Confocal projections of an early oozooid in three channels and their overley. (A6) Details of larval regressing muscle. (B1-B3) Selected Z-stacks highlighting the forming intersiphonal muscles. (D1-D4) and (E1-E4)Confocal projections of a fully developped oozooid in three channels and their overley in two Z level respectively. Hoechst (cyan), Myh3 (magenta), bright field (grey). SV: sensory vesicle. Scale bar 50 micron.

Figure Legend

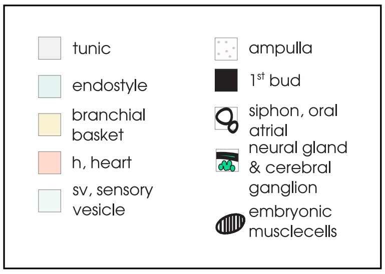

Supplement: Supplementary file 3 — Additional file 3. Figure 3: Myh3 expression in B. schlosseri oozooid. [file 13227_2019_116_MOESM3_ESM.pdf]
