# Supplementary material for: Modular co-option of cardiopharyngeal genes during non-embryonic myogenesis
Source: EvoDevo. 2019 Mar 5;10:3. doi: 10.1186/s13227-019-0116-7 (PMC6399929; doi:10.1186/s13227-019-0116-7)

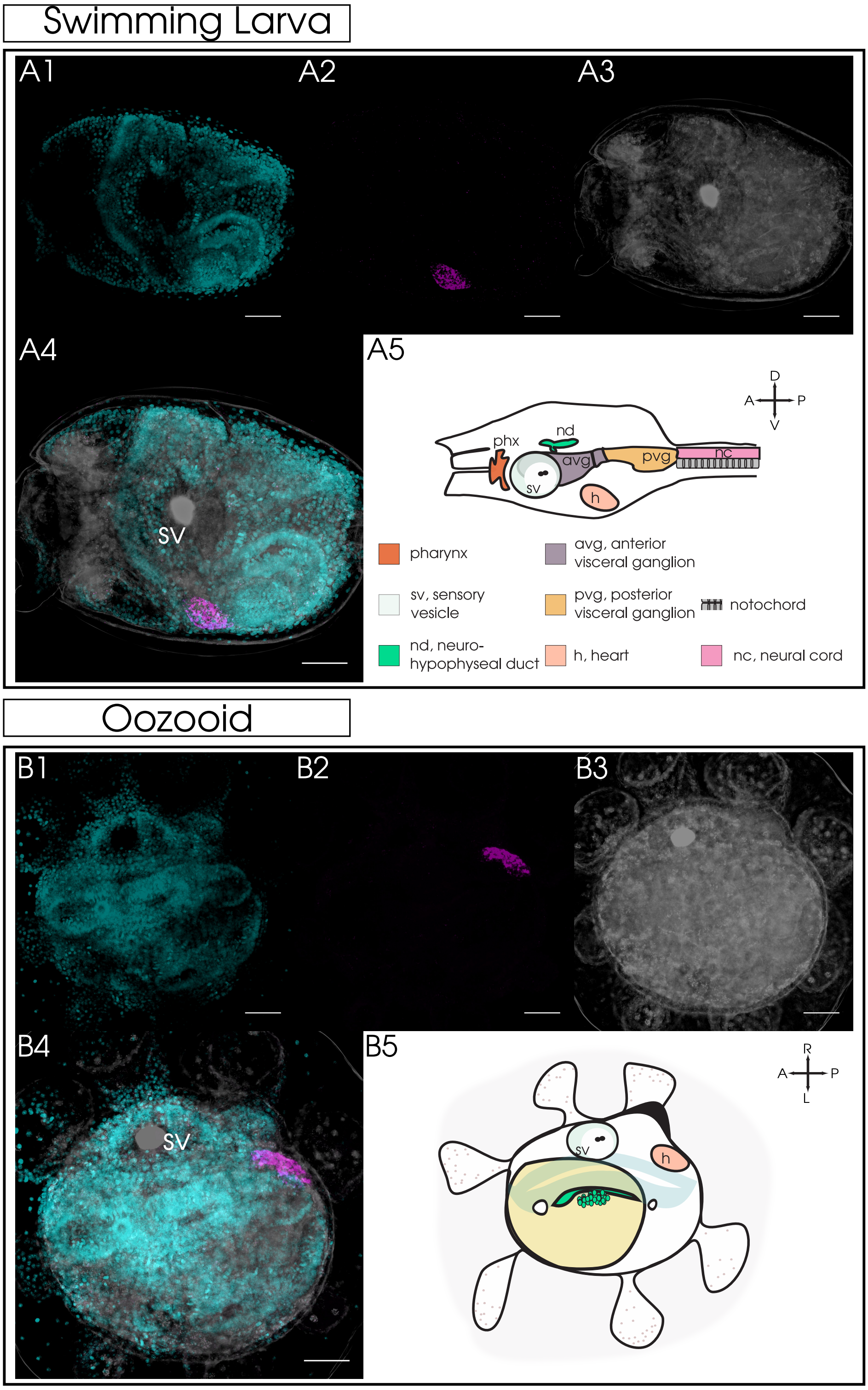

Supp. Fig. 4. Expression of Myh2 in the swimming larvae and in the oozooid. (A1-A4) Confocal projections in three channels and their overley. (B1-B4) Confocal projections of a fully developped oozooid in three channels. Hoechst (cyan), Myh2 (magenta), bright field (grey). SV: sensory vesicle. Scale bar 50 micron.

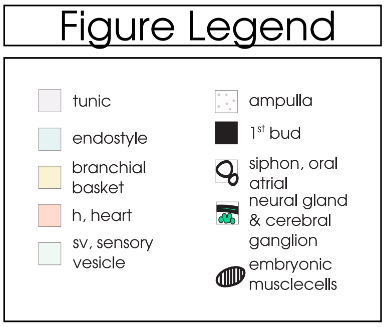

Supplement: Supplementary file 4 — Additional file 4. Figure 4: Myh2 expression in swimming larva and oozooid. [file 13227_2019_116_MOESM4_ESM.pdf]
