# Supplementary material for: Modular co-option of cardiopharyngeal genes during non-embryonic myogenesis
Source: EvoDevo. 2019 Mar 5;10:3. doi: 10.1186/s13227-019-0116-7 (PMC6399929; doi:10.1186/s13227-019-0116-7)
Supplement: Supplementary file 5 — Additional file 5. Figure 5: Embryonic development Botryllus schlosseri. [file 13227_2019_116_MOESM5_ESM.pdf]

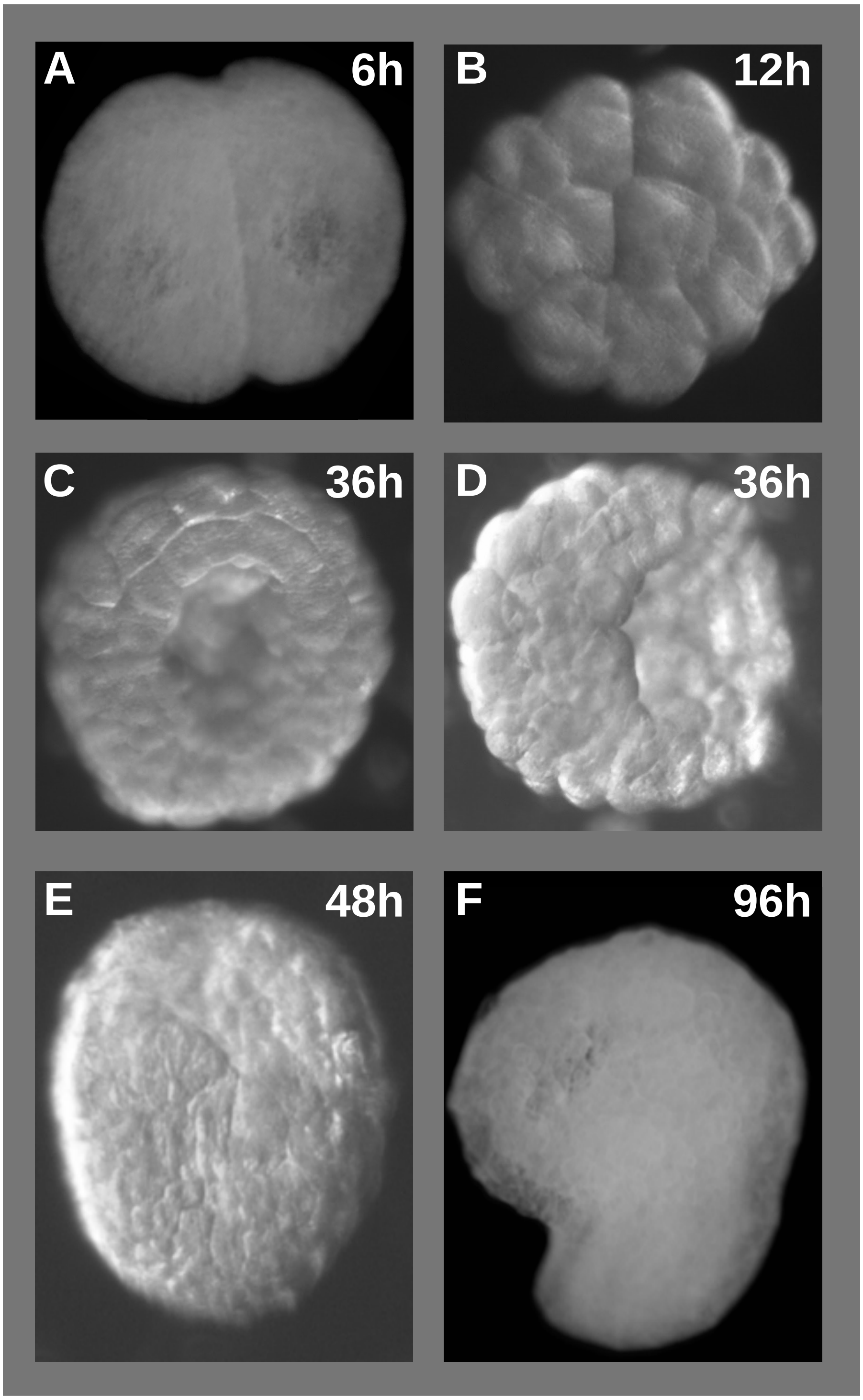

Fig 5 . Embryogenesis of *Botryllus schlosseri* . Embryos are dechorionated. (A) 2-cell embryo, (B) 32-cell embryo, (C-D) Gastrula, (E) Neurula (F) early tailbud.
