# Supplementary material for: Modular co-option of cardiopharyngeal genes during non-embryonic myogenesis
Source: EvoDevo. 2019 Mar 5;10:3. doi: 10.1186/s13227-019-0116-7 (PMC6399929; doi:10.1186/s13227-019-0116-7)
Supplement: Supplementary file 6 — Additional file 6. Figure 6: Expression of myogenic factors in early embryo. [file 13227_2019_116_MOESM6_ESM.pdf]

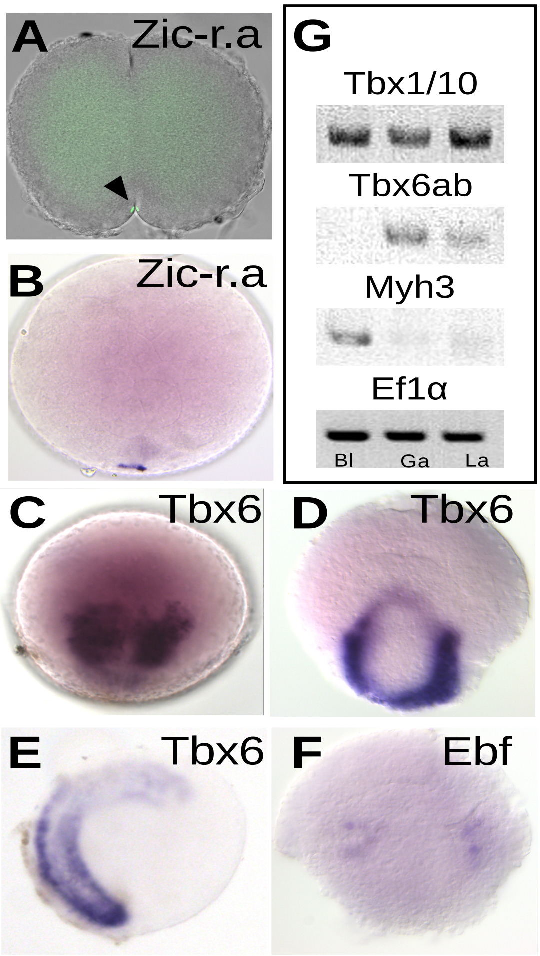

Supp. Fig. 6. (A-B) Expression of Zic-r.a and at 2 cells stage and 36 cells stage. (C-E) Tbx6 expression in(C) 64-cell (D) early gastrula and (E) larval tail. (F) Ebf expression at gastrula stage. Tbx1/10,

Tbx6 (ab), Myh3 and Ef1α (control), in mixed RNA of all 7 stages of blastogensis, infertile (Bl), gastrula (Ga) and at larval stage (La). Tbx6 sequence stretched over 2 contigs in the assembly of the transcriptome (ab), hence the in situprobe was designed containing parts of both.
