# Supplementary material for: Modular co-option of cardiopharyngeal genes during non-embryonic myogenesis
Source: EvoDevo. 2019 Mar 5;10:3. doi: 10.1186/s13227-019-0116-7 (PMC6399929; doi:10.1186/s13227-019-0116-7)
Supplement: Supplementary file 7 — Additional file 7. Figure 7: Components of myogenic motif in the transcriptomes of entire colonies at different blastogenetic stages. [file 13227_2019_116_MOESM7_ESM.pdf]

A

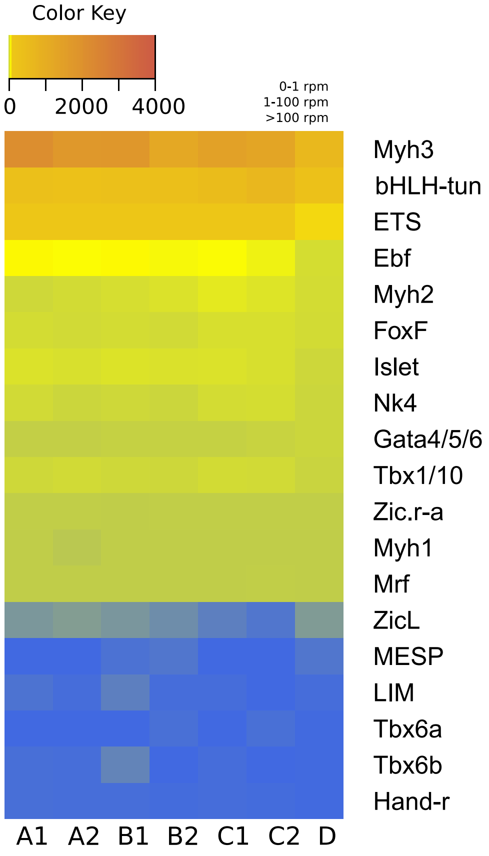

B

Gene	A1	A2	B1	B2	C1	C2	D
bHLH-tun	338.1832344448	334.4823776686	345.952430341	407.8792000956	494.0000000000	654.6019575702	299.0759295378
Ebf	56.4600073649	48.4133773784	55.6827886519	44.3695326785	47.0525686183	38.410017002	17.4087550631
ETS	104.0025480018	117.3867153252	132.2294652458	121.7097877807	135.5831727255	139.0011248496	85.2616872523
FoxF	16.7980187201	14.9624020638	16.7002611816	14.1427885413	19.2286391529	19.9163051122	15.9961617265
Gata4/5/6	3.9402759961	3.9402759961	5.0510019706	5.2617261257	5.6848463744	7.9750116302	10.0040426600
hand-r	0.0646311636	0.0538896136	0.0551139166	0.037293309	0.0443056202	0.0390797988	0.0196408153
Islet	22.1381296096	20.4422626923	23.6548904955	21.4452741279	22.684477526	19.4115139688	11.87997786
LIM	0.1036914736	0.047650962	0.2287707011	0.0462182632	0.0443056202	0.0000000000	0.045692223
MESP	0.0000000000	0.0000000000	0.0915082804	0.1386547896	0.0000000000	0.0000000000	0.1370766676
Mrf	1.3998348933	1.2865759736	1.2865759736	1.3726242067	1.7100757386	2.3038922487	1.7438239499
Myh1	1.2961434198	0.9530192397	1.6013949078	1.4327661594	1.1076405042	1.4684833262	1.9190733465
Myh2	12.0282264891	15.9630579702	18.7134433514	22.2772028656	29.7733767528	24.7347660264	16.9061223384
Myh3	2137.0294247695	1781.8600725287	1822.3416509599	1233.1032623555	1464.9653308522	1292.1735468851	649.4235589055
Nk4	14.2480898476	10.6610926782	12.318936222	10.1477280879	16.1688487185	17.8691934653	10.9788815388
Tbx1/10	12.6503597769	14.5335434059	12.7654051223	11.9243119073	15.1082164773	14.4553827427	8.5901378368
Tbx6a	0.0000000000	0.0000000000	0.0000000000	0.074586618	0.0000000000	0.0781595976	0.0196408153
Tbx6b	0.0646311636	0.0538896136	0.2755695828	0.0000000000	0.0443056202	0.0000000000	0.0196408153
Zic.r-a	2.3330581556	2.6684538713	1.8759197492	2.3109131603	2.3038922487	2.2486150933	2.1018422367
ZicL	0.4666116311	0.5241605819	0.4575414022	0.3697461057	0.2215281008	0.1376703118	0.5026144479

Supp. Fig. 7. Components of myogenic motif in the transcriptomes of entire colonies at different blastogenetic stages [A1-D].(A) Heat map representing the mRNA expression level of candidate genes for myogenesis along the seven successive stages of colony blastogenetic development.
Each stage of the colony include the transcripts expressed in the adult zooid, in the primary and in the secondary bud. Royal blue: RPM<1 (e.g. *Hand-r*), yellow: RPM 1>100 (eg.*Myh2*), coral: RPM >100 (eg. *Ets)*. (B) raw data in RPM.
