# Supplementary material for: Modular co-option of cardiopharyngeal genes during non-embryonic myogenesis
Source: EvoDevo. 2019 Mar 5;10:3. doi: 10.1186/s13227-019-0116-7 (PMC6399929; doi:10.1186/s13227-019-0116-7)

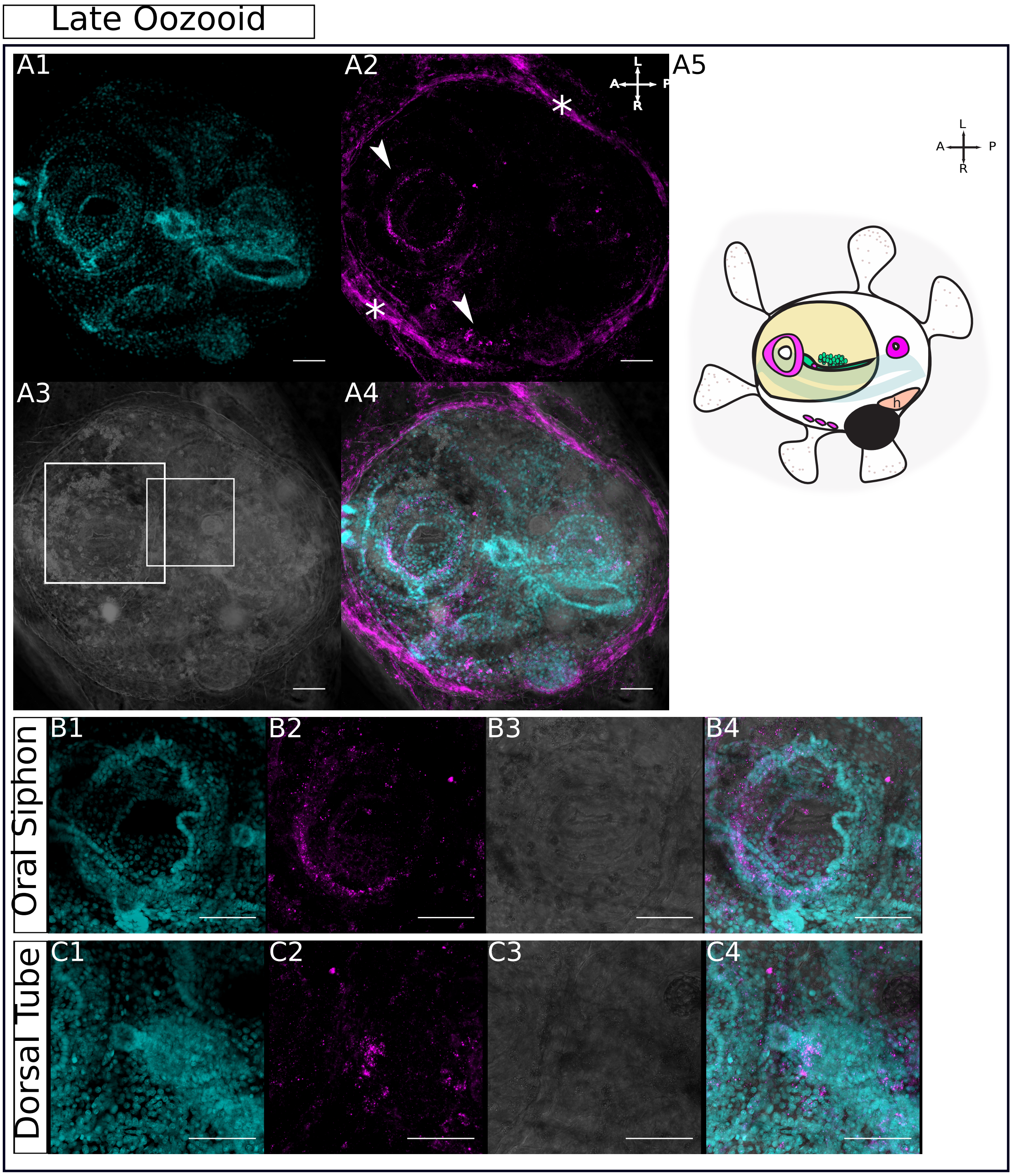

Supp. Fig. 9. Expression of bHLH-tun in the oozooid. (A1-A4) Confocal projections of an early oozooid in three channels and their overley. (B1-B4) and (C1-C4) Selected Z-stacks highlighting the oral siphon and the dorsal tube respectively. Hoechst (cyan), bHLH-tun (magenta), bright field (grey). Asterisk: unspecific stain in the tunic. Scale bar 50 micron.

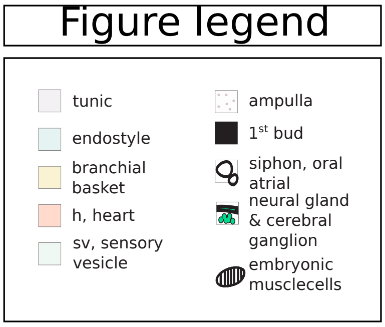

Supplement: Supplementary file 11 — Additional file 11. Figure 9: Expression of bHLH-tun in oozooid. [file 13227_2019_116_MOESM11_ESM.pdf]
