# Supplementary material for: Modular co-option of cardiopharyngeal genes during non-embryonic myogenesis
Source: EvoDevo. 2019 Mar 5;10:3. doi: 10.1186/s13227-019-0116-7 (PMC6399929; doi:10.1186/s13227-019-0116-7)
Supplement: Supplementary file 12 — Additional file 12. Figure 10: Zic-r.a expression in embryo, oozooid, and blastozooid. [file 13227_2019_116_MOESM12_ESM.pdf]

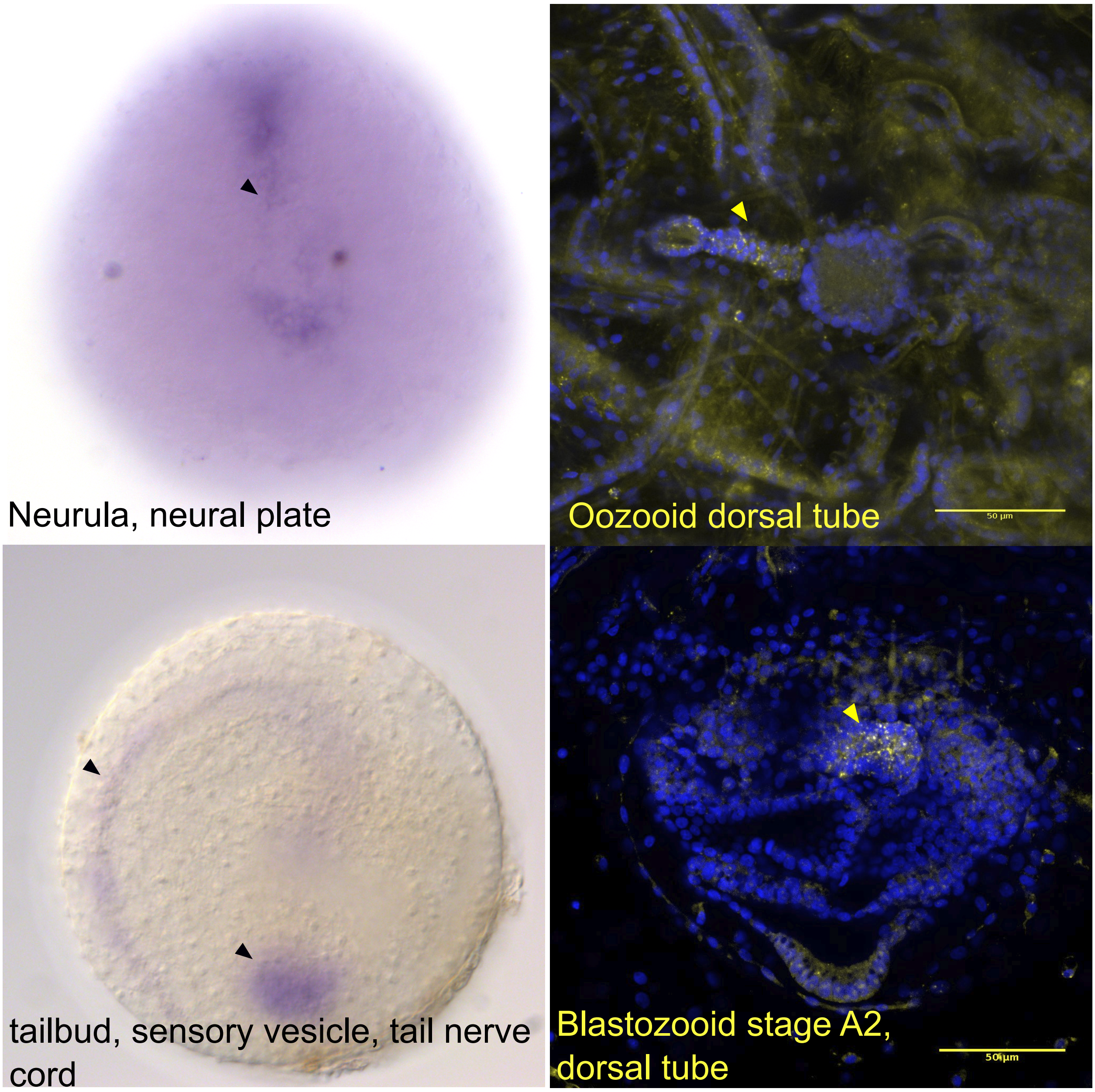

Supp. Fig. 10. Expression of *Zic-r.a* in embryo, oozooid and blastozooid.
