# Supplementary material for: Modular co-option of cardiopharyngeal genes during non-embryonic myogenesis
Source: EvoDevo. 2019 Mar 5;10:3. doi: 10.1186/s13227-019-0116-7 (PMC6399929; doi:10.1186/s13227-019-0116-7)
Supplement: Supplementary file 13 — Additional file 13. Figure 11: Phalloidin in Polyandrocarpa zorritensis (Styelidae) adult, showing the absence of intersiphonal muscle bands. [file 13227_2019_116_MOESM13_ESM.pdf]

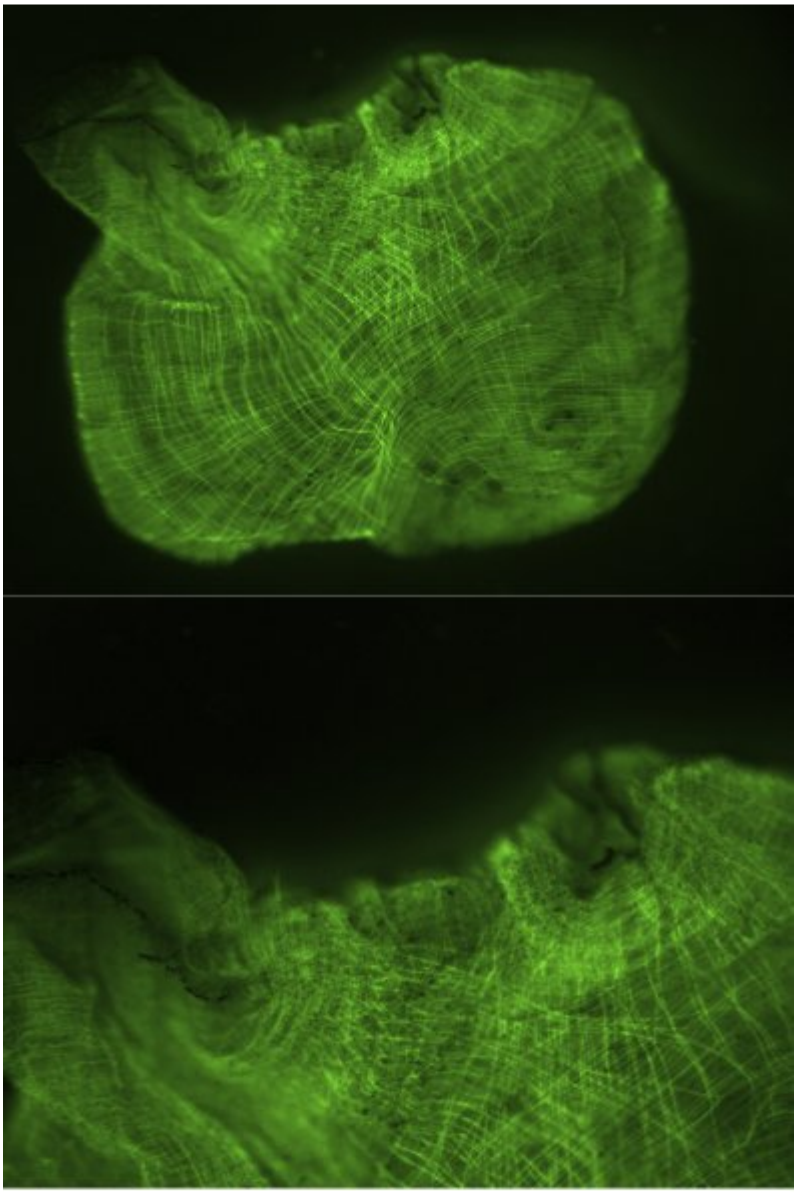

Supp. Fig. 11. Zooid of *Poliandrocarpa zorritensis* stained with phalloidin showing the muscle fibers and close up of intersiphonal zone.
