# Supplementary material for: Modular co-option of cardiopharyngeal genes during non-embryonic myogenesis
Source: EvoDevo. 2019 Mar 5;10:3. doi: 10.1186/s13227-019-0116-7 (PMC6399929; doi:10.1186/s13227-019-0116-7)
Supplement: Supplementary file 14 — Additional file 14. Figure 12: ML tree of Nk4. [file 13227_2019_116_MOESM14_ESM.pdf]

Nkx

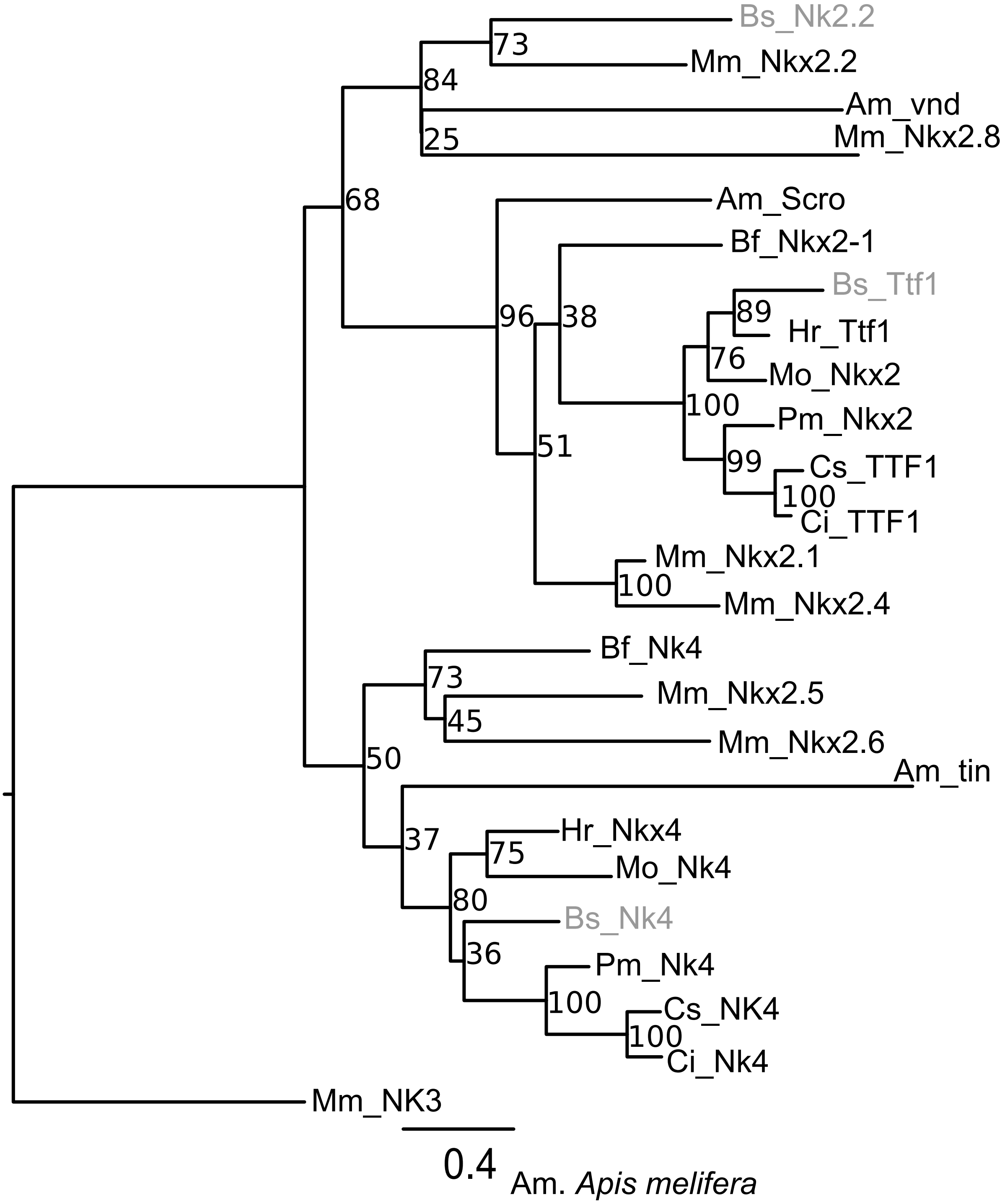

Am. *Apis melifera*
Bf. *Branchiostoma floridae*
Bs. *Botryllus schlosseri*
Ci. *Ciona intestinalis*
Cs. *Ciona savigny*
Hr. *Halocynthia roretzi*
Mm. *Mus musculus*
Mo. *Molgula oculata*
Pm. *Phallusia mammilata*

PhyML, LG, 100 bootstrap
