# Supplementary material for: Modular co-option of cardiopharyngeal genes during non-embryonic myogenesis
Source: EvoDevo. 2019 Mar 5;10:3. doi: 10.1186/s13227-019-0116-7 (PMC6399929; doi:10.1186/s13227-019-0116-7)
Supplement: Supplementary file 16 — Additional file 16. Figure 14: ML tree of Mesp. [file 13227_2019_116_MOESM16_ESM.pdf]

MESP

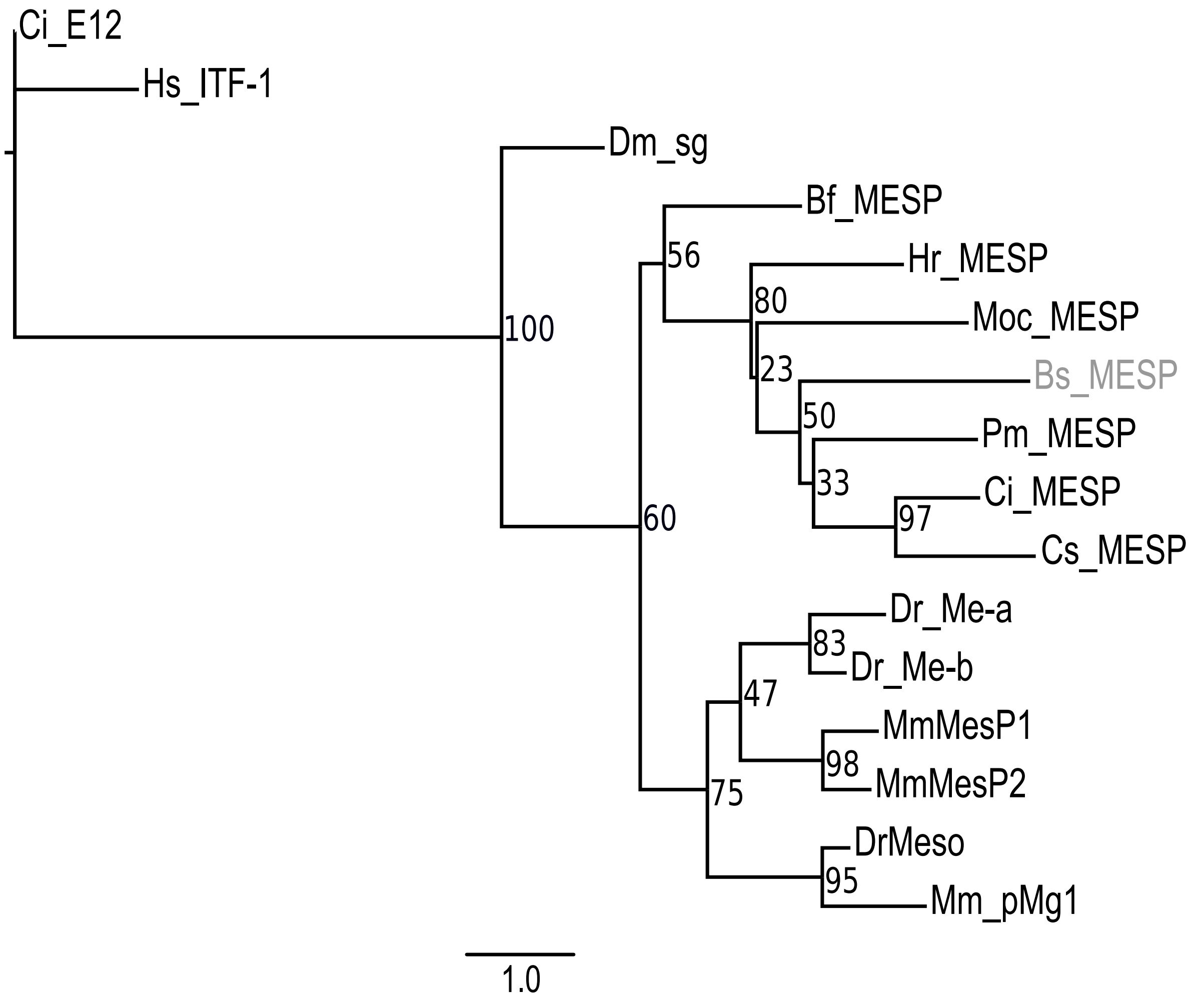

PhyML, LG, 100 bootstrap

Bf. *Branchistoma floridae*
Bs. *Botryllus schlosseri*
Ci. *Ciona intestinalis*
Cs. *Ciona savigny*
Dm. *Drosophila melanogaster*
Dr. *Danio rerio*
Hs. *Homo sapiens*
Hr. *Halocynthia roretzi*
Mm. *Mus musculus*
Moc. *Molgula occidentalis*
Pm. *Phallusia mammilata*
