# Supplementary material for: Modular co-option of cardiopharyngeal genes during non-embryonic myogenesis
Source: EvoDevo. 2019 Mar 5;10:3. doi: 10.1186/s13227-019-0116-7 (PMC6399929; doi:10.1186/s13227-019-0116-7)
Supplement: Supplementary file 17 — Additional file 17. Figure 15: ML tree of Ebf. [file 13227_2019_116_MOESM17_ESM.pdf]

Ebf /COE

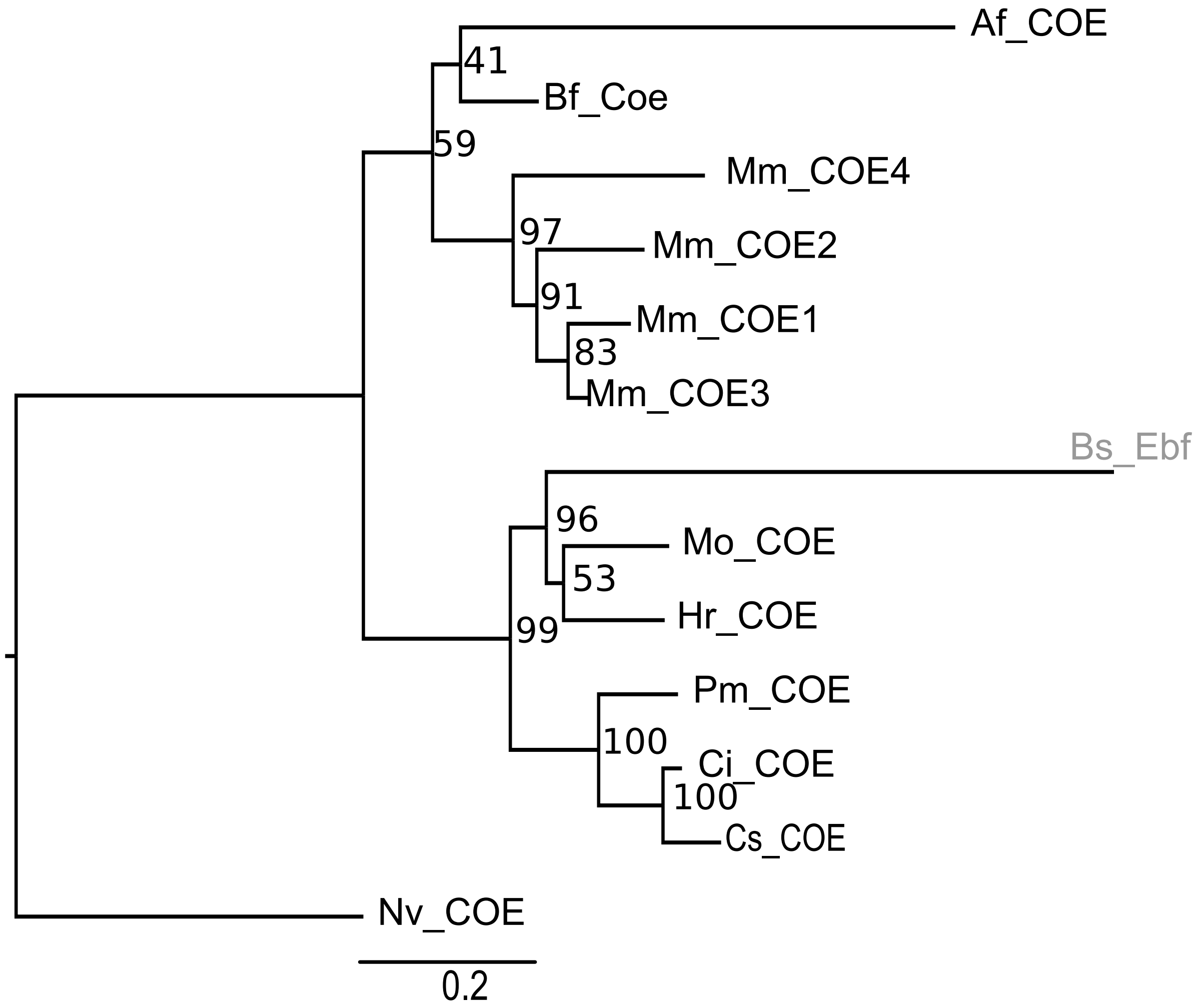

PhyML, LG, 100 bootstrap

Af. *Apis florea*
Bf. *Branchiostoma floridae*
Bs. *Botryllus schlosseri*
Ci. *Ciona intestinalis*
Cs. *Ciona savigny*
Hr. *Halocynthia roretzi*
Mm. *Mus musculus*
Mo. *Molgula oculata*
Pm. *Phallusia mammilata*
