# Supplementary material for: Modular co-option of cardiopharyngeal genes during non-embryonic myogenesis
Source: EvoDevo. 2019 Mar 5;10:3. doi: 10.1186/s13227-019-0116-7 (PMC6399929; doi:10.1186/s13227-019-0116-7)
Supplement: Supplementary file 18 — Additional file 18. Text 1: Sequences used for phylogeny. [file 13227_2019_116_MOESM18_ESM.docx]

#Ebf/COE

>Bs_Ebf

MAMIVPDFGSDMTSALRDQPMLPITGWMQTNLVEPIHMPQPGILRIHRAEFSIQPPKNLRKSNFFNFSLLFYDNRGLPARATKTEFKNFVEEASSEHCSGTEKANNGVHYEITFILSNGVSVLQDIYVRLVDAANRERIICYEGQDKNPEMRRVLLTHEIMCSRCCDHKSCGNRNETPSDPTFDKGQGPYDIKFFLKCNQNCLKNAGNPRDMRRFAVAISTDVDMKFPLAYSETMFVHNNSKHGRRAKTDLENGKRSSGPCITSVCPSEGWVTGGTTVIIVGNNFTRDMQVAFDTTIVFTEFVSEHALKIQSPGYHLTGPVEVTITIKNKQYCKNQPGRFTYTALNEPSIEYGFQRLMKMIPRHQADPLQISKEMILTRAAELLEAFSVRPYSQLQSAPPPPLQNSFTSTSSTMMASAMGGYNVPNQYAPFAAQDRMDSNGYSPHAGPQHSSNISLSSIGAVTNAPPYGSPVNGYVGPPTFTNMTNSSANMFCNAGLLPQSPNPAMNCLSGSGTTPGIFSFSPANMISAVKQKSAFAPVIRAHNTPSPNHSGVSETTIHEINGYT*

>Ci_COE
MATMAGPQLSGPAVRGWMQTTLVEPMPNGNVGLHRAHFEKQPPNNLRKSNFFHFVLALYDRQGQPVEIERSAFVGFVENETEIAGEKTNNGIQYRLQLLYHSGVRTEQDVFVRLIDSATKQSITYEGQDKNPEMRRVLLTHEIMCSRCCDKKSCGNRNETPSDPVVIDRYFLKFFLKCNQNCLKNAGNPRDMRRFQVVVSTTVHVDGHVLAVSDNMFVHNNSKHGRRARRVDPSEASPTIKAINPAEGWTTGGATVVIVGENFFDGLQVVFGSMVVWSELITQHAIRVQTPPRHLPGVVEVTLSYKNKQFCSGAPGRFVYTALNEPTLDYGFQRLLKTVPRHPGDPERLPKEIILKRAADVMEAVISRQYAPPSQMPPSAGITPPAPHLAAAPCAPPGSFVPQSASAAMAVAMNGYAAAAVSSQFGGTPDRFDTGSDSGYSRGNSVSPRNGYSPQTTPHSLNSGSIGSMVGLTTVGVVPAPAPYHCAPSFNSYSSASGPTFTNMNNTSPGLFSGSGIIPPSPHNGMNPLPSSGTTPGIFSFSPANMISAAKQKSAFAPVH
RPHNSPSPLAPSNGNIALNGYS
>Cs_COE
NMDGPQLPGSAVRGWMQTSLIEPMASGNVGLHRAHFEKQPPNNLRKSNFFHFVLALYDRQGQPVEIERSAFVGFIENETEIAGENTNNGIQYRLQLLYQNGLRTEQDVYVRLIDSATKQSITYEGQDKNPEMRRVLLTHEIMCSRCCDKKSCGNRNETPSDPVVIDRYFLKFFLKCNQNCLKNAGNPRDMRRFQVVVSTTVHVDGHVLAVSDNMFVHNNSKHGRRARRVDPSEASPMIKAINPAEGWTTGGATVVIVGDNFFDGLQVVFGSMVVWSELITQHAIRVQTPPRHLPGVVEVTLSYKNKQFCSGAPGRFVYTALNEPTLDYGFQRLLKTIPRHPGDPERLPKEIILKRAADVMEAVISRQYASPSQMHPSSGITPPTPHLGATPCPPSGSFVPQSASAAMAVAMNGYAAAAVSSQFVGNPDRFDNGSDSGYSRGNSVSPRNGYSPQTTPHSLNSGSIGSMVGLTTVGAVPAPYHGPPSFNSYSTSGPTFTNMNNSSPGIFPGSSIIPPHTHNGMNGLPSSGTTPGIFSFSPASMISAAKQKSAFAPVHRPHNSPSPMAPPNSSLVENSES

>M0_COE

MSNLPSQQLAPLRGWVQTGLVDTMPSAGLHRAHFEKQPPGNLRKSNFFHFVLALYDRQGQPVEVERTAFIDFVEGDREAVTATGEKTHNGIHYRLQLLYHNGVRTEQDLFVRLIDSVTKQAISYEGQDKNPEMRRVLLTHEIMCSRCCEKKSCGNRNETPSDPVVIDRYFLKFFLKCNQNCLKNAGNPRDMRRFQVVISTTVHVDGHVLAVSDNMFVHNNSKHGRRARRLEPTDATPVIKALSPSEGWTTGGATVIIVGDNFFDGLQVVFGSMIVWSELVTQHAIRVQTPPRHVPGVVEVTLSYKNKQFCKGSPGRFVYTALNEPTIDYGFQRLLKAIPRHPGDPERLPKEIVLKRAADVMEAVMTRPYNQVPAPPPAPMHNTFNGSSPAMMAAGVNTYNHSVQMAPSQYTITTPDRLDSANGSDSGKIIIDTTLGGYSRGSVSPKTGYSPQATPHSTANVGLSAVSGNAPPYCAAMNGYSNPTFTNMTNSSSNMFAGTGLFPTSPNGGMNALPTCGTTPGIFSFSPANMISAVKQKSAFAPVVRNHNSPSPGPAGAVDQIPNVSVMPSGLTEINTYT

>Pm_COE

TMPGQQLVPAVRGWMQTSLVESSSSVSLHRAHFEKQPPNNLRKSNFFHFVLALYDRQGQPVEVERSAFAGFVENETEVAGEKTNNGIHYRLQLLYHNGIRTEQDVFVRLIDSATKQSIVYEGQDKNPEMRRVLLTHEIMCSRCCDKKSCGNRNETPSDPVVIDRYFLKFFLKCNQNCLKNAGNPRDMRRFQVVVSTTVHVDGHVLAVSDNMFVHNNSKHGRRARRVDPAEATPAIKAINPSEGWTTGGATVIIVGDNFFDGLQVIFGSMVVWSELITQHAIRVQTPPRHLPGVVEVTLSYKNKQFCGAAPGRFVYTALNEPTLDYGFQRLLKTVPRHPGDPERLPKEIILKRAADLMEAFQYKYNPHGQMGSTGAVPPPPHLAPPPTGNHGSFVPQASVAMAAAAMNGYAAAAVSSGFVPATERFDTTGSDSGYSRGNSVSPRNGYSPQGTPNSVTSGSQGVMSALTTVAAAPAPSAYNSTQPFNSYTCAAPTFTNMTNPSTALFSGSSILPSSPPNPMNPLSSSTTPGIFSFSPANMISAAKQKSAFAPVIRPHNSPSPLASSNGNLGSTIMHALNGYS*

>Hr_COE

MSNLSSQQLAPVRGWIQTSLVDALPSVGLHRAHFEKQPPSNLRKSNFFHFVLALYDRQGQPVEIERTAFIDFVENETAICYEGQDKNPEMRRVLLTHEIMCSRCCEKKSCGNRNETPSDPVIIDRYFLKFFLKCNQNCLKNAGNPRDMRRFQVVVCTTVQVDGHVLAVSDNMFVHNNSKHGRRAKRIEPAEATPSIKAMSPSEGWTTGGATVIIVGDNFFDGIQVVFGSMIVWSELITQHAIRVQTPPRHLPGVVEVTLSYKNKQFCKGTPGRFVYTALNEPTIDYGFQRLLKCIPRHPGDPERLPKEIVLKRAADVMEAFMSRPYNQVTPAPSAPLQGSYNGSTSAMAAASGYHTGVPVHNQYSISTPDRLDSVNGSDSGKNRGLSYSRGNSVSPKTGYSPQSTTHNVLNTGLTSIGAVTNVSHYGTTMNAYSCPPTFTNMTNTASNMFCNTGLLPPSPNSAMNGLPAAGPTPGIFSFSPANMISAVKQKSAFAPVIRAHNSPSSSSGTPGPGILQGTYYKENIFFTFPCVICKNLFVELDIAKIPDDFRDKIRLGICLQRVPLLYISVTI*

>Mm_COE1
MFGIQESIQRSGSSMKEEPLGSGMNAVRTWMQGAGVLDANTAAQSGVGLARAHFEKQPPSNLRKSNFFHFVLALYDRQGQPVEIERTAFVGFVEKEKEANSEKTNNGIHYRLQLLYSNGIRTEQDFYVRLIDSMTKQAIVYEGQDKNPEMCRVLLTHEIMCSRCCDKKSCGNRNETPSDPVIIDRFFLKFFLKCNQNCLKNAGNPRDMRRFQVVVSTTVNVDGHVLAVSDNMFVHNNSKHGRRARRLDPSEGTPSYLEHAATPCIKAISPSEGWTTGGATVIIIGDNFFDGLQVIFGTMLVWSELITPHAIRVQTPPRHIPGVVEVTLSYKSKQFCKGTPGRFIYTALNEPTIDYGFQRLQKVIPRHPGDPERLPKEVILKRAADLVEALYGMPHNNQEIILKRAADIAEALYSVPRNHNQLPALANTSVHAGMMGVNSFSGQLAVNVSEASQATNQGFTRNSSSVSPHGYVPSTTPQQTNYNSVTTSMNGYGSAAMSNLGGSPTFLNGSAANSPYAIVPSSPTMASSTSLPSNCSSSSGIFSFSPANMVSAVKQKSAFA
PVVRPQTSPPPTCTSTNGNSLQAISGMIVPPM


>Mm_COE2
MFGIQDTLGRGPALKDKSLGAEMDSVRSWVRNVGVVDANVAAQSGVALSRAHFEKQPPSNLRKSNFFHFVLALYDRQGQPVEIERTAFVDFVENDKEQGNEKTNNGTHYKLQLLYSNGVRTEQDLYVRLIDSVTKQPIAYEGQNKNPEMCRVLLTHEVMCSRCCEKKSCGNRNETPSDPVIIDRFFLKFFLKCNQNCLKTAGNPRDMRRFQVVLSTTVNVDGHVLAVSDNMFVHNNSKHGRRARRLDPSEATPCIKAISPSEGWTTGGAMVIIIGDNFFDGLQVVFGTMLVWSELITPHAIRVQTPPRHIPGVVEVTLSYKSKQFCKGAPGRFIYTALNEPTIDYGFQRLQKVIPRHPGDPERLAKEMLLKRAADLVEALYGTPHNNQDIILKRAADIAEALYSVPRNPSQIPALSSSPAHSGMMGINSYGSQLGVSISESTQGNNQGYIRNTSSISPRGYSSSSTPQQSNYSTSSNSMNGYSNVPMANLGVPGSPGFLNGSPTGSPYGIMSSSPTVGSSSTSSILPFSSSVFPAVKQKSAFAPVIRPQGSPSPACSSGN
GNGFRAMTGLVVPPM
>Mm_COE3
MFGIQENIPRGGTTMKEEPLGSGMNPVRSWMHTAGVVDANTAAQSGVGLARAHFEKQPPSNLRKSNFFHFVLALYDRQGQPVEIERTAFVDFVEKEKEPNNEKTNNGIHYKLQLLYSNGVRTEQDLYVRLIDSMTKQAIVYEGQDKNPEMCRVLLTHEIMCSRCCDKKSCGNRNETPSDPVIIDRFFLKFFLKCNQNCLKNAGNPRDMRRFQVVVSTTVNVDGHVLAVSDNMFVHNNSKHGRRARRLDPSEGTAPSYLENATPCIKAISPSEGWTTGGATVIIIGDNFFDGLQVVFGTMLVWSELITPHAIRVQTPPRHIPGVVEVTLSYKSKQFCKGAPGRFVYTALNEPTIDYGFQRLQKVIPRHPGDPERLPKEVLLKRAADLVEALYGMPHNNQEIILKRAADIAEALYSVPRNHNQIPTLGNTPAHTGMMGVNSFSSQLAVNVSETSQANDQVGYSRNTSSVSPRGYVPSSTPQQSNYNTVSTSMNGYGSGAMANLGVPGSPGFLNGSSANSPYGIVPSSPTMAASSVTLPSNCSSTHGIFSFSPANVISAVKQK
SAFAPVVRPQASPPPSCTSANGNGLQAMSGLVVPPM
>Mm_COE4
MFPAQDALPRGGLHLKEEPLLPSSLGSVRSWMQSAGILDSNTAAQSGVGLARAHFEKQPPSNLRKSNFFHFVLAMYDRQGQPVEVERTAFIDFVEKDREPGTEKTNNGIHYRLRLVYNNGLRTEQDLYVRLIDSMSKQAIIYEGQDKNPEMCRVLLTHEIMCSRCCDRKSCGNRNETPSDPVIIDRFFLKFFLKCNQNCLKNAGNPRDMRRFQVVVSTTVSVDGHVLAVSDNMFVHNNSKHGRRARRLDPSEAATPCIKAISPGEGWTTGGATVIIIGDNFFDGLQVVFGNVLLWSELITPHAIRVQTPPRHIPGVVEVTLSYKSKQFCKGAPGRFVYTALNEPTIDYGFQRLQKVIPRHPGDPERLPKEVLLKRAADLAEALYGVPSSNQELLLKRAADVAEALYSAPRAPAPLGPLAPSHPHPAVVGINAFSSPLAIAVGDTTPEPGYARSCGSASPRFAPSPGSQQSSYGSGLGAGLGSYGAPGVTGLGVPGSPSFLNGSTATSPFAIMPSSPPLAAASSMSLPAAAPTTSVFSFSPVNMICAVKQRSAFAPVLRPP
SSPSQACPRAHREGLPDQPFEDTDKFHSAARGLQGLAYS
>Bf_Coe
MDTLPRGTTTLKEEPLGSQIGSVRNWMQPSIVDQTATSGSVGLARAHFEKQPPSNLRKSNFFHFVIALYDRQGQPVEIERTAFIDFVEKDRISPQETAGENTRNGIHYRLQLLYSNGVRTEQDLYVRLIDSMTKQAIVYEGQDKNPEMQRVLLTHEIMCSRCCDKKSCGNRNETPSDPVIIDRFFLKAFLKCNQNCLKNAGNPRDMRRFQVVIATTVNVDGHVLAVSDNMFVHNNSKHGRRARRLDPSEDGEMSFCGDSPNINRGQKATPCIKAISPSEGWTTGGATVIIIGDNFFDGLQVVFGTMLVWSELITPHAIRVQTPPRHIPGVVEVTLSYKSKQFCKGAPGRFVYTSLNEPTIDYGFQRLAKLVPRHPGDPERLPKEIILKRAADLAEAIYSMPRNPNLPALTGPRSPAVNNSVGAGGMMGMNSFGNQLAVSVPDNSSTNGNGQVQGFVPVFTYSGLKEDCAEQTNHGYSRQSNSVSPRGYGASTPHSTNGSISSYSTSASGLNGYGSSGNLSNMPVPSSPGFLNGSTIPSSPTMPPTPSSLATNASTPGIFF
FSPANMISAVKQKSAFAPVVRPQSSPSPAGASSNGTGLQGGWTPTIPSAVC
>Nv_COE
MSFDMQEQDENSMKNDRPVVGCLQHRVLEPQVTSTASGLLDRCHFEKQPPANLRKSNFFHFVLAFFDHNGQAVEIERASFIDFIEHLPEDKNVRNGALYRVLLLYSNGIRTEQDIFVRLIDAATKQIVPYEGQDKNPEMRRILLTHEVMCSRCCEKKSCGNKNETPSDPVVVDRYCLKFFMKCNQNCLKNAGNPKDMRRFQVHVSTSVDPMYDMIACSDNMFVHNNSKHGRRTRSRADGNEQDTSADDPCIKAICPNEGWTIGGSNVILIGDNFFDGLQVVFGSFIVWSEFITPHALRVQAPPSPMPGVVKVYLMHKEKQYCKYAPAKFGYTALVEPTIDYGFQRLSKLIPRHPGDPERIPKEIVLKRAADLAETLYQMPRTPTQVHPSQYASTGLATPKSPALVGGQHYVTLLPDSMTNVFTQSITPDNQPIAYSSHNDNASDQTDMNNNVVQSSNSSRLTSNATLSGVTNMSNHDSHHNGVMASSDEGLSAIATVSNSCYGNGSPALSCMAVPASPGYFTGAFIPQSPSLPPTPNSVPPSSNNSIFSFPPNMIQAVKQ
KSAFNPVSRGPESTAHPRSPAVVQGVMTSTYNGPFAVPV

>Af_COE
MRQILKEEPVPRAWPQPALADNGTVGVGRAHFEKQPPSNLRKSNFFHFVIALYDRHGQPIEIERTSFMGVEKDQESEGQKTNNGIQYSLHLLYSNGSYECNRDIFYRCIKNVSRQAIMYEGQDKNPEMCRVLLTHEVMCSRCCDKKSCGNRNETPSDPVIIDRFFLKFFLKCNQNCLKNAGNPRDMRRFQVVISTQVGVEGPLLAVSDNMFVHNNSKHGRRTKRLDPSDPGEYNSLYTPVPLQTPCIKAISPNEGWTSGGSTVIIIGENFFDGLQVVFGSMLVWSELITPNAIRVQTPPRQIPGVVEVTLSYKTKQFCKGAPGRFVYVSLNEPTIDYGFQRLQKLIPRHPGDPEKLPKEIILKRAADLAEALYSMPRSGNAGITGAPRSPGSGHPPAPPTSSSATAFNSYTGQLAVTVQENGSAAKWTDDGSSVTTGAGAGGGGGGGGSVGGSVGGSADAYRQSSSASPRGVVTGGGYCGSSASTPHSHSTNGSYSVANPYTGSPTLYTSPHEQYGIFYTSGDGSAFAPVVRPPTTSVPPHWSTQHHLATAAQ

#*******************************************************************************

#MESP

> Bs_MESP

MMRKLRVYDGRPMPYVRNGPILQRRQFDNYYRVFPSNREIASLREKNRVQHLTAQLLTLKSYIPESWFPEGCERRNASKIVILRAAISYIRYLTDLLNKAGVSSGIAATGKENARDPELQEVTEYIDEVYEKQCKPRCESRPPAVAAFR*

>Ci_MESP
MELSMHQDPIDDWLMELLRENECGTDSNDFQEQQLVKKTDVFSTTAQQHAQLESSENIYPSIASQVRPFTRNNANIYHSQEQCPKFPNTHRHAFGKQRNERNPLTNVCLNKQHTNQKPDSCVPKNRYYRTLKLKSKRQTASERERLRMQKITEQLMTLKHHLPDHYFRNDNPSKVQILRKSISYIHTLSKLLQEGKT

>Cs_MESP
MEHILEPIEDSIDQRLDELLQENICEGPTFNTGNAPRRKEMQIEYRCQAQQPSNKNDQPRQRFYNELNQTWLLSNNSLPVLEQHSVTVCKRKTEEINRRPLGAVCVNTKPMSQVKHVQRTAKRISGNEYCNRRSKRQIASERERLRMQQISRELMNLKRHLPEYLFCDKNPSKIQILRNSITYIKNLSSFLYVDSKTQNLSL

>Bf_MESP
MAYATTDFFENDDGLSSLLRADTPVTSADRDGSDSSPSFEDSGLSSGGSPESSALLLNGGSRRRRRRRKPRLTGLSKQRQAANERERVRMQNLTAALGVLREHIPPPVAPKDKRLSKIETLKLAIGYIDYLRRVLQESTENAASLLPPSLESLAKEDAEFDIDGSFEIKGQDKEQRRRRKDRTARHRLTTPHFNAKKSQQTKPAPRPTAAKVRPRPSTVSSCSPKSGGSNDGMSVKLET

>Moc_ME

MDQSVLGAFSSISGQQETGFGNMPSKTTFINYPNIGNIFIETESNFLERNNQSFDTIKHSTMKSKRPQNKNKKMTNRQFGDDITNFGPFQRQLPRVETCNHVPLTNENFSPTYNYKSLRNDNDETMFPNSKRARTEIHDPSAIRSSRSNKAMFSRRKIASEREKLRMQKISNQFQVLKSVLPPTRFAGNGRVTKIEILHQAMD

>Phm_MESP

MQVSNEIKMSQFTQQRHITAQPGTLAELPLNLLSYLNQQYNTETKLSKKATQCPDNNFQNQRFQYFAEKPKQHVATGCVQQLQYDEPTRENRQCKYSTNQRSVRYRSILTNEELENRSAKRRAASEREKIRMQKVTGQLLKLKQHLPDSLFENRNPSKIAILRQAIYYIDTLSKTLRDSDDEDVQMFFSQ

>Hr_MESP

MQRSFPEDNSYQIYQYRCQSEWNRQVHPSLLTPQCDVENNPFSFFNDTSLYSTGQQENRAGTQIMQEIQYKNERTSNTGPLRTRKSNFTKAKPYAIGLASRNGRSERRKTASDREKLRMQGVTRQLNVLKDHLPSSWLPDKAKTSKINILRIAISYINYLSEQLSADNADNVHHDAHENTCKIDFELQDIIDYIDDSYCGNLEQGEQRTKLSLFR*

>Dr_Me-a
MDASTFSLQLQNCSFFLPDSQNQSFAVSDAGYYSATGSLSPTSSIDSCSFSPPAYSLLPQIFPKSIQKTDVQPPKRTGRPKSKFPGVKRQTASEREKLRMRDLTKALHHLRTFLPASVAPVGKTLTKIETLRLAIQYISCLSDQLGCGEDVEICEAQDEVISTSASVFDNFSSASSASQSLPAQQFMSMSCYQTQNPVQGDFHSFPAQDVWFSQRHNFFHGQC

>Dr_Me-b
MQTSSKNRWSSSSSESEFSSISSPETTSPDQSFSPPHQTKPPCSKLVKSSNIMRKKRRLRLKNPSERRQNASEKEKLRMRDLTKALHHLRSFLPASVAPVGQTLTKIETLRLTIQYISFLSSQLGLSEEELSYRRQENSSGCSLSSFECSSVNGGFVGTEQGYALCDGQYEDCSGYGGQYRERYGGLTQQHSTEQNGLVSIDGFIQSQQCGQMTQTPYQVYGKNFGYHLVPQTYWR

>MmMesP1
MAQPLCEPRSESWILSPAGRQPPMPSDGNSVCSPAWSSDPWDGAQASSPAPPCARPARRAGTPGRRGTHGSRLGSGQRQSASEREKLRMRTLARALHELRRFLPPSVAPTGQNLTKIETLRLAIRYIGHLSAVLGLSEDNLRRQRHAVSPRGCPLCPDSDLAQSQSLGPGLSPAVCSGVSWGSPPAYPRPRVAAESWDPSFQYAETASQERQEMEPSPSSPLFSSDMLALLETWTPPQEWPPA

>MmMesP2
MAQSSPPQSLQGLVPLGLLPGLGLGSAIGLHVSGLVLRFVRFLPFYATRRPSQPAGPARSTRTTQATAPRRTRPAPAGGQRQSASEREKLRMRTLARALQELRRFLPPSVAPAGQSLTKIETLRLAIRYIGHLSALLGLSEDSLRRRRRRSADAAFSHRCPQCPDGGSPSQAQMLGPSLGSAMSSGVSWGCPPACPGPLISPENLGNRISNVDPRVTPPYCPQIQSPLHQSLERAADSSPWAPPQACPGMQMSPEPRNKTGHWTQSTEPAELTKVYQSLSVSPEPRLSLGSPLLLPRPSCQRLQPQPQPQPQWGCWGHDAEVLSTSEDQGSSPALQLPVASPTPSSGLQLSGCPELWQEDLEGPPLNIFY

>DrMeso
MAQIDVDVFTAKVLSHWDWSREDRSFGDSASSPESESFDSACSSPDARSSPTAGCEHAEQQKPKVKMSMRRRMKASEREKLRMRSLAEALHQLRDYLPPGYSRRGQPLTKIQTLKYTIQYIKELSGILEQQ

>Mm_pMg1
MDNLGETFLSLEDGLDSSDTAGLLASWDWKSRARPLELVQESPTQSLSPAPSLESYSEVALPCGHSGASTGGSDGYGSHEAAGLVELDYSMLAFQPPYLHTAGGLKGQKGSKVKMSVQRRRKASEREKLRMRTLADALHTLRNYLPPVYSQRGQPLTKIQTLKYTIKYIGELTDLLNSSGREPRPQSV

>Dm_sg
MTDQLLSSTAGVPGDPPPVLVPVSATTGGPYLSGGSEGEAESSEQPVLLELGAPQSSSCPAVSLNYTLTADGAGLLAYAPTHPHSYSPSVMGGYEKEAHNMGLLPPTYSVIPQPVSMWHAGQVATGSPVGLECNKPSELVMYMSYSGGSIANSTEVDIAKEHNPAWREKALQMEKDYRRTACDRERTRMRDMNRAFDLLRSKLPISKNGKKYSKIESLRIAINYINHLQAMLRESSVGQNGNGCCAWSGGSSSPYDNGNDHWGA

>Ci_E12
MFSSKSSSISGIENSNHGNVYSSKSYAESTGWSAPTDPPRESYSSPTNYPNTVTNTAARSPNVVVGQPGGSGKPPMTPGPYGYPREQSVMLNLHRPNSSSPNSSNNNNNPYPNKRRQPEVHAPGKRRRGPYSPTQSEDYHSYQAPITPGDPMHGSGDWSSPGYTGTMGQYGQCHPAERMQDYRPDSSPHMGSCQQTGMHGSYPNLTSPYGINEIYQNNSGFQASPAATPSPMLGNNQWRQHPVESFPNQHNTPSQSLDDVVAVLGDHAGIHNEMHRQQVGYPPHTPITWGSQNGSDVAGYSRMAHSNTPLISPTNSTNMSGNTANYPLSQPPASHQTRQIFSPTGTSNVQPASINSDNTSNSGASSIDDNILNIKKEKDSADLKVKTNSRAAKTKARSASSKSMEDKVDDDDESKEERDLRRSSIGSKDNDEDLTAEEREKREKDRRMANNARERLRVRDINEAFKELGHMVQIHMNQDKPQTKLTILHHAVQVILGLEHEVRERNLNPKAACLKRRDEEKAATSSMQQQPAVDEGRLSNTIHAQPSKRSRSGSGVTAPVAPGYPGASTHPNYHQNPHNQGDPNSIPQHPYGIKHYGPHPGEMMTSESMSSFNEHGYVSPDVMGMDPGVQLDTPFSTGHP

>Hs_ITF-1
MNQPQRMAPVGTDKELSDLLDFSMMFPLPVTNGKGRPASLAGAQFGGSGLEDRPSSGSWGSGDQSSSSFDSRTFSEGTHFTESHSSLSSSTFLGPGLGGKSGERGAYASFGRDAGVGGLTQAGFLSGELALNSPGPLSPSGMKGTSQYYPSYSGSSRRRAADGSLDTQPKKVRKVPPGLPSSVYPPSSGEDYGRDATAYPSAKTPSSTYAPFYVADGSLHPSAELWSPPGQAGFGPMLGGGSSPLPLPPGSGPVGSSGSSSTFGGLHQHERMGYQLHGAEVNGGLPSASSFSSAPGATYGGVSSHTPPVSGADSLLGSRGTTAGSSGDALGKALASIYSPDHSSNNFSSSPSTPVGSPQGLAGTSQWPRAGAPGALSPSYDGGLHGLQSKIEDHLDEAIHVLRSHAVGTAGDMHTLLPGHGALASGFTGPMSLGGRHAGLVGGSHPEDGLAGSTSLMHNHAALPSQPGTLPDLSRPPDSYSGLGRAGATAAASEIKREEKEDEENTSAADHSEEEKKELKAPRARTSPDEDEDDLLPPEQKAEREKERRVANNARERLRVRDINEAFKELGRMCQLHLNSEKPQTKLLILHQAVSVILNLEQQVRERNLNPKAACLKRREEEKVSGVVGDPQMVLSAPHPGLSEAHNPAGHM

#*******************************************************************************

#MRF

>BsMRF
MPSLSSTDGSATRVMNSSSMDQQQDIIVSKDVFVNSPVDSDSDDSFRQSPNESRRFGDTFIHPDSGIDHDFNLDDFAVNSTPLDTLCRPGDPDFEGFDLSSDQQDYVFSPSTILDLKLTDGIEDWKPGDVFENITTEVLDNNEESKSDIKAEYDDSEFLDLTDVDTLPFDLGNTARDTSLSQNNIFVNAPVSVRSKRKGVIQDAGTVLNLSLLDRGFDQENCLQTRAVNNYIPFVANGIHGNIGHLGYSQGNVLAQNQQLNFLSNIPQQQVQVEGLSFNTNDLNSLAELLMTPKEQTSVKPSTRLAPCSTLLPGSKIMTTSPSKESCVQSNCDMQHARDEFMDHEESSSNSDGGEPNFFNHSLNYEIINNAGVNFQRSDVIDSNDHSTSSHHFSTTIIPDSSGIGATVSSTTDILSQSCEAIQGEFSVGYGSHACHPNGQQCLVWACKACKRKSGPHDRRRAATLRERRRLKRVNEAYETLKRCSCSNPNQRLPKVEILRNAINYIENLQRMLYESNESAEKWPENNSQSSIQQQGVTDDISSPKQLCSETTITEHHALSEIKLENDFDSISAASSSGEQVLNLSTSTSFARTSNYNTDLQSMNLSSTST

>Mo_MRF

MYFEIPNFVMASQLDSVVNEKGNDVMLSYAPGSPTNESNSGSEFEPTADAKNELGLIELPAITNENQLIQTAFASSDVVDNSKCDIFDPTMLINSSGQDVKLEYDPSEFLDLNDVDAFEFSLENSIADASRQNDNVFQTNDKETHLQPISNMLPYLQTTETGQSKSPDINTIADLLNCNPERRQQPNTRLAPCASLLPDPKVSLGPNNFATSSIPQLSSSDVTQCIPTMNYDQMTPTFVT

DHSSNLLMTSNMASYTMNATPVQDMEVTDSNHTSHPNGHQCLVWACKACKRKTGPHDRRRAATLRERRRLKRVNEAYETLKRCACTNPNQRLPKVEILRNAISYIDNLQQLLYGDKVTTTPSKDDTKLDESSLASNVKTPPRSGLSCSPGHVGLLDKSLSPESIFQMNGQVVTSVSQGGGRL

>Ci_MRF
MTCISLEELDLSSIFSNSSSYFTSYATNPIMTSQKRMPSRLKRASSDVLLSDTAVSPESREVSGVLSELDELKRCVEGNYIGIDAEKSDISILEELSNMASGCSDSDVAYSSPDSRYGSTGNLTSKTSFGSAMSFRDAGIGGPIPRGSSLRNPDIRGKRNSIEVKQENEQNPIEFVLESFLNSNETSKPRMPESRVEDIQFTGPPISSIEPTITFASTEENHNDTVKAMMQYLTETNQMCQESSVSEQIIFSDLNSVSASDSLPSVEELLQIPNEKSRSFKPDNTAKHQPTLHRPKHFHNQVANQQHVPILNPELQSFESFSPVMNNGLITPTNTFTSNRMMELSPLSDLHSLSQDEDMDTKMSHYHHTSHPNGHQCLVWACKACKRKTGPHDRRRAATLRERRRLKRVNQAYDALKRCACANPNQRLPKVEILRNAITYIYNLQHMLYGDQQSDAKSPETKPETTLSLGEIFVSKTEIDSPFYQSDDVRLTSSRTSSPVESLLESTSSSFIMSDLGDENIQPQVL

>Cs_MRF

MTCISLEELDLSSIFSNSNNYYSGYATSNIMTTQKRTPPRLKRASSDVLLSDTAVSPNCRGVSGVLSELEELKRCVEGNYIGIDAGKSDISILEELSHLATNCTDSDAAYSPGSRDSTLSNRASFGSEMNFQNPGIGGPMSRGNSPQNYDLRCKRMDVKQENEANTIEFMLESFLGSNETSKQKQDHRTEDTQSTPISSIEPTITFATNDETHTVKAMMQYLSDTNQMCQDNSVSEQIIF

SDLNSVSASETSLPSVEELLQIPNEKPSSFKTGNTAKTQSMHSRPKHFNGQMLNQTQIPINNVMSPEVQSYESYTPVLSSGMLSSNTFSSNRMMELSPLSDLQSLSQDDEMDSKMSHYHHTSHPNGHQCLVWACKACKRKTGPHDRRRAATLRERRRLKRVNQAYDSLKRCACANPNQRLPKVEILRNAITYIYNLQRMLYGEQNSEAKTSVSKPEATLTLGETFISKTECESPFFETDD

VRMTSSRTPSPVSSLIESGPSFVISELGEENIEPKQDRLSASLFPNDVTTPTSMVDDVITITEESTTTPNTDEENTPVANRLNSDTKGASSLVCLSSIVERID

>Hr_AMD1
MHANNSVLPFNPRPELQILGQDFNGHLNLLSSPEQLLESTPNQMDLTTDANVILNPHDLSSLAEFLAVSPDRNAIIRNSLTPGRLGPCSTLLPGSKIATLKSMDKPYIPVHDLTITTTNEMVHTVTPGQSIISNAYSNSVDHLDISNAIDLTNQNAYPFQIGQSNSNIITNTTNTGFDITNAKIFSISDTLNDSGFASSNTDASDATYSDHTHQGSHTSHPNGHQCLVWACKACKRKTGPHDRRRAATLRERRRLKRVNEAYENLKRCACSNPNQRLPKVEILRNAITYIENLQRILYEASENGENEYRSKNATHASAIKFMTSSFEDDDVKPFAKRIRLDNTSSVVKRGSHVKPVRNESLTSGMMSQNTSLITQSMTSASIATLITDSDVSSLAMNKETTCDNSMMIDLKAELGQLIKTSGNHDAVLITDISGR

>Hr_anis

MPSIMMSSKMADNIMTSSPEGQSHIISNDVFITSPMHSVEGERSTPDIRDLSDNFINPPEHIGGIGNGGFNLNEITTSSAIENLCFITSNSTSSYQNATLNCNSSEQLIFSPSGIFETNNTTGDVKLSNSELTLDDDVTNTNDVGEWKTCDIFDTSVNLSTKSQQINSQNEIKLNYDPTDYLDLNDVDAFEFEFENSIADASLRNNNLTFNTSQIQTQSTNQNEKHHILNLSILEPTFSD

DGDKDGYMHANNSVLPFNPRPELQILGQDFNGHLNLLSSPEQLLESTPNQMDLTTDANVILNPHDLSSLAEFLAVSPDRNAIIRNSLTPGRLAPCSTLLPGSKIATLKSMDKPYIPVHDLTITTTNEMVHTVTPGQSIISNAYSNSVDHLDISNAIDLTNQNAYPFQIGQSNSNIITNTTNTGFDITNAKIFSISDTLNDSGFASSNTDASDATYSDHTHQGSHTSHPNGHQCLVWACKA

CKRKTGPHDRRRAATLRERRRLKRVNEAYENLKRCACSNPNQRLPKVEILRNAITYIENLQRILYEASENGENEYRSKNATHASAIKFMTSSFEDDDVKPFAKRIRLDNTSSVVKRGSHVKPVRNESLTSGMMSQNTSLITQSMTSASIATLITDSDVSSLAMNKETTCDNSMMIDLKAELGQLIKTSGNHDAVLITDISGRYVKPQ*

>Pm_Anis

MFSNGSVCLQSLMATPEMTSQLRFVTQSPRRKRSSHDVLLSESISEREVSESRDAKTKEEEQKRMDEIAMKSDLSILEELTNFTSSTTHSDVMFTSPEPVFPTQTDQYVVSPQLGPVQGLKTQGQVTLEKPIKKEQIDNSANFELALENLLESDYVIASEPKLHYPVVTSFDEEAAAPPISSIEPTLTFGNCEENEDAIKAMMQYFTEGTPAKRPHLSIDTALPSVEELLSLSSSSEKSRDFKPSHKVPNPVTSAVFPSNKIDMRTSAFTNSEQLIFKSDAMTNFSEDALDFDAHSNTERVHFAKIPGLQISNSLSGSDGMFDQGDEENSLSSFHHTSHPNGHQCLAWACKACKRKSGPHDRRRAATLRERRRLKRVNQAYETLKRCACANPNQRLPKVEILRNAITYICNLQRILYGDHQQPEEQADKTGNSLASTTHAEALSAFSSPASMSPNFCNGNDVIRLAMTSPSTPVSPVNTEGCTDALNEQLSMLQRGDDVSLTSPPGVMCFSEDSVDHTSPTTSVNSDTKRNSSLVCLSSIVESITDEL*

>Pm_AMD1
MTCLPFEELDFASYMFSNGSVCLQSLMATPEMTSQLRFVTQSPRRKRSSHDVLLSESISEREVSESRDAKTKEEEQKRMDEIAMKSDLSILEELTNFTSSTTHSDVMFTSPEPVFPTQTDQYVVSPQLGPVEGLKTQGQVTLEKPIKKEQIDNSANFELALENLLESDYVIASEPKLHYPVVTSFDEEAAAPPISSIEPTLTFGNCEENEDAIKAMMQYFTEGTPAKRPHLSIDTALPSVEELLSLSSSSEKSRDFKPSHKVPNPVTSAVFPSNKIDMRTSAFTNSEQLIFKSDAMTNFSEDALDFDAHSNTERVHFAKIPGLQISNSLSGSDGMFDQGDEENSLSSFHHTSHPNGHQCLAWACKACKRKSGPHDRRRAATLRERRRLKRVNQAYETLKRCACANPNQRLPKVEILRNAITYICNLQRILYGDHQQPEEQADKTGNSLASTTHAEALSAFSSPASMSPNFCNGNDVIRLAMTSPSTPVSPVNTEGCTDALNEQLSMLQRGDDVSLTSPPGVMCFSEDSVDHTSPTTSVNSDTKRNSSLVCLSSIVESITDEL

>Af_MRF
MTLVTNTISYACQSIGRESYDVYRHHHLQLRHHNHHLHRYYYHRHHHQYYHHYHYRRRQSVYTSIGNPSIEYLQRHLTEENVDEETKNRVIETTENDFALGSTNSQRYQSQSLRYAVDSDDASSIVSVDASADLESGSVEAEESNEGNELNESNERSNELNETELHEHVPHPEVALDATTSIHGPRRCLLWACKACKKKTVTVDRRKAATLRERRRLRKVNEAFEILKRRTSNNPNQRLPKVEILRNAIEYIENLEALLQNNRSSLAEDHGSVENTTDTTSPQYMMEKLRQFSDPLARFQPINEFEEMLESQSSQLNGSSLDRLNMIVQSINGPICATTDTLRYPSHQ

>Sp_SUM1
SVYANTFANDAHPHQARNGGSQCYGSDSANSSPGESCREEDLEHVLAPGFPGQGERRCLMWACKACKRKNVAVDKRKAATLRERRRLRKVNEAFEALKRHTCANPNQRLPKVEILRNAIEYIEKLERLLHVEKANGDSEMDSAETSSNTSDAMTDGSSPGSYSSDKAQQYGDGYDVSSPYGYNGCGNASSLDCLSLIVESITPTKGKAITSPKKATSDGLCMV

>Mm_myog
MELYETSPYFYQEPHFYDGENYLPVHLQGFEPPGYERTELSLSPEARGPLEEKGLGTPEHCPGQCLPWACKVCKRKSVSVDRRRAATLREKRRLKKVNEAFEALKRSTLLNPNQRLPKVEILRSAIQYIERLQALLSSLNQEERDLRYRGGGGPQPMVPSECNSHSASCSPEWGNALEFGPNPGDHLLAADPTDAHNLHSLTSIVDSITVEDMSVAFPDETMPN

>Mm_MRF4
MMMDLFETGSYFFYLDGENVTLQPLEVAEGSPLYPGSDGTLSPCQDQMPQEAGSDSSGEEHVLAPPGLQPPHCPGQCLIWACKTCKRKSAPTDRRKAATLRERRRLKKINEAFEALKRRTVANPNQRLPKVEILRSAISYIERLQDLLHRLDQQEKMQELGVDPYSYKPKQEILEGADFLRTCSPQWPSVSDHSRGLVITAKEGGANVDASASSSLQRLSSIVDSISSEERKLPSVEEVVEK

>Mm_Myf5
MDMTDGCQFSPSEYFYEGSCIPSPEDEFGDQFEPRVAAFGAHKAELQGSDDEEHVRAPTGHHQAGHCLMWACKACKRKSTTMDRRKAATMRERRRLKKVNQAFETLKRCTTTNPNQRLPKVEILRNAIRYIESLQELLREQVENYYSLPGQSCSEPTSPTSNCSDGMPECNSPVWSRKNSSFDSIYCPDVSNACAADKSSVSSLDCLSSIVDRITSTEPSELALQDTASLSPATSANSQPATPGPSSSRLIYHVL

>Mm_MyoD1
MELLSPPLRDIDLTGPDGSLCSFETADDFYDDPCFDSPDLRFFEDLDPRLVHMGALLKPEEHAHFPTAVHPGPGAREDEHVRAPSGHHQAGRCLLWACKACKRKTTNADRRKAATMRERRRLSKVNEAFETLKRCTSSNPNQRLPKVEILRNAIRYIEGLQALLRDQDAAPPGAAAFYAPGPLPPGRGSEHYSGDSDASSPRSNCSDGMMDYSGPPSGPRRQNGYDTAYYSEAARESRPGKSAAVSSLDCLSSIVERISTDSPAAPALLLADAPPESPPGPPEGASLSDTEQGTQTPSPDAAPQCPAGSNPNAIYQVL

>Bf_MRF1
MEFVELSSCRFDATPTFCDRPAAPNATVLPGEHFPVPNGSYESDHGDGHVLAPGPSFHGPGRCLLWACKACKKKTVPIDRRKAATMRERRRLVKVNEAFDILKKKSCANPNQRLPKVEILRNAISYIEQLHKLLRDSKENSSGEVSDSSAPSPGSCSDGMAAHSPHSFCTDTSGNSSWEHGDRHHGNGHENQSCGNNVSSLDCLSLIVQSISTIEGEENNNASNPPR

>Bf_MRF2
MMNYTELSSCRFQFDTSYPQSPDQGLDMFGSLQVPAPMEAYGGYGESAGHVPTPAAAAHGPGRCLMWACKTCRSRKASRHDRRKAATMRERRRLVKVNEAFEVLKKKTHMKPNQKTPKVDILRNAIAYIEQLHQTLRDGQENSAGQSADSPAASPGACSDGMSANSPDSVWSDTSGTFQGEDNMASFGCDDVTASWSSASCLDSLSLVVQNISAFAAQDSHQGYTPLRS

#*******************************************************************************

# Nkx

>Bs_Nk4
FLRLNISVVRSDERSTAFKLETGGYQYHYFYCIENIHICVWCVSQISLRKYYSTHSCERVKRFELCICSNMLSSPNNSTPFSVKDILNLESQRQQVEAGFLVNDHHPSSRRSISHETLPQGTIESSYEYPGGERGFRYGNGAPGSPLTSPDCNMASSGFSKSVGDEPSYAYMGPMPPHEYGSAPIPPTSNIRHHSPMSNSQYYGPDKNANIYRQSEPRSIEESGRKHGDGSERASSAHSD
GFSYTTITSNPGSVPMAPTGLNGSGFMSSPNSTTGGYLNEYGYSASYSSPSGQLQDPSYPYSHHQATYPPTDNATTLPRHHSPNSHQYASLPYSQPHQQTCVPAVQNGSSPQPTHFYPRGGPASEAGSEELIDPSGPGDMADTANNFPSSQPFPRSGVDRDNADSNGLYHVNESMHARTDVSQFNASKSPESQNKLTESDATPDDVSIHMEESEKLDNVSNSSGSPEDDDKKKPEEDPSK
SRQRTRRKPRVLFSQAQVFELERRFKQQRYLSAPEREHLAQLLTLTSTQVKIWFQNRRYKCKRQRQDKTLELASACPPRKVHVPVLIRDGQPCLPSPTGSIPGGHPSLQASYSAPYNVTVSPYHSYPSSYTSCGYNTYHPNSNTYSHNAAAAAAAAAYGSAATAGYNPLSAPPMGSGMSAVGGIAGSTLHHQSAVRPTTFATMQGQMQQQPIRNHSTTDYMYKFGLCT*

>Bs_Ttf1
MSLSPKLQQSSTTTRFSVIDILNPLEQQSYGGDDSGQSTSVESSADSCRENNAIYNQNQILSLENSHPHSLSGVHHYSNHTDHTESGTVSIATMDPGSLAAVSAGLHVPSMGEQGVIGSMSPPGNPLQLYRHSLGSHHHQPQQLQVTTPYPCMNSTSAMTGMNGSYNLHSMPPTQSSFHSMNATGAPGTGYCNGAMTDLSHHSYVPSATANGWYSPPTNPDPRFGGVQMQRYLSPSPGMGMGSIGMNMGIDGMHKPILPSSQRRKRRVLFSQAQVFELERRFKQQKYLSAPEREHLAQMIRLTPTQVKIWFQNHRYKNKRALKEKGSDSDPSPGSSQQSSESQPITQSSQHQQQANNIQQSYSPTVSGEVINGPTPESTTGHEATESRAEHHQDRDDQSPRRVAIPVLIKDGKPCNGADIGAGNDGMNTDQMDSASDNVDDCRVQITPYANSQVKTEMPANNEQNICIGGSLEPMTQNHYVNTSSHQDLMSVNVASLNGDAVTQMTYHTLQGQHHPLGTTNAVVNNSLIYGIYR

>Bs_Nk2.2
MTLISKDVDAVECSLHSHRPNASGFSVKDILDLPVSNTRTDFCLGNGHYNRAERLQNVGVPHYPHGSERGETKYSISQHHPGDLYQPHYKGEGIRYYPTRSENLSYPSKNPYSESSCGEAPYYSGCDTEIGLLRRPSESGSPHCEQQRKFIDRFEQDRSPTSPQTPKYTEIGYLENDASGMRLNSVGSPVASGSPCSTTNHDHSPFHRVSYQTSCSDPRIASDVSFPEKEESIQTLDTNSTSRQETRNPDSVLTEEYPNPETRWEQQDTMTKFIADGNDVNEGEAPDSTSCNAEKSPSSDENEDEEEMDMDVGGKKRKRRVLFSKAQTYELERRFRHQKYLSAPEREALANAIRLTPTQVKIWFQNHRYKMKRAKQDKGSISGFDHMGPLQSPRRVVVPVLINNGKPCHSAGHYPGLSVGQDASSNPSVYGPSFHHSPHYEDSMRGYSHMPLGFDMRRPLTGSSVHSQYGNSVQQSSYSYEPTYHLSAYSQRYPSSHPASSPQYAASSYTPPYTPGSANLGPQGPTHYGLEQQFWQ

>Am_vnd
MADKNKILPWSMLSYQTSLLNHVWYSQLAFSNRILESFKMPRSTSFHIHDILQLDSKPSQEETEVQGSTTVLPTTNDVPSTYQQFFEHTTAMLQPAIYANLNRGTLPPPPPPLLAWPTGPTLPTTLPQPLEEVNVLQQPDSTSPTISDLSFPPKQEANHECKEEESAEFEDEQQANEIQPHETEHKKRKRRVLFSKAQTFELERRFRQQKYLSAPEREHLASIIRLTPTQVKIWFQNHRYKTKRAATEASGSGCSPRRVAVPLLIKDGKPCQSKLMEPATYPATGQVPMPPYIQKPYWW

>Am_Scro
MSGGSGGNSAGNQGNGNATSYHQHQQQQQQQQQQQQQQQQQQQQQQQQTQLEQASLLTDLNGIDLAMSLSPKQHSSHVQAAGGAHTTPFSVTDILSPIDNESFRKLEQVIGVGVVTHSQVSPYGNACGARVGGNTGSSSASSGSPPLHGGSTPGGGTPGPVSGPNDAAGGGSAAAGTGSGMTAMGNPYATMQLTSQYQYCAAELSYHHAGPAVAGGATATDPMRSHHPWYAPAPPATNDPRFAISRLMGAAAAGAGTNMAGCSVGQSMGDVTKSSGMGVGVGVGVGVGMGVGAMQFPHLPQRRKRRVLFTQQQVHELERRFKQQKYLSAPEREHLAALIHLTPTQVKIWFQNHRYKCKRQAKEKAMAEQNAQNQSASSPRRVAVPVLVKDGKPCGSGGGNEGSRGGPSAALASPAPHMTAASSPHGSHHAASSVPHHSVVGVNHVITSSQSLQHCSYSRTGGQQSMQQQQQPQCGAYLPFQGRAW

>Am_tin
MLSSSGTPFSVRDILSEDQQLGIMDCYPSVHHHQHQLQQQHVPQEYYGYNTIVQENNWENVEKFKEQGSMPAYPHYPDLGHVHQLNSIAPPYQDPPVTEDGNVVTSSKTELRKSQSGKRTKRKPRVLFSQNQVYELEQRFKQQRYLSAPERELLAQTLKLTSTQVKIWFQNRRYKNKRARLEGVESLQAQNAMKGQSSLKKIHVPVLIKDGKPNLQDNPYNPSSYWSNIRPDLGVAMQPDYGCRTMNEIRLSPEFRNGEMRMDTPPQFKAEITTPELGGKSLNGGEVQAGRQHVLADFRNNLDPRLSHDCHRQIAKGNQVGGDEASHVAATTFPEVKNIGADSNKVMGSDGRPVMDVTSSDYGFPNYLAPANYQMQYVNYMEQVPMDQNLQRLW

>Mm_nkx2.2
MSLTNTKTGFSVKDILDLPDTNDEDGSVAEGPEEESEGPEPAKRAGPLGQGALDAVQSLPLKSPFYDSSDNPYTRWLASTEGLQYSLHGLAASAPPQDSSSKSPEPSADESPDNDKETQGGGGDAGKKRKRRVLFSKAQTYELERRFRQQRYLSAPEREHLASLIRLTPTQVKIWFQNHRYKMKRARAEKGMEVTPLPSPRRVAVPVLVRDGKPCHALKAQDLAAATFQAGIPFSAYSAQSLQHMQYNAQYSSASTPQYPTAHPLVQAQQWTW

>Mm_Nkx2.1
MSMSPKHTTPFSVSDILSPLEESYKKVGMEGGGLGAPLAAYRQGQAAPPAAAMQQHAVGHHGAVTAAYHMTAAGVPQLSHSAVGGYCNGNLGNMSELPPYQDTMRNSASGPGWYGANPDPRFPAISRFMGPASGMNMSGMGGLGSLGDVSKNMAPLPSAPRRKRRVLFSQAQVYELERRFKQQKYLSAPEREHLASMIHLTPTQVKIWFQNHRYKMKRQAKDKAAQQQLQQDSGGGGGGGGGAGCPQQQQAQQQSPRRVAVPVLVKDGKPCQAGAPAPGAASLQSHAQQQAQQQAQAAQAAAAAISVGSGGAGLGAHPGHQPGSAGQSPDLAHHAASPAGLQGQVSSLSHLNSSGSDYGAMSCSTLLYGRTW

>Mm_Nkx2.4
MSLSPKHTTPFSVSDILSPIEETYKKFGGVMDGAPPGLGAPLGAAAYRAPPSGPSSQAAAVAAGMQPPHAMAGHNAAAAAAAAAAAAAAAATYHMPPGVSQFPHSAMGSYCNGGLGNMGELPAYTDGMRGGAAAAATGWYGANTDPRYSSISRFMGPSAGVNVAGMGSLTGIADAAKSLAPLHAAAPRRKRRVLFSQAQVYELERRFKQQKYLSAPEREHLASMIHLTPTQVKIWFQNHRYKMKRQAKDKAAQQLQQEGGLGPPPPPPPPSPRRVAVPVLVKDGKPCQNGAGTPTPGQGGQQPQAPTPAPELEELSPSPPALHGPGGGLAALDAATGDYGGGVLGANLLYGRTW

>Mm_Nkx2.5
MFPSPALTPTPFSVKDILNLEQQQRSLASGDLSARLEATLAPASCMLAAFKPEAYSGPEAAASGLAELRAEMGPAPSPPKCSPAFPAAPTFYPGAYGDPDPAKDPRADKKELCALQKAVELDKAETDGAERPRARRRRKPRVLFSQAQVYELERRFKQQRYLSAPERDQLASVLKLTSTQVKIWFQNRRYKCKRQRQDQTLELLGPPPPPARRIAVPVLVRDGKPCLGDPAAYAPAYGVGLNAYGYNAYPYPSYGGAACSPGYSCAAYPAAPPAAQPPAASANSNFVNFGVGDLNTVQSPGMPQGNSGVSTLHGIRAW

>Mm_Nkx2.6
MLSSPVASTPFSVADILRLECQQKDSKTLSQWELHRNPVKPRYLRMNQESGWFESSDRAQAVPFRCTWETVLEMGSNPVGEPQTPPGTISRLGARNPMTDRGVGNLSGDMRRGGPVSTRTRPQRKSRVLFSQAQVLALERRFKQQRYLTAPEREHLASALQLTSTQVKIWFQNRRYKSKSQRQDQTLELAGHPLAPRRVAVPVLVLDGKPCLDPDVAAFLGPYKATSPYSCFGGYAGTPYDASYASRCTSASAGPGPLTPLASSGFSPGGQSAAPQGHLPATPQGVTAW

>Mm_Nkx2.8
MATSGRLGFTVRSLLNLPEQDAKPRVRREQQTCVPQTAAWLESECSHYLSSDESGLETSPADSSQLASLRRESPGSDPEKRRKRRVLFSKAQTLELERRFRQQRYLSAPEREQLARLLRLTPTQVKIWFQNHRYKLKRGRAPGITEPSDMAASSDLHAAPGLLRRVVVPVLVHDRPPSNNGRGEGTSAVPQDKCSARLATACPVPGYTAFGPGSALGLFPAYQHLAPPALVSWNW

>CsNK4
MMIPSPVGSTPFSVKDILNLERHQMTCSDDDRASMLYQAPCGGKNEELAMQSIYQMNNMEAGQGNGEIIKPRDGKGNKHHPNTMDTNNMAAPDPHFVIYSQSEKYSKDVGEVNYTGPANTSEAAASNLLHFSQNFQQHSYSDGRALANVAPFDMPRYGSDYFPSPNYTGYNLPFENPSSTEDPRSTLHSQTFPKPNNNAVTSASHMAPSEARGNFYQSPNHFDTVREAQENGEDFYSKTESPFPAYSQASSQSDQQPLPSFDRSTEKMYTEQRSADAGNQIFESSGSDPLVVDFPSPSGSPSKLPEDGSCFTDESLPRPFSGVDIRHDDSVAQVTDSEKSDAVSTCSTSPGESEKKKLEEDGLKSRHRTRRKPRVLFSQAQVFELERRFKQQRYLSAPEREHLAQMLKLTSTQVKIWFQNRRYKCKRMRQDKTLELASIGAPRRVAVPVLVRDGKSCLGGPGQVPHNSAPYNVTVTRYHNYPGSYSSCGYNTYPHNAPGYGPSAAAAAAVAGAAAAAYGNAATNGYNSFGPNMGGGQPGPTPYPTPGLHQQHPPPMAMGN
SPFQSSSVPPGHHVTQGGLHHHAPTDYMYKLGLCT


>Cs-TTF1
MSVSPKHSTTTPFSVTDILSPLEESYGNGSVDGIVSGRREFSELYCGMDSGTTSSTGIHVPNLGGGGSTGSISSPMPSYRQPVMGSPPHHSIHHQQMPVTNPYQSATGVNGAYNLHPMPTPPQTTFHTMSGTGGGPMYGNGGVSDLPPYNNVQSHWYGAPSNPDPRFVPSYPRLQIGYGSMMPGTGMDGMHKSLLPPSQRRKRRVLFSQAQVFELERRFKQQKYLSAPEREHLAQMIHLTPTQVKIWFQNHRYKNKRSLKDKQTQDMGVQQQQPQNSVNPQQQHTNEMTHSNPAQLQPIPSNIAPGGSRTPQETYSPVPSGIPPQQGMEEVSDAAHRMVHITNQNGEQSSPRQIAMSDGQACHETTIIESPNLQMSMPSVVHSTDSESGLVPFSSVIKLEEVNVESNETAAGNHGINVPQTHYLGSTHHHDLMNVNVAALNSVDAYQNHHHHNHIPIGNSVLHDPSILYSVYR

>Ci_TTF1
MSVSPKHSTTTPFSVTDILSPLEESYGTGSVEGIVSGRREFSELYSNMESGAASSAGIHVPNLGGGGSTGSISSPMTSYRQPMMGTPPHHSIHHQQMSSANPYQTAAGMNGSYNLHPMPTPPQTSFHAMPGSGGPTPYGNGGVPELPPYNNVQSPWYGAPSNPDPRFVPSYPRLQITGYGSMMPGTGMDGMHKTLLPPSQRRKRRVLFSQAQVFELERRFKQQKYLSAPEREHLAQMIHLTPTQVKIWFQNHRYKNKRSLKDKQTQEMSVQQQQQQQQQSQTSGNAQPQHTNDLSHSNPSQLQPVASAVPGGTRTPQETYSPVPTGVQASQPGMVDGNDGHRMVHISNQNGDQSSPRHLTMSDGSCHDSTIIESPNMQMPMPNVVQPTDTEAGLGPFSSVIKLEEVNADSAEGSGGNSGNIVPQTHYLGSTHHHDLMNVNVTTLNAVDAYQHHPHHNHIPIGNTVLNDQSILYSVYR

>Ci_Nk4
MIPSPVGSTPFSVKDILNLERHQMSCQSSADDRDTPHVVNGYPVSCNDSIKSEDTQVLDMHSIYHHSSMEATQQASGELVKGRFEKLSKPHSRQIETNNMAASDSQFDMFSQNENAKYSKGAIDTRYAEATNTADTAASNLLHFSQNFHQIPYGESRALVAPFDMPRYGGEYFSPTSNYSGGYTHPYDNPPSNVEASRSSSFHSQQFTKPNDDVISTTHSTSSATAGNFYHRPANNFEPVREPGLERCPDNGDEFYTKTESPFPAFNQQHQPTPQSEHHSMPSYDEMNEKMYTDQTSETDITNNSMFDSSSSEAVVVDFPSPSGSPNKQHEDGRFGGSCTDESLPRTFSAADIRQDESVTQVTDSEKSDNVSTCSTSPEETEKKKTEDDTLKSRHRTRRKPRVLFSQAQVFELERRFKQQRYLSAPEREHLAQILKLTSTQVKIWFQNRRYKCKRMRQDKTLELASIGPPRRVAVPVLVRDGKSCLGGPGQVSHNAAPYNVTVTRYHNYPSSYSSCGYNSYPPNPPGYGPSAAAAAAVAGAAAAAYGNAATNGYNGFPPS
MNGGQPGASPYPTPGLHQQHPPSMGVGNSPFQTSTVPTGHHMSQGGLHHHTPTDYMYKLGLCT
>Mo_Nk4

MQSSPQNIDVGYDYRQQVDGISIGATNPDHNVATMSGLNMAASLTSAYTNSVDVMSYNGAISIEGVEAKSKLNHSSEAVSSLLKALSPEQRYQREFYDSNASDRHGVNASTHDANFGFQSGFIQSEPANSSTSSVLSDVSSIQQSINILPNTSPISTHSASSYPSYASSTYLPVQDNVAELDNSYSSFAPHHPNPSSYSSFSSPSDHIDVQYNTSPGHSVDMQRQYYNVDKHNTTPLKRG

STSDVTPYSLGGSRSGSEGNVSAFADGLSSSSSQQQYSSTSTEVSSIDNVNNQTADSEKSDNVSNCSESPTDGEKKKTEDDASKNRPRTRRKPRVLFSQAQVFELERRFKQQRYLSAPEREHLAQILKLTSTQVKIWFQNRRYKCKRQRQDKTLELAGACPPRRVAVPVLVRDGKPCLGGPGGPMGGGNPSLQVPYSAAPYNVTVTPYHSYPSSYNSCGYNSYPHNPHANYSSATAPYGS

TQNYSHIAGNTLGATTTAVGGMGAGLHQTGQVGMGPSPFAPTMSSQVPQRNPHAPSDYMYKLGLCT

>Mo_Nkx2

MSDSPKAHHTSSTTTPFSVTDILSPLENSYGEAESSVINHGRSNVEETQELTPDDHRSYQYLHINHHQHSIDGNRHEIRQSEDQENFDRNSTHLATVATMDPGSVISASAALHMPTIGDSSVLGSISPQGSTSSQVYRHSMPLTTSQHSSMHHQQMPYQSMNTAAAMAAGMNGSYNLHSMPPPPQSSFHAMSAPAAAASGYCNGGMAELSPYNNVQSSATAGWYSSPTNPDPRFGTMSMS

RYLSPSPGMGMNTYGGMNMMGGGMDGMHKPILPSSQRRKRRVLFSQAQVFELERRFKQQKYLSAPEREHLAQMIHLTPTQVKIWFQNHRYKNKRSLKDKQGDQPTQQNQSQTNEHQKQQPQQQQVQQQQQTLMTQTQQCYSPASSGLQPSNGGQVPITIPNDSSQANHVMASDANGVSDSESNNSPQRMAKEVKEGMMPNHEICQQPTSQSSTEGSIASDHAPLMLLPYGSLHSNAVKME

EVDNENCMGGLSSVQQYVAPEQHQDMISVNVTTLNTAHATQESSGSPSPYLTHPQPHHHHLTASNGIVNNSILYGLYR

>Hr_Nkx4

MCRGQNIPKAVRFINEIGFNLFKFRPVTGSTFSILHVTDSAHAKSEKSDNLSNCSGSPRDEDKKKREEDGTKNRQRTRRKPRVLFSQAQVFELERRFKQQRYLSAPEREHLAQILKLTSTQVKIWFQNRRYKCKRQRQDKTLELASACPPRRVAVPVLVRDGKPCLGSPTGHMNGTHQLNPYSAPYNVTVTPYHNYPSSYSSCGYNSYHHPHNPAASYTAAAYGSAAASAGYNTLSGAAM

GAGMGGVGGLPGSALHHHQSATVGVAPSHFSAMQGQVQQGASIRNHSTTDYMYKLGLLKTVNSLTHIIRLTCSNACSSKRIIYHIYIFLNLIEISSTIRRFSIKTQRLFSGNSG*


>Hr_Ttf1
MSLSPKQHQSSSTTTPFSVTDILSPLEDHYAGSEVNRGNTNGEVNSNHNHCINISNSNILYNNQQLLSLEQQGHSSLSVRHYESCPTSATGPVHLSAMDPGSLVAVSAALNVPSIGDQSVMGSMSPTGGSVSSIPLYRHPMSIGTSQHGVHHQQMQATMPYQSMNSSAAMGMNGGYNLHTIPPSQSSFHSMSGAGSGSTGYCNGGMADLASYNNVQSSPGWYSTPTNPDPRFGTMPMSRYLTPSPGMGMNTYGGMNMMTGTGMDGMHKPMLPSSQRRKRRVLFSQAQVFELERRFKQQKYLSAPEREHLAQMIRLTPTQVKIWFQNHRYKNKRALKENGSDVQTPSQTTSNENSTQNQGPSQHQQQTNSLMQGQQTYSPNNQVNINCQQNEGGNQQHHMVESVADHQHDQGTQSPRRISVPVLVKDGKPCSAGSAGTPNGSIMTDGMDGVSQASAQESLNAETAIMNVYGNGHLKIDGCGNGNDNSLCVAGATLVAPSPQGHYIGTSQHQDLISVNVSTLSTENMPPMAYHPLQSQHHHLPGASSVVNNSLLYGIYR

>Pm_Nk4

MQLLHSTRLELASLVPAAPFRSLYNAGQCKEFAPQCDESDLVMRFCFADETCYLRYSEKSDNFSESNCSGSPEESDKKKGDEDGMKARHRTRRKPRVLFSQAQVFELERRFKQQRYLSAPEREHLAQILKLTSTQVKIWFQNRRYKCKRMRQDKTLELASIGPPRRVAVPVLVRDGKPCLGGPGAGIQNSHAPYSAPYNVTVTPYHNYPNSYNSCGYNSYSHPNPNAYAPGAAAAAAVAG

AAAAAYGTAVSAGYNGLSGPGMSGGQGGPMAGYPTPTLHPQHPNPVAMGSSPFGTSTMSPGQVGQPGLHPTPSDYMYKLGLCT*

>Pm_Nkx2

MSLSPKQTTTTPFSVTDILSPLDEPYGQHGPATVDDGGNLQTRREIHDSYSSASMDPGHSAVASSGLHVPSLGSSLSTGSTSPPMSSLYRQPMSMGTPPHHGVHHHQQIPAASVPYQSMPTAAAVAAGMNSGYNLHPMAPAPQATFHTMPGSGSSGYCNGTVADLPSYNNVQATGWYGAPANPDPRFGTVSMSRYLNPSAGMGMNTYGGVNMMSGTGMDGMHKSLLPASQRRKRRVLFSQ

AQVFELERRFKQQKYLSAPEREHLAQMIHLTPTQVKIWFQNHRYKNKRSLKDKQAQVDSVTVVTQQQQGSQNSNSQNHHSEINHQQQQQQAMASGNHTPQQTYSPATVAGSGNTGQVQVVTSMVDNADGSHHMVHGMVQQNQQNGDHSPSRRAVMSDVSVGSDRGVEESPNMHLALTPVAQSNLPEPGMPNGNEVGMGQFATTVKIEEVNVVGSEASVPHSHYLSSTNNSQHQDLMNVNV

TTLNTVDAYQQAQHHGHHHLAIGNTMLNDSSLLYGVYR*

>Bf_Nk4
MMLPSPVNSTPFSVKDILNLADTGFDDSESGGLPSPEETMLATVQQNHVRCEQLTIGSTTTHHGVCGSTAAMLTSTQKQCMQAGNIDRPCESGNMGLLKKDDRKDDSDDEPPKIPPQAQRQRQRRKPRVLFSQAQVFELERRFKQQRYLSAPEREQLAQMLKLTSTQVKIWFQNRRYKCKRQRTDKTLELTHPPPRRVAVPVLVRDGKPCQMTPPPYSAPYNVSVNPYGYNMNVHGTYSSYNFNSGYSTNYTTYNATVPPVQTVTTMQPPPYSLQPGVHQGIRAW

>Bf_Nkx2-1
MESISPKQTTPFSVTDILSPLEEMYKKPMDGTMTGGYAGTMNAAAAGMGAGGYRQQVTQPLQHQSMNVPVSNPYMHVPTQLSHGMANPYCNGNVSDLPHYNEHVRNTASSWYGANPDPRFSFPRLMGGHSGGMGNMGMSLGTIEGPKPILPTTQRRKRRVLFSQAQVYELERRFKQQKYLSAPEREHLAQLINLTPTQVKIWFQNHRYKCKRQDKERQKSSTDQPSQQQQQQQQPQQQQQQQQQQQVSQHQAGQVQGQAGQQNMCAAGNSPRRVAVPVLVKDGKPCGNTPSTTPVTGVTANMSAATPQLNPQSQANIIGTTVATVNVNGLNSHMSSGNYANNTMSSCSSSQYLLQQGRAW

>Mm_NK3
MAVRGSGTLTPFSIQAILNKKEERGGLATPEGRPAPGGTEVAVTAAPAVCCWRIFGETEAGALGGAEDSLLASPARTRTAVGQSAESPGGWDSDSALSEENEGRRRCADVPGASGTGRARVTLGLDQPGCELHAAKDLEEEAPVRSDSEMSASVSGDHSPRGEDDSVSPGGARVPGLRGAAGSGASGGQAGGVEEEEEPAAPKPRKKRSRAAFSHAQVFELERRFNHQRYLSGPERADLAASLKLTETQVKIWFQNRRYKTKRRQMAADLLASAPAAKKVAVKVLVRDDQRQYLPGEVLRPPSLLPLQPSYYYPYYCLPGWALSTCAAAAGTQ

#*******************************************************************************

#MYH Type II Myosin Heacy chain

# class II type Myosins

>Bs_MYH9/10/11
AMNPPKFTKVEDMADLTCLNEASVLHNLTDRYYSGLIYTYSGLFCVVVNPYKFYPIYTEKVIEVYKGRKRHEVPPHVFAITDTAYRSMLQDREDQSILCTGESGAGKTENTKKVIQYLAHVAASPKSGKVSHGGTNLSYGELEQQLLQANPILEAFGNAKTVKNDNSSRFGKFIRINFDASGYIAGANIETYLLEKARVIQQASNERTFHIFYQLITGANKELQKDLLLEDISKYRFISNGSLSVPGHKDSDEFQETLNAMEIMNIAPSDVKSMLKVISAVMQFGNMDFKKERSSDQACMPDNTVAQRLCALLGIDVVSFTKALLKPKIKVGRDFVQKAQTKEQVDFAVEALAKAIYERLFRWLVHRINRSLDRSTRQGASFIGILDIAGFEIFETNSYEQLCINMTNEKLQQLFNHTMFILEQEEYRREGIDWKFIDFGLDLQPCINLIENPSNPPGILALLDEECWFPKATDKSFVEKLVKEQGNHPKFMKPKSLKDKGDFIIVHYAGKVEYTAKKWLMKNMDPLNDNVTDLLHNSNDEFVAGLWKDMSKVVGMENLVGADKPFGSSTYKTRKGMFRTVGALYKDQLGRLMTTLRNTNPNFVRCIIPNYEKKPGKIVAHLVLEQLRCNGVLEGIRICRQGFPNRIPFQEFRQRYEILTPNVLPKGFMDGKKAAVKMLEELDLDENLFRIGQSKIFFRGGVLAHLEEERDL

>Bs_MYH2
BEAFGNAKTIRNDNSSRFGKFVRTHFGNTGKLASGDIETYLLEKSRVIFQQGGERGFHIFYQICSGAKPELLESLLVTTDPYSYRYISQGEVTVSGLDDSDELRATDSAFDVLGFTPEEKMGIYKIMGSIMHAGNMKFKQKPRDEQAEADGTEDADKISYLLGINSSEFVKAMTQRVRVGNDYVTKGQTVKQVYYSCGAMCKAVYDRLFSWLVKRINETLSTKMPRSFFIGVLDIAGFEIFDFNSFEQLCINFTNEKLQQFFNHHMFVLEQEEYKREGIDWEFIDFGLDLQACIELIEKPLGIMSILEEECMFPKASDSTFKEKLYTNHLGKSNNFIKPRPQIKRKFEAHFELIHYAGIVGYNIEGWLEKNKDPLNNSVVALYRKSTLKVLATVWEGYVSPDDVTEQKKAGRGKRQKGGSFQTVSSLHRESLNRLMTNLRSTQPHFVRCIIPNEMKQPGMMDNSLVLHQLRCNGVLEGIRICRKGFPSRILYAEFKQRYRILNPNAAPEGQFMDSKKATEKLMASLEMDSSQFKFGLTKIFFKAGMLGELEDMRDQRLSLIITMIQSRARGKQMRIEYQRMLERRQALIVIQANLRSYFAVRNWEWMRLMFKIKPLLKSAESAKEREAIEKEMGDVKEVLEKEKKRRQELEENQVSLIQEKNDLVLQLTAEQENLQDAEDRCDQLIRTKVELESKIKDIVERLEDEEEVSNDLVSKKRKLEDECSELKKDIDNLELTLAKVEKEKHATENKVRNLTDEVTTLEETVAKIQKEKKALQEAHQQTLDDLQLEEDKVNSLTKQKSKLEQQVDDLEASLEHEKKVRMDMERAKRKLEGDLRLAQESIMDLENDRQRNEEKLKKKEFEFNQLSTKLEDEQALISQLQRKIKELQSRIEELEEELDAERAARAKAEKQKTDMSRELEELSERLEEAGGATSAQVELNKRREAEFSKLRRELEESNLAHESMLSTLRKKHADSDAELTEQVDNLQRVKQKLEKEKSELKMEIDDLSTNVESVTKGKLQFEKLSRNLEDQLSEAKAKNEEMGRELSELNQKHARLSSEKGELGRAVEEKEAIMSQYTRTRNSMQQQIEELKRLLEEESKAKSALAHGVQSSRHDNDLLREQYEDEQEAKAELQRALSKANAEVAQWRTKYETDAIQRTEELEEAKKKLAARLQDAEEAVEAAQAKASSLEKTKQRLQGELEDMSIEIEKSNSAAAALDKRQRNFDKVLAEQKQKSEELQVDLEQSQKEARSLSTELFKMKNAYEEALDALETIKRENKNIQEEISDLTDQLGEGGKSIHELEKARRSLEHERTELQAAFEEAEAAVENEESKVLRLQVELAQTKQDFERRLQEKDEEIDNARRNASRSIESMQATLDAESKARSEAQRIKKKMESDINDLEMQIAHTNRQAHDAIRQLKDSQASSKEMQMQVDDAIRRNEDMQEQQSVTERRANLLQAELEEMGASLEQAERGRKLAEGELMEVSERANLLHAQNTALINQKRKLEGEIQTMQSDVEEAVQEQRDAEEKAKKAITDAAMMAEELKKEQDQSAHLERMKRNMEQNVKDLQMRLDEAEQVALKGGRKQVQKLETRVRELENEVDAEQRRHVETSKLLRKAERRNKEVTYQSDEDKKNLLRMQDLVEKLQVKVKTYKRQCEEAEEQANVNLSKYRKLAHELDDAEERAEMAESALAKLRSKARESVGSGLGGKLGSSYKGVE

>Bs_MYH3
MNPPKFEQASDMANMTFLNEATVLYNLRSRYASMRIYTYSGLFCVCVNPYKWLPVYGARVVGMFRGKKRNEVPPHLFCVADNAYHDMLLDRENQSILITGESGAGKTENTKKVIQYFANIAASQKQDAAAEKKGNLEDQIVQTNPVLEAWGNAKTIRNNNSSRFGKFIRIHFGTTGKLSGGDIESYLLEKSRVIFQLSAERSYHIFYQMLSSKKPELLDQLCVTDNAADYRYVSNGVLTVDNMDDTLEFGYTDEAFDILGFTASEKFESYKITCGCMLFGNMVYKQRPREEQAEVENVDVADKVAHLFGINSNELCKSITRPRVKVGNEYVQKGQTQDQCYYGTGALSKAVYDRYFKWLVMRLNETLDTKLPRNYFIGVLDIAGFEIFDFNSFEQLCINFTNEKLQQFFNHHMFVLEQEEYKKEGIEWEFIDFGMDLQACIDLLEKPMGIFSVLEEESIVPKATDTTFKNKLYTGHEKKSKAFLKPKPGRKSNADFTVAHYAGLVDYNVTGWLEKNKDPLNESVLGLFRKSTSKLMAGLFPEDAPDTGGKKRKKKGGSFQTVSALYREQLNKLMTNLRNTKPHFVRCLIPNEMKQCGTMDAALVLGQLRCNGVLEGIRICRKGFPNRMQYPEFKQRYQILAAKKVAGIVDSKKATEIILSHIELDTAMYKIGHTKVFFKAGILADLEDKRDE

>Bs_MYHE
MNPPRYKKCEDMVNMTHLNEASVLNNLKERYESFMIYTYSGLFCVTVNPYKMLPVYNSYVIAAYKGKRRTEMPPHLYSIADNAYTEMLMNRENQSMLITGESGAGKTVNTKKVIQYFALVAAQGGGGQNDDKGTLEDQIVQCNPAMEAFGNAKTARNDNSSRFGKFIRIHFGSTGSLASGDIEHYLLEKSRVIFQQGGERNYHIFYQLISGSKPELIDQLLLTKDPYDYKSISQGVVTVDGLDDGAELILTDDAFKILGFTPEEVNGIYRIMAGIMHQQNMKFKNKQREEQAEPDGTEDADKVSYLLGLNSADFLKYLCHPRVKVGNEYVTKGQTVNQVSYGMGALSKGVFEKHFNWLVKIINNSLSTKLPRSYFIGVLDIAGFEIFDFNSFEQLCINFTNEKLQQFFNHHMFVLEQEEYKKEGIDWVFIDFGMDLAACIELIEKPLGIMSILEEECMFPKASDKTFLDKLYQNHLGKTKSFGKPVKKTKFEAHFELHHYAGSVGYSITDWLEKNKEPLNNSVVELYKKSSLKLMQTIWEGYVSADDNSGGGGKGGKRKKGGSFQTVSSMHRESLNKLMTNLRSTAPHFVRCIVPNETKTPGSMDNHVVLHQLRCNGVLEGIRICRKGFPNRLPYGDFKQRYRILNPNAVPDGQFLDSKKASEKVLTSLENIDHEKYKLGHTKVFFRAGMLGVLEELRDN


>Hr_MYHE
MIASLVQLSLLDSCYSASNVIHIQRKNSLYYVERLWSITSLYKMATLDLSMFQDAAQYLRMSQQEMLELQTQKPDGKKYAWVPDKQHAYLQGEVISSEGGKAKLKTLEEGKEVTLKEDDVQLMNPPRYNKCEDMVNMTHLNEASVLKNLNDRYKAFMIYTYSGLFCVTVNPYKMLPVYAPYVIQAYKGKRRTEMPPHLYSIADNAYTEMLMNRENQSMLITGESGAGKTVNTKKVIQYFALVAAADDGSSGNDDKGTLEDQIVQCNPAMEAFGNAKTMRNDNSSRFGKFIRIHFGSTGSLASGDIEHYLLEKSRVIWQQGGERNYHIFYQIISGGKPELIDQLLVTKDPYDFKSISQGVVTINNLDDGEELRLTDEAFHVLGFTQEEINGIYRIMAAIMHQQNMKFKNKQREEQAEPDGTEDADKVAYLLGMNSADFLKYICHPRVKVGNEFVTKGQTVPQVTYAIGGLSKGVFEKHFNWLVKIINQSLSTKLPRSYFIGVLDIAGFEIFDFNSFEQLCINFTNEKLQQFFNHHMFVLEQEEYKKEGIDWVFIDFGMDLAAVIELIEKPLGIMSILEEECMFPKATDNSFKEKLYQNHLGKTKAFGKPVKKTKFEAHFELHHYAGTVAYSVTDWLEKNKEPLNNSVVELYKKSSLKLMQTIWEGYVSTDDASSGGGKGKRKKGGSFMTVSSMHRESLNKLMTNLRSTAPHFVRCIVPNDTKTPGEMDPHIVLHQLRCNGVLEGIRICRKGFPNRLPYGDLKQRYNILNPNVVPEGQFLDSKKASEKLLGSLDIDHTQYRLGHTKVFFRAGMLGVLEELRDSKLSTIFKLIQARLRAKLMQIEYNKLIEQRVAVRVIQSNLRAFFGVRDWEWMKLMFKIKPLLQTAEAAKEFEQLEAENEELKTNYEKESKRRKELEESQVSLIQEKNDLLLQVQSEQDRIEDAEDRCDQLIRTKVELDGKVKELTERLEDEEELNNELVSKKRKLEDECSELKKDIDDLEITLAKVEKEKHATENKLKNLQEELASQDEQIAKLQKEKKALQEAHQQTLDDLQSEEDKVNSLTKQKSKLEQQVDDLEASLEQEKKLRMDLERTKRKLEGDLRLTQETVMDLENDKQRLEEKLKKQEFEYSQLATKLEDEQALVMQLQKKIKELQARIEELEEELEAERAARAKVEKQRADLSRELEELSERLEEAGGATAAQIELNKRREAEFSKLRRELEESNLAHEATVSTLRKKHADSSAEMSEQIDNLQRVKQKLEKEKSEMKMEVDDLAANVESVTKAKLNYEKMARNLEEQYSESKTKCDNFMKEVNELNAAKARLTSEVGELSRQLEEREHLMAQLTRSKNSSSQQIEELKRVVEEETKAKAALAHSVQASRHDNDLLREQYEEEQEGKAELQRALSKANAEVAQWRNKYETDAIQRTEELEEAKKKLAARLQEAEEQVEATQAKCASLEKTKNRLQGELEDLTVDLERSNSAAAALDKKQRNFDKVLSEHKQKEEEIQVELEQAQKEARGLSTELFKMKNAYEESLDALETVKRENKNLQEEIADLTDQLGEGGKSIHELEKAKRTLEHERNEIQAALEEAEGAIEGEESKVLRLQVELAQIKQDFERRLSEKEQEIDNQRRNQQRSLESMQTTLDSESKSRQEAVRMKKKMEGDLNDLEIQLGHATRQAGEAQKTVKTAQAHIKDLEMQVDESQRQSDDFQEQFAVIERRENLVKAEIDELRAALEQAERGRKLAETELLESSERSNLLHTQNTALINQKRKLEGELQNMQAEVEEAVQEQRNAEEKAKKSITDAATMAEELKKEQDLSSHLERMKKNMEQTVKDLQQRLDEAEQVALKGGKKQVQKLETRVRELENELDSEQRRNSESVKNQRKFERKLKEVTYQAEEDKKNLVRIQDLVDKLQIKVKTYKRQAEEAEEQANQNMSKYRKLQHELDDAEERADMAESSLNKLRSKAKNM*

>Hr_MYH3
MENRSEFSPAALLSSRRRSRRFSKQTSQAEKQALNDTDDVYRLLKYGVKAHEVSDDTNIHTKNCNFEHDHTFQKSENSVVLKSVCKTSMSRFVETNTVSQNSTQFLQNCDSTTVGGEEEEEKTLENSFISPQFGEESLEFEESIGFDSNYSIDSYNLYNNELHIEEEVFDKDSIELKVSSATNMASESGSDYEVDTDVVNSWLDEPTAVQSSKRTFSSEKTIKSARIPASSKMVGVDLTTGNFVCDSRTVQRDGNELANVHDMEQETVADLEERNEQLVKAKQELETTVADLSERLEEEEATNEQLSAAKRKLEKQNDDLQHDVEDLESNVSRLEKDKQSLELKIRNLNADVDQRDQNIDRLQKEKKQLEQINQQTLEDLQAMEDKANHLGKLKIKLEQQIEDVEDQLEQERRLRTDLDKAKRKLETDLHNAHETISELEKDKAGLEDVIRKKDFEANQLATRLEDESNLVAQLQRKIKELQARIEDLESELEQERQARTKVERSRNELQRELEGLGEQLEEAGGATVAQFELNKRREADYLKLRREYEETVLQSDAALSSLKKKHSDMVSELTDQVESLTRVKGKIDKERSMLRIEVDDLASQLEEVTKIKARAEANCRSLEDTVVDSKVRIEEHIRTINEVTIMKSKLTNENIDISHQLEEAENKVNSLTRFKSNLTSQTEELKRLVEEETKAKQSLGHALQAARHDLDLMREQVEEEQEGKAELQRALSKANAEVANWRTKYETDAIQRTEELEEAKKKLAIRLQEAEEQTENALAKCASLEKTKIRLTNEVEDLTIDLERANATIAALDKRQRDFDKELASWKMRVEELQAELDAAQRECRNYSTEIYKLRASYEETIEQIEIVKRENKTLSEEIGDLMDQLGAGGRTLHESEKARKKAEMESDELRTALEEAEGALEIEEGKVLRLQLELTQVKADIDRRLQEKDEEFESTRKNHARAIESLQASLDIEIKARTDATRAKKKIEAQLNDLEMQLDHANRNLAEQLKLVRKFQLTIKEMQDQMDEDQRIHDELREQYSIQERKLNILLSELEETRSSLESNERARKMAEHELLEITERVNIMSAQNSALSSAKRKLETENDQMRGEFEEALAEARNADERAKKAVGDAARMAEELRQEQLHVMSVEKIKKSLEIQVHEISIKLDEAEANALKGGRRALAALQMRVKDLENELEAEQRRHAETTKNFRKMDRRLKELSFQADEDQKNQLRMQELVEKLQAKLKQYKKMTEEAEEQANLNLAKYRKVTHDLEEAEERADISESALSKVRSKSRYMSGSTGPGGSYSYSISRKVVTTKGASSSAYES*


>Hr_MYH9/10/11
MQLEELDLDGNLYRTGKSKIFFRGGVLAHLEEERDLRLTDIIIQFQAACRGFLARKFFAKRKDQLRAIRVIQRNALAYLKLRNWPWWRLFTKVKPLLDVTRQEEDLHAKEKELKTVMEKRDQLEEDLSHVEKKYAQVCEEKLALAERLAQEQELAEEAEDSRARLALKKVDLEVLLNELETRLEDEEERSLSLSNERKKLQVCIADLEDNLEKEEAARQKLQLEKVTSEAKIKKYEEDLAITDENLNKIQRERKSLEERVNELQASLADEEDKAKTYGKQNHKYEAIIADLEERLKREEKLRMELEKIKRKLEAEIAELKDTIEDLQLQRDDLKSQCQRKDDEIAKLNEKLDQDQAERTTLTKVLREVENAKSELQEDLEAERVQRAKSEKNKRDLKEELDALKAELEDSIDSTTAQQETRVKREMELESLRNHLKEENETHEQQIQDMRKKQTMQLEELQVEIDSYKKSKSSLEKAKNAVESECSEVIEELRLVTQAKADSDSKRRKQDTVIQEYSAKLQDTERQRNDLQDKYTKSQNELDTFSQNLEQLESQLSRSTKDVSQLNMQVQDLQAQLQEESSAKLKLTSQIRGLDDDRNNLEEQLEEEEEARRNVEKQLATANAAMIELKKTMAGDNEAIEHLEENKKRLQRDLEEKTQALEDKSAQADKLDKTNKRLTNEVDDLNLELDKYRQQMTMMDKKQRQFDKALADERQISTKYADEKDRAVREAMEKEARAISLMDQCESLMAKVEDLEKKRKAQQQELEELVSSTDASGKNVHGLEKSKRALESQLEEMKTQLEELEDELQLTEDSKLRLEVNMQAIKSNYDRDVQQREEQAEEKRKSILKQLREMEAELEEERKQKSAAIAAKKKLEMDLSDIDGHLEGAAKAKEDALKQLKKSQAQMKECIRDLDDVRASRDDLALVARDLEKKSKSKDADILQLHEDLATSERSRKNIEQERDDLSDELQNSMKEKTTLQDEKRKMEIQLSDMEEEKEDVEQSLEFTEDKLRKAIAQAEQAQNELQMERSSAQKAENARAQLDRQNKELKLKLAEMEQMVRTKQKNAIAAMESKILNLEDQLEAESKDRAAAFKQVRRTEKKAKEISMQVEDERRQTDLYKEQVDKLNTRVKGLKRQVDDAEEEVSRANASKRKIQRELDETMEQTEALQRENNQLRNKVRLSELNLYMQCTDQRDNHKMNPLTWYVLFDATVVIVTLLNLFRFTPVALCPRLNFAFLFLIQSGGRSDLPRSKRDRYIHIVLKP*


>Mo_MYHE_v1
MTVSASHRDSLNRLMNNLKSTSPHFVRCIVPNGTKTPGTMDGEIVLHQLRCNGVLEGIRICRKGFPNRLPYGDFKQRYRILNAEACPEGQFLDSKKASEKLLGSLDVDHTQYKLGHTKVFFRAGFLGVLEELRDDKLSSIFKLIQARIRAKLMKIEYNKLIEARVATRVIQSNLRAFFGLRNWEWMKLMFKIKPLLQTAEAAKEREQLEKEFEETKEQLEKESKRRKELEESQVSLIQEKNDLVLQLQSEQDRIDDAEDRCDQLIKTKVELDGKIKEIQERLEDEEELNNDLVSKKRKLEDECSELKKDIDDLEITLAKVEKEKHATENKLKNLQEELASQDEQIAKLQKEKKALQESHQQTLDDLQSEEDKQEFEYSQLATKLEDEQALVSQLQKKIKELQARIEELEEELEAERAARAKIEKQRSDLSRELEELSERLEEAGGATAAQVELNKRREAEFSKLRREFEESNLSHEATVSTLRKKQADTAAEMSEQIDNLQRVKQKLEKEKSEMKMEIDDLATNVESVTKAKLNYEKMARNLEEQLSEAKTKGDNLTKEVNELNAAKARLSSENGEYQRQLEEREHLMAQLTRSKNSSAQQIDELKRVVEEETKAKSALAHAVQAARHDNDLLREQYEEEQEAKADLQRALSKANAEVAQWRNKYETDAIQRTEELEEAKKKLASRLQDAEEQVEAAQAKAGSLEKTKNRLQGELEDMTIDLERVNSAAASLDKKQRNFDKVLAEHKQKYEEIQVELEQSQKEARSLSTELFKMKNAYEESLDGLETVKRENKNLQEEIADLTDQIGEGGKSIHELEKAKRSLEQERNEIQAALEEAEAAIEGEESKVLRLQVELAQNKQDFERRLAEKEEEIDNQRRNSQRSLESMQTTLDSESKSRQEAVRIKKKMEGDLNDLEIQLGHSNKQVGESQKQSKSLQAHVKDLELQVDESQRQSEDCQEQLAVVERRSNLLMGEIEELRSALEQAERGRKLAETELLESSERSNLLHTQNTALINQKRKLEGEIQTMQGEVEESVQEQRNAEDKAKKAIVDAATMAEELKKEQDMSAHLERMKKNMEQTVKDLQQRLDEAEQVALKGGKKHVQKLETRVRELENELDAEQRRNNDALKGQRKYERKLKEVTYQAEEDKKNLTRIQDLVDKLQIKVKTYKRQAEEAEEQANANLGKYRKLQHELDDAEERADMAESALNKLRSKARDIKYRFCIPAVYKVNEVFNSKEYLLYRNTCGLFKTTCGSSSF


>Mo_MYH3
MLKLMHVLSLCVRNSILIDLVIATAITFYQNGTKSIERDCEGKIDLVTLLEIFFIGECGPEDDPMLFLRPTEKEKLQFASQSYDSKKNIWVPNKKEGYLKGEVVKREDGKVTVNTSRGDELVLKEDDIQQQNPPKFEQTSDMANMTFLNEASVLYNLRTRYACMRIYTYSGLFCVCINPYKWLPVYGARVVGMFRGKKRSEVPPHLFCIADNAYHDMLQDRENQSILITGESGAGKTENTKKVIQYFANIAAASAIKSEAEQKKGNLEDQIVQTNPVLEAWGNAKTVRNNNSSRFGKFIRIHFGTSGKLSGGDIESYLLEKSRVIFQLKAERSYHIFYQVLTSGRPELLNSLMVEPDAGQYKNVSNGVLVVDNMDDKMEFDFTDEAFDILGFTATEKAESYKVTIGTMLFGNMLYKQRPREEQAEVDSTDIADKVAHLFAINSSELCKGMTRPRVKVGNEFVQKGQTAQQCTYSTGALCKAIYDKYFKWMVFRLNETLDTKLPRNYFIGVLDIAGFEIFEFNSFEQLCINFTNEKLQQFFNHHMFVLEQEEYKKEGIEWTFIDFGMDLQKCIDLLEKPMGVLSILEEESIVPKATDDTFKNKLYDQHEKKSEAFLKPKPGRKGNAHFTVKHYAGEVGYNVTGWLEKNKDPLNNTVVSLFQKSSSKLMSSLFPVVADEVGKKKKKGGSFQTVSALYREQLNKLMTQLRNTKPHFVRCIIPNEMKQPGVLDSHLVLNQLKCNGVLEGIRICRKGFPNRMQYPEFKQRYQILAAKKVASIVDSKKATEIILSHIELDTNMYKIGHTKVFFKAGILAELEDQRDEVLAVIVTGMQARARGKLMRIEFKKMIERRRAAKVIQRNVRKFVQFRDWQWWKLYTKVKPLLNVVRVEDELKAKDEEIADLKGKLEKEGVLRKEYEEKSVTLLSEKNDLMLQLQAEQETLAEMEERNELLVKQKQDLDTQVADLSERVEEEESNNEKLSAAKRQLEKQSEDLRSDVEDLEGNIGRLEKDKQGLELKVRNLNSDVEQRDDNINRLQKEKKQLEQVNQQTLEDLQAMEDKANHLGKLKIKLEQQIEDVEDQLEQERKIRADVEKAKRKLESSLHEANDNIMDLEKDKSNLEDNLKKKEFELNQRSSQLEDEQNLVLQLQRKIKELQGRIEELESELESERSARSKVERNRNEIARELDQLGEQLEEAGGATQAQIELNKRRESDYLKLRRDYEEAVMQSDANLGSLKKKHSEMQQELSEQVEGLTRVKAKIEKERNQLRVEVEDLTSQLDDMSKAKSRAEANSRVLEEQSMDLKVRIEEHTRSVTELTSFQTKLTNENIDISHQLEDAESKASGLSRIKANITAQVEELKRSFEEESKAKQSLNHALQAARHDLDLMREHVEEEQEGKMELQRALSKANSEVANWRTKYETDAIQRTEELEEAKKKLALRLQEAEEQTENALAKCASLEKTKLRLTNEVEDLTIDLERANATIAALDKRQRDFDKELAIWKQRVEELQADLDQSQRECRNYSTEIYKLRASYDECIEQIEIIRRENKVLTEEIGDLTDQLGAGGRTLHESEKARKKAEMECEELRTALEEAEGALEIEEGKVLRLQLELTQVKADIDRRLQEKDEEFESTRKNHARAMESMQASLDAEIKGRTDAVRAKKKLEAQLNDVEMQLEHANRNLAEQHKLCRKLQTTIKEMQDQMDEDQRIHEELREQYSLQEHKIHILTSELEETRVSLETNERARKQAEAELIDATERLNALSSQNAALSGLKRKLETENEQMRGELDDALSEARNADDRAKKAVGDAARMAEELRQEQQHVMSIERVKKSLEVQVHETQLKLEEAEANALKGGKRQIASLQARVKDLNSDLDAEQRRHAETLKNFRKMERRMKELAFQADEDQKNQARLQELVERLQAKLKQYKKMAEEAEEQANANLAKYRKVTHELDEAEERAEISESALNKIRSKSKYLSGSGGGAGGSYSYTISRKNNVLKSSASMPYDSYSHKNSLIGGFASYHLLSGRSTPTHDNDVFSSNIPSWRTSASILEDNKSNTSTEKFSADGGAKEQQEFVMKGDARNPNSTECELDVAEDDESSRQNVENVGTGDENVDGTIEVVARRRSRKHDLNSSSSSIKNDE

>Mo_MYH2
MAAWDPAAAKDAAQYLRLSQQDLIQIHAQPYAGKKAVFIPHKTNVFQKAEVVGPSEKKGCKVVKLEDGKEMNVKEENIEEQNPPKFSIIEDMANMTYLNEPAVLQNLGQRYERFMIYTYSGLFCVTINPYKMLPVYKDYVVGCYKGKRRAEMPPHIFSIADNAYNDMLRNRENQSMLITGESGAGKTVNTKRVIQYFATVAALSDAADTKPKDGSKGTLEDQIIQANPALEAFGNAKTIRNDNSSRFGKFIRIHFGNTGKLASGDIEKYLLEKSRVIFQQGGERGFHVFYQLCSGGKPELMETLLITSDPYEYKFCSQGDTTVQGLDDVEELIATDTAFDVLGFSPEEKMGIYKIMGSIMHAGNMKFKQKPREEQAEADGTEDADKLCYLMGINTAEYMKALTLPRVKVGNDFVTKGQTVQQVYYSVGALCKAVYEKVFSWLVKRINETLSTKLPRSFFIGVLDIAGFEIFEFNSFEQLCINFTNEKLQQFFNSHMFVLEQEEYKKEGIEWEFIDFGLDLQACIDLIEKPLGVFSILEEECMFPKATDKSFQEKLYTNHLGKSNNFIKPRPQIKRKFEAHFELIHYAGIVGYNITGWLEKNKDPLNTSVVGLYKKSSLKVLATVWESYVSPEEAAAAKKSGGGKGRRQKGGSFQTVSSLHRESLNRLMTNLRSTQPHFVRCIIPNEKKQPGMMDNQLVIHQLRCNGVLEGIRICRKGFPSRIVYSEFKQRYRILNPSSIPEGQFVDSKKATEKLMASLELDHSQYRFGHTKIFFKAGMLGMLEDMRDERLAKIITLIQARARGKQMRIEYLKMLERRQDCLQACKVIQANIRAYLSVRNWEWMKLMFKIKPLLKTAESAKEREIIEKEVEDLREELEKEKKKRKDLEENQVTLIQEKNDLVLQLTAEQDNLQDAEDRCDQLIKTKVELESKIKDYDERLEDEEETNNDLVSKKKKLEDENGELKKDIDDLELTLAKIEKEKHATENKARNLTEEVASLEEMLAKSNREKKALQETHHQTLDDLQTEEDKVSSLSKQKAKLEQQVDDLEVGLEQEKKTRMEMERIKRKLEGDLRLSHETIMDLENDKQILEEKLKKREFEYNQLMSKLEDVESLVAQLQRKIKELQARIEELEEELDAERAARAKVEKQRMDMQRELEEISERLEEAGGATSAQIELNKRRELELSKLRRELDEATLAHDAMVVSLRKKHADEVATLSEQVDNLQRVKHKLEKDKSEMKMEIDDLTTNVETITKQKVHFERMSRNLEDQFTESKSRNDQMTRELNELNLKHERLSNEKSELSRAIEEKETIMSQYTRTRNSMQQQMEELKRQLDEETKAKNALAHGVQGARHDNDLLREQYEEEQEAKAELQRALSKANAEVAQWRTKYETDAIQRTEELEEAKKKLAARLQEAEEEVEAAQAKASSLEKTKLRLQGEIEDITIDLERSNSAAAGLDKKQRNFDKMIAEYKQKQEELQVEFEQSQKECRSVSTELFKMKNAYEEALDALETIKRENKNLQEEISDLSDQLGEGGKSIHELEKARRSLEHERTEIQAALEEAEAAVENEESKVLRIQIELAQEKQEHERRMREKEEDIENTRRNCQRSLESMQATLDSESKSRSEAHRIKKKLESDLNDMEVQLSHAFRQAQEAQRLLKEHQAHGKDSQIQLDEMIRRNEDLEEQQATSERRCNLLQGEIEEIRASLEQAERGRKLAENELMEVSERANLLHTQNTALINQKRKMESEIHSMSADIEELVQEQRNAEEKAKKAITDAAMMAEELKKEQDQSAHLERMKKNNEQVVKDLQMRLDEAEQVALKGGRKQVQKLETRVRELENEVDGEQRRQVDTSKMLRKAERRMKEVTYQAEEDKKNLSRMKDLVEKLQNKVKTYKRQCEETEEQANINLAKYRKLQHELDDAEERADMAESALNKLRAKARDSVSAYSITKNID


>Pm MYH3b
MSDDAHRLSVRRRTRNRSIINPKDVEESKSYEVTNSLQEVTRSVHDVITVTQSSEVEVTTVRRSRASRDLKRLSVNTGSTSSPANSTRHSNDVDSSYSPRHSENMEQHLDSPRSHEANDDVGVTRRCSLMNSQRSGKLMELRNRYTSDEVDSMTFEDEEVELKSERSQQSDATDEQYDVTTNGAHESVTSWLMNDCSSSASEDSDSSSDSESGRGETKAPSRQISAENLNELVNGCELSAEEVNEPLVEQEHVNESINSRLPPMEQYRLCFEEQDNLADAEERNEQLVKNKQDLENQVADLSERLEEEEANNEQLSAAKKKLDRQVEDLTHDIEEAESNINRLDKDKQNLELKLRGLSVDLEQREESISRLNKEKKQLDQVNQQTLEDLQAMEDKANHLSKLKTKLEQQVEDVEDSLEQERKHKNDLEKSKRKLEADLRNANDTITDLEKDKSSLEDALRKKDFEINQLNGRVEDEQTQCVQLTRKLKELQARIEDSENELEMERMARSKVEKNRNELVRELDHLGEQLEEAGGATVAQIELIKRREADYIKLRRDYEESVMQSDATISQMKKKHQDTVTELVEQVENLVRVKNKVEKDRTQLQMELDDVSSQLEEISKMKARAEANVRVMEEQVTDYKIRVEENNRSITELNMFKSKLMSENSDTSHMLEEAENKVNGLSRAKSNMTSTIEDLKRQLDEETKSKQSLNHALQAARHDLDLLREQVEEEQEGKSELQRALSRANTEIANWRTKYETDAIQRMEELEEAKKKLAIRLQEAEEQTENALAKCASLDKTKIRLQNEVEDLTIDLERANATISALDKKQRHFDKEIATWQQRVEELQAELDASQREARNYSTEILKLKASYEESIEHLEIIKRENKNLSEEINDLTDQLTTGGKSLHELDKAKKKAELECEELRSALEEAEGALELEESRVLRLQLELTQVKADIDRRLQEKDEEFDSTRKNHQRAIESMEASLEVEIKSRNDAMRGKKKAEAALNDSELALDHANRNLAEQMKLVRKLQVTIKEIQDQMDEDQRIHEELREQYSIQERKLTIMMSELEETRSALESNERARKHAESELMEISDRINVLIAQNSALSSARRKLETDNDQLRTELEEALIEARNADERAKKATGDAARMSEELRQEQQRIISLERIKKTLEVQVHEISIKLDDAEANALKGGRKALATMQARLKDFENELAAEQRRHAETLKNYRKMDRRLKELTFQADEDQKNQTRMQELVEKLQLKLKQYKKMAEEAEEQANNNLTKYRKATHELEEAEERAEISESALNKVRSKSRYMTNLISESTNCNVTSESSTSFMLTSTTSSPQPPSSNHILEASIRFASEVSPRFDFKHSALTSPTEILRHKSITSLQSFDDSELEPDKKRSHSPSGVSESVTSQSSISSQSGSQDMTNDL*

>Pm_MYH3a
MPGCGIGQVGECGPGDDPMPFLAPTEKEQMAFASQTYDGKKNVWVPHKKEGYIKAEVLDSSGGKVTVKTIKNETITVKEDDVQQMNPPKYEQTSDMANMTHLNEASVLYNLRSRYASMRIYTYSGLFCVCVNPYKWLPVYGAKVVVMFRGKKRAEMPPHLFSVADNAYHDMLMDRENQSILITGESGAGKTENTKKVIQYFANIAAQTTQKPDSEKKANLEDQIVQTNPVLEAWGNAKTIRNNNSSRFGKFIRIHFGTSGKLSGGDIESYLLEKSRVIFQLPAERSYHIFYQIMSSGKPDMIEQLCITTNPKDYQWISQGVLTVDNMDDKQEFAFTDEAFDVLGFTEDEKVGSYRLTCGVMVFGSMRYKQKPRDEQAEVDSVEIADKCSYLFGINSNELCKAITRPRVKVGTEYVQKGQNVDQCKNSTGALGKAVYNNLFRWIVFRLNITLDTKLPRNYYVGVLDIAGFEIFEFNTFEQLCINFTNEKLQQFFNHHMFVLEQEEYKKEGIQWTFIDFGMDLQDCIELLEKPMGIFSILEEESIVPKATDETFKNKLYEQHDKKSKAFVKPKVSGKKSGNAHFSVRHYAGIVDYNVDGWLNKNKDPLNESVLSLFRKSSNKLMSGLFPEVKEEGGGKKKKKGGSFQTVSALYREQLNKLMTNLRNTKPHFVRCIIPNEMKQPGIMDAHLVLAQLKCNGVLEGIRICRKGFPNRLPYPEFKQRYQVLAAKLVANMVDSKKATETVVSHVELDTALYKIGHTKIFFKAGVLADLEDKRDEILSIIVTKMQSRVRGKLMRIEFKKMLERQRAARAIQRNIRKFLQFRDWQWWKLYTKVKPLLNVVRVEDELKAKDDEIADLKDKYGKEEKLRKEYEEKCVHLLSEKNDLTLQLQATRDGEPLTRGSRSDPPREF*


>Pm_MYH9/10/11
EYRREGIEWKFIDFGLDLQPCINLIERPSNPPGILSLLDEECWFPKATDKSFVEKVVKQLGDHPKFQKAKQLKGSSDFSIIHYAGKVEYTAERWLMKNMDPLNDNVTDLLHNSLDPFVSAIWKDMSKVVGMEQLVAGGENKFSQAYKTRKGMFRTVGALYKDQLQKLMTTLRNTNPNFVRCIIPNYEKKPGKIVAQLVLEQLRCNGVLEGIRICRQGFPNRVPFQEFRQRYEILTPNVLPKDIMDGKKAAIKMIHALDLDENLYRIGQSKIFFRGGVLAHLEEERDLKLTDIIIQFQASCRGFIARRNFSKRKDQLRAIRVIQRNGLAYLKLRNWPWWRLFTKVKPLLQVTRQEEDLNAKERELKAVQEKKDALEKDLQDIEKKFAQTVEEKNALSDQLIAEQELYADAEEMRSRLATKKTELEEILQDMESRLDDEEERNQLLGQEKKKMQLNIKDLEEQLDEEEAARQRLQLEKVTLDAKCKKMDEDLNMMEDTHAKIQRERKALEERVNELESGLGDEEEKAKMLTKLKNKNEAVIQDLEERLKKEEKHRQELEKIRRQLEAEIAELRDQIADLQMQVEELKSQNQRKDEELASLQERFDQEQSARNQTNKSLRDLQNSHAELQEDLEQERSQRNKAEKQKRDLSEELEALKTELEDSLDTTNAQQELKSQRERELENLKRTLDSETQSFETQLQQMRQKHNGQVEQFQDEIDSLKRFKSGLEKSKHSLDSENQELQEELRLITSAKNDSETKRRKQEAQIQELSAKLQDTERNRNEFQDKYNRIQSEYDALVVNLEQVETQLTRSQREA

>Pm_MYH2
MGSIMHMGNMKFKQKPREEQAEADGTEDADKITYLLGINSAEFVKSLLQPRVRVGNDYVTKGQTVQQVYYGTGALSKAVYDRLFKWLVKRINETLSTKLQRNFFIGVLDIAGFEIFDFNSFEQLCINFTNEKLQQFFNHHMFILEQEEYKREGIDWEFIDFGLDLQACIELIEKPLGIMSILEEECMFPKATDMTFKEKLYTNHLGKSNNFIKPRPQIKRKFEAHFELIHYAGIVGYNIVGWLEKNKDPLNNSVVNLYKKSTMKVLATVWDTYLSPEEAMAGAKKGGGGGRRQKGGSFQTVSSLHRESLNRLMTNLRSTQPHFVRCIIPNEMKKPGYLDSQLTLHQLRCNGVLEGIRICRKGFPSRILYAEFKQRYRILNPNSIPDGQFVDSKKATEKLMASLELDTAQYRFGNTKIFFKAGMLGTLEDMRDERLTIIITRMQARARGKNMRIEFKKMLERKQACSLIQANIRAYLAVRNWEWMRLMFKIKPLLKSAETAKEREAIEKEMADAVENLDREKKRRQELEDSQVALIQEKNDLVLQLSAEQENLQDAEDRCEQLIRSKVDLEGKIKDLQERLEDEEEANNDMVSKKRKLEDECSELKKDIDDLELTLARVEKEKHATENKLKNMAEKLMTLEESLDKAHKEKKALQEAHQQTLDDLQAEEDKVNSLTKHKSKLEQQVDDLEASLEHEKKIRMELERTKRKQEGDIRLLQETIMDLENDRQRLEERIKKKEFEYNQLATKLEDEQALIAQLQRKIKELQARIEELEEELDAERSARAKVEKQRTELSRELEELSERLEESGGATSAQMELNKRREIEYSKLRREFEESNLAHEAMISTMRKKNADTLAELSEQVDNLQRVKQKLEKEKSEMKMEIDDLASNVEVVTKHKLSFEKMSRNLEDQLSETKSKNDEMQRELNEVNAKFARLSSEKGELSRFLEEKESLMNQYTRTRNSLQQQLEELKRQVEEEMKAARHDNDLLREQYEEEQEAKAELQRALSKANAEVAQWRNKYETDAIQRTEELEDAKKKLACRLQEAEEAVEXSRLQEAEEAVEAAQAKASSLEKTKIRLQGELEDVVVDLEKSNAAAAALDKKQRNFDKVLSDHKEKQEELRVDFEQSQKESRSLSTELFKMKNAYEEALEALETIKRENKNLQEEISDISDQLGEGGKSIHELEKAKRALEHERTELQGALEEAEGAVENEESKVLRLQVELAQVKQDFERRVHEKDEEIDNTRRAGQRSIESMQATLDAEGKARSEAVRIKKKMESDLNDLEMQLNHANRQAQDAVRQLKDGHAANKDLQIQYDEATRHNEDLEEQMSMVERRANLLMAELEEMRTSLEQAERGRKMAESELMEVSERSNLLHTQNTALINQKRKLEGELQNMQGDIEESIQEQRNAEEKAKKAILDAAMMAEELKKEQDQSSHLERMKKNMEQTVKDLQMRLDEAEQVALKGGRKQLQKLETRVRELENELDGEQRRSVDITKQLRKSERRIKEVSYQAEEDKKNLTRMQDLLDKLQIKVKTYKRQCEETEEQANLNLAKYRKLQHELDDAEERADVAESALNKLRAKARDGTSYANYGKKMREDKWNNHTAPGSVPDDEEISKSAVSIRSIREQKIERQKHREEQRQRRKKQEPAIMVATDPPRGGSAGKSRPSTASRREESLPLVGGSHNSNNKYNHATYINPALENDDQSVTTIKVAPSNNPISSVPDKPPVMQEYSSSDDGVDSMKRSDTERRMAEQGIQQSIDYDRDSEEEDIEGGMMPVGPRPSTARPNTANRRPPSATKNFDGKKVGWNSALPPASLNGGNLFETTRASGKIMSPVPTTVSIDRSKLLSTESGADDVFITADVFSPISQVFEQLPGSPSTPLPPDPTLNRSEETIAAPEKLADMELLDLDDDEIPDDKINTCSKSFTKSDKTGSRTVLRENWTCASYQLSDEEGQSLCDETTTIENLDGFDPGPRVSHEPCKVAARSPPSGQYVVESECRDLSHQADPVLDAIKNLQQLLTKTQARALASNKCSQVVLHMQNRILLEQKFTTDSPAAYEPNSEEELRDWQNISDICAYLSGLVLQCQLSTNKSACERHPLVNPSYRAATSVVQSILQCDVTISQLLLAIDVVLKQYGLESINCSQLATEKGKNRLVQEESRQHRKVTEDRPMSSGGRSQVPGMDLDDLESFCLQPAPQGYTIKCKISRDKKGLDRHAYPTYYLHYERDDGKKTFILAGRKRKRSKTSNYLISVDATDLSRGGESFIGKLRSNIMGTKFTVYDQGIKWGNPGIATDRSNLREELASICYETNVLGFKGPRKMTVIIPGMDMSHERVRFKPNHVSISYHSHTVPPPDRETILSRWQNKNMENLIELSNKTPVWNEETQSYVLNFRGRVTQASVKNFQIVHQSDPEYIVMQFGRVAEDVFTVDYNYPMNAVQAFAIALSSFDSKLACE*

>Pm_MYHE
MATFDYEAVQDAAPYLRMTHEKILENQTKKPDGKKYVWFPDKQNAYVVGELIKNEGGKCTIKSMDEGKEITVKEDDLQEMNPPRYEKCEDMANMTFLNEASVLNNLRSRYESFMIYTYSGLFCVTVNPYKMLPVYAPYVISAYKGKRRTEMPPHLYSIADNAYASMLMNRENQSMLITGESGAGKTVNTKKVIQYFALVAAFGGKQDDSKGTMEDQIVQCNPAMEAFGNAKTVRNDNSSRFGKFIRIHFGSTGMLASGDIEHYLLEKSRVIYQLEGERNYHIFYQIISGGKPELIDQLLVSKDPYEYRTISQGVVTVDNLDDSEELLLTDEAFRILGFSQEEISGIYRLMAGILHQQNMKFKNKQREEQAEPDGTEEADKVAYLYGLNSTDFIKYLCHPRVKVGNEFVTKGQSCSQVSYGLGALSKALFGRHFDWLVKLINQTLSTKLPRSFFIGVLDIAGFEIFDCNSFEQLCINFTNEKLQQFFNHHMFVLEQEEYKKEGIQWTFIDFGMDLAACIELIEKPLGIMSILEEECMFPKASDNSFKEKLYQNHLGKSKAFGKPTKKTKYEAHFELYHYAGTVGYNITGWLEKNKDPVNNSVVDLYKKASLKLMQTIWDGWVSPDEASGGGKGGKRKKGGSFNTVSSLHRQSLNNLMTNLRSTSPHFVRCLIPNEQKQPGMMESHLVLHQLRCNGVLEGIRICRKGFPNRIPYGDFKQRYRILNPNAAPEGQFMDSKKSSEKLLGSIDIDHESYKLGHTKVFFRAGMIGRLEEMRDNKLSSIFKLVQARMRGMIMRLEYQKMIERRQACRVIQSNLRAYFGMATWEWMKLMFKIKPLLKTAEAAKELEVLEKDYEECKVNLEKEVKRRKELEEMQVSFIQEKNDLLMQLQAQQDQIDDGEDRCDQLIKTKVELDGKIKELTERLEDEEELNNELVSKKRKLEDECSELKKDIDDLEITLAKVEKEKHATENKLKNLQEELATQDEQIAKLQKEKKALQEAHQQTLDDLQSEEDKVNSLTKQKSKLEQQVDDLEASLEQEKKLRIELERTKRKLEGDLRLTQETVMDLENDKQRLEEKLKKQEFEYSQLATKLEDEQALVSQLQKKIKELQARIEELEEELEAERAARAKVEKQRADLSRELEELSERLEEAGGATAAQIELNKRREAEFAKLRRELEESNLSHEATVSTLRKKHADTSAEMSEQIDNLQRVKQKLEKEKSEMKMEIDDLASNVESVTKSKLNYEKMSRNLEEQLNEAKMKNDNFTKEVNELNAAKARLSSENGEFGRQLEEREHLMAQLTRSKNSSSQQIDELKRVVEEETKAKAALAHAVQAARHDNDLLREQYEEEQEAKAELQRALSKANAEVAQWRNKYETDAIQRTEELEDAKKKLAIRLQDAEEQVEAMQAKASSLEKTKNRLQGEIEDLTIDLERSNSAAASLDKKQRNFDKVLAEHKQKHEEVQVELEQSQKESRSLSTELFKMKNAYEESLDALETVKRENKNLQEEIADLTDQLGEGGKSIHELEKAKRTLEHERNEMQAALEEAEGAIEGEESKVLRLQIELAQVKQEFERRVSEKEEEIDNQRRNQQRAIESMQTTLDSESKARQEAVRIKKKMEGDLNDLEIQLGHATRQASEAQKQAKSVQAHIKDLEMQVDEAQRHSEDLQEQTAVIERRGNLLTAEIEELRSALDQAERGRKLAETELLESSERSNLLHTQNTALINQKRKLEGELQTMQSEVEESVQEQRNAEEKAKKAIVDAATMAEELKKEQDLSSHLERMKKNMEQTVKDLQQRLDEAENIALKGGKKQVQKLETRIRELENELDSEQRRNGDSVKSQRKLERKLKEITYQGEEDKKNLTRIQDLVDKLQIKVKTYKRQAEEAEEQANTNLSKYRKLQHELDDAEERAEMAESALNKMRSKARDRKE*


>Hs_MYH7
MGDSEMAVFGAAAPYLRKSEKERLEAQTRPFDLKKDVFVPDDKQEFVKAKIVSREGGKVTAETEYGKTVTVKEDQVMQQNPPKFDKIEDMAMLTFLHEPAVLYNLKDRYGSWMIYTYSGLFCVTVNPYKWLPVYTPEVVAAYRGKKRSEAPPHIFSISDNAYQYMLTDRENQSILITGESGAGKTVNTKRVIQYFAVIAAIGDRSKKDQSPGKGTLEDQIIQANPALEAFGNAKTVRNDNSSRFGKFIRIHFGATGKLASADIETYLLEKSRVIFQLKAERDYHIFYQILSNKKPELLDMLLITNNPYDYAFISQGETTVASIDDAEELMATDNAFDVLGFTSEEKNSMYKLTGAIMHFGNMKFKLKQREEQAEPDGTEEADKSAYLMGLNSADLLKGLCHPRVKVGNEYVTKGQNVQQVIYATGALAKAVYERMFNWMVTRINATLETKQPRQYFIGVLDIAGFEIFDFNSFEQLCINFTNEKLQQFFNHHMFVLEQEEYKKEGIEWTFIDFGMDLQACIDLIEKPMGIMSILEEECMFKATDMTFKAKLFDNHLGKSANFQKPRNIKGKPEAHFSLIHYAGIVDYNIIGWLQKNKDPLNETVVGLYQKSSLKLLSTLFANYAGADAPIEKGKGKAKKGSSFQTVSALHRENLNKLMTNLRSTHPHFVRCIIPNETKSPGVMDNPLVMHQLRCNGVLEGIRICRKGFPNRILYGDFRQRYRILNPAAIPEGQFIDSRKGAEKLLSSLDIDHNQYKFGHTKVFFKAGLLGLLEEMRDERLSRIITRIQAQSRGVLARMEYKKLLERRDSLLVIQWNIRAFMGVKNWPWMKLYFKIKPLLKSAEREKEMASMKEEFTRLKEALEKSEARRKELEEKMVSLLQEKNDLQLQVQAEQDNLADAEERCDQLIKNKIQLEAKVKEMNERLEDEEEMNAELTAKKRKLEDECSELKRDIDDLELTLAKVEKEKHATENKVKNLTEEMAGLDEIIAKLTKEKKALQEAHQQALDDLQAEEDKVNTLTKAKVKLEQQVDDLEGSLEQEKKVRMDLERAKRKLEGDLKLTQESIMDLENDKQQLDERLKKKDFELNALNARIEDEQALGSQLQKKLKELQARIEELEEELEAERTARAKVEKLRSDLSRELEEISERLEEAGGATSVQIEMNKKREAEFQKMRRDLEEATLQHEATAAALRKKHADSVAELGEQIDNLQRVKQKLEKEKSEFKLELDDVTSNMEQIIKAKANLEKMCRTLEDQMNEHRSKAEETQRSVNDLTSQRAKLQTENGELSRQLDEKEALISQLTRGKLTYTQQLEDLKRQLEEEVKAKNALAHALQSARHDCDLLREQYEEETEAKAELQRVLSKANSEVAQWRTKYETDAIQRTEELEEAKKKLAQRLQEAEEAVEAVNAKCSSLEKTKHRLQNEIEDLMVDVERSNAAAAALDKKQRNFDKILAEWKQKYEESQSELESSQKEARSLSTELFKLKNAYEESLEHLETFKRENKNLQEEISDLTEQLGSSGKTIHELEKVRKQLEAEKMELQSALEEAEASLEHEEGKILRAQLEFNQIKAEIERKLAEKDEEMEQAKRNHLRVVDSLQTSLDAETRSRNEALRVKKKMEGDLNEMEIQLSHANRMAAEAQKQVKSLQSLLKDTQIQLDDAVRANDDLKENIAIVERRNNLLQAELEELRAVVEQTERSRKLAEQELIETSERVQLLHSQNTSLINQKKKMDADLSQLQTEVEEAVQECRNAEEKAKKAITDAAMMAEELKKEQDTSAHLERMKKNMEQTIKDLQHRLDEAEQIALKGGKKQLQKLEARVRELENELEAEQKRNAESVKGMRKSERRIKELTYQTEEDRKNLLRLQDLVDKLQLKVKAYKRQAEEAEEQANTNLSKFRKVQHELDEAEERADIAESQVNKLRAKSRDIGTKGLNEE

>Hs_MYH9
MAQQAADKYLYVDKNFINNPLAQADWAAKKLVWVPSDKSGFEPASLKEEVGEEAIVELVENGKKVKVNKDDIQKMNPPKFSKVEDMAELTCLNEASVLHNLKERYYSGLIYTYSGLFCVVINPYKNLPIYSEEIVEMYKGKKRHEMPPHIYAITDTAYRSMMQDREDQSILCTGESGAGKTENTKKVIQYLAYVASSHKSKKDQGELERQLLQANPILEAFGNAKTVKNDNSSRFGKFIRINFDVNGYIVGANIETYLLEKSRAIRQAKEERTFHIFYYLLSGAGEHLKTDLLLEPYNKYRFLSNGHVTIPGQQDKDMFQETMEAMRIMGIPEEEQMGLLRVISGVLQLGNIVFKKERNTDQASMPDNTAAQKVSHLLGINVTDFTRGILTPRIKVGRDYVQKAQTKEQADFAIEALAKATYERMFRWLVLRINKALDKTKRQGASFIGILDIAGFEIFDLNSFEQLCINYTNEKLQQLFNHTMFILEQEEYQREGIEWNFIDFGLDLQPCIDLIEKPAGPPGILALLDEECWFPKATDKSFVEKVMQEQGTHPKFQKPKQLKDKADFCIIHYAGKVDYKADEWLMKNMDPLNDNIATLLHQSSDKFVSELWKDVDRIIGLDQVAGMSETALPGAFKTRKGMFRTVGQLYKEQLAKLMATLRNTNPNFVRCIIPNHEKKAGKLDPHLVLDQLRCNGVLEGIRICRQGFPNRVVFQEFRQRYEILTPNSIPKGFMDGKQACVLMIKALELDSNLYRIGQSKVFFRAGVLAHLEEERDLKITDVIIGFQACCRGYLARKAFAKRQQQLTAMKVLQRNCAAYLKLRNWQWWRLFTKVKPLLQVSRQEEEMMAKEEELVKVREKQLAAENRLTEMETLQSQLMAEKLQLQEQLQAETELCAEAEELRARLTAKKQELEEICHDLEARVEEEEERCQHLQAEKKKMQQNIQELEEQLEEEESARQKLQLEKVTTEAKLKKLEEEQIILEDQNCKLAKEKKLLEDRIAEFTTNLTEEEEKSKSLAKLKNKHEAMITDLEERLRREEKQRQELEKTRRKLEGDSTDLSDQIAELQAQIAELKMQLAKKEEELQAALARVEEEAAQKNMALKKIRELESQISELQEDLESERASRNKAEKQKRDLGEELEALKTELEDTLDSTAAQQELRSKREQEVNILKKTLEEEAKTHEAQIQEMRQKHSQAVEELAEQLEQTKRVKANLEKAKQTLENERGELANEVKVLLQGKGDSEHKRKKVEAQLQELQVKFNEGERVRTELADKVTKLQVELDNVTGLLSQSDSKSSKLTKDFSALESQLQDTQELLQEENRQKLSLSTKLKQVEDEKNSFREQLEEEEEAKHNLEKQIATLHAQVADMKKKMEDSVGCLETAEEVKRKLQKDLEGLSQRHEEKVAAYDKLEKTKTRLQQELDDLLVDLDHQRQSACNLEKKQKKFDQLLAEEKTISAKYAEERDRAEAEAREKETKALSLARALEEAMEQKAELERLNKQFRTEMEDLMSSKDDVGKSVHELEKSKRALEQQVEEMKTQLEELEDELQATEDAKLRLEVNLQAMKAQFERDLQGRDEQSEEKKKQLVRQVREMEAELEDERKQRSMAVAARKKLEMDLKDLEAHIDSANKNRDEAIKQLRKLQAQMKDCMRELDDTRASREEILAQAKENEKKLKSMEAEMIQLQEELAAAERAKRQAQQERDELADEIANSSGKGALALEEKRRLEARIAQLEEELEEEQGNTELINDRLKKANLQIDQINTDLNLERSHAQKNENARQQLERQNKELKVKLQEMEGTVKSKYKASITALEAKIAQLEEQLDNETKERQAACKQVRRTEKKLKDVLLQVDDERRNAEQYKDQADKASTRLKQLKRQLEEAEEEAQRANASRRKLQRELEDATETADAMNREVSSLKNKLRRGDLPFVVPRRMARKGAGDGSDEEVDGKADGAEAKPAE


>Hs_MYH6
MTDAQMADFGAAAQYLRKSEKERLEAQTRPFDIRTECFVPDDKEEFVKAKILSREGGKVIAETENGKTVTVKEDQVLQQNPPKFDKIEDMAMLTFLHEPAVLFNLKERYAAWMIYTYSGLFCVTVNPYKWLPVYNAEVVAAYRGKKRSEAPPHIFSISDNAYQYMLTDRENQSILITGESGAGKTVNTKRVIQYFASIAAIGDRGKKDNANANKGTLEDQIIQANPALEAFGNAKTVRNDNSSRFGKFIRIHFGATGKLASADIETYLLEKSRVIFQLKAERNYHIFYQILSNKKPELLDMLLVTNNPYDYAFVSQGEVSVASIDDSEELMATDSAFDVLGFTSEEKAGVYKLTGAIMHYGNMKFKQKQREEQAEPDGTEDADKSAYLMGLNSADLLKGLCHPRVKVGNEYVTKGQSVQQVYYSIGALAKAVYEKMFNWMVTRINATLETKQPRQYFIGVLDIAGFEIFDFNSFEQLCINFTNEKLQQFFNHHMFVLEQEEYKKEGIEWTFIDFGMDLQACIDLIEKPMGIMSILEEECMFPKATDMTFKAKLYDNHLGKSNNFQKPRNIKGKQEAHFSLIHYAGTVDYNILGWLEKNKDLNETVVALYQKSSLKLMATLFSSYATADTGDSGKSKGGKKKGSSFQTVSALHRENLNKLMTNLRTTHPHFVRCIIPNERKAPGVMDNPLVMHQLRCNGVLEGIRICRKGFPNRILYGDFRQRYRILNPVAIPEGQFIDSRKGTEKLLSSLDIDHNQYKFGHTKVFFKAGLLGLLEEMRDERLSRIITRMQAQARGQLMRIEFKKIVERRDALLVIQWNIRAFMGVKNWPWMKLYFKIKPLLKSAETEKEMATMKEEFGRIKETLEKSEARRKELEEKMVSLLQEKNDLQLQVQAEQDNLNDAEERCDQLIKNKIQLEAKVKEMNERLEDEEEMNAELTAKKRKLEDECSELKKDIDDLELTLAKVEKEKHATENKVKNLTEEMAGLDEIIAKLTKEKKALQEAHQQALDDLQVEEDKVNSLSKSKVKLEQQVDDLEGSLEQEKKVRMDLERAKRKLEGDLKLTQESIMDLENDKLQLEEKLKKKEFDINQQNSKIEDEQVLALQLQKKLKENQARIEELEEELEAERTARAKVEKLRSDLSRELEEISERLEEAGGATSVQIEMNKKREAEFQKMRRDLEEATLQHEATAAALRKKHADSVAELGEQIDNLQRVKQKLEKEKSEFKLELDDVTSNMEQIIKAKANLEKVSRTLEDQANEYRVKLEEAQRSLNDFTTQRAKLQTENGELARQLEEKEALISQLTRGKLSYTQQMEDLKRQLEEEGKAKNALAHALQSARHDCDLLREQYEEETEAKAELQRVLSKANSEVAQWRTKYETDAIQRTEELEEAKKKLAQRLQDAEEAVEAVNAKCSSLEKTKHRLQNEIEDLMVDVERSNAAAAALDKKQRNFDKILAEWKQKYEESQSELESSQKEARSLSTELFKLKNAYEESLEHLETFKRENKNLQEEISDLTEQLGEGGKNVHELEKVRKQLEVEKLELQSALEEAEASLEHEEGKILRAQLEFNQIKAEIERKLAEKDEEMEQAKRNHQRVVDSLQTSLDAETRSRNEVLRVKKKMEGDLNEMEIQLSHANRMAAEAQKQVKSLQSLLKDTQIQLDDAVRANDDLKENIAIVERRNNLLQAELEELRAVVEQTERSRKLAEQELIETSERVQLLHSQNTSLINQKKKMESDLTQLQSEVEEAVQECRNAEEKAKKAITDAAMMAEELKKEQDTSAHLERMKKNMEQTIKDLQHRLDEAEQIALKGGKKQLQKLEARVRELEGELEAEQKRNAESVKGMRKSERRIKELTYQTEEDKKNLLRLQDLVDKLQLKVKAYKRQAEEAEEQANTNLSKFRKVQHELDEAEERADIAESQVNKLRAKSRDIGAKQKMHDEE

>Hs_MYH10
MAQRTGLEDPERYLFVDRAVIYNPATQADWTAKKLVWIPSERHGFEAASIKEERGDEVMVELAENGKKAMVNKDDIQKMNPPKFSKVEDMAELTCLNEASVLHNLKDRYYSGLIYTYSGLFCVVINPYKNLPIYSENIIEMYRGKKRHEMPPHIYAISESAYRCMLQDREDQSILCTGESGAGKTENTKKVIQYLAHVASSHKGRKDHNIPGELERQLLQANPILESFGNAKTVKNDNSSRFGKFIRINFDVTGYIVGANIETYLLEKSRAVRQAKDERTFHIFYQLLSGAGEHLKSDLLLEGFNNYRFLSNGYIPIPGQQDKDNFQETMEAMHIMGFSHEEILSMLKVVSSVLQFGNISFKKERNTDQASMPENTVAQKLCHLLGMNVMEFTRAILTPRIKVGRDYVQKAQTKEQADFAVEALAKATYERLFRWLVHRINKALDRTKRQGASFIGILDIAGFEIFELNSFEQLCINYTNEKLQQLFNHTMFILEQEEYQREGIEWNFIDFGLDLQPCIDLIERPANPPGVLALLDEECWFPKATDKTFVEKLVQEQGSHSKFQKPRQLKDKADFCIIHYAGKVDYKADEWLMKNMDPLNDNVATLLHQSSDRFVAELWKDVDRIVGLDQVTGMTETAFGSAYKTKKGMFRTVGQLYKESLTKLMATLRNTNPNFVRCIIPNHEKRAGKLDPHLVLDQLRCNGVLEGIRICRQGFPNRIVFQEFRQRYEILTPNAIPKGFMDGKQACERMIRALELDPNLYRIGQSKIFFRAGVLAHLEEERDLKITDIIIFFQAVCRGYLARKAFAKKQQQLSALKVLQRNCAAYLKLRHWQWWRVFTKVKPLLQVTRQEEELQAKDEELLKVKEKQTKVEGELEEMERKHQQLLEEKNILAEQLQAETELFAEAEEMRARLAAKKQELEEILHDLESRVEEEEERNQILQNEKKKMQAHIQDLEEQLDEEEGARQKLQLEKVTAEAKIKKMEEEILLLEDQNSKFIKEKKLMEDRIAECSSQLAEEEEKAKNLAKIRNKQEVMISDLEERLKKEEKTRQELEKAKRKLDGETTDLQDQIAELQAQIDELKLQLAKKEEELQGALARGDDETLHKNNALKVVRELQAQIAELQEDFESEKASRNKAEKQKRDLSEELEALKTELEDTLDTTAAQQELRTKREQEVAELKKALEEETKNHEAQIQDMRQRHATALEELSEQLEQAKRFKANLEKNKQGLETDNKELACEVKVLQQVKAESEHKRKKLDAQVQELHAKVSEGDRLRVELAEKASKLQNELDNVSTLLEEAEKKGIKFAKDAASLESQLQDTQELLQEETRQKLNLSSRIRQLEEEKNSLQEQQEEEEEARKNLEKQVLALQSQLADTKKKVDDDLGTIESLEEAKKKLLKDAEALSQRLEEKALAYDKLEKTKNRLQQELDDLTVDLDHQRQVASNLEKKQKKFDQLLAEEKSISARYAEERDRAEAEAREKETKALSLARALEEALEAKEEFERQNKQLRADMEDLMSSKDDVGKNVHELEKSKRALEQQVEEMRTQLEELEDELQATEDAKLRLEVNMQAMKAQFERDLQTRDEQNEEKKRLLIKQVRELEAELEDERKQRALAVASKKKMEIDLKDLEAQIEAANKARDEVIKQLRKLQAQMKDYQRELEEARASRDEIFAQSKESEKKLKSLEAEILQLQEELASSERARRHAEQERDELADEITNSASGKSALLDEKRRLEARIAQLEEELEEEQSNMELLNDRFRKTTLQVDTLNAELAAERSAAQKSDNARQQLERQNKELKAKLQELEGAVKSKFKATISALEAKIGQLEEQLEQEAKERAAANKLVRRTEKKLKEIFMQVEDERRHADQYKEQMEKANARMKQLKRQLEEAEEEATRANASRRKLQRELDDATEANEGLSREVSTLKNRLRRGGPISFSSSRSGRRQLHLEGASLELSDDDTESKTSDVNETQPPQSE


>Hs_MYH3
MSSDTEMEVFGIAAPFLRKSEKERIEAQNQPFDAKTYCFVVDSKEEYAKGKIKSSQDGKVTVETEDNRTLVVKPEDVYAMNPPKFDRIEDMAMLTHLNEPAVLYNLKDRYTSWMIYTYSGLFCVTVNPYKWLPVYNPEVVEGYRGKKRQEAPPHIFSISDNAYQFMLTDRENQSILITGESGAGKTVNTKRVIQYFATIAATGDLAKKKDSKMKGTLEDQIISANPLLEAFGNAKTVRNDNSSRFGKFIRIHFGTTGKLASADIETYLLEKSRVTFQLKAERSYHIFYQILSNKKPELIELLLITTNPYDYPFISQGEILVASIDDAEELLATDSAIDILGFTPEEKSGLYKLTGAVMHYGNMKFKQKQREEQAEPDGTEVADKTAYLMGLNSSDLLKALCFPRVKVGNEYVTKGQTVDQVHHAVNALSKSVYEKLFLWMVTRINQQLDTKLPRQHFIGVLDIAGFEIFEYNSLEQLCINFTNEKLQQFFNHHMFVLEQEEYKKEGIEWTFIDFGMDLAACIELIEKPMGIFSILEEECMFPKATDTSFKNKLYDQHLGKSNNFQKPKVVKGRAEAHFSLIHYAGTVDYSVSGWLEKNKDLNETVVGLYQKSSNRLLAHLYATFATADADSGKKKVAKKKGSSFQTVSALFRENLNKLMSNLRTTHPHFVRCIIPNETKTPGAMEHSLVLHQLRCNGVLEGIRICRKGFPNRILYGDFKQRYRVLNASAIPEGQFIDSKKACEKLLASIDIDHTQYKFGHTKVFFKAGLLGTLEEMRDDRLAKLITRTQAVCRGFLMRVEFQKMVQRRESIFCIQYNIRSFMNVKHWPWMKLFFKIKPLLKSAETEKEMATMKEEFQKTKDELAKSEAKRKELEEKLVTLVQEKNDLQLQVQAESENLLDAEERCDQLIKAKFQLEAKIKEVTERAEDEEEINAELTAKKRKLEDECSELKKDIDDLELTLAKVEKEKHATENKVKNLTEELSGLDETIAKLTREKKALQEAHQQALDDLQAEEDKVNSLNKTKSKLEQQVEDLESSLEQEKKLRVDLERNKRKLEGDLKLAQESILDLENDKQQLDERLKKKDFEYCQLQSKVEDEQTLGLQFQKKIKELQARIEELEEEIEAERATRAKTEKQRSDYARELEELSERLEEAGGVTSTQIELNKKREAEFLKLRRDLEEATLQHEAMVAALRKKHADSVAELGEQIDNLQRVKQKLEKEKSEFKLEIDDLSSSMESVSKSKANLEKICRTLEDQLSEARGKNEEIQRSLSELTTQKSRLQTEAGELSRQLEEKESIVSQLSRSKQAFTQQTEELKRQLEEENKAKNALAHALQSSRHDCDLLREQYEEEQEGKAELQRALSKANSEVAQWRTKYETDAIQRTEELEEAKKKLAQRLQDSEEQVEAVNAKCASLEKTKQRLQGEVEDLMVDVERANSLAAALDKKQRNFDKVLAEWKTKCEESQAELEASLKESRSLSTELFKLKNAYEEALDQLETVKRENKNLEQEIADLTEQIAENGKTIHELEKSRKQIELEKADIQLALEEAEAALEHEEAKILRIQLELTQVKSEIDRKIAEKDEEIEQLKRNYQRTVETMQSALDAEVRSRNEAIRLKKKMEGDLNEIEIQLSHANRQAAETLKHLRSVQGQLKDTQLHLDDALRGQEDLKEQLAIVERRANLLQAEVEELRATLEQTERARKLAEQELLDSNERVQLLHTQNTSLIHTKKKLETDLMQLQSEVEDASRDARNAEEKAKKAITDAAMMAEELKKEQDTSAHLERMKKNLEQTVKDLQHRLDEAEQLALKGGKKQIQKLETRIRELEFELEGEQKKNTESVKGLRKYERRVKELTYQSEEDRKNVLRLQDLVDKLQVKVKSYKRQAEEADEQANAHLTKFRKAQHELEEAEERADIAESQVNKLRAKTRDFTSSRMVVHESEE

>Hs_MYH14
MAAVTMSVPGRKAPPRPGPVPEAAQPFLFTPRGPSAGGGPGSGTSPQVEWTARRLVWVPSELHGFEAAALRDEGEEEAEVELAESGRRLRLPRDQIQRMNPPKFSKAEDMAELTCLNEASVLHNLRERYYSGLIYTYSGLFCVVINPYKQLPIYTEAIVEMYRGKKRHEVPPHVYAVTEGAYRSMLQDREDQSILCTGESGAGKTENTKKVIQYLAHVASSPKGRKEPGVPGELERQLLQANPILEAFGNAKTVKNDNSSRFGKFIRINFDVAGYIVGANIETYLLEKSRAIRQAKDECSFHIFYQLLGGAGEQLKADLLLEPCSHYRFLTNGPSSSPGQERELFQETLESLRVLGFSHEEIISMLRMVSAVLQFGNIALKRERNTDQATMPDNTAAQKLCRLLGLGVTDFSRALLTPRIKVGRDYVQKAQTKEQADFALEALAKATYERLFRWLVLRLNRALDRSPRQGASFLGILDIAGFEIFQLNSFEQLCINYTNEKLQQLFNHTMFVLEQEEYQREGIPWTFLDFGLDLQPCIDLIERPANPPGLLALLDEECWFPKATDKSFVEKVAQEQGGHPKFQRPRHLRDQADFSVLHYAGKVDYKANEWLMKNMDPLNDNVAALLHQSTDRLTAEIWKDVEGIVGLEQVSSLGDGPPGGRPRRGMFRTVGQLYKESLSRLMATLSNTNPSFVRCIVPNHEKRAGKLEPRLVLDQLRCNGVLEGIRICRQGFPNRILFQEFRQRYEILTPNAIPKGFMDGKQACEKMIQALELDPNLYRVGQSKIFFRAGVLAQLEEERDLKVTDIIVSFQAAARGYLARRAFQKRQQQQSALRVMQRNCAAYLKLRHWQWWRLFTKVKPLLQVTRQDEVLQARAQELQKVQELQQQSAREVGELQGRVAQLEEERARLAEQLRAEAELCAEAEETRGRLAARKQELELVVSELEARVGEEEECSRQMQTEKKRLQQHIQELEAHLEAEEGARQKLQLEKVTTEAKMKKFEEDLLLLEDQNSKLSKERKLLEDRLAEFSSQAAEEEEKVKSLNKLRLKYEATIADMEDRLRKEEKGRQELEKLKRRLDGESSELQEQMVEQQQRAEELRAQLGRKEEELQAALARAEDEGGARAQLLKSLREAQAALAEAQEDLESERVARTKAEKQRRDLGEELEALRGELEDTLDSTNAQQELRSKREQEVTELKKTLEEETRIHEAAVQELRQRHGQALGELAEQLEQARRGKGAWEKTRLALEAEVSELRAELSSLQTARQEGEQRRRRLELQLQEVQGRAGDGERARAEAAEKLQRAQAELENVSGALNEAESKTIRLSKELSSTEAQLHDAQELLQEETRAKLALGSRVRAMEAEAAGLREQLEEEAAARERAGRELQTAQAQLSEWRRRQEEEAGALEAGEEARRRAAREAEALTQRLAEKTETVDRLERGRRRLQQELDDATMDLEQQRQLVSTLEKKQRKFDQLLAEEKAAVLRAVEERERAEAEGREREARALSLTRALEEEQEAREELERQNRALRAELEALLSSKDDVGKSVHELERACRVAEQAANDLRAQVTELEDELTAAEDAKLRLEVTVQALKTQHERDLQGRDEAGEERRRQLAKQLRDAEVERDEERKQRTLAVAARKKLEGELEELKAQMASAGQGKEEAVKQLRKMQAQMKELWREVEETRTSREEIFSQNRESEKRLKGLEAEVLRLQEELAASDRARRQAQQDRDEMADEVANGNLSKAAILEEKRQLEGRLGQLEEELEEEQSNSELLNDRYRKLLLQVESLTTELSAERSFSAKAESGRQQLERQIQELRGRLGEEDAGARARHKMTIAALESKLAQAEEQLEQETRERILSGKLVRRAEKRLKEVVLQVEEERRVADQLRDQLEKGNLRVKQLKRQLEEAEEEASRAQAGRRRLQRELEDVTESAESMNREVTTLRNRLRRGPLTFTTRTVRQVFRLEEGVASDEEAEEAQPGSGPSPEPEGSPPAHPQ

>Hs_MYH11
MAQKGQLSDDEKFLFVDKNFINSPVAQADWAAKRLVWVPSEKQGFEAASIKEEKGDEVVVELVENGKKVTVGKDDIQKMNPPKFSKVEDMAELTCLNEASVLHNLRERYFSGLIYTYSGLFCVVVNPYKHLPIYSEKIVDMYKGKKRHEMPPHIYAIADTAYRSMLQDREDQSILCTGESGAGKTENTKKVIQYLAVVASSHKGKKDTSITGELEKQLLQANPILEAFGNAKTVKNDNSSRFGKFIRINFDVTGYIVGANIETYLLEKSRAIRQARDERTFHIFYYMIAGAKEKMRSDLLLEGFNNYTFLSNGFVPIPAAQDDEMFQETVEAMAIMGFSEEEQLSILKVVSSVLQLGNIVFKKERNTDQASMPDNTAAQKVCHLMGINVTDFTRSILTPRIKVGRDVVQKAQTKEQADFAVEALAKATYERLFRWILTRVNKALDKTHRQGASFLGILDIAGFEIFEVNSFEQLCINYTNEKLQQLFNHTMFILEQEEYQREGIEWNFIDFGLDLQPCIELIERPNNPPGVLALLDEECWFPKATDKSFVEKLCTEQGSHPKFQKPKQLKDKTEFSIIHYAGKVDYNASAWLTKNMDPLNDNVTSLLNASSDKFVADLWKDVDRIVGLDQMAKMTESSLPSASKTKKGMFRTVGQLYKEQLGKLMTTLRNTTPNFVRCIIPNHEKRSGKLDAFLVLEQLRCNGVLEGIRICRQGFPNRIVFQEFRQRYEILAANAIPKGFMDGKQACILMIKALELDPNLYRIGQSKIFFRTGVLAHLEEERDLKITDVIMAFQAMCRGYLARKAFAKRQQQLTAMKVIQRNCAAYLKLRNWQWWRLFTKVKPLLQVTRQEEEMQAKEDELQKTKERQQKAENELKELEQKHSQLTEEKNLLQEQLQAETELYAEAEEMRVRLAAKKQELEEILHEMEARLEEEEDRGQQLQAERKKMAQQMLDLEEQLEEEEAARQKLQLEKVTAEAKIKKLEDEILVMDDQNNKLSKERKLLEERISDLTTNLAEEEEKAKNLTKLKNKHESMISELEVRLKKEEKSRQELEKLKRKLEGDASDFHEQIADLQAQIAELKMQLAKKEEELQAALARLDDEIAQKNNALKKIRELEGHISDLQEDLDSERAARNKAEKQKRDLGEELEALKTELEDTLDSTATQQELRAKREQEVTVLKKALDEETRSHEAQVQEMRQKHAQAVEELTEQLEQFKRAKANLDKNKQTLEKENADLAGELRVLGQAKQEVEHKKKKLEAQVQELQSKCSDGERARAELNDKVHKLQNEVESVTGMLNEAEGKAIKLAKDVASLSSQLQDTQELLQEETRQKLNVSTKLRQLEEERNSLQDQLDEEMEAKQNLERHISTLNIQLSDSKKKLQDFASTVEALEEGKKRFQKEIENLTQQYEEKAAAYDKLEKTKNRLQQELDDLVVDLDNQRQLVSNLEKKQRKFDQLLAEEKNISSKYADERDRAEAEAREKETKALSLARALEEALEAKEELERTNKMLKAEMEDLVSSKDDVGKNVHELEKSKRALETQMEEMKTQLEELEDELQATEDAKLRLEVNMQALKGQFERDLQARDEQNEEKRRQLQRQLHEYETELEDERKQRALAAAAKKKLEGDLKDLELQADSAIKGREEAIKQLRKLQAQMKDFQRELEDARASRDEIFATAKENEKKAKSLEADLMQLQEDLAAAERARKQADLEKEELAEELASSLSGRNALQDEKRRLEARIAQLEEELEEEQGNMEAMSDRVRKATQQAEQLSNELATERSTAQKNESARQQLERQNKELRSKLHEMEGAVKSKFKSTIAALEAKIAQLEEQVEQEAREKQAATKSLKQKDKKLKEILLQVEDERKMAEQYKEQAEKGNARVKQLKRQLEEAEEESQRINANRRKLQRELDEATESNEAMGREVNALKSKLRRGNETSFVPSRRSGGRRVIENADGSEEETDTRDADFNGTKASE


>Hs_MYH8
MSASSDAEMAVFGEAAPYLRKSEKERIEAQNKPFDAKTSVFVAEPKESYVKSTIQSKEGGKVTVKTEGGATLTVREDQVFPMNPPKYDKIEDMAMMTHLHEPGVLYNLKERYAAWMIYTYSGLFCVTVNPYKWLPVYKPEVVAAYRGKKRQEAPPHIFSISDNAYQFMLTDRENQSILITGESGAGKTVNTKRVIQYFATIAVTGEKKKDESGKMQGTLEDQIISANPLLEAFGNAKTVRNDNSSRFGKFIRIHFGTTGKLASADIETYLLEKSRVTFQLKAERSYHIFYQITSNKKPDLIEMLLITTNPYDYAFVSQGEITVPSIDDQEELMATDSAIDILGFTPEEKVSIYKLTGAVMHYGNMKFKQKQREEQAEPDGTEVADKAAYLQSLNSADLLKALCYPRVKVGNEYVTKGQTVQQVYNAVGALAKAVYEKMFLWMVTRINQQLDTKQPRQYFIGVLDIAGFEIFDFNSLEQLCINFTNEKLQQFFNHHMFVLEQEEYKKEGIEWTFIDFGMDLAACIELIEKPLGIFSILEEECMFPKATDTSFKNKLYDQHLGKSANFQKPKVVKGKAEAHFSLIHYAGTVDYNITGWLDKNKDPLNDTVVGLYQKSAMKTLASLFSTYASAEADSSAKKGAKKKGSSFQTVSALFRENLNKLMTNLRSTHPHFVRCIIPNETKTPGAMEHELVLHQLRCNGVLEGIRICRKGFPSRILYGDFKQRYKVLNASAIPEGQFIDSKKASEKLLASIDIDHTQYKFGHTKVFFKAGLLGLLEEMRDEKLAQIITRTQAVCRGFLMRVEYQKMLQRREALFCIQYNVRAFMNVKHWPWMKLFFKIKLLKSAETEKEMATMKEEFQKTKDELAKSEAKRKELEEKMVTLLKEKNDLQLQVQSEADSLADAEERCEQLIKNKIQLEAKIKEVTERAEEEEEINAELTAKKRKLEDECSELKKDIDDLELTLAKVEKEKHATENKVKNLTEEMAGLDETIAKLSKEKKALQETHQQTLDDLQAEEDKVNILTKAKTKLEQQVDDLEGSLEQEKKLRMDLERAKRKLEGDLKLAQESTMDMENDKQQLDEKLEKKEFEISNLISKIEDEQAVEIQLQKKIKELQARIEELGEEIEAERASRAKAEKQRSDLSRELEEISERLEEAGGATSAQVELNKKREAEFQKLRRDLEEATLQHEAMVAALRKKHADSMAELGEQIDNLQRVKQKLEKEKSELKMETDDLSSNAEAISKAKGNLEKMCRSLEDQVSELKTKEEEQQRLINDLTAQRARLQTEAGEYSRQLDEKDALVSQLSRSKQASTQQIEELKHQLEEETKAKNALAHALQSSRHDCDLLREQYEEEQEGKAELQRALSKANSEVAQWRTKYETDAIQRTEELEEAKKKLAQRLQEAEEHVEAVNAKCASLEKTKQRLQNEVEDLMLDVERSNAACAALDKKQRNFDKVLSEWKQKYEETQAELEASQKESRSLSTELFKVKNVYEESLDQLETLRRENKNLQQEISDLTEQIAEGGKQIHELEKIKKQVEQEKCEIQAALEEAEASLEHEEGKILRIQLELNQVKSEVDRKIAEKDEEIDQLKRNHTRVVETMQSTLDAEIRSRNDALRVKKKMEGDLNEMEIQLNHANRLAAESLRNYRNTQGILKETQLHLDDALRGQEDLKEQLAIVERRANLLQAEIEELWATLEQTERSRKIAEQELLDASERVQLLHTQNTSLINTKKKLENDVSQLQSEVEEVIQESRNAEEKAKKAITDAAMMAEELKKEQDTSAHLERMKKNLEQTVKDLQHRLDEAEQLALKGGKKQIQKLEARVRELEGEVENEQKRNAEAVKGLRKHERRVKELTYQTEEDRKNVLRLQDLVDKLQAKVKSYKRQAEEAEEQSNANLSKFRKLQHELEEAEERADIAESQVNKLRVKSREVHTKISAE

>Hs_MYH2
MSSDSELAVFGEAAPFLRKSERERIEAQNRPFDAKTSVFVAEPKESFVKGTIQSREGGKVTVKTEGGATLTVKDDQVFPMNPPKYDKIEDMAMMTHLHEPAVLYNLKERYAAWMIYTYSGLFCVTVNPYKWLPVYKPEVVTAYRGKKRQEAPPHIFSISDNAYQFMLTDRENQSILITGESGAGKTVNTKRVIQYFATIAVTGEKKKEEITSGKIQGTLEDQIISANPLLEAFGNAKTVRNDNSSRFGKFIRIHFGTTGKLASADIETYLLEKSRVVFQLKAERSYHIFYQITSNKKPELIEMLLITTNPYDYPFVSQGEISVASIDDQEELMATDSAIDILGFTNEEKVSIYKLTGAVMHYGNLKFKQKQREEQAEPDGTEVADKAAYLQSLNSADLLKALCYPRVKVGNEYVTKGQTVEQVSNAVGALAKAVYEKMFLWMVARINQQLDTKQPRQYFIGVLDIAGFEIFDFNSLEQLCINFTNEKLQQFFNHHMFVLEQEEYKKEGIEWTFIDFGMDLAACIELIEKPMGIFSILEEECMFPKATDTSFKNKLYDQHLGKSANFQKPKVVKGKAEAHFALIHYAGVVDYNITGWLEKNKDPLNETVVGLYQKSAMKTLAQLFSGAQTAEGEGAGGGAKKGGKKKGSSFQTVSALFRENLNKLMTNLRSTHPHFVRCIIPNETKTPGAMEHELVLHQLRCNGVLEGIRICRKGFPSRILYADFKQRYKVLNASAIPEGQFIDSKKASEKLLASIDIDHTQYKFGHTKVFFKAGLLGLLEEMRDDKLAQLITRTQARCRGFLARVEYQRMVERREAIFCIQYNIRSFMNVKHWPWMKLFFKIKPLLKSAETEKEMATMKEEFQKIKDELAKSEAKRKELEEKMVTLLKEKNDLQLQVQAEAEGLADAEERCDQLIKTKIQLEAKIKEVTERAEDEEEINAELTAKKRKLEDECSELKKDIDDLELTLAKVEKEKHATENKVKNLTEEMAGLDETIAKLTKEKKALQEAHQQTLDDLQAEEDKVNTLTKAKIKLEQQVDDLEGSLEQEKKLRMDLERAKRKLEGDLKLAQESIMDIENEKQQLDEKLKKKEFEISNLQSKIEDEQALGIQLQKKIKELQARIEELEEEIEAERASRAKAEKQRSDLSRELEEISERLEEAGGATSAQIEMNKKREAEFQKMRRDLEEATLQHEATAATLRKKHADSVAELGEQIDNLQRVKQKLEKEKSEMKMEIDDLASNVETVSKAKGNLEKMCRTLEDQLSELKSKEEEQQRLINDLTAQRGRLQTESGEFSRQLDEKEALVSQLSRGKQAFTQQIEELKRQLEEEIKAKNALAHALQSSRHDCDLLREQYEEEQESKAELQRALSKANTEVAQWRTKYETDAIQRTEELEEAKKKLAQRLQAAEEHVEAVNAKCASLEKTKQRLQNEVEDLMLDVERTNAACAALDKKQRNFDKILAEWKQKCEETHAELEASQKEARSLGTELFKIKNAYEESLDQLETLKRENKNLQQEISDLTEQIAEGGKRIHELEKIKKQVEQEKCELQAALEEAEASLEHEEGKILRIQLELNQVKSEVDRKIAEKDEEIDQLKRNHIRIVESMQSTLDAEIRSRNDAIRLKKKMEGDLNEMEIQLNHANRMAAEALRNYRNTQGILKDTQIHLDDALRSQEDLKEQLAMVERRANLLQAEIEELRATLEQTERSRKIAEQELLDASERVQLLHTQNTSLINTKKKLETDISQMQGEMEDILQEARNAEEKAKKAITDAAMMAEELKKEQDTSAHLERMKKNMEQTVKDLQLRLDEAEQLALKGGKKQIQKLEARVRELEGEVESEQKRNAEAVKGLRKHERRVKELTYQTEEDRKNILRLQDLVDKLQAKVKSYKRQAEEAEEQSNTNLAKFRKLQHELEEAEERADIAESQVNKLRVKSREVHTKVISEE

>Hs_MYH4
MSSDSEMAIFGEAAPFLRKSEKERIEAQNKPFDAKTSVFVVDPKESYVKAIVQSREGGKVTAKTEAGATVTVKEDQVFSMNPPKYDKIEDMAMMTHLHEPAVLYNLKERYAAWMIYTYSGLFCVTVNPYKWLPVYNPEVVTAYRGKKRQEAPPHIFSISDNAYQFMLTDRENQSILITGESGAGKTVNTKRVIQYFATIAVTGEKKKEEPASGKMQGTLEDQIISANPLLEAFGNAKTVRNDNSSRFGKFIRIHFGATGKLASADIETYLLEKSRVTFQLKAERSYHIFYQILSNKKPELIEMLLITTNPYDFAFVSQGEITVPSIDDQEELMATDSAVDILGFTADEKVAIYKLTGAVMHYGNMKFKQKQREEQAEPDGTEVADKAAYLTSLNSADLLKSLCYPRVKVGNEFVTKGQTVQQVYNAVGALAKAIYEKMFLWMVTRINQQLDTKQPRQYFIGVLDIAGFEIFDFNSLEQLCINFTNEKLQQFFNHHMFVLEQEEYKKEGIEWEFIDFGMDLAACIELIEKPMGIFSILEEECMFPKATDTSFKNKLYEQHLGKSNNFQKPKPAKGKPEAHFSLVHYAGTVDYNIAGWLDKNKDPLNETVVGLYQKSAMKTLAFLFSGAQTAEAEGGGGKKGGKKKGSSFQTVSALFRENLNKLMTNLRSTHPHFVRCIIPNETKTPGAMEHELVLHQLRCNGVLEGIRICRKGFPSRILYADFKQRYKVLNASAIPEGQFIDSKKASEKLLGSIEIDHTQYKFGHTKVFFKAGLLGTLEEMRDEKLAQLITRTQAICRGFLMRVEFRKMMERRESIFCIQYNIRAFMNVKHWPWMKLYFKIKPLLKSAETEKEMANMKEEFEKTKEELAKTEAKRKELEEKMVTLMQEKNDLQLQVQAEADALADAEERCDQLIKTKIQLEAKIKEVTERAEDEEEINAELTAKKRKLEDECSELKKDIDDLELTLAKVEKEKHATENKVKNLTEEMAGLDETIAKLTKEKKALQEAHQQTLDDLQMEEDKVNTLTKAKTKLEQQVDDLEGSLEQEKKLCMDLERAKRKLEGDLKLAQESTMDTENDKQQLNEKLKKKEFEMSNLQGKIEDEQALAIQLQKKIKELQARIEELEEEIEAERASRAKAEKQRSDLSRELEEISERLEEAGGATSAQIEMNKKREAEFQKMRRDLEESTLQHEATAAALRKKHADSVAELGEQIDSLQRVKQKLEKEKSELKMEINDLASNMETVSKAKANFEKMCRTLEDQLSEIKTKEEEQQRLINELSAQKARLHTESGEFSRQLDEKDAMVSQLSRGKQAFTQQIEELKRQLEEETKAKSTLAHALQSARHDCDLLREQYEEEQEAKAELQRGMSKANSEVAQWRTKYETDAIQRTEELEEAKKKLAQRLQDAEEHVEAVNSKCASLEKTKQRLQNEVEDLMIDVERSNAACIALDKKQRNFDKVLAEWKQKYEETQAELEASQKESRSLSTELFKVKNAYEESLDHLETLKRENKNLQQEISDLTEQIAEGGKHIHELEKVKKQLDHEKSELQTSLEEAEASLEHEEGKILRIQLELNQVKSEIDRKIAEKDEELDQLKRNHLRVVESMQSTLDAEIRSRNDALRIKKKMEGDLNEMEIQLNHANRQAAEALRNLRNTQGILKDTQLHLDDAIRGQDDLKEQLAMVERRANLMQAEVEELRASLERTERGRKMAEQELLDASERVQLLHTQNTSLINTKKKLETDISQIQGEMEDIVQEARNAEEKAKKAITDAAMMAEELKKEQDTSAHLERMKKNMEQTVKDLQLRLDEAEQLALKGGKKQIQKLEARVRELESEVESEQKHNVEAVKGLRKHERRVKELTYQTEEDRKNILRLQDLVDKLQTKVKAYKRQAEEAEEQSNVNLAKFRKLQHELEEAKERADIAESQVNKLRVKSREVHTKVISEE

>Hs_MYH1
MSSDSEMAIFGEAAPFLRKSERERIEAQNKPFDAKTSVFVVDPKESFVKATVQSREGGKVTAKTEAGATVTVKDDQVFPMNPPKYDKIEDMAMMTHLHEPAVLYNLKERYAAWMIYTYSGLFCVTVNPYKWLPVYNAEVVTAYRGKKRQEAPPHIFSISDNAYQFMLTDRENQSILITGESGAGKTVNTKRVIQYFATIAVTGEKKKEEVTSGKMQGTLEDQIISANPLLEAFGNAKTVRNDNSSRFGKFIRIHFGTTGKLASADIETYLLEKSRVTFQLKAERSYHIFYQIMSNKKPDLIEMLLITTNPYDYAFVSQGEITVPSIDDQEELMATDSAIEILGFTSDERVSIYKLTGAVMHYGNMKFKQKQREEQAEPDGTEVADKAAYLQNLNSADLLKALCYPRVKVGNEYVTKGQTVQQVYNAVGALAKAVYDKMFLWMVTRINQQLDTKQPRQYFIGVLDIAGFEIFDFNSLEQLCINFTNEKLQQFFNHHMFVLEQEEYKKEGIEWTFIDFGMDLAACIELIEKPMGIFSILEEECMFPKATDTSFKNKLYEQHLGKSNNFQKPKPAKGKPEAHFSLIHYAGTVDYNIAGWLDKNKDPLNETVVGLYQKSAMKTLALLFVGATGAEAEAGGGKKGGKKKGSSFQTVSALFRENLNKLMTNLRSTHPHFVRCIIPNETKTPGAMEHELVLHQLRCNGVLEGIRICRKGFPSRILYADFKQRYKVLNASAIPEGQFIDSKKASEKLLGSIDIDHTQYKFGHTKVFFKAGLLGLLEEMRDEKLAQLITRTQAMCRGFLARVEYQKMVERRESIFCIQYNVRAFMNVKHWPWMKLYFKIKPLLKSAETEKEMANMKEEFEKTKEELAKTEAKRKELEEKMVTLMQEKNDLQLQVQAEADSLADAEERCDQLIKTKIQLEAKIKEVTERAEDEEEINAELTAKKRKLEDECSELKKDIDDLELTLAKVEKEKHATENKVKNLTEEMAGLDETIAKLTKEKKALQEAHQQTLDDLQAEEDKVNTLTKAKIKLEQQVDDLEGSLEQEKKIRMDLERAKRKLEGDLKLAQESTMDIENDKQQLDEKLKKKEFEMSGLQSKIEDEQALGMQLQKKIKELQARIEELEEEIEAERASRAKAEKQRSDLSRELEEISERLEEAGGATSAQIEMNKKREAEFQKMRRDLEEATLQHEATAATLRKKHADSVAELGEQIDNLQRVKQKLEKEKSEMKMEIDDLASNMETVSKAKGNLEKMCRALEDQLSEIKTKEEEQQRLINDLTAQRARLQTESGEYSRQLDEKDTLVSQLSRGKQAFTQQIEELKRQLEEEIKAKSALAHALQSSRHDCDLLREQYEEEQEAKAELQRAMSKANSEVAQWRTKYETDAIQRTEELEEAKKKLAQRLQDAEEHVEAVNAKCASLEKTKQRLQNEVEDLMIDVERTNAACAALDKKQRNFDKILAEWKQKCEETHAELEASQKESRSLSTELFKIKNAYEESLDQLETLKRENKNLQQEISDLTEQIAEGGKRIHELEKIKKQVEQEKSELQAALEEAEASLEHEEGKILRIQLELNQVKSEVDRKIAEKDEEIDQMKRNHIRIVESMQSTLDAEIRSRNDAIRLKKKMEGDLNEMEIQLNHANRMAAEALRNYRNTQAILKDTQLHLDDALRSQEDLKEQLAMVERRANLLQAEIEELRATLEQTERSRKIAEQELLDASERVQLLHTQNTSLINTKKKLETDISQIQGEMEDIIQEARNAEEKAKKAITDAAMMAEELKKEQDTSAHLERMKKNLEQTVKDLQHRLDEAEQLALKGGKKQIQKLEARVRELEGEVESEQKRNVEAVKGLRKHERKVKELTYQTEEDRKNILRLQDLVDKLQAKVKSYKRQAEEAEEQSNVNLSKFRRIQHELEEAEERADIAESQVNKLRVKSREVHTKIISEE

>Hs_MYH7B
MMDVSELGESARYLRQGYQEMTKVHTIPWDGKKRVWVPDEQDAYVEAEVKSEATGGRVTVETKDQKVLMVREAELQPMNPPRFDLLEDMAMMTHLNEASVLHNLRQRYARWMIYTYSGLFCVTINPYKWLPVYTASVVAAYKGKRRSDSPPHIYAVADNAYNDMLRNRDNQSMLITGESGAGKTVNTKRVIQYFAIVAALGDGPGKKAQFLATKTGGTLEDQIIEANPAMEAFGNAKTLRNDNSSRFGKFIRIHFGPSGKLASADIDSYLLEKSRVIFQLPGERSYHVYYQILSGRKPELQDMLLLSMNPYDYHFCSQGVITVDNMNDGEELIATDHAMDILGFSVDEKCACYKIVGALLHFGNMKFKQKQREEQAEADGTESADKAAYLMGVSSGDLLKGLLHPRVRVGNEYVTKGQSVEQVVFAVGALAKATYDRLFRWLVSRINQTLDTKLPRQFFIGVLDIAGFEIFEFNSFEQLCINFTNEKLQQFFNQHMFVLEQEEYKREGIDWVFIDFGLDLQPCIDLIEKPLGILSILEEECMFPKASDASFRAKLYDNHAGKSPNFQQPRPDKKRKYQAHFEVVHYAGVVPYSIVGWLEKNKDPLNETVVPIFQKSQNRLLATLYENYAGSCSTEPPKSGVKEKRKKAASFQTVSQLHKENLNKLMTNLRATQPHFVRCIVPNENKTPGVMDAFLVLHQLRCNGVLEGIRICRQGFPNRLLYTDFRQRYRILNPSAIPDDTFMDSRKATEKLLGSLDLDHTQYQFGHTKVFFKAGLLGVLEELRDQRLAKVLTLLQARSRGRLMRLEYQRLLGGRDALFTIQWNIRAFNAVKNWSWMKLFFKMKPLLRSAQAEEELAALRAELRGLRGALAAAEAKRQELEETHVSITQEKNDLALQLQAEQDNLADAEERCHLLIKSKVQLEGKVKELSERLEDEEEVNADLAARRRKLEDECTELKKDIDDLKLTLAKAEKEKQATENKVKNLTEEMAALDESVARLTKEKKALQEAHQQALGDLQAEEDRVSALTKAKLRLEQQVEDLECSLEQEKKLRMDTERAKRKLEGDLKLTQESVADAAQDKQQLEEKLKKKDSELSQLSLRVEDEQLLGAQMQKKIKELQARAEELEEELEAERAARARVEKQRAEAARELEELSERLEEAGGASAGQREGCRKREAELGRLRRELEEAALRHEATVAALRRKQAEGAAELGEQVDSLQRVRQKLEKEKSELRMEVDDLAANVETLTRAKASAEKLCRTYEDQLSEAKIKVEELQRQLADASTQRGRLQTESGELSRLLEEKECLISQLSRGKALAAQSLEELRRQLEEESKAKSALAHAVQALRHDCDLLREQHEEEAEAQAELQRLLSKANAEVAQWRSKYEADAIQRTEELEEAKKKLALRLQEAEEGVEAANAKCSSLEKAKLRLQTESEDVTLELERATSAAAALDKKQRHLERALEERRRQEEEMQRELEAAQRESRGLGTELFRLRHGHEEALEALETLKRENKNLQEEISDLTDQVSLSGKSIQELEKTKKALEGEKSEIQAALEEAEGALELEETKTLRIQLELSQVKAEVDRKLAEKDEECANLRRNHQRAVESLQASLDAETRARNEALRLKKKMEGDLNDLELQLGHATRQATEAQAATRLMQAQLKEEQAGRDEEQRLAAELHEQAQALERRASLLAAELEELRAALEQGERSRRLAEQELLEATERLNLLHSQNTGLLNQKKKLEADLAQLSGEVEEAAQERREAEEKAKKAITDAAMMAEELKKEQDTSAHLERMKKTLEQTVRELQARLEEAEQAALRGGKKQVQKLEAKVRELEAELDAEQKKHAEALKGVRKHERRVKELAYQAEEDRKNLARMQDLVDKLQSKVKSYKRQFEEAEQQANTNLAKYRKAQHELDDAEERADMAETQANKLRARTRDALGPKHKE

>Hs_MYH13
MSSDAEMAIFGEAAPYLRKPEKERIEAQNRPFDSKKACFVADNKEMYVKGMIQTRENDKVIVKTLDDRMLTLNNDQVFPMNPPKFDKIEDMAMMTHLHEPAVLYNLKERYAAWMIYTYSGLFCVTVNPYKWLPVYKPEVVAAYRGKKRQEAPPHIFSISDNAYQFMLTDRDNQSILITGESGAGKTVNTKRVIQYFATIAVTGDKKKETQPGKMQGTLEDQIIQANPLLEAFGNAKTVRNDNSSRFGKFIRIHFGATGKLASADIETYLLEKSRVTFQLSSERSYHIFYQIMSNKKPELIDLLLISTNPFDFPFVSQGEVTVASIDDSEELLATDNAIDILGFSSEEKVGIYKLTGAVMHYGNMKFKQKQREEQAEPDGTEVADKAGYLMGLNSAEMLKGLCCPRVKVGNEYVTKGQNVQQVTNSVGALAKAVYEKMFLWMVTRINQQLDTKQPRQYFIGVLDIAGFEIFDFNSLEQLCINFTNEKLQQFFNHHMFVLEQEEYKKEGIEWEFIDFGMDLAACIELIEKPMGIFSILEEECMFPKATDTSFKNKLYDQHLGKSNNFQKPKPAKGKAEAHFSLVHYAGTVDYNIAGWLDKNKDPLNETVVGLYQKSSLKLLSFLFSNYAGAETGDSGGSKKGGKKKGSSFQTVSAVFRENLNKLMTNLRSTHPHFVRCLIPNETKTPGVMDHYLVMHQLRCNGVLEGIRICRKGFPSRILYADFKQRYRILNASAIPEGQFIDSKNASEKLLNSIDVDREQFRFGNTKVFFKAGLLGLLEEMRDEKLVTLMTSTQAVCRGYLMRVEFKKMMERRDSIFCIQYNIRSFMNVKHWPWMNLFFKIKPLLKSAEAEKEMATMKEDFERTKEELARSEARRKELEEKMVSLLQEKNDLQLQVQSETENLMDAEERCEGLIKSKILLEAKVKELTERLEEEEEMNSELVAKKRNLEDKCSSLKRDIDDLELTLTKVEKEKHATENKVKNLSEEMTALEENISKLTKEKKSLQEAHQQTLDDLQVEEDKVNGLIKINAKLEQQTDDLEGSLEQEKKLRADLERAKRKLEGDLKMSQESIMDLENDKQQIEEKLKKKEFELSQLQAKIDDEQVHSLQFQKKIKELQARIEELEEEIEAEHTLRAKIEKQRSDLARELEEISERLEEASGATSAQIEMNKKREAEFQKMRRDLEEATLQHEATAATLRKKQADSVAELGEQIDNLQRVKQKLEKEKSELKMEIDDMASNIEALSKSKSNIERTCRTVEDQFSEIKAKDEQQTQLIHDLNMQKARLQTQNGELSHRVEEKESLISQLTKSKQALTQQLEELKRQMEEETKAKNAMAHALQSSRHDCDLLREQYEEEQEAKAELQRALSKANSEVAQWRTKYETDAIQRTEELEEAKKKLAQRLQEAEENTETANSKCASLEKTKQRLQGEVEDLMRDLERSHTACATLDKKQRNFDKVLAEWKQKLDESQAELEAAQKESRSLSTELFKMRNAYEEVVDQLETLRRENKNLQEEISDLTEQIAETGKNLQEAEKTKKLVEQEKSDLQVALEEVEGSLEHEESKILRVQLELSQVKSELDRKVIEKDEEIEQLKRNSQRAAEALQSVLDAEIRSRNDALRLKKKMEGDLNEMEIQLGHSNRQMAETQKHLRTVQGQLKDSQLHLDDALRSNEDLKEQLAIVERRNGLLLEELEEMKVALEQTERTRRLSEQELLDASDRVQLLHSQNTSLINTKKKLEADIAQCQAEVENSIQESRNAEEKAKKAITDAAMMAEELKKEQDTSAHLERMKKNLEQTVKDLQHRLDEAEQLALKGGKKQIQKLENRVRELENELDVEQKRGAEALKGAHKYERKVKEMTYQAEEDHKNILRLQDLVDKLQAKVKSYKRQAEEAEEQANTQLSRCRRVQHELEEAAERADIAESQVNKLRAKSRDVGSQKMEE

>Hs_Myh15 MVESCLLTFRAFFWWIALIKMDLSDLGEAAAFLRRSEAELLLLQATALDGKKKCWIPDGENAYIEAEVKGSEDDGTVIVETADGESLSIKEDKIQQMNPPEFEMIEDMAMLTHLNEASVLHTLKRRYGQWMIYTYSGLFCVTINPYKWLPVYQKEVMAAYKGKRRSEAPPHIFAVANNAFQDMLHNRENQSILFTGESGAGKTVNSKHIIQYFATIAAMIESRKKQGALEDQIMQANTILEAFGNAKTLRNDNSSRFGKFIRMHFGARGMLSSVDIDIYLLEKSRVIFQQAGERNYHIFYQILSGQKELHDLLLVSANPSDFHFCSCGAVTVESLDDAEELLATEQAMDILGFLPDEKYGCYKLTGAIMHFGNMKFKQKPREEQLEADGTENADKAAFLMGINSSELVKCLIHPRIKVGNEYVTRGQTIEQVTCAVGALSKSMYERMFKWLVARINRALDAKLSRQFFIGILDITGFEILEYNSLEQLCINFTNEKLQQFFNWHMFVLEQEEYKKESIEWVSIGFGLDLQACIDLIEKPMGILSILEEECMFPKATDLTFKTKLFDNHFGKSVHLQKPKPDKKKFEAHFELVHYAGVVPYNISGWLEKNKDLLNETVVAVFQKSSNRLLASLFENYMSTDSAIPFGEKKRKKGASFQTVASLHKENLNKLMTNLKSTAPHFVRCINPNVNKIPGILDPYLVLQQLRCNGVLEGTRICREGFPNRLQYADFKQRYCILNPRTFPKSKFVSSRKAAEELLGSLEIDHTQYRFGITKVFFKAGFLGQLEAIRDERLSKVFTLFQARAQGKLMRIKFQKILEERDALILIQWNIRAFMAVKNWPWMRLFFKIKPLVKSSEVGEEVAGLKEECAQLQKALEKSEFQREELKAKQVSLTQEKNDLILQLQAEQETLANVEEQCEWLIKSKIQLEARVKELSERVEEEEEINSELTARGRKLEDECFELKKEIDDLETMLVKSEKEKRTTEHKVKNLTEEVEFLNEDISKLNRAAKVVQEAHQQTLDDLHMEEEKLSSLSKANLKLEQQVDELEGALEQERKARMNCERELHKLEGNLKLNRESMENLESSQRHLAEELRKKELELSQMNSKVENEKGLVAQLQKTVKELQTQIKDLKEKLEAERTTRAKMERERADLTQDLADLNERLEEVGGSSLAQLEITKKQETKFQKLHRDMEEATLHFETTSASLKKRHADSLAELEGQVENLQQVKQKLEKDKSDLQLEVDDLLTRVEQMTRAKANAEKLCTLYEERLHEATAKLDKVTQLANDLAAQKTKLWSESGEFLRRLEEKEALINQLSREKSNFTRQIEDLRGQLEKETKSQSALAHALQKAQRDCDLLREQYEEEQEVKAELHRTLSKVNAEMVQWRMKYENNVIQRTEDLEDAKKELAIRLQEAAEAMGVANARNASLERARHQLQLELGDALSDLGKVRSAAARLDQKQLQSGKALADWKQKHEESQALLDASQKEVQALSTELLKLKNTYEESIVGQETLRRENKNLQEEISNLTNQVREGTKNLTEMEKVKKLIEEEKTEVQVTLEETEGALERNESKILHFQLELLEAKAELERKLSEKDEEIENFRRKQQCTIDSLQSSLDSEAKSRIEVTRLKKKMEEDLNEMELQLSCANRQVSEATKSLGQLQIQIKDLQMQLDDSTQLNSDLKEQVAVAERRNSLLQSELEDLRSLQEQTERGRRLSEEELLEATERINLFYTQNTSLLSQKKKLEADVARMQKEAEEVVQECQNAEEKAKKAAIEAANLSEELKKKQDTIAHLERTRENMEQTITDLQKRLAEAEQMALMGSRKQIQKLESRVRELEGELEGEIRRSAEAQRGARRLERCIKELTYQAEEDKKNLSRMQTQMDKLQLKVQNYKQQVEVAETQANQYLSKYKKQQHELNEVKERAEVAESQVNKLKIKAREFGKKVQEE


>Mm_MYH10
MAQRTGLEDPERYLFVDRAVIYNPATQADWTAKKLVWIPSERHGFEAASIKEERGDEVMVELAENGKKAMVNKDDIQKMNPPKFSKVEDMAELTCLNEASVLHNLKDRYYSGLIYTYSGLFCVVINPYKNLPIYSENIIEMYRGKKRHEMPPHIYAISESAYRCMLQDREDQSILCTGESGAGKTENTKKVIQYLAHVASSHKGRKDHNIPGELERQLLQANPILESFGNAKTVKNDNSSRFGKFIRINFDVTGYIVGANIETYLLEKSRAVRQAKDERTFHIFYQLLSGAGEHLKSDLLLEGFNNYRFLSNGYIPIPGQQDKDNFQETMEAMHIMGFSHEEILSMLKVVSSVLQFGNISFKKERNTDQASMPENTVAQKLCHLLGMNVMEFTRAILTPRIKVGRDYVQKAQTKEQADFAVEALAKATYERLFRWLVHRINKALDRTKRQGASFIGILDIAGFEIFELNSFEQLCINYTNEKLQQLFNHTMFILEQEEYQREGIEWNFIDFGLDLQPCIDLIERPANPPGVLALLDEECWFPKATDKTFVEKLVQEQGSHSKFQKPRQLKDKADFCIIHYAGKVDYKADEWLMKNMDPLNDNVATLLHQSSDRFVAELWKDVDRIVGLDQVTGMTETAFGSAYKTKKGMFRTVGQLYKESLTKLMATLRNTNPNFVRCIIPNHEKRAGKLDPHLVLDQLRCNGVLEGIRICRQGFPNRIVFQEFRQRYEILTPNAIPKGFMDGKQACERMIRALELDPNLYRIGQSKIFFRAGVLAHLEEERDLKITDIIIFFQAVCRGYLARKAFAKKQQQLSALKVLQRNCAAYLKLRHWQWWRVFTKVKPLLQVTRQEEELQAKDEELLKVKEKQTKVEGELEEMERKHQQLLEEKNILAEQLQAETELFAEAEEMRARLAAKKQELEEILHDLESRVEEEEERNQILQNEKKKMQAHIQDLEEQLDEEEGARQKLQLEKVTAEAKIKKMEEEVLLLEDQNSKFIKEKKLMEDRIAECSSQLAEEEEKAKNLAKIRNKQEVMISDLEERLKKEEKTRQELEKAKRKLDGETTDLQDQIAELQAQVDELKVQLTKKEEELQGALARGDDETLHKNNALKVARELQAQIAELQEDFESEKASRNKAEKQKRDLSEELEALKTELEDTLDTTAAQQELRTKREQEVAELKKALEDETKNHEAQIQDMRQRHATALEELSEQLEQAKRFKANLEKNKQGLETDNKELACEVKVLQQVKAESEHKRKKLDAQVQELHAKVSEGDRLRVELAEKANKLQNELDNVSTLLEEAEKKGIKFAKDAAGLESQLQDTQELLQEETRQKLNLSSRIRQLEEEKNSLQEQQEEEEEARKNLEKQVLALQSQLADTKKKVDDDLGTIESLEEAKKKLLKDVEALSQRLEEKVLAYDKLEKTKNRLQQELDDLTVDLDHQRQIVSNLEKKQKKFDQLLAEEKGISARYAEERDRAEAEAREKETKALSLARALEEALEAKEEFERQNKQLRADMEDLMSSKDDVGKNVHELEKSKRALEQQVEEMRTQLEELEDELQATEDAKLRLEVNMQAMKAQFERDLQTRDEQNEEKKRLLLKQVRELEAELEDERKQRALAVASKKKMEIDLKDLEAQIEAANKARDEVIKQLRKLQAQMKDYQRELEEARASRDEIFAQSKESEKKLKSLEAEILQLQEELASSERARRHAEQERDELADEIANSASGKSALLDEKRRLEARIAQLEEELEEEQSNMELLNDRFRKTTLQVDTLNTELAAERSAAQKSDNARQQLERQNKELKAKLQELEGAVKSKFKATISALEAKIGQLEEQLEQEAKERAAANKLVRRTEKKLKEIFMQVEDERRHADQYKEQMEKANARMKQLKRQLEEAEEEATRANASRRKLQRELDDATEANEGLSREVSTLKNRLRRGGPISFSSSRSGRRQLHIEGASLELSDDDTESKTSDVNDTQPPQSE

>Ms_MYH9
MAQQAADKYLYVDKNFINNPLAQADWAAKKLVWVPSSKNGFEPASLKEEVGEEAIVELVENGKKVKVNKDDIQKMNPPKFSKVEDMAELTCLNEASVLHNLKERYYSGLIYTYSGLFCVVINPYKNLPIYSEEIVEMYKGKKRHEMPPHIYAITDTAYRSMMQDREDQSILCTGESGAGKTENTKKVIQYLAHVASSHKSKKDQGELERQLLQANPILEAFGNAKTVKNDNSSRFGKFIRINFDVNGYIVGANIETYLLEKSRAIRQAKEERTFHIFYYLLSGAGEHLKTDLLLEPYNKYRFLSNGHVTIPGQQDKDMFQETMEAMRIMGIPEDEQMGLLRVISGVLQLGNIAFKKERNTDQASMPDNTAAQKVSHLLGINVTDFTRGILTPRIKVGRDYVQKAQTKEQADFAIEALAKATYERMFRWLVLRINKALDKTKRQGASFIGILDIAGFEIFDLNSFEQLCINYTNEKLQQLFNHTMFILEQEEYQREGIEWNFIDFGLDLQPCIDLIEKPAGPPGILALLDEECWFPKATDKSFVEKVVQEQGTHPKFQKPKQLKDKADFCIIHYAGKVDYKADEWLMKNMDPLNDNIATLLHQSSDKFVSELWKDVDRIIGLDQVAGMSETALPGAFKTRKGMFRTVGQLYKEQLAKLMATLRNTNPNFVRCIIPNHEKKAGKLDPHLVLDQLRCNGVLEGIRICRQGFPNRVVFQEFRQRYEILTPNSIPKGFMDGKQACVLMIKALELDSNLYRIGQSKVFFRAGVLAHLEEERDLKITDVIIGFQACCRGYLARKAFAKRQQQLTAMKVLQRNCAAYLRLRNWQWWRLFTKVKPLLNSIRHEDELLAKEAELTKVREKHLAAENRLTEMETMQSQLMAEKLQLQEQLQAETELCAEAEELRARLTAKKQELEEICHDLEARVEEEEERCQYLQAEKKKMQQNIQELEEQLEEEESARQKLQLEKVTTEAKLKKLEEDQIIMEDQNCKLAKEKKLLEDRVAEFTTNLMEEEEKSKSLAKLKNKHEAMITDLEERLRREEKQRQELEKTRRKLEGDSTDLSDQIAELQAQIAELKMQLAKKEEELQAALARVEEEAAQKNMALKKIRELETQISELQEDLESERASRNKAEKQKRDLGEELEALKTELEDTLDSTAAQQELRSKREQEVSILKKTLEDEAKTHEAQIQEMRQKHSQAVEELADQLEQTKRVKATLEKAKQTLENERGELANEVKALLQGKGDSEHKRKKVEAQLQELQVKFSEGERVRTELADKVTKLQVELDSVTGLLSQSDSKSSKLTKDFSALESQLQDTQELLQEENRQKLSLSTKLKQMEDEKNSFREQLEEEEEAKRNLEKQIATLHAQVTDMKKKMEDGVGCLETAEEAKRRLQKDLEGLSQRLEEKVAAYDKLEKTKTRLQQELDDLLVDLDHQRQSVSNLEKKQKKFDQLLAEEKTISAKYAEERDRAEAEAREKETKALSLARALEEAMEQKAELERLNKQFRTEMEDLMSSKDDVGKSVHELEKSKRALEQQVEEMKTQLEELEDELQATEDAKLRLEVNLQAMKAQFERDLQGRDEQSEEKKKQLVRQVREMEAELEDERKQRSMAMAARKKLEMDLKDLEAHIDTANKNREEAIKQLRKLQAQMKDCMRELDDTRASREEILAQAKENEKKLKSMEAEMIQLQEELAAAERAKRQAQQERDELADEIANSSGKGALALEEKRRLEARIAQLEEELEEEQGNTELINDRLKKANLQIDQINTDLNLERSHAQKNENARQQLERQNKELKAKLQEMESAVKSKYKASIAALEAKIAQLEEQLDNETKERQAASKQVRRTEKKLKDVLLQVEDERRNAEQFKDQADKASTRLKQLKRQLEEAEEEAQRANASRRKLQRELEDATETADAMNREVSSLKNKLRRGDLPFVVTRRIVRKGTGDCSDEEVDGKADGADAKAAE

>Mm_Myh6
MTDAQMADFGAAAQYLRKSEKERLEAQTRPFDIRTECFVPDDKEEYVKAKVVSREGGKVTAETENGKTVTIKEDQVMQQNPPKFDKIEDMAMLTFLHEPAVLYNLKERYAAWMIYTYSGLFCVTVNPYKWLPVYNAEVVAAYRGKKRSEAPPHIFSISDNAYQYMLTDRENQSILITGESGAGKTVNTKRVIQYFASIAAIGDRSKKENPNANKGTLEDQIIQANPALEAFGNAKTVRNDNSSRFGKFIRIHFGATGKLASADIETYLLEKSRVIFQLKAERNYHIFYQILSNKKPELLDMLLVTNNPYDYAFVSQGEVSVASIDDSEELLATDSAFDVLSFTAEEKAGVYKLTGAIMHYGNMKFKQKQREEQAEPDGTEDADKSAYLMGLNSADLLKGLCHPRVKVGNEYVTKGQSVQQVYYSIGALAKSVYEKMFNWMVTRINATLETKQPRQYFIGVLDIAGFEIFDFNSFEQLCINFTNEKLQQFFNHHMFVLEQEEYKKEGIEWEFIDFGMDLQACIDLIEKPMGIMSILEEECMFPKASDMTFKAKLYDNHLGKSNNFQKPRNVKGKQEAHFSLVHYAGTVDYNIMGWLEKNKDLNETVVGLYQKSSLKLMATLFSTYASADTGDSGKGKGGKKKGSSFQTVSALHRENLNKLMTNLKTTHPHFVRCIIPNERKAPGVMDNPLVMHQLRCNGVLEGIRICRKGFPNRILYGDFRQRYRILNPAAIPEGQFIDSRKGAEKLLGSLDIDHNQYKFGHTKVFFKAGLLGLLEEMRDERLSRIITRIQAQARGQLMRIEFKKIVERRDALLVIQWNIRAFMGVKNWPWMKLYFKIKPLLKSAETEKEMANMKEEFGRVKDALEKSEARRKELEEKMVSLLQEKNDLQLQVQAEQDNLNDAEERCDQLIKNKIQLEAKVKEMTERLEDEEEMNAELTAKKRKLEDECSELKKDIDDLELTLAKVEKEKHATENKVKNLTEEMAGLDEIIAKLTKEKKALQEAHQQALDDLQAEEDKVNTLTKSKVKLEQQVDDLEGSLEQEKKVRMDLERAKRKLEGDLKLTQESIMDLENDKLQLEEKLKKKEFDISQQNSKIEDEQALALQLQKKLKENQARIEELEEELEAERTARAKVEKLRSDLSRELEEISERLEEAGGATSVQIEMNKKREAEFQKMRRDLEEATLQHEATAAALRKKHADSVAELGEQIDNLQRVKQKLEKEKSEFKLELDDVTSNMEQIIKAKANLEKVSRTLEDQANEYRVKLEEAQRSLNDFTTQRAKLQTENGELARQLEEKEALISQLTRGKLSYTQQMEDLKRQLEEEGKAKNALAHALQSSRHDCDLLREQYEEEMEAKAELQRVLSKANSEVAQWRTKYETDAIQRTEELEEAKKKLAQRLQDAEEAVEAVNAKCSSLEKTKHRLQNEIEDLMVDVERSNAAAAALDKKQRNFDKILAEWKQKYEESQSELESSQKEARSLSTELFKLKNAYEESLEHLETFKRENKNLQEEISDLTEQLGEGGKNVHELEKIRKQLEVEKLELQSALEEAEASLEHEEGKILRAQLEFNQIKAEIERKLAEKDEEMEQAKRNHLRMVDSLQTSLDAETRSRNEALRVKKKMEGDLNEMEIQLSQANRIASEAQKHLKNSQAHLKDTQLQLDDAVHANDDLKENIAIVERRNNLLQAELEELRAVVEQTERSRKLAEQELIETSERVQLLHSQNTSLINQKKKMESDLTQLQTEVEEAVQECRNAEEKAKKAITDAAMMAEELKKEQDTSAHLERMKKNMEQTIKDLQHRLDEAEQIALKGGKKQLQKLEARVRELENELEAEQKRNAESVKGMRKSERRIKELTYQTEEDKKNLMRLQDLVDKLQLKVKAYKRQAEEAEEQANTNLSKFRKVQHELDEAEERADIAESQVNKLRAKSRDIGAKKMHDEE

>Mm_MYH14
MAAVTMSVSGRKVASRPGPVPEAAQSFLYAPRTPNVGGPGGPQVEWTARRMVWVPSELHGFEAAALRDEGEEEAEVELAESGRRLRLPRDQIQRMNPPKFSKAEDMAELTCLNEASVLHNLRERYYSGLIYTYSGLFCVVINPYKQLPIYTEAIVEMYRGKKRHEVPPHVYAVTEGAYRSMLQDREDQSILCTGESGAGKTENTKKVIQYLAHVASSPKGRKEPGVPASVSTMSYGELERQLLQANPILEAFGNAKTVKNDNSSRFGKFIRINFDIAGYIVGANIETYLLEKSRAIRQAKDECSFHIFYQLLGGAGEQLKADLLLEPCSHYRFLTNGPSSSPGQERELFQETLESLRVLGLLPEEITAMLRTVSAVLQFGNIVLKKERNTDQATMPDNTAAQKLCRLLGLGVTDFSRALLTPRIKVGRDYVQKAQTKEQADFALEALAKATYERLFRWLVLRLNRALDRSPRQGASFLGILDIAGFEIFQLNSFEQLCINYTNEKLQQLFNHTMFVLEQEEYQREGIPWTFLDFGLDLQPCIDLIERPANPPGLLALLDEECWFPKATDKSFVEKVAQEQGSHPKFQRPRNLRDQADFSVLHYAGKVDYKASEWLMKNMDPLNDNVAALLHQSTDRLTAEIWKDVEGIVGLEQVSSLGDGGGRPRRGMFRTVGQLYKESLSRLMATLSNTNPSFVRCIVPNHEKRAGKLEPRLVLDQLRCNGVLEGIRICRQGFPNRILFQEFRQRYEILTPNAIPKGFMDGKQACEKMIQALELDPNLYRVGQSKIFFRAGVLAQLEEERDLKVTDIIVSFQAAARGYLARRAFQRRQQQQSALRVMQRNCAAYLKLRNWQWWRLFIKVKPLLQVTRQDEVLQARAQELQKVQELQQQSAREVGELQGRVAQLEEERTRLAEQLRAEAELCSEAEETRARLAARKQELELVVTELEARVGEEEECSRQLQSEKKRLQQHIQELESHLEAEEGARQKLQLEKVTTEAKMKKFEEDLLLLEDQNSKLSKERRLLEERLAEFSSQAAEEEEKVKSLNKLRLKYEATISDMEDRLKKEEKGRQELEKLKRRLDGESSELQEQMVEQKQRAEELLAQLGRKEDELQAALLRAEEEGGARAQLLKSLREAQAGLAEAQEDLEAERVARAKAEKQRRDLGEELEALRGELEDTLDSTNAQQELRSKREQEVTELKKALEEESRAHEVSMQELRQRHSQALVEMAEQLEQARRGKGVWEKTRLSLEAEVSELKAELSSLQTSRQEGEQKRRRLESQLQEVQGRSSDSERARSEAAEKLQRAQAELESVSTALSEAESKAIRLGKELSSAESQLHDTQELLQEETRAKLALGSRVRALEAEAAGLREQMEEEVVARERAGRELQSTQAQLSEWRRRQEEEAAVLEAGEEARRRAAREAETLTQRLAEKTEAVERLERARRRLQQELDDATVDLGQQKQLLSTLEKKQRKFDQLLAEEKAAVLRAVEDRERIEAEGREREARALSLTRALEEEQEAREELERQNRALRAELEALLSSKDDVGKNVHELERARKAAEQAASDLRTQVTELEDELTAAEDAKLRLEVTVQALKAQHERDLQGRDDAGEERRRQLAKQLRDAEVERDEERKQRALAMAARKKLELELEELKAQTSAAGQGKEEAVKQLKKMQVQMKELWREVEETRSSRDEMFTLSRENEKKLKGLEAEVLRLQEELAASDRARRQAQQDRDEMAEEVASGNLSKAATLEEKRQLEGRLSQLEEELEEEQNNSELLKDHYRKLVLQVESLTTELSAERSFSAKAESGRQQLERQIQELRARLGEEDAGARARQKMLIAALESKLAQAEEQLEQESRERILSGKLVRRAEKRLKEVVLQVDEERRVADQVRDQLEKSNLRLKQLKRQLEEAEEEASRAQAGRRRLQRELEDVTESAESMNREVTTLRNRLRRGPLTFTTRTVRQVFRLEEGVASDEEEAEGAEPGSAPGQEPEAPPPATPQ

>Mm_Myh7
MADAEMAAFGAAAPFLRKSEKERLEAQTRPFDLKKDVFVPDDKEEFVKAKIVSREGGKVTAETENGKTVTVKEDQVMQQNPPKFDKIEDMAMLTFLHEPAVLYNLKERYASWMIYTYSGLFCVTVNPYKWLPVYNAEVVAAYRGKKRSEAPPHIFSISDNAYQYMLTDRENQSILITGESGAGKTVNTKRVIQYFAVIAAIGDRSKKDQTPGKGTLEDQIIQANPALEAFGNAKTVRNDNSSRFGKFIRIHFGATGKLASADIETYLLEKSRVIFQLKAERDYHIFYQILSNKKPELLDMLLITNNPYDYAFISQGETTVASIDDSEELMATDSAFDVLGFTPEEKNSIYKLTGAIMHFGNMKFKQKQREEQAEPDGTEEADKSAYLMGLNSADLLKGLCHPRVKVGNEYVTKGQNVQQVSYAIGALAKSVYEKMFNWMVTRINATLETKQPRQYFIGVLDIAGFEIFDFNSFEQLCINFTNEKLQQFFNHHMFVLEQEEYKKEGIEWTFIDFGMDLQACIDLIEKPMGIMSILEEECMFKATDMTFKAKLYDNHLGKSNNFQKPRNVKGKQEAHFSLVHYAGTVDYNILGWLQKNKDPLNETVVGLYQKSSLKLLSNLFANYAGADAPADKGKGKAKKGSSFQTVSALHRENLNKLMTNLRSTHPHFVRCIIPNETKSPGVMDNPLVMHQLRCNGVLEGIRICRKGFPNRILYGDFRQRYRILNPAAIPEGQFIDSRKGAEKLLGSLDIDHNQYKFGHTKVFFKAGLLGLLEEMRDERLSRIITRIQAQSRGVLSRMEFKKLLERRDSLLIIQWNIRAFMGVKNWPWMKLYFKIKPLLKSAETEKEMATMKEEFGRVKDALEKSEARRKELEEKMVSLLQEKNDLQLQVQAEQDNLADAEERCDQLIKNKIQLEAKVKEMTERLEDEEEMNAELTAKKRKLEDECSELKRDIDDLELTLAKVEKEKHATENKVKNLTEEMAGLDEIIVKLTKEKKALQEAHQQALDDLQAEEDKVNTLTKAKVKLEQQVDDLEGSLEQEKKVRMDLERAKRKLEGDLKLTQESIMDLENDKQQLDERLKKKDFELNALNARIEDEQALGSQLQKKLKELQARIEELEEELEAERTARAKVEKLRSDLSRELEEISERLEEAGGATSVQIEMNKKREAEFQKMRRDLEEATLQHEATAAALRKKHADSVAELGEQIDNLQRVKQKLEKEKSEFKLELDDVTSNMEQIIKAKANLEKMCRTLEDQMNEHRSKAEETQRSVNDLTSQRAKLQTENGELSRQLDEKEALISQLTRGKLTYTQQLEDLKRQLEEEVKAKNALAHALQSARHDCDLLREQYEEETEAKAELQRVLSKANSEVAQWRTKYETDAIQRTEELEEAKKKLAQRLQDAEEAVEAVNAKCSSLEKTKHRLQNEIEDLMVDVERSNAAAAALDKKQRNFDKILAEWKQKYEESQSELESSQKEARSLSTELFKLKNAYEESLEHLETFKRENKNLQEEISDLTEQLGSTGKSIHELEKIRKQLEAEKLELQSALEEAEASLEHEEGKILRAQLEFNQIKAEIERKLAEKDEEMEQAKRNHLRMVDSLQTSLDAETRSRNEALRVKKKMEGDLNEMEIQLSHANRMAAEAQKQVKSLQSLLKDTQIQLDDAVRANDDLKENIAIVERRNNLLQAELEELRAVVEQTERSRKLAEQELIETSERVQLLHSQNTSLINQKKKMDADLSQLQTEVEEAVQECRNAEEKAKKAITDAAMMAEELKKEQDTSAHLERMKKNMEQTIKDLQHRLDEAEQIALKGGKKQLQKLEARVRELENELEAEQKRNAESVKGMRKSERRIKELTYQTEEDRKNLLRLQDLVDKLQLKVKAYKRQAEEAEEQANTNLSKFRKVQHELDEAEERADIAESQVNKLRAKSRDIGAKGLNEE

>Mm_MYH11
MAQKGQLSDDEKFLFVDKNFMNSPMAQADWVAKKLVWVPSEKQGFEAASIKEEKGDEVVVELVENGKKVTVGKDDIQKMNPPKFSKVEDMAELTCLNEASVLHNLRERYFSGLIYTYSGLFCVVVNPYKYLPIYSEKIVDMYKGKKRHEMPPHIYAIADTAYRSMLQDREDQSILCTGESGAGKTENTQKVIQYLAVVASSHKGKKDSSITGELEKQLLQANPILEAFGNAKTVKNDNSSRFGKFIRINFDVTGYIVGANIETYLLEKSRAIRQARDERTFHIFYYLLAGAKEKMKSDLLLESFNSYTFLSNGFVPIPAAQDDEMFQETLEAMSIMGFNEEEQLAILKVVSSVLQLGNIVFKKERNTDQASMPDNTAAQKVCHLVGINVTDFTRAILTPRIKVGRDVVQKAQTKEQADFAIEALAKATYERLFRWILSRVNKALDKTHRQGASFLGILDIAGFEIFEVNSFEQLCINYTNEKLQQLFNHTMFILEQEEYQREGIEWNFIDFGLDLQPSIELIERPNNPPGVLALLDEECWFPKATDKSFVEKLCSEQGNHPKFQKPKQLKDKTEFSIIHYAGKVDYNASAWLTKNMDPLNDNVTSLLNASSDKFVADLWKDVDRIVGLDQMAKMTESSLPSASKTKKGMFRTVGQLYKEQLGKLMATLRNTTANFVRCIIPNHEKRSGKLDAFLVLEQLRCNGVLEGIRICRQGFPNRIVFQEFRQRYEILAANAIPKGFMDGKQACILMIKALELDPNLYRIGQSKIFFRTGVLAHLEEERDLKITDVIMAFQAMCRGYLARKAFTKRQQQLTAMKVIQRNCAAYLKLRNWQWWRLFTKVKPLLQVTRQEEEMQAKEEEMQKITERQQKAETELKELEQKHTQLAEEKTLLQEQLQAETELYAESEEMRVRLAAKKQELEEILHEMEARLEEEEDRRQQLQAERKKMAQQMLDLEEQLEEEEAARQKLQLEKVTAEAKIKKLEDDILVMDDQNSKLSKERKLLEERVSDLTTNLAEEEEKAKNLTKLKSKHESMISELEVRLKKEEKSRQELEKLKRKLEGDASDFHEQIADLQAQIAELKMQLAKKEEELQAALARLDEEIAQKNNALKKIRELEGHISDLQEDLDSERAARNKAEKQKRDLGEELEALKTELEDTLDSTATQQELRAKREQEVTVLKKALDEETRSHEAQVQEMRQKHTQAVEELTEQLEQFKRAKANLDKSKQTLEKENADLAGELRVLGQAKQEVEHKKKKLEVQLQDLQSKCSDGERARAELSDKVHKLQNEVESVTGMLNEAEGKAIKLAKDVASLGSQLQDTQELLQEETRQKLNVSTKLRQLEDERNSLQDQLDEEMEAKQNLERHVSTLNIQLSDSKKKLQDFASTIEVMEEGKKRLQKEMEGLSQQYEEKAAAYDKLEKTKNRLQQELDDLVVDLDNQRQLVSNLEKKQKKFDQLLAEEKNISSKYADERDRAEAEAREKETKALSLARALEEALEAKEELERTNKMLKAEMEDLVSSKDDVGKNVHELEKSKRALETQMEEMKTQLEESEDDVQATEDAKLRLEVNMQALKGQFERDLQARDEQNEEKRRQLQRQLHEYETELEDERKQRALAAAAKKKLEGDLKDLELQADSAIKGREEAIKQLRKLQAQMKDFQRELDDARASRDEIFATSKENEKKAKSLEADLMQLQEDLAAAERARKQADLEKEELAEELASSLSGRNTLQDEKRRLEARIAQLEEELEEEQGNMEAMSDRVRKATLQAEQLSNELATERSTAQKNESARQQLERQNKELRSKLQEVEGAVKAKLKSTVAALEAKIAQLEEQVEQEAREKQAATKSLKQKDKKLKEVLLQVEDERKMAEQYKEQAEKGNTKVKQLKRQLEEAEEESQCINANRRKLQRELDEATESNEAMGREVNALKSKLRRGNEASFVPSRRAGGRRVIENTDGSEEEMDARDSDFNGTKASE

>Mm_MYH3
MSSDTEMEVFGIAAPFLRKSEKERIEAQNQPFDAKTYCFVVDSKEEYVKGKIKSSQDGKVTVETEDSRTLVVKPEDVYAMNPPKFDKIEDMAMLTHLNEPAVLYNLKDRYTSWMIYTYSGLFCVTVNPYKWLPVYNPEVVDGYRGKKRQEAPPHIFSISDNAYQFMLTDRENQSILITGESGAGKTVNTKRVIQYFATIAATGDLAKKKDSKMKGTLEDQIISANPLLEAFGNAKTVRNDNSSRFGKFIRIHFGTTGKLASADIETYLLEKSRVTFQLKAERSYHIFYQILSNKKPELIELLLITTNPYDYPFISQGEILVASIDDAEELLATDSAIDILGFTPEEKSGLYKLTGAVMHYGNMKFKQKQREEQAEPDGTEVADKTAYLMGLNSSDLLKALCFPRVKVGNEYVTKGQTVDQVHHAVNALSKSVYEKLFLWMVTRINQQLDTKLPRQHFIGVLDIAGFEIFEYNSLEQLCINFTNEKLQQFFNHHMFVLEQEEYKKEGIEWTFIDFGMDLAACIELIEKPMGIFSILEEECMFPKATDTSFKNKLYDQHLGKSNNFQKPKVVKGKAEAHFSLVHYAGTVDYSVSGWLEKNKDLNETVVGLYQKSSNRLLAHLYATFATTDADGGKKKVAKKKGSSFQTVSALFRENLNKLMSNLRTTHPHFVRCIIPNETKTPGAMEHSLVLHQLRCNGVLEGIRICRKGFPNRILYGDFKQRYRVLNASAIPEGQFIDSKKACEKLLASIDIDHTQYKFGHTKVFFKAGLLGTLEEMRDERLAKLITRTQAVCRGFLMRVEFQKMMQRRESIFCIQYNIRAFMNVKHWPWMKLFFKIKPLLKSAETEKEMATMKEEFQKTKDELAKSEAKRKELEEKLVTLVQEKNDLQLQVQAESENLLDAEERCDQLIKAKFQLEAKIKEVTERAEDEEEINAELTAKKRKLEDECSELKKDIDDLELTLAKVEKEKHATENKVKNLTEELAGLDETIAKLTREKKALQEAHQQTLDDLQAEEDKVNSLSKLKSKLEQQVDDLESSLEQEKKLRVDLERNKRKLEGDLKLAQESILDLENDKQQLDERLKKKDFEYSQLQSKVEDEQTLSLQLQKKIKELQARIEELEEEIEAERATRAKTEKQRSDYARELEELSERLEEAGGVTSTQIELNKKREAEFLKLRRDLEEATLQHEATVATLRKKHADSAAELAEQIDNLQRVKQKLEKEKSEFKLEIDDLSSSVESVSKSKANLEKICRTLEDQLSEARGKNEEMQRSLSELTTQKSRLQTEAGELSRQLEEKESIVSQLSRSKQAFTQQIEELKRQLEEENKAKNALAHALQSSRHDCDLLREQYEEEQEGKAELQRALSKANSEVAQWRTKYETDAIQRTEELEEAKKKLAQRLQDSEEQVEAVNAKCASLEKTKQRLQGEVEDLMVDVERANSLAAALDKKQRNFDKVLAEWKTKCEESQAELEAALKESRSLSTELFKLKNAYEEALDQLETVKRENKNLEQEIADLTEQIAENGKSIHELEKSRKQMELEKADIQMALEEAEAALEHEEAKILRIQLELTQVKSEIDRKIAEKDEEIEQLKRNYQRTVETMQGALDAEVRSRNEAIRLKKKMEGDLNEIEIQLSHANRQAAETIKHLRSVQGQLKDTQLHLDDALRGQEDLKEQLAIVERRANLLQAEVEELRATLEQTERARKLAEQELLDSNERVQLLHTQNTSLIHTKKKLETDLTQLQSEVEDACRDARNAEEKAKKAITDAAMMAEELKKEQDTSAHLERMKKNLEQTVKDLQHRLDEAEQLALKGGKKQIQKLETRIRELEFELEGEQKRNTESVKGLRKYERRVKELTYQSEEDRKNVLRLQDLVDKLQVKVKSYKRQAEEADEQANAHLTKFRKAQHELEEAEERADIAESQVNKLRAKTRDFTSSRMVVHESEE

>Mm_MYH4
MSSDAEMAVFGEAAPYLRKSEKERIEAQNKPFDAKSSVFVVDAKESYVKATVQSREGGKVTAKTEGGATVTVKDDQVFSMNPPKYDKIEDMAMMTHLHEPAVLYNLKERYAAWMIYTYSGLFCVTVNPYKWLPVYNPEVVAAYRGKKRQEAPPHIFSISDNAYQFMLTDRENQSILITGESGAGKTVNTKRVIQYFATIAVTGDKKKEEATSGKMQGTLEDQIISANPLLEAFGNAKTVRNDNSSRFGKFIRIHFGATGKLASADIETYLLEKSRVTFQLKAERSYHIFYQIMSNKKPELIEMLLITTNPYDFAYVSQGEITVPSIDDQEELMATDTAVDILGFSADEKVAIYKLTGAVMHYGNMKFKQKQREEQAEPDGTEVADKAAYLTSLNSADLLKALCYPRVKVGNEYVTKGQTVQQVYNSVGALAKSMYEKMFLWMVTRINQQLDTKQPRQYFIGVLDIAGFEIFDFNTLEQLCINFTNEKLQQFFNHHMFVLEQEEYKKEGIDWEFIDFGMDLAACIELIEKPMGIFSILEEECMFPKATDTSFKNKLYEQHLGKSNNFQKPKPAKGKAEAHFSLVHYAGTVDYNIIGWLDKNKDPLNETVVGLYQKSGLKTLAFLFSGGQAAEAEGGGGKKGGKKKGSSFQTVSALFRENLNKLMTNLKSTHPHFVRCLIPNETKTPGAMEHELVLHQLRCNGVLEGIRICRKGFPSRILYADFKQRYKVLNASAIPEGQFIDSKKASEKLLGSIDIDHTQYKFGHTKVFFKAGLLGTLEEMRDEKLAQLITRTQAVCRGYLMRVEFKKMMERRESIFCIQYNVRAFMNVKHWPWMKLYFKIKPLLKSAETEKEMANMKEDFEKAKEDLAKSEAKRKELEEKMVALMQEKNDLQLQVQAEADGLADAEERCDQLIKTKIQLEAKIKELTERAEDEEEINAELTAKKRKLEDECSELKKDIDDLELTLAKVEKEKHATENKVKNLTEEMAGLDENIAKLTKEKKALQEAHQQTLDDLQAEEDKVNTLTKAKTKLEQQVDDLEGSLEQEKKLRMDLERAKRKLEGDLKLAQESTMDIENDKQQLDEKLKKKEFEMSNLQSKIEDEQALGMQLQKKIKELQARIEELEEEIEAERASRAKAEKQRSDLSRELEEISERLEEAGGATSAQIEMNKKREAEFQKMRRDLEEATLQHEATAAALRKKHADSVAELGEQIDNLQRVKQKLEKEKSELKMEIDDLASNMETVSKAKGNLEKMCRTLEDQLSEVKTKEEEQQRLINELSTQKARLHTESGEFSRQLDEKDAMVSQLSRGKQAFTQQIEELKRQLEEESKAKNALAHALQSARHDCDLLREQYEEEQEAKAELQRAMSKANSEVAQWRTKYETDAIQRTEELEEAKKKLAQRLQDAEEHVEAVNSKCASLEKTKQRLQNEVEDLMIDVERSNAACAALDKKQRNFDKVLAEWKQKYEETQAELEASQKESRSLSTELFKVKNAYEESLDQLETLKRENKNLQQEISDLTEQIAEGGKHIHELEKIKKQIDQEKSELQASLEEAEASLEHEEGKILRIQLELNQVKSEIDRKIAEKDEEIDQLKRNHLRVVESMQSTLDAEIRSRNDALRIKKKMEGDLNEMEIQLNHANRQAAEAIRNLRNTQGMLKDTQLHLDDALRGQDDLKEQLAMVERRANLMQAEIEELRASLEQTERSRRVAEQELLDASERVQLLHTQNTSLINTKKKLETDISQIQGEMEDIVQEARNAEEKAKKAITDAAMMAEELKKEQDTSAHLERMKKNMEQTVKDLQHRLDEAEQLALKGGKKQIQKLEARVRELENEVENEQKRNIEAVKGLRKHERRVKELTYQTEEDRKNVLRLQDLVDKLQTKVKAYKRQAEEAEEQSNVNLAKFRKIQHELEEAEERADIAESQVNKLRVKSREVHTKVISEE

>Mm_MYH1
MSSDAEMAVFGEAAPYLRKSEKERIEAQNKPFDAKSSVFVVDAKESFVKATVQSREGGKVTAKTEGGTTVTVKDDQVYPMNPPKYDKIEDMAMMTHLHEPAVLYNLKERYAAWMIYTYSGLFCVTVNPYKWLPVYNAEVVAAYRGKKRQEAPPHIFSISDNAYQFMLTDRENQSILITGESGAGKTVNTKRVIQYFATIAVTGEKKKEEATSGKMQGTLEDQIISANPLLEAFGNAKTVRNDNSSRFGKFIRIHFGTTGKLASADIETYLLEKSRVTFQLKAERSYHIFYQIMSNKKPDLIEMLLITTNPYDYAFVSQGEITVPSIDDQEELMATDSAIDILGFTSDERVSIYKLTGAVMHYGNMKFKQKQREEQAEPDGTEVADKAAYLQNLNSADLLKALCYPRVKVGNEYVTKGQTVQQVYNSVGALAKAVYEKMFLWMVTRINQQLDTKQPRQYFIGVLDIAGFEIFDFNSLEQLCINFTNEKLQQFFNHHMFVLEQEEYKKEGIEWEFIDFGMDLAACIELIEKPMGIFSILEEECMFPKATDTSFKNKLYEQHLGKSNNFQKPKPAKGKVEAHFSLVHYAGTVDYNIAGWLDKNKDPLNETVVGLYQKSSMKTLAYLFSGAAAAAEAESGGGGGKKGAKKKGSSFQTVSALFRENLNKLMTNLRSTHPHFVRCIIPNETKTPGAMEHELVLHQLRCNGVLEGIRICRKGFPSRILYADFKQRYKVLNASAIPEGQFIDSKKASEKLLGSIDIDHTQYKFGHTKVFFKAGLLGLLEEMRDDKLAQLITRTQAMCRGYLARVEYQKMVERRESIFCIQYNVRAFMNVKHWPWMKLYFKIKPLLKSAETEKEMANMKEEFEKAKENLAKAEAKRKELEEKMVALMQEKNDLQLQVQSEADSLADAEERCDQLIKTKIQLEAKIKEVTERAEDEEEINAELTAKKRKLEDECSELKKDIDDLELTLAKVEKEKHATENKVKNLTEEMAGLDETIAKLTKEKKALQEAHQQTLDDLQAEEDKVNTLTKAKIKLEQQVDDLEGSLEQEKKIRMDLERAKRKLEGDLKLAQESTMDVENDKQQLDEKLKKKEFEMSNLQSKIEDEQALGMQLQKKIKELQARIEELEEEIEAERASRAKAEKQRSDLSRELEEISERLEEAGGATSAQIEMNKKREAEFQKMRRDLEEATLQHEATAATLRKKHADSVAELGEQIDNLQRVKQKLEKEKSEMKMEIDDLASNMEVISKSKGNLEKMCRTLEDQVSELKTKEEEQQRLINELTAQRGRLQTESGEYSRQLDEKDSLVSQLSRGKQAFTQQIEELKRQLEEEIKAKSALAHALQSSRHDCDLLREQYEEEQEAKAELQRAMSKANSEVAQWRTKYETDAIQRTEELEEAKKKLAQRLQDAEEHVEAVNAKCASLEKTKQRLQNEVEDLMIDVERTNAACAALDKKQRNFDKILAEWKQKYEETHAELEASQKESRSLSTELFKIKNAYEESLDHLETLKRENKNLQQEISDLTEQIAEGGKRIHELEKIKKQIEQEKSELQAALEEAEASLEHEEGKILRIQLELNQVKSEIDRKIAEKDEEIDQLKRNHIRVVESMQSTLDAEIRSRNDAIRLKKKMEGDLNEMEIQLNHSNRMAAEALRNYRNTQGILKDTQLHLDDALRGQEDLKEQLAMVERRANLLQAEIEELRATLEQTERSRKIAEQELLDASERVQLLHTQNTSLINTKKKLETDISQIQGEMEDIVQEARNAEEKAKKAITDAAMMAEELKKEQDTSAHLERMKKNLEQTVKDLQHRLDEAEQLALKGGKKQIQKLEARVRELEGEVENEQKRNVEAIKGLRKHERRVKELTYQTEEDRKNVLRLQDLVDKLQSKVKAYKRQAEEAEEQSNVNLAKFRKIQHELEEAEERADIAESQVNKLRVKSREVHTKIISEE

>Mm_MYH8
MSAGSDAEMAIFGEAAPYLRKSEKERIEAQNKPFDAKTSVFVAEPKESYVKSVIQSKDGGKVTVKTESGATLTVKEDQVFPMNPPKYDKIEDMAMMTHLHEPGVLYNLKERYAAWMIYTYSGLFCVTVNPYKWLPVYNPEVVAAYRGKKRQEAPPHIFSISDNAYQFMLTDRENQSILITGESGAGKTVNTKRVIQYFATIAVTGDKKKEESGKMQGTLEDQIISANPLLEAFGNAKTVRNDNSSRFGKFIRIHFGTTGKLASADIETYLLEKSRVTFQLKAERSYHIFYQITSNKKPELIEMLLITTNPYDYAFVSQGEITVPSIDDQEELMATDSAIDILGFSPEEKVSIYKLTGAVMHYGNMKFKQKQREEQAEPDGTEVADKAAYLQCLNSADLLKALCYPRVKVGNEYVTKGQTVQQVYNAVGALAKAVYEKMFLWMVTRINQQLDTKQPRQYFIGVLDIAGFEIFDFNSLEQLCINFTNEKLQQFFNHHMFVLEQEEYKKEGIEWTFIDFGMDLAACIELIEKPLGIFSILEEECMFPKATDTSFKNKLYDQHLGKSNNFQKPKPTKGKAEAHFSLVHYAGTVDYNITGWLDKNKDPLNDTVVGLYQKSAMKTLASLFSTYASAEADGGAKKGAKKKGSSFQTVSALFRENLNKLMTNLRSTHPHFVRCIIPNETKTPGAMEHELVLHQLRCNGVLEGIRICRKGFPSRILYGDFKQRYKVLNASAIPEGQFIDSKKASEKLLGSIDIDHTQYKFGHTKVFFKAGLLGLLEEMRDEKLAQIITRTQAVCRGYLMRVEYQKMLLRRESIFCIQYNVRAFMNVKHWPWMKLFFKIKLLKSAETEKEMATMKEEFQKTKDELAKSEAKRKELEEKMVTLLKEKNDLQLQVQSEADSLADAEERCEQLIKNKIQLEAKIKEVTERAEDEEEINAELTAKKRKLEDECSELKKDIDDLELTLAKVEKEKHATENKVKNLTEEMAGLDENIAKLTKEKKALQEAHQQTLDDLQAEEDKVNTLTKAKTKLEQQVDDLEGSLEQEKKLRMDLERAKRKLEGDLKLAQESTMDIENDKQQLDEKLKKKEFEISNLISKIEDEQAVEIQLQKKIKELQARIEELEEEIEAERASRAKAEKQRSDLSRELEEISERLEEAGGATSAQVEMNKKRETEFQKLRRDLEEATLQHEATAAALRKKHADSVAELGEQIDNLQRVKQKLEKEKSELKMEIDDLSSNAEAIAKAKGNLEKMCRTLEDQVSELKSKEEEQQRLINELTAQRARLQTEAGEYSRQLDEKDALVSQLSRSKQASTQQIEELKRQLEEETKAKNALAHALQSSRHDCDLLREQYEEEQEGKAELQRALSKANSEVAQWRTKYETDAIQRTEELEEAKKKLAQRLQAAEEHVEAVNAKCASLEKTKQRLQNEVEDLMIDVERTNAACAALDKKQRNFDKVLSEWRQKYEETQAELESCQKESRTLSTELFKVKNAYEESLDHLETLRRENKNLQQEISDLTEQIAEGGKHIHELEKIKKQVEQEKCEIQAALEEAEASLEHEEGKILRIQLELNQVKSEIDRKIAEKDEEIDQLKRNHVRVVETMQSTLDAEIRSRNDALRVKKKMEGDLNEMEIQLNHANRLAAESLRNYRNTQGILKDTQLHLDDALRGQEDLKEQLAIVERRANLLQAEIEELRATLEQTERSRKIAEQELLDASERVQLLHTQNTSLINTKKKLENDVSQLQSEVEEVIQESRNAEEKAKKAITDAAMMAEELKKEQDTSAHLERMKKNMEQTVKDLQHRLDEAEQLALKGGKKQIQKLEARVRELEGEVENEQKRNAEAVKGLRKHERRVKELTYQTEEDRKNVLRLQDLVDKLQAKVKSYKRQAEEAEEQSNANLAKFRKLQHELEEAEERADIAESQVNKLRVKSREVHTKISAE

>Mm_Myh7B
MMDMSELGESACYLRQGYQEMMKVHTVPWDGKKRVWVPDEQDAYVEAEVKTEATGGKVTVETKDQKVLTVRETEMQPMNPPRFDLLEDMAMMTHLNEAAVLHNLRQRYARWMIYTYSGLFCVTINPYKWLPVYTAAVVAAYKGKRRSEAPPHIYAVADNAYNDMLRNRENQSMLITGESGAGKTVNTKRVIQYFAIVAALGDGPGKKAQFLATKTGGTLEDQIIEANPAMEAFGNAKTLRNDNSSRFGKFIRIHFGPTGKLASADIDSYLLEKSRVIFQLPGERGYHVYYQILSGKKPELQDMLLLSMNPYDYHFCSQGVTTVDNMDDGEELIATDHAMDILGFSVDEKCACYKIVGALLHFGNMKFKQKQREEQAEADGTESADKAAYLMGVSSGDLLKGLLHPRVRVGNEYVTKGQSVEQVVFAVGALAKATYDRLFRWLVSRINQTLDTKLPRQFFIGVLDIAGFEIFEFNSFEQLCINFTNEKLQQFFNQHMFVLEQEEYKREGIDWVFIDFGLDLQPCIDLIEKPLGILSILEEECMFPKASDASFRAKLYDNHSGKSPNFQQPRPDKKRKYQAHFEVVHYAGVVPYSIVGWLEKNKDPLNETVVPIFQKSQNRLLATLYENYAGSCSTEPPKSGVKEKRKKAASFQTVSQLHKENLNKLMTNLRATQPHFVRCIVPNENKTPGVMDSFLVLHQLRCNGVLEGIRICRQGFPNRLLYADFRQRYRILNPSAIPDDTFVDSRKATEKLLGSLDIDHTQYQFGHTKVFFKAGLLGILEELRDQRLAKVLTLLQARSRGRLMRLEYQRMLGGRDALFTIQWNIRAFNAVKNWSWMKLFFKMKPLLRSAQAEEELAALRAELRGLRGALATAEAKRQELEETQVSVTQEKNDLALQLQAEQDNLADAEERCHLLIKSKVQLEAKVKELSERLEDEEEVNADLAARRRKLEDECTELKKDIDDLELTLAKAEKEKQATENKVKNLTEEMAALDEAVVRLTKEKKALQEAHQQALGDLQAEEDRVSALAKAKIRLEQQVEDLECSLEQEKKLRMDTERAKRKLEGDLKLTQETVTDTTQDKQQLEEKLKKKDSELSQLNLRVEDEQLVGVQLQKKIKELQARAEELEEELEAERAARARVEKQRAEAARELEELSERLEEAGGASAGQREGCRKREAELGRLRRELEEAVLRHEATVAALRRKQADSAAELSEQVDSLQRIRQKLEKEKSELRMEVDDLGASVETLARGKASAEKLCRTYEDQLSEAKIKVEELQRQLADASTQRGRLQTENGELGRLLEEKESMISQLSRGKTSAAQSLEELRRQLEEESKAKGALAHAVQALRHDCDLLREQHEEESEAQAELQRLLSKANAEVAQWRSKYEADAIQRTEELEEAKKKLALRLQEAEEGVEAANAKCSSLEKAKLRLQTESEDVTLELERATSAAAALDKKQRHLERALEERRRQEEEMQRELEAAQREARGLGTELFRLRHSHEEALEALETLKRENKNLQEEISDLTDQVSLSGKSIQELEKAKKALEGEKSELQAALEEAEGALELEETKTLRIQLELSQVKAEVDRKLAEKDEECTNLRRNHQRAVESLQASLDAETRARNEALRLKKKMEGDLNDLELQLGHATRQAMEAQAATRLLQAQLKEEQAGRDEEQRLAAELREQGQALERRAALLAAELEELRAALEQGERSRRLAEQELLEATERLNLLHSQNTGLLNQKKKLEVDLAQLSGEVEEAAQERREAEEKAKKAITDAAMMAEELKKEQDTSAHLERMKKTLEQTVRELQARLEEAEQAALRGGKKQVQKLEAKVRELEAELDAEQKKHAEALKGVRKHERRVKELVYQTEEDRKNLARMQDLVDKLQSKVKSYKRQFEEAEQQASTNLAKYRKAQHELDDAEERADMAETQANKLRARSRDALGPKHKE


>Ci_MYHE.v1
MSFDYEACQDAAQYLRLSREKIIENQTQKPDGKKYVWFPTKAEAYVKGELIKTEKGKCTIKSIDEGKEMTVKEDDLQEMNPPRYEKCEDMAGMTFLNEASVLNNLRSRYESFMIYTYSGLFCVTVNPYKMLPVYAPYVIAAYKGKRRTEMPPHLYSIADNAYTEMLMNRENQSMLITGESGAGKTVNTKKVIQYFALVAAYGAAQDDGKGTLEDQIVQCNPAMEAFGNAKTVRNDNSSRFGKFIRIHFGSTGLLASGDIEHYLLEKSRVIYQQEGERNYHIFYQIISGSKPELIDQLLVTKDPYDYKSISQGVVTVDNMDDGAELLLTDDAFRVLGFNQEEISGIYRIMAGIMHQQNMKFKNKQREEQAEPDGTEDADKVAYLFGLNSADFIKYICHPRVKVGNEYVTKGQSCNQVSYGMGALSKGLFGRHFDWLVKLINQTLSTKLPRSFFIGVLDIAGFEIFDSNSFEQLCINFTNEKLQQFFNHHMFVLEQEEYKKEGIDWVFIDFGMDLAACIELIEKPLGIMSILEEECMFPKASDDTFKDKLYQNHLGKSKAFGKPVKKTKYEAHFELYHYAGTVAYNICGWLEKNKDPLNNSVVDLYKKASLKLMQTIWEGYVSPEEAASGGGKGGKRKKGGSFMTVSSLHRQSLNSLMTNLRSTAPHFVRCLIPNERKCPGEMDSHLVLHQLRCNGVLEGIRICRKGFPNRVPYGDFKQRYRILNPNAAPEGQFMDSKKTSEKLLGSIDIDHESYKLGHTKVFFRAGMIGKLEEMRDNKLSSIFKLIQGRMRGVLMRREYQKMIERRQACRIIQSNLRAFFGMANCEWMKLMFKIKPLLKTAESAKELEEMEKEFAETKVNLEKETKRRKELEEMQVSFIQEKNDLVMQLQAQQDQIDDGEDRCDQLIKTKSEMKMEIDDLASNVESVTKAKLNYEKMARNLEEQLNETKMKNDNFTKEVNELNAAKARLSSENGEFGRQLDEREHLMAQLTRSKNSSSQQIDELKRVVEEETKAKAALAHAVQASRHDNDLLREQYEEEQEAKAELQRALSKANAEVAQWRNKYETDAIQRTEELEEAKKKLALRLQEAEEQVEAMQAKAASLDKTKNRLQGEVEDLTIDLERSNAGAAALDKKQRNFDKVLSEHKQKAEEVQVELEQSQKEARSLSTELFKMKNAYEEALDGLETVKRENKNLQEEIADLTDQLGEGGKSIHELEKAKRTLEHERNEIQAALEEAEGAMEGEESKVLRLQVEVAQMKQEFERKIAEKEEEVDTQRRNQQRSIESMQTTLDSESKARQEAVRIKKKMEGDLNDLEIQLGHANRQASEAQKQAKSIQSHVKDLEMQVDEAQRHAEDLQEQSAVIERRGNLLTAEIEELRSALEQAERGRKLAETELLDSSERSNMLHTQNTALINQKRKLEGELQTMQGEVEEAVQEQRNAEEKAKKSIVDAATMAEELKKEQDLSSHLERMKKNMEQTVKDLQQRLDEAENIALKGGKKQVQKLEARIRELENEVDSEQRRNSDSVKNQRKFERRLKEVTYQGEEDKKNLTRIQDLVDKLQIKVKTYKRQAEEAEEQANTNLSKYRKLQHELDDAEERAEMAESQLNKMRSKARDMKSDK

>Ci_MYHE.v2
MSFDYEACQDAAQYLRLSREKIIENQTQKPDGKKYVWFPDKAEAYVKGELIKTEKGKCTIKSIAEGKEMTVKEDDLQEMNPPRYEKCEDMAGMTFLNEASVLNNLRSRYESFMIYTYSGLFCVTVNPYKMLPVYAPYVIAAYKGKRRTEMPPHLYSIADNAYTEMLMNRENQSMLITGESGAGKTVNTKKVIQYFALVAAYGSAQDDGKGTLEDQIVQCNPAMEAFGNAKTVRNDNSSRFGKFIRIHFGSTGLLASGDIEHYLLEKSRVIYQQEGERNYHIFYQIISGSKPELIDQLLVTKDPYDYKSISQGVVSVDNLDDGAELLLTDDAFRVLGFNQEEITGIYRIMAGIMHQQNMKFKNKQREEQAEPDGTEDADKVAYLFGLNSADFIKYICHPRVKVGNEYVTKGQSCNQVSYGMGALSKGLFGRHFDWLVKLINQTLSTKLPRSFFIGVLDIAGFEIFDSNSFEQLCINFTNEKLQQFFNHHMFVLEQEEYKKEGIDWVFIDFGMDLAACIELIEKPLGIMSILEEECMFPKASDDTFKDKLYQNHLGKSKAFGKPVKKTKYEAHFELYHYAGTVAYNICGWLEKNKDPLNNSVVDLYKKASLKLMQTIWEGYVSPEEAASGGGKGGKRKKGGSFMTVSSLHRQSLNSLMTNLRSTAPHFVRCLIPNERKCPGEMESHLVLHQLRCNGVLEGIRICRKGFPNRVPYGDFKQRYRILNPNAAPEGQFMDSKKASEKLLGSIDINHESYKLGHTKVFFRAGMIGRLEEMRDNKLASIFKLLQARMRGMLMRKEYQKMIERRQACRIIQSNLRAFFGMANCEWMKLMFKIKPLLKTAESAKELEEMEKEFAETKVNLEKETKRRKELEEMQVSFVQEKNDLVMQLQAQQDQIDDGEDRCDQLIKTKVELDGKIKELTERLEDEEELNNELVSKKRKLEDECSELKKDIDDLEITLAKVEKEKHATENKLKNLQEELATQDEQIAKLQKEKKALQEAHQQTLDDLQSEEDKVNSLTKQKAKLEQQVDDLEASLEQEKKLRMELERTKRKLEGDLRLTQETVMDLENDKQRLEEKLKKQEFEYSQLATKLEDEQALVSQLQKKIKELQARIEELEEELEAERAARAKVEKQRADLSRELEELSERLEEAGGATAAQIELNKRREAEFAKLRRELEESNLGHEATVSTLRKKNADTSSEMSEQIDNLQRVKQKLEKEKSEMKMEIDDLASNVESVTKAKLNYEKMARNLEEQLNETKMKNDNFTKEVNELNAAKARLSSENGEFGRQLEEREHLMAQLTRSKNSSSQQIDELKRVVEEETKAKAALAHAVQASRHDNDLLREQYEEEQEAKAELQRALSKANAEVAQWRNKYETDAIQRTEELEEAKKKLAIRLQEAEEQVEAMQAKASSLDKTKNRLQSELEDLTIDLEKSNSAAAALDKKQRNFDKILAEHKQKAEEIQVELEQSQKEARSLSTELFKMKNAYEESLDALETVKRENKNLQEEIADLTDQLGEGGKSIHELEKAKRTLEHERNEMQSALEEAEGAIEGEESKVLRLQVELAQIKQEFERRLAEKEEEVDNQRRNQQRSIESMQTTLDSESKARQEAVRIKKKMEGDLNDLEIQLGHANRQASEAQKQAKSIQSHVKDLEMQVDEAQRHGEDLQEQSAVIERRGNLLTAEIEELRSALEQAERGRKLAETELLESSERSNLLHTQNTALINQKRKLEGELQTMQGEVEESVQEQRNAEDKAKKAIVDAATMAEELKKEQDLSSHLERMKKNMEQTVKDLQQRLDEAENIALKGGKKQVQKLETRIRELENELDSEQRRNGDSVKGQRKLERRLKEVSYQGEEDKKNLTRIQDLVDKLQIKVKTYKRQAEEAEEQANTNLSKYRKLQHELDDAEERAEMAESQLNKMRSKARDSKGAN


>Ci_MYHE.v3
MSFDYEACQDAAQYLRLSREKIIENQTQKPDGKKYVWFPDKAEAYVKGELIKTEKGKCTIKSIAEGKEMTVKEDDLQEMNPPRYEKCEDMAGMTFLNEASVLNNLRSRYESFMIYTYSGLFCVTVNPYKMLPVYAPYVIAAYKGKRRTEMPPHLYSIADNAYTEMLMRIIKYNTNVYYRGESGAGKTVNTKKVIQYFALVAAYGASQDDGKGTLEDQIVQCNPAMEAFGNAKTVRNDNSSRFGKFIRIHFGSTGLLASGDIEHYLLEKSRVIYQQEGERNYHIFYQIISGSKPELIDQLLVTKDPYDYKSISQGVVSVDNLDDGAELLLTDDAFRVLGFNQEEITGIYRIMAGIMHQQNMKFKNKQREEQAEPDGTEDADKVAYLFGLNSADFIKYICHPRVKVGNEYVTKGQSCNQVSYGMGALSKGLFGRHFDWLVKLINQTLSTKLPRSFFIGVLDIAGFEIFDSNSFEQLCINFTNEKLQQFFNHHMFVLEQEEYKKEGIDWVFIDFGMDLAACIELIEKPLGIMSILEEECMFPKASDDTFKDKLYQNHLGKSKAFGKPVKKTKYEAHFELYHYAGTVAYNICGWLEKNKDPLNNSVVDLYKKASLKLMQTIWEGYISPEEAASGGGKGGKRKKGGSFMTVSSLHRQSLNSLMTNLRSTAPHFVRCLIPNERKCPGEMESHLVLHQLRCNGVLEGIRICRKGFPNRVPYGDFKQRYRILNPNAAPEGQFMDSKKASEKLLGSIDIDHESYKLGHTKVFFRAGMIGRLEELRDNKLASIFKLLQARMRGMLMRKEYQKMIERRQACRIIQSNLRAFFGMANCEWMKLMFKIKPLLKTAESAKELEEMEKEFAETKVNLEKETKRRKELEEMQVSFIQEKNDLVMQLQAQQDQIDDGEDRCDQLIKTKVELDGKIKELTERLEDEEELNNELVSKKRKLEDECSELKKDIDDLEITLAKVEKEKHATENKLKNLQEELATQDEQIAKLQKEKKALQEAHQQTLDDLQSEEDKVNSLTKQKAKLEQQVDDLEASLEQEKKLRMELERTKRKLEGDLRLTQETVMDLENDKQRLEEKLKKQEFEYSQLATKLEDEQALVSQLQKKIKELQARIEELEEELEAERAARAKVEKQRADLSRELEELSERLEEAGGATAAQIELNKRREAEFAKLRRELEESNLGHEATVSTLRKKNADTSSEMSEQIDNLQRVKQKLEKEKSEMKMEIDDLASNVESVTKAKLNYEKMARNLEEQLNETKMKNDNFTKEVNELNAAKARLSSENGEFGRQLEEREHLMAQLTRSKNSSSQQIDELKRVVEEETKAKAALAHAVQASRHDNDLLREQYEEEQEAKAELQRALSKANAEVAQWRNKYETDAIQRTEELEEAKKKLAIRLQEAEEQVEAMQAKASSLDKTKNRLQSELEDLTIDLEKSNSAAAALDKKQRNFDKILAEHKQKAEEIQVELEQSQKEARSLSTELFKMKNAYEESLDALETVKRENKNLQEEIADLTDQLGEGGKSIHELEKAKRTLEHERNEMQSALEEAEGAIEGEESKVLRLQVELAQIKQEFERRLAEKEEEVDNQRRNQQRSIESMQTTLDSESKARQEAVRIKKKMEGDLNDLEIQLGHANRQASEAQKQAKSIQSHVKDLEMQVDEAQRHAEDLQEQSAVIERRGNLLTAEIEELRSALEQAERGRKLAETELLESSERSNLLHTQNTALINQKRKLEGELQTMQGEVEESVQEQRNAEDKAKKAIVDAATMAEELKKEQDLSSHLERMKKNMEQTVKDLQQRLDEAENIALKGGKKQVQKLETRIRELENELDSEQRRNGDSVKSQRKIERRLKEVSYQGEEDKKNLTRIQDLVDKLQIKVKTYKRQAEEAEEQANTNLSKYRKLQHELDDAEERAEMAESQLNKMRSKARDTKGSN

>Ci_MYH2
MQEVHVKEDNIDEQNPPKFTLIEDMANMTYLNEPSVLNNLKLRYEKFLIYTYSGLFCVTINPYKLLPVYETYVVGCYKGKRRAEMPPHIFSIADNAYNDMLRNRENQSMLITGESGAGKTVNTKRVIQYFATVAALGDAKVEDGKVYCTLEDQIIQANPAMEAFGNAKTIRNDNSSRFGKFIRIHFGTTGKLASGDIETYLLEKSRVIFQQPGERGFHIFYQIISGAKPELLENLLITTDYDYKYMSQGDVAVASIDDADELNATDTAFDVLGFSQDEKNGIYRIMGSIMHTGNMKFKQKPREEQAEADGTEDADKVTYLLGINSAEFVKSILSPRVRVGNDYVTKGQTVQQCYYSTGALSKAVYEKLFNWLVKRINETLSTRLPRSFFIGVLDIAGFEIFDFNSFEQLCINFTNEKLQQFFNHHMFVLEQEEYKREGIDWVFIDFGLDLQACIELIEKPLGIMSILEEECMFPKATDLTFKEKLYMNHLGKSNNFIKPRPQIKRKFEAHFELIHYAGIVGYNISGWLEKNKDPLNNSVVALYKKSSLKVLAMIWESYVSPEEAMANKKSSGKGGGRRQKGGSFQTVSSLHRESLNRLMTNLRSTQPHFVRCIIPNEMKKPGYLDNALTLHQLRCNGVLEGIRICRKGFPSRILYAEFKQRYRILNPASIPDGQFLDSKKATEKLMASLELDVAQYRFGNTKIFFKAGMLGTLEDMRDERLTIIITRMQSRGRGKQMRVEFKKMLERKQACSLIQANIRAYLAVRNWVWMRLMFKIKPLLKSAENAKEMEQIEKEKADLEENYEREKKRRQELEDSQVSLIQDKNDLVLQLNAEQENLQDAEDRCDQLIKSKVEMESKLKDLSERLEDEEEANNDILSKKRKLEDECSELKKDIDDLELTLAKVEKEKHATENKVKNLNEEVSTLEESLERSNKEKKSLQEAHQQTLDDLQQEEDKXXXNFELEEMRSGLEQAERARKAAEAELMEVAERSNLLHTQNTALINQKRKQESEIVQIRGEVEELSEEQRHAEEKAKKAIIDAAMMAEELKKEQDQSSHLERMKKNLEQTIKDLQMRLDEAEQVALKGGRKQVHKLETRVRELENELDSEQRRSVEVTKVMRKSERRMKEVTYQADEDKKNLVRMQDLVDKLQVKVKTYKRQCEETEEQANLNLAKYRKLQHELDDAEERAEVAESSLNKLRAKARDGTSFANYKD

>Ci_MYH3
MPFLEPTEKEKLSYAAQNYDGKKSVWVPHKKEGFVKGDLVDSSGEKCTVKTVKNETVSLKKDDIQQMNPPKFEQTADMANMTFLNEASVLHNLRSRYASLRIYTYSGLFCVCVNPYKWLPVYGVKVVHMYRGKKRSEMPPHLFSVADNAYHDMLMDRENQSILITGESGAGKTENTKKVIQYFANIAASGVQKPGEEKKANLEDQIVQTNPVLEAWGNAKTIRNNNSSRFGKFIRIHFGTSGKLSGGDIESYLLEKSRVIFQLPAERGYHIFYQIMASGRKDMLESLCVSNNPRDYHWVSQGVISVDNMNDFEEFNFTDEAFEVLGFTEEERFNCYRLTCGCMVFGSMVYKQKPRDEQAEVDTVEVADKVAHLFGISSPDLCKAITRPRVKVGTEYVQKGQNVDQCFNSTGALAKATYDKLFKWIVFRLNITLDTKLPRNYFVGVLDIAGFEIFEFNTFEQLCINFTNEKLQQFFNHHMFVLEQEEYKKEGIEWTFIDFGMDLQACIELLEKPMGVFSILEEESIVPKATDETFKNKLYEKHEKKSEAFIKPKVTKKGNAHFSVKHYAGVVDYNVDGWLNKNKDPLNDSVVQLFQKSTNKLMSAIFPETKEEPAGKKKKKGGSFQTVSALYREQLNKLMTNLRNTKPHFVRCLIPNEMKQCGIMDAALVLAQLKCNGVLEGIRICRKGFPNRLQYPEFKQRYQILAASKVANMVDSKKATETILLHIELDTALYKIGHTKIFFKAGVLADLEDQRDDILAIIMTKMQSKARGKLMRIEFKKMLERQRAAKAIQRNIRKFLQFRDWQWWKLYTKVKPLLNIVRVEDELKAKDEEIAELKDKYSHEEKLRKEYEEKCVSLLSEKNDLTLQLQAEQENLADAEERNEQLVKVKGDLEGQVGDLSERLDEEEANNVQLSASKKKLEKQCEDLGHDIEEAESNINHLEKDKQGLELKLRSLSADLEQRDDSIQRLNKEKKQLDQVNQQTLEDLQAMEDKANHLGKIKIKLEQQVEDIEDSLEQERKHKADLEKSKRKLENDLRSAQDTIMDLEKDKASLEDALRKRDFDINQLNGRIEDEQNLASQLNRKVKELQARVEETESELDMERQARSKVERNRSELVRELDQLSEQLEEAGGATQAQIELIKRRESDYLKLRRDYEEAVMQNDATVGQLKKKHQDVVNELVEQVENLSRVKNKIEKDRAQLHMELDDVTTQLEEVSKLKANVRVMEEQVTDYKFKVEENIRIVNELTIIKNKLTSESMESSHQLEEAESKVSALSRAKSNMTSMLEDLKRQLEEESKSKQSLAHALQAARHDLDLLREQVEEEQEGKAELQRALSKANAEVANWRTKYETDAIQRMEELEEAKKKLAIRLQEAEEQTETALAKCASLDKTKIRLTNEVEDLTIDLERANATISALDKKQRNFDKEISTWMLKVEELQAELDGAMRENRNYQTEIYKIKVSYEESIEQLEIVKRENKNLSEEINDLTDQLTTGGKSLHELDKARKKAELECEELRSALEEAEGALELEESRVLRLQLELTQVKADIDRKLQEKEEEFDSTRKNHQRAIESMQASLDVEIKSRTDAVRAKKKLETQLNDAEMQLDHANRNLAEQIKLVRKLQVTIKEIQDQMDEDQRIHEELREQYSIQERRLTITISELEETKSALESNERARKHAEAELLDISDRINTLSAQNSALSSAKRKLETDNEQLRGDLEDALMEAKTADERAKKATSDAARMSEELRSEQQHILSIERVKKTLEVQVHEMSIKLDEAEAYALKGGRKALAQLQARLKDVQNELEAEQRRHAETLKNYRKMDRRLKELSFQADEDQKNQVRMQELVEKLQLKLKQYKKMAEEAEEQANQNLAKYRKVVHELEEAEERAEISESALNKVRSKSRYMTSSVGGGGGGGYSYSISRKVVTTKGSSSTS


>Tr_MYH_M86
MSTDAEMEQYGPASIYLRKPERERIEAQNTPFDAKTAYFVAEPAEMYLKGKLIKREGGKATVETVTGKTITVKEDDIHAMNPPKYDKIEDMAMMTHLNEPAVLYNLKERFASWMIYTYSGLFCVVVNPYKWLPVYDAQVVNAYRGKKRIEAPPHIFSISDNAYQFMLTDRENQSILITGESGAGKTVNTKRVIQYFATIAVAGGGKKAEQGSGKIQGSLEDQIIAANPLLEAYGNAKTVRNDNSSRFGKFIRIHFGTTGKLASADIETYLLEKSRVTFQLSAERSYHIFYQLMTGHKPELLEALLITTNPYDYPMISQGEITVKSIDDVEEFIATDTAIDILGFTADEKINIYKLTGAVMHHGTMKFKQKQREEQAEPDGTEVADKIAYLLGLNSADMLKCLCYPRVKVGNEMVTKGQTVPQVNNAVSALCKSVYEKMFLWMVVRINEMLDTKQSRSYFIGVLDIAGFEIFDFNSLEQLCINFTNEKLQQFFNHHMFVLEQEEYKKEGIDWEFIDFGMDLAACIELIEKPMGIFSILEEECMFPKASDTTFKNKLHDQHLGKTKAFEKPKPGKGKAEAHFALVHYAGTVDYNISGWLDKNKDPLNDSVVQLYQKSSNKLMSLLYAARAGDEAAAGAGKKAGKKKGGSFQTVSALFRENLGKLMTNLKSTHPHFVRCLIPNESKTPGLMENFLVIHQLRCNGVLEGIRICRKGFPSRILYGDFKQRYKVLNASVIPEGQFIDNKKASEKLLGSIDVDHTQYKFGHTKVFFKAGLLGTLEEMRDEKLAELVTMTQALCRGYLMRKEFVKMMERRESLFTIQYNVRSFMNVKNWPWLKLYFKIKPLLKSAETEKELAQMKDNYEKMQSDLATALAKKKELEEKMVSLLQEKNDLQLQVASEFDNLSDAEERCEGLIKSKIQLEAKLKETTERLEDEEEINAELTAKKRKLEDECSELKKDIDDLELTLAKVEKEKHATENKVKNLTEEMASQDESIAKLTKEKKALQESHQQTLDDLQAEEDKVNTLTKAKTKLEQQVDDLEGSLEQEKKLRMDLERAKRKLEGDLKLAQESIMDLENEKQQSDEKIKKRDFEISQLLSKIEDEQSLGAQLQKKIKELQARIEELEEEIEAERAARAKVEKQRADLSRELEEISERLEEAGGATAAQIEMNKKREAEFQKMRRDLEESTLQHEATAAALRKKQADSVAELGEQIDNLQRVKQKLEKEKSEYKMEIDDLSSNMEAVAKSKGNLEKMCRTLEDQLSELKSKNDENVRQLNDINAQRARLQTENGEFGRQLEEKEALVSQLTRGKQAFTQQIEELKRHVEEEVKAKNALAHGVQSARHDCDLLREQYEEEQEAKAELQRAMSKANSEVAQWRSKYETDAIQRTEELEEAKKKLAQRLQDAEESIEAVNSKCASLEKTKQRLQGEVEDLMIDVERANSLAANLDKKQRNFDNFLAEWKQKYEEGQAELEGAQKEARSLSTELFKMKNSYEEALDHLETMKRENKNLQQEISDLTEQIGETGKSIHELEKSKKTVETEKSEIQAALEEAEGTLEHEEAKILRVQLELNQIKGEVDRKLAEKDEEMEQIKRNSQRVIDSMQSTLDAEVRSRNDALRVKNKMEGDLNEMEIQLSHANRQAAEAQKQLRNVQGQLKDAQLHLDDAVRGQEDMKEQFAMVERRNGLMMAEIEELRAALEQTERGRKVAEQELVDASERVGLLHSQNTSLINTKKKLEADLVQVQGEVDDSIQEARNAEEKAKKAITDAAMMAEELKKEQDTSAHLERMKKNLEVTVKDLQHRLDEAESLAMKGGKKQLQKLESRVRELEAEVEAEQRRGVDAVKGVRKYERRVKELTYQTEEDKKNVSRLQDLVDKLQLKVKAYKRQAEEAEEQANTHMSRLRKVQHEMEEAQERADIAESQVNKLRVKSRDMGKSDSAE

>Tr_MYH_M2528
MGDAEMECFGPAAIYLRKPERERMEAQNTPFDAKSAYFVVEPKEMYLKGKLVKKEGGKATVETLCGKSITVKDTEIFPMNPPKFDKIEDMAMMTHLSEPSVLYNLKERYAAWMIYTYSGLFCVTVNPYKWLPVYDSMVVAGYRGKKRVEAPPHIFSISDNAYQFMLTDRENQSILITGESGAGKTVNTKRVIQYFATIAVAGGKKEPVPGKMQGSLEDQIIAANPLLEAYGNAKTVRNDNSSRFGKFIRIHFATTGKLASADIETYLLEKSRVTFQLSAERSYHIFYQLMTGHKPELIEALLITTNPYDYHMISQGEITVKSINDIEEFIATDTAIDILGFTGEEKISMYKLTGAVMQHGNMKFKQKQREEQAEPDGSEVADKIAYLMGLNSADLLKALCYPRVKVGNEFVTKGQTVPQVNNSVMALCKSVYEKMFLWMVVRINEMLDTKQSRQFFIGVLDIAGFEIFDYNSLEQLCINFTNEKLQQFFNHHMFVLEQEEYKKEGIEWEFIDFGMDLAACIELIEKPMGIFSILEEECMFPKATDMTFKNKLYDQHLGKTKCFEKPKPAKGKAEAHFSLVHYAGTVDYNITGWLDKNKDPLNDSVVQLYQKSSVKLLSFLYASHASSEAETGGGGKKGGKKKGGSFQTVSALFRENLGKLMTNLRSTHPHFVRCLIPNESKTPGLMENFLVIHQLRCNGVLEGIRICRKGFPSRILYGDFKQRYKVLNASVIPEGQFIDNKKASEKLLGSIDVDHTQYRFGHTKVFFKAGLLGVLEEMRDEKLAELVTMTQALCRGFLMRREFVKMMERRDAIFTIQYNIRAFMNVKTWPWMKLYFKIKPLLKSAETEKEMAQMKEDFEKTKEDLSKALSKKKELEEKMVSLLQEKNDLLLQVQTESENLSDAEERCEGLIKAKIQLEAKKETTERLEDEEEINAELTAKKRKLEDECSELKKDIDDLELTLAKVEKEKHATENKVKNLTEEMASQDEAIAKLSKEKKALQEAHQQTLDDLQAEEDKVNTLTKAKTKLEQQVDDLEGSLEQEKKLRMDLERAKRKLEGDLKLAQESIMDLENDKQQSDEKIKKKDFEISQLLNKIEDEQTLGIQLQKKIKELQARIEELEEEIEAERAARAKVEKQRSDLSRELEEISERLEEAGGATSAQIEMNKKREAEFQKLRRDLEESTLQHEATAAALRKKQADSVAELGEQIDNLQRVKQKLEKEKSEYKMEIDDLSSNMEAIAKSKSNLEKMCRTLEDQLSELKSKNDENVRQLNDASTQKSRLQTENGEYLRQLEEKEALVSQLTRGKQAYTQQIDELKRHIEEEVKAKSALAHAVQSSRHDCELLREQYEEEQEAKAELQRSMSKANSEVAQWRTKYETDAIQRTEELEEAKKKLAQRLQDAEESIEAANSKCASLEKTKQRLQGEVEDLMIDVERANAVAASLDKKQRNFDKVLAEWKQKYEESQAELEGAQKEARSLSTELFKMKNSYEEALDHLETLKRENKNLQHEISDLSEQLGETGKTIHELEKGKKTVEGEKAEIQTALEEAEATLEHEESKILRVQLELTQIKSEIDRKLAEKDEEIEQIKRNSQRVIESMQSTLDAEVRSRNDALRIKKKMEGDLNEMEIQLSHANRQAAEAQKQLRNIQGQLKDAQLHLDEALRGQDDMKEQVAMVERRNNLMLAEIEELRAALEQTERSRKVAEQELVDASERVGLLHSQNTSLINTKKKLEGDLVQIQGEVEEAVQEARNAEEKAKKAITDAAMMAEELKKEQDTSAHLERMKKNLEVTVKDLQHRLDEAENLAMKGGKKQLQKLEARVRELEGEVEAEQRRGADAIKGVRKYERRVKELTYQTEEDKKNVARLQDLVDKLQLKVKAYKRQAEEAEEQANTHLSKYRKVQHELEEAQERADIAESQVNKLRAKSREIGK

>Tr_MYH_M1034
MGDAEMECYGPAAVFLRKPEKERIEAQNTPFDAKTAYFVTEPKEMYLKGKLIKKEGGKATVETLCKKTITVKDDEIFPMNPPKFDKIEDMAMMTHLSEPTVLYNLKERYAAWMIYTYSGLFCVTVNPYKWLPVYDSVVVAGYRGKKRIEAPPHIFSISDNAYQFMLQDRENQSILITGESGAGKTVNTKRVIQYFATIAVAGGKKEQQSSSKMQGSLEDQIIAANPLLEAYGNAKTVRNDNSSRFGKFIRIHFGTTGKLASADIETYLLEKSRVTFQLSAERSYHIFYQLATGHKPELIEALLITTNPYDFPMISHGEITVKSIDDIEEFIATDTAIDILGFTAEEKASMYKLTGAVMHHGNMKFKQKQREEQAEPDGTEVADKIAYLMGLNSADLLKALCYPRVKVGNEFVTKGQTVPQVNNSVMALSKSVYEKMFLWMVVRINEMLDTRQSRSFFIGVLDIAGFEIFDYNSLEQLCINFTNEKLQQFFNHHMFVLEQEEYKKEGIEWEFIDFGMDLAACIELIEKPMGIFSILEEECMFPKATDMTFKNKLYDQHLGKSAPFQKPKPAKGKAEAHFSLMHYAGTVDYNVTGWLDKNKDPLNDSVVQLYQKSSVKLLAYLYAAHGGAEEGGGAKKGKKKGGSFQTVSGLFRENLGKLMTNLRSTHPHFVRCLIPNESKTPGLMENFLVIHQLRCNGVLEGIRICRKGFPSRILYGDFKQRYKVLNASVIPEGQFIDNKKASEKLLGSIDVDHTQYKFGHTKVFFKAGLLGTLEEMRDEKLVELVTMTQALCRAYLMRREFVKMMERRESLFTIQYNIRSFMNVKTWPWMKLYFKIKPLLKSAEAEKEMAQMKEDFEKTKEDLAKALAKKKELEEKMVSLLQEKNDLQLQIQSESETLSDAEERCEGLIKAKIQLEAKAKETAERLEDEEEINAELTAKKRKLEDECSELKKDIDDLELTLAKVEKEKHATENKVKNLVEEMASQDEAIAKLSKEKKALQEAHQQTLDDLQAEEDKVNTLTKAKTKLEQQVDDLEGSLEQEKKLRMDLERAKRKLEGDLKLAQESIMDLENDKQQSDEKIKKKDFEISQFLSRIEDEQALSIQFQKKIKELQARIEELEEEIEAERAARAKVEKQRSDLSRELEEISERLEEAGGATSVQIEMNKKREAEFQKLRRDLEESTLQHEATAAALRKKQADTTAELGDQIDNLQRVKQKLEKEKSEYKMEIDDLSSNMEAIAKSKTHLEKLCRTLEDQMSEMKTKSDENVRQLNDIGLQRARLQTENGEISRQLEEKETLVSQLTRSKQAFIQQIEELKRHIEEEVKAKNALAHAVQSSRHDCELLREQYEEEQEAKAELQRSMSKANSEVAQWRTKYETDAIQRTEELEEAKKKLAQRLQDAEESIEAVNAKCASLEKTKQRLQGEVEDLMIDVDRANALAASLDKKQRNFDKVLAEWKQKYEESQAELEGAQKEARSLSTEMFKMKNSYEEALDHLETLKRENKNLQQEISDLTEQIGETGKTIHELEKGKKTAESEKCELQTSLEEAEATLEHEESKILRIQLELTQVKSEIDRKIAEKDEEIEQIKRNSQRVIESMQSTLDAEIRSRNDALRIKKKMEGDLNEMEIQLSHANRQAAEAQKQLRNVQGQLKDAQLHLDEAIRGQEEMKEQVAMVERRNNLMLAEIEELRAALEQTERSRKVAEQELVDASERVGLLHSQNTSLINTKKKLEADLIQIQGEVEDSIQEARNAEEKAKKAITDAAMMAEELKKEQDTSAHLERMKKNLEVTVKDLQHRLDEAENLAMKGGKKQLQKLEARVRELEGEVEAEQKRGADAIKGVRKYERRVKELTYQTEEDKKNLVRLQDLVDKLQLKMKSYKRQAEDAEEQANSHLTRYRKVQHELEEAQERADIAESQVNKLRVKSREIVK

>Tr_MYH_M743
MSTDAEMAIYGKAAIYLRKPEKERIEAQNKPFDAKSACYVTDTKELYLKGTILKKDGAKVTVKVLGTEEERTVKEDDVTPMNPPKFDKIEDMAMMTHLNEASVLYNLKERYAAWMIYTYSGLFCATVNPYKWLPVYDSEVVSAYRGKKRMEAPPHIFSVSDNAYQNMLTDRENQSVLITGESGAGKTVNTKRVIQYFATIAVGGGEKKKESKMGGSLEDQIIAANPLLEAYGNAKTVRNDNSSRFGKFIRIHFGTSGKLSSADIETYLLEKSRVTFQLPDERGYHIFYQMMTNHKPELIEMSLITTNPYDFPMCSMGQITVASIDDKVELEATDNAIDILGFTHEEKMSIYKMTGAVLHHGNMKFKQKQREEQAEPDGTEDADKVAYLLGLNSADMLKALCYPRVKVGNEFVTKGQTVPQVNNSVPALAKSIYERMFLWMVVRINQMLDTKQPRQFFIGVLDIAGFEIFDYNSMEQLCINFTNEKLQQFFNHHMFVLEQEEYKKEGIHWEFIDFGMDLAACIELIEKPMGIFSILEEECMFPKATDTSFKNKLYDQHLGKNKAFEKPKPAKGKAEAHFSLVHYAGTVDYNISGWLDKNKDPLNESVVQLYQKSPVKLLSTLYPPVVDEPAGGKKGGKKKGGSMQTVSSQFRENLGKLMTNLRSTHPHFVRCLIPNESKTPGLMENFLVIHQLRCNGVLEGIRICRKGFPSRIVYGDFKQRYKVLNASVIPEGQFIDNKKASEKLLGSIDVDHDQYRFGHTKVFFKAGLLGTLEEMRDDKLAALVTMTQALCRGYLMRKEFVKMTARRDAIYTIQYNVRSFMNVKNWPWMHVYYKIKPLLKSAETEKELAQMKENYEKMQTDLAAALAKKKELEEKMVSLLQEKNDLQLQVASESENLSDAEERCEGLIKSKIQLEAKLKETTERLEDEEEINAELTAKKRKLEDECSELKKDIDDLELTLAKVEKEKHATENKVKNLTEEMASQDESIAKLTKEKKALQEAHQQTLDDLQAEEDKVNTLTKAKTKLEQQVDDLEGSLEQEKKLRMDLERAKRKLEGDLKLAQESIMDLENDKQQSDEKMKKKDFEISQLLSKIEDEQSLGAQLQKKIKELQARIEELEEEIEAERAARAKVEKQRADLSRELEEISERLEEAGGATAAQIEMNKKREAEFQKLRRDLEESTLQHEATAAALRKKQADSVAELGEQIDNLQRVKQKLEKEKSEYKMEIDDLSSNMEAVAKAKGNLEKMCRTLEDQLSELKTKNDENVRQINDLGAQKARLLTENGEFGRQIEEKEALVSQLTRGKQAFTQQIDELKRQIEEEVKAKNALAHGLQSARHDCDLLREQFEEEQEAKAELQRGMSKANSEVAQWRTKYETDAIQRTEELEEAKKKLAQRLQEAEEQIEAVNSKCASLEKTKQRLQSEVEDLMIDVERANGLAANLDKKQRNFDKVLAEWKQKYEEGQAELEGAQKEARSLGTELFKMKNSYEEALDQLETMKRENKNLQQEISDLTEQIGETGKSIHELEKAKKQVETEKAEIQTALEEAEGTLEHEESKILRVQLELNQIKGEIDRKLAEKDEEIEQIKRNSQRVTDSMQSTLDSEVRSRNDALRIKKKMEGDLNEMEIQLSHANRQAAESQKQLRNVQAQLKDAQLHLDDAVRAQEDLKEQAAMVDRRNGLMIAEIEELRAALEQTERSRKVAEQELVDASERVGLLHSQNTSLMNTKKKLESDLVQIQSEVDDTVQEARNAEEKAKKAITDAAMMAEELKKEQDTSAHLERMKKNLEVAVKDLQHRLDEAENLAMKGGKKQLQKLESRVRELETEVETEQRRGADAVKGVRKYERRVKELTYQTEEDKKNVTRLQDLVDKLQLKVKAYKRHSEEAEEQANVHLSKCRKLQHELEEAEERADIAESQVNKLRAKSRDSGKGKDAAE

>Tr_MYH_M454
MSSDAEMAQYGPAAVYLRKPEKERLEAQNRPFDARTACFVPDAKELYVKGIVQSREGGQVIVKTQADETVKVKEEDCLPMNPPKYDKIEDMAMMTHLNEPSVLFNLKERYAAWMIYTYSGLFCATVNPYKWLPVYDPQVVAAYRGKKRMEAPPHIFSVSDNAYQNMLTDRENQSVLITGESGAGKTVNTKRVIQYFATIAVSGGDKKEQSGKMQGTLEDQIISANPLLEAFGNAKTVRNDNSSRFGKFIRIHFGTTGKLSSADIETYLLEKSRVTFQLSEERSYHIFYQIMTGHKPELIEMLLITTNPYDFPMISQGQISVQSIDDKEELLATDMATDILGFTNEEKVSIYKLTGAVMHYGNMKFKQKQREEQAEPDGTEVADKVAFLMGLNSADLLKGLCYPRVKVGNEYVTKGQTVPQVTNAVGALAKSVYEKMFLWMVIRINEMLDTKQPRQFFIGVLDIAGFEIFDFNSMEQLCINFTNEKLQQFFNHHMFVLEQEEYKKEGIDWEFIDFGMDLAACIELIEKPMGIFSILEEECMFPKATDVSFKNKLYDQHLGKNNAFQKPKVVKGKPEAHFSLLHYAGTVDYNISGWLEKNKDPLNESVVQLYQKSSIKLLAVLYASFSGAEAGGKKGGKKKSGSFQTVSAVFRENLGKLMTNLRSTHPHFVRCLIPNESKTPGIMDNHLVIHQLRCNGVLEGIRICRKGFPSRILYADFKQRYRILNASAIPEGQFIDGKKASEKLLGSIDVDHTQYRFGSTKVFFKAGLLGTLEELRDEKLASLVTQTQALCRGYVMRKEFNNLIARRDCVWILQYNLRSFMSVKHWPWMKLFFKIKPLLKSAKTEKEMATMKDDFIKCKDDLAKSEAKRKELEEKMVSLLQEKNNLLLQVQSDSENLCDAEERCEGLIKSKIQLEAKLKEVSERLEDEEDVTAELTAKKRKLEDECSELKKDIDDLEMTLAKVEKEKHATENKMKNLMDELSGQDENIGKLTKEKRALQEAHQQVLDDLQAEEDKVNTLTKAKSKLEQQVNDLEGSLEQEKKIRMDLERAKRKLEGDLKITQESVMDLENDKQQSEEKIKKKEFENNQLLSKIADEQAMNNQLQKKMKELHARIEELEEEVEAERAVRAKIEKQRSDLLREIEEISERLEEAGGATTAQVEINKRREAEFLKLRRDLEESTLHHEATTAALRKKHADSMAELGEQVDNLQRIRQKLEKEKSELKMEIDDLSINMENVAKAKVNLEKMCRSLEDQQMELKTKNDEHMRQLTDVINQRARFQTENAEFSRQMEERESLISQLTRGKQGFTTQIDELKRLIDEESKAKNALAHSLQSARHDCDLLREQFEEEQEAKAELQRSLSKANSEVALWRNKYETDAIQRTEELEEAKKKLAQRLQEAEEQIEAVNSKCASLEKTKQRLQSEMEDLMVDMEKSNSVATSLDKRQRNFDKIQAEWKQKYEESQAELESLQKESRSLNTELFKLKNSYEEALDHLETMKRENKNLQQEVSDLTEQVGESGKTIHELEKFKKQAETEKYEMQTSLEEAEASVEQEESKILRVQMEFNQVKAEIDRKLAEKDEEMDQMKRNHQRVMESIQATLDAEVRSRNDALRVKKKMECDLNEMEIQMSHANRQAAEAQKQLRNIQGQLKDAQIHLDDSTRGQDDMKEQVAMMERRTALLQAEVEELRAAVEQTERSRKLAEQELVDASERAGLLHSQNTSLLNTKKKLESDITQLHSEIEEALQEARNVEEKAKKAITDAAMMAEELRKEQDTSANLERMKKNLEATVKDLQHRLDEAENLAMKGGKKQLQKLEARVRELETELEAEQKRCSEAVKGVRKYERKVKELTYQSDEDKKNNIRLQDLVDKLQNKMKIYKRQAEEAEEQSNVHASRFRKVQHDLEEAEERADTAESLANRMRAKSREIGSK

>Lc_MYH1
MGDLHEFGKAAPFLRKSEKERLEAQTRPFDMKKNCFVDDPKIEFVKATIKSSEGGKVTVDTENGQTVTVREDQVHQMNPPKFDKIEDMVMLTFLNEPSVLFNLKERYAAWMIYTYSGLFCVTVNPYKMLPVYDPIVVAGYRGKKRQEAPPHIFSISDNAYQFMLSDRENQSILITGESGAGKTVNTKRVIQYFATIAALGDSAKKKQEGKIKGTLEDQIVEANPLMEAFGNAKTVRNDNSSRFGKFIRIHFGATGKLASADIETYLLEKSRVTFQLPTERNYHIFFQILSQQKPELLDMLLVTTNPYDYPFISQGEITVSSIDDKEELMATDSAIEILGFTNEEKMSIYKLTGSIMHYGNMKFKQKQREEQAEPDGSEDADKACFLMGINSADLLKALCLPRVKVGNEYVTKGQTVPQVYNNIGALGKACFERLFLWMVIRINQSLDTKQARQHFIGVLDIAGFEIFDFNTFEQLCINFTNEKLQQFFNHHMFVLEQEEYKKEGIDWVFIDFGMDLQACIDLIEKPLGIMSILEEQCMFPKANDQTFKEKLYDQHLGKNKFFQKPKPSKGKHEAHFSLGHYAGNVDYNIAGWLEKNKDPLNDSVVQVYQKASLKVLGALFATFAGADADSGAKKGKKKGSSFQTVSALHRENLSKLMTNLKTTHPHFVRCLIPNETKTPGTMDNNLVMHQLRCNGVLEGIRICRKGFPNRIVYGDFKQRYRVLNPNAMPEGQFVDSKKACEKLMASLELDETQYKFGHTKVFFRAGLLGTLEEMRDDRLSLLLTRTQALGRGYLSRVEFKKMLERRESLIVIQYNIRAFMGVKNWPWMNLYFKIKPLLKSAESEKEMANMKEEFIKTKEALEKSEARRKELEEKMISVVQEKNDLLMQVQSGEDSINDSEERCDQLIKAKIQLESKLKEMQERIEEEEEMNSELTAKKRKLEDECSELKKDIDDLELTLAKVEKEKHATENKVKNLTEEMAVLDENVSKLTKEKKALQEAHQQTLDDLQAEEDKVNSLTKAKAKLEQQVDDLEGSLEQEKKVRMDIERTKRKLEGDFKMAQESIMDLENDKQQIDEKTKKKDFEISQLNSKIEDELALSAQLQKKIKELQARIEELEEEIEAERAARAKVEKQRSDLSRELEEISERLEEAGGATSAQIEMNKKREAEFQKLRRDLEESTLQHEATAAALRKKQADSVAELGEQIDNLQRVKQKLEKEKSEFKMEVDDLSSNMEQITKSKVNLEKLSRSLEDQLSELKTKDDENIRTINDITAQRARLQTESGELTRKLEEKEVLVVQLSRGKQSYTQQVEDLKRQLEEEVKAKNALAHAVQSARHDCDLLREQFDEEQEAKGELQRAMSKANAEVAQWRTKYETDAIQRTEELEDAKKKLAARLQDAEEQVEAANSKCASLEKTKQRLQGEVEDLMIDVERAHSAAAALDKKQKAFDKVLSEWKQKFEESQAELEAAQKESRSLGTELFKLKNAYEETLDHLETFKRENKNLQEEISDLTEQLGESSKSLHELEKNRKAIEQEKSDIQAALEEAEASLEHEEGKILRVQLELNQVKAEVDRKIAEKDEEIDQIKRGFQRTVESMQATLDSETKSRNEALRLKKKMEGDLNEMEIQLNHANRQAAESQKHLRNVQGQIKDLQVHLDDALRANEDHKEQAAMLERRNNLLVAEVEEMRSALEQTEKARKLAEQELLGATERVQLLHSQNTSLINTKRKLEGDLSQLQSEVEESIQESRNADEKAKKAITDAAMMAEELKKEQDTSAHLERMKKNMEQTVKDLQHRLDEAEQIAMKGGKKQLQKLETRVRELESELDAEQRRTAETIKGSRKYERRIKELSYQTEEDRKNNLRLQDLVDKLQLKVKAYKRQSEESEEQANTNLSKYRKLQHELEEADERADIAESQVNKLRARSRDSGVVKAGESE

>Lc_MYH2
MGDLHEFGKAAPFLRKSEKERLEAQTRPFDMKKECYVDDPKVEFVKATIKSSEGGKVTVDTETGQTVTVKDSQVHQMNPPKFDKIEDMAMLTFLNEPSVLFNLKERYAAWMIYTYSGLFCVTINPYKWLPVYDPIVVAGYRGKKRQEAPPHIFSISDNAYQFMLTDRENQSILITGESGAGKTVNTKRVIQYFATIAALGDATKKKEESRKGTLEDQIVEANPLMEAFGNAKTVRNDNSSRFGKFIRIHFGATGKLSSADIETYLLEKSRVTFQLPTERNYHIFYQIESQKKPELLEMLLITNNPYDYVFISQGEITVPSLDDGDELMATDSAIDILGFTNDEKLSIYKVTGALMHYGNMKFKQKQREEQAEPDGNEDADKAAYLMGLNSADLLKGLCYPRVKVGNEYVTKGQTVQQVHNSVGALARSVFEKMFLWMVIRINETLATKLARQHFIGVLDIAGFEIFDFNTFEQLCINFTNEKLQQFFNHHMFVLEQEEYKKEGIDWVFIDFGMDLQACIDLIEKPMGIMSILEEQCMFPKANDQTFKEKLYDQHLGKNQFFQKPKPSKGKHEAHFSLGHYAGNVDYNIGGWLEKNKDPLNDSVVQLYQKSSLKMLAAVYATYASVDSDTGSKGGKKKKGSSFQTVSALHRENLNKLMTNLKTTHPHFVRCIIPNETKTPGTMDNILVMHQLRCNGVLEGIRICRKGFPNRIVYGDFKQRYRVLNPNAIPEGQFMDSKKACEKLMASLELDDTQYKFGHTKVFFRAGLLGTLEEMRDDRLSLLLTRTQALARGYLSRVEFKKMLERRESLIIIQYNIRAFMGVKNWPWMNLYFKIKPLLKSAESEKEMANMKEEFIKTKEALEKSETRRKELEEKMISVMQDKNDLLIQVQSSEETLNDAEERCDQLIKTKIQLEGKIKEIQERVEEEEEMNAEITAKKRKLEDECSELKKDIDDLELTLAKVEKEKHATENKVKNLTEEMAVLDENVSKLTKEKKALQEAHQQTLDDLQAEEDKVNSLTKAKAKLEQQVDDLEGSLEQEKKVRMDVERTKRKMEGDLKLAQESLMDLENDKQQLDEKLKKKDFEMSQLNSRIEDEVSLSAQLQKKIKELQARIEELEEEIEAERAARAKVEKQRSDLSRELEEISERLEEAGGATSAQIEMNKKREAEFQKLRRDLEEATLQHEATAAALRKKQADSVAELGEQIDNLQRVKQKLEKEKSEFKMEVDDLSSNLEQVTKAKVNLEKLCRTIEDQLAEVKTKEEEQIRSINDINTQRARLLAENGELSRKLEEKEVLVVQLSRGKLSYTQQVEDLKRQLEEEIKAKNALAHAVQSARHDCDLLREQFDEEQEAKGELQRAMSKANAEVAQWRTKYETDAIQKTEELEEAKKKLAARLQEAEEQVEASNSKCASLEKTKQRLLGEVEDLMIDVERAHSAAAALDKKQKAFDKILSEWKQKYEESQSELEASQKESRSLSTEVFKLKNAYEETLDHLETLKRENKNLQEEISDLTEQLGESGKALHEVEKSRKQLEQEKLEIQAALEEAEASLEHEEGKILRIQLELNQVKADIDRKIAEKDEELDQLKRSNQRTVESMQASLDAETKSRNEALRLKKKMEGDLNEMEIQLNHANRQAAESQKHLRNVQGQIKDLQVHLDDTLRSNDDLKEQVAMIERRNNLLLAEVEEMRTALEQTERARKVAEQELLGATERVQLLHSQNTSLINTKRKLEGDIVQLQNECEEAIQESRNADEKAKKAITDAAMMAEELKKEQDTSAHLERMKKNMEQTVKDLQNRLDEAEQIAMKGGKKQIQKLETRVRELESELDSEQRRIAETIKGARKYERRVKELTYQAEEDRKNNLRLQDLVDKLQLKVKTYKRQSEEAEEQANTNLSKYRKMQHELEEADERADIAESQVNKLRARSRDVHPTKAEAE

>Lc_MYH5
MGDLRDFGEAAPFLRKSEKERLEAQHRPFDTKKEVYVVDVKDMYVKATIKSSDGGKVTVETEDGRTVTVKAEDVHQMNPPKFDKIEDMAMLTFLNEAAVLYNLKERYAAWMIYTYSGLFCVTVNPYKWLPVYNKEVVAGYRGKKRQEAPPHIYSISDNAYQSMLTDRENQSVLITGESGAGKTVNTKRVIQYFATIAALGEATKKEASQSQMKMTLEDQIVQANPLMEAFGNAKTVRNDNSSRFGKFIRIHFGASGKLASCDIETYLLEKSRVTFQLPTERNYHIFFQIISQQKPELIDMLLITTNPYDYVFISQGEITVASIDDKEELMATDSAIDILGFTNDEKLSIYKLTGSIMHYGNMKFKQKQREEQAEPDGVEDADKACYLMGINSADLLKALCYPRVKVGNEVVTKGQTVKQVYNNIGALAKTVFEKLFLWMVIRINQSLDTKKARQHFIGVLDIAGFEIFDYNTFEQLCINFTNERLQQFFNHHMFVQEQEEYKKEGIDWEFIDFGMDLQACIDLIEKPMGIMSILEEQCMFPKSNDSTFKEKLYDQHLGKSSFFQKPKPSKGKHEAHFTLGHYAGNVDYNIAGWLEKNKDPLNDSVVQIYQKASLKVLGALFANFASADADAAGAKKAKKKGSSFQTVSALHRENLNKLMTNLKTTHPHFVRCLIPNETKTPGVMDNNLVMHQLRCNGVLEGIRICRKGFPNRILYGDFKQRYRILNPSAMPDGQFIDNKTGCEKLMDSLQLDNLQYKFGYTKIVFRAGLLGTLEEMRDDRLSLLLTRTQAVTRGYLMRKKMKQLMETRDSIIVIQYNIRVFMGVKNWPWMNLYFKIKPLLKSAESEKEMANMKEEFIKTKEALEKSEARRKELEEKMISVVQEKHDLLMQVESVEGTMSDAEERCNELIKSKIHLEAKLKEAQERADEEEELNAELTTKKRAFEDECSELKKDIDDLELTLAKVEREKHATENKVKTLSEEMAALEENIAKLTKEKKALQEAHQQTLDDLQAEEDKVNSLTKAKAKLEQQVDELEGSLEQEKKTRMDVERAKRKVDGDFKMSQEAVTDLENDKQQLEEKMKKKDVEIGQTNSKMQDEMALSAQLQKKIKELQARIEELEEEIEAERAARAKVEKQRSDLSRELEEISERLEEAGGATSAQIEMNKKREAEFQKLRRDLEEATLQHEATAAALRKKQADSVAELGEQIDNLQRVKQKLEKEKSEFKMEVDDLSSNMEQITKSKINFERLSKSLEDQISDLKSINEEKLHSVNELTTQKARMQTEVGELSRKLEEKETLTGQLSRVKQCNSQQIEDLKRQLEDEVKAKNALAHAVQSARHDCDLLREQFDEEQEAKGELQRAMSKANAEVAQWRTKYETDAIQKTEELEEAKKKIAVRLQEAEEHIETANAKCASLEKTKQRLQAEIEDLTTDVERAHSAATALDKKQKSFDKVMSEWKHKCEESQAELEGSQKEARSLSTELFKIKNAYEEALDQLETVKRENKNLQEEIFDLTEQLGESGKNVHELDKYRKQLEQDQAGLQASLEEAEASLEHEGGKILRVQLELNQVKAEVDRKIAEKDEEIDQIKRGFQRTVESMQATLDSETKSRNEALRLKKKMEGDLNEMEIQLNHANRQAAESQKHLRNAQNQMKDLQVHLDEALHGNEDLTEQNAMMRRRSDLLQAEIEETRSALEQTEKSRRVSEQELLATTERVQLLHSQNMCLINSKKKLESDLFQLQSEAEEALLESRSSEEKAKKAITDAAMMAEELKKEQDTSAHLERMKKNMEQTVKDLQHRLDEAEQIAMKGGKKHIQKLEARVHELEAEVDAGQRRNSEAVKGTRKYESKIKELSFQAEEDRKNLLRLQDLVDKLQLKVKAYKRHSEEAEEQANVSLSKFRTLQHELDEAQERADVAESQVNKLRAKSRDVASQKVRSDAEE

> Tr_Myh6/7
MGDAAMSEFGAAAPYLRKSDKERLEAQTRVFDMKKECFVPDPEIEYIKASITNREGDKVTVNTAGGKTVTVKECDVHTQNPPKFDKIEDMAMFTFLHEPAVLFNLKERYAAWMIYTYSGLFCVTVNPYKWLPVYNQEVVVAYRGKKRTEAPPHIFSISDNAYQYMLTDRENQSILITGESGAGKTVNTKRVIQYFASIAAGGGKKEGNPDKKGTLEDQIIQANPALEAFGNAKTIRNDNSSRFGKFIRIHFAASGKLASADIETYLLEKSRVTFQLKAERDYHIFYQILSQKKPELLEMMLITNNPYDYAYISQGETTVTSINDAEELMATDSAFDVLGFTQEEKNSVYKLIGAIMHYGNMKFKQKQREEQAEADGTEDADKSAYLMGLNSADLIKGLCHPRVKVGNEWVTKGQNVQQVNYAIGALSKSVYEKMFLWMVVRINQSLDTRQPRQYFIGVLDIAGFEIFDFNTFEQLCINFTNEKLQQFFNHHMFVLEQEEYKKEGIEWTFIDFGMDLQACIDLIEKPMGIMSILEEECMFPKASDATFKAKLYDNHLGKSANFQKPRIVKGKPEAHFALMHYAGTVDYNINNWLVKNKDPLNETVVALYQKSNLKLLSILFANYSGADAAMTEGTEGKKEKKKKGSSFQTVSALHRENLNKLMTNLRSTHPHFVRCIIPNETKTPGAMENPLVMHQLRCNGVLEGIRICRKGFPNRILYGDFKQRYRILNPAAIPEGQFIDSRKGAEKLLGSLDIDHNQYKFGHTKVFFKAGLLGLLEEMRDERLSKIITAIQARSRGLLSRIEYQKIVERRDALLVIQWNVRAFMGVKNWPWMKLYFKIKPLLRSAEAEKEMANMKEEFLKLKEAFAKSEARRKELEEKMVTLLQEKNDLQLQVQAEQDNLCDAEERCEGLIKNKIQLEAKSKELTERLEDEEEMNAELTAKKRKLEDECSELKKDIDDLELTLAKVEKEKHATENKVKNLTEEMAALDEIIAKLTKEKKALQEAHQQTLDDLQSEEDKVNTLTKAKTKLEQQVDDLEGSLEQEKKIRMDLERAKRKLEGDLKLTQETVMDLENDKQQLEERLKKKDFEISQLNGKIEDEQAIIIQLQKKLKELQARVEELEEELEAERAARAKVEKQRADLARELEEISERLEEAGGATTLQIEMNKKREAEFLKLRRDLEEATLQHEATAAALRKKQADSVADLGEQIDNLQRVKQKLEKEKSELRLELDDVVSNMEQIVKSKTNLEKTCRTLEDQMNDYRSKCDEYQRTIHDFTTQKAKLQAENDEFTRQADDKESLISQLTRGKNSYTQQLEDIKRQLEEEVKAKNALAHALQSARHDCDLLREQYEEEQEAKAELQRSMSKANSEVAQWRTKYETDAIQRTEELEEAKKKLXQRLQEAEEAVEAVNAKCSSLEKTKHRLQNEIEDLMVDVERSNAAAAALDKKQRNFDKVLSEWKQKYEESQCELEGSQKEARALSTELFKLKNSYEESLDHLETMKRENKNLQEEISDLSEQLSEGGKSIHELEKLRKQLEQEKNEIQSALEEAEASLEHEEGKILRAQLEFNQVKADIERKLAEKDEEMEQSKRNLQRTIDTLQSSLEAECRSRNEALRLKKKMEGDLNEMEIQLSQANRQAAEAQKQLKSVHAHLKDCQIQLDEAMRANDEMKENIAIVERRNNLLQAEVEELRAALEQTERSRKLAEQELLDVSERVQLLHSQNTGLINQKKKLEVDASQLQTEVEEAVQECRNAEDKAKKAITDAAMMAEELKKEQDTSAHLERMKKNMEQTIKDLQHRLDEAEQIAMKGGKKQVQKLEARVRDLETEVEMEQKKSSEALKGIRKYERRIKELTYQTEEDRKNVVRLQDLVDKLQMKVKAYKRAAEEAEEQANTHLGKFRKLQHELDEAEERADIAESQVNKLRAKSRDVGSKVRGL


>Tr_Myh5
MPLFDTTEFGEAAPYLRKSDQELLTSRTIAFDGKKRAWIPDDKEAYIEIEIKELKGDKVIVETKDGRTLTVKDGDIQQMNPPKYDMMEDMAMLTHLNEASVLYNLRRRYSAWMIYTYSGLFCVTVNPYKWLPVYTAPVVAAYKGKRRSESPPHIYSIADNAYNDMLRNRENQSMLITGESGAGKTVNTKRVIQYFAIVAALGDTTAKKGGTLEDQIIEANPAMEAFGNAKTIRNDNSSRFGKFIRIHFGHTGKLASSDIDIYLLEKSRVIFQQPGERSYHIYYQIMSQKKPELLDMLLVSSNPYDYHFCSQGVTTVESMDDGQELMATDHAMDILGFLPDEKYGCYKIVGAIMHFGNMKFKQKQREEQAEADGTESADKASYLMGVSSADLMKGLLHPRVKVGNEYVVKGQNVEQVNYAVGALAKATYDRMFKWLVGRINRTLYTSLPRQYFIGVLDIAGFEIFELNSFEQLCINFTNEKLQQFFNHHMFILEQEEYKREGIEWTFIDFGLDLQACIDLIEKPMGIMSILEEECMFPKATDYSFKAKLYDNHIGKSPNFQKPRPDKKRKYEAHFELVHYAGVVPYNIVGWLDKNKDPLNETVVACFQKSANKLLACLYENYVGSDSASDHKTGIKEKRKKAASFQTVSQVHKENLNKLMTNLRSTQPHFVRCIIPNETKTPGIIDPFLVLHQLRCNGVLEGIRICRKGFPNRIFYAEFKQRYRILNPQAIPDDKFVDSRKGAEKLLSTLDIDHIQYRFGHTKVFFKAGLLGHLEEMRDERLAKVLTLLQAAARGKIMRMELRKMTERRDALMIIQWNIRAFNAVKHWPWMKLFFKIKPLLKSATTEKELASLKEELAKLKEALEKSEVKRKELEERQVSLIQEKNDLALQLQAEQDNLADAEERCDLLIKTKIHLEAKVKEIMERLEDEEEINTSILASKRKLEDECAELKKDIDDLEITLAKVEKEKHATENKVKNLIEEMAALDETILKLSKEKKALQEAHQQTLDDLQAEEDKVNTLTKAKIKLEQQVDDLEGSLEQEKKLRMDLERAKRKLEGDVKLSLESIMDLENDKQQLEEKLKKKDFEMNELSTRVEDEQALVNQLQKKIKELQARTEELEEELESERACRAKVEKQRSEVARELEELSERLEEAGGATSAQIEMNKKREADFLKLRRDLEEAMLHHEATTAALRKKHADSVAELSEQIDSLQRVKQKLEKERSEAKMEIDDLASTVEQLSKNKASAEKTCRLYEDQMNEAKAKVDELQRQLNDSNSQRARAQTESGELSRKLEEREAMVAQLQRSKNSFSQSVEELKKQLEEENKAKSSLAHALQSSRHDCDLLREQYEEEQEAKGELQRALSKANAEVAQWRTKYETDAIQRTEELEEAKKKLVMRLQEAEETVEGSNAKCSSLEKTKHRLQTEIEDLVVDLERANAAATALDKKQRNFDKVLAECRQKYEECQSELEASQKESRGLSTELFKLKNSYXESLDHLETVKRENKNLQEEIADLTDQISQGAKTIHELEKMKKGLELEKSEIQAALEEVEGTLEHEESKTLRIQLELNQMKADVDRKLAEKDEELDNLRRNHQRTLNFMQATLDAEAKSRNEAVRLRKKMEGDLNEMEVQLNHANRQAAESQKLLRNLQVQIKDIQMELDETVHQNEELKDQVVVTERRNNLLAAEVEELRALLEQNDRARKLAEHELLEATERVNLLHSQNTSLISQKKKLENDLSTLSNEVDDAVQECRNAEDKAKKAITDAAMMAEELKKEQDTSAHLERMKKNMEQTVKDLQMRLDEAEQIALKGGKKQVQKLEARVKELENELESEQKKSQEFQKGVRKYERRIKELSYQAEEDKKNLIRLQELIDKLQVKVKSYKRQTEEAEEQANCNLSKYRKLQHELNDAEERADMAETQVNKLRVRTRDQGSKLAE

>Ai_MYH
MNIDFSDPDFQYLAVDRKKLMKEQTAAFDGKKNCWVPDEKEGFASAEIQSSKGDEITVKIVADSSTRTVKKDDIQSMNPPKFEKLEDMANMTYLNEASVLYNLRSRYTSGLIYTYSGLFCIAVNPYRRLPIYTDSVIAKYRGKRKTEIPPHLFSVADNAYQNMVTDRENQSCLITGESGAGKTENTKKVIMYLAKVACAVKKKDEEASDKKEGSLEDQIIQANPVLEAYGNAKTTRNNNSSRFGKFIRIHFGPTGKIAGADIETYLLEKSRVTYQQSAERNYHIFYQICSNAIPELNDVMLVTPDSGLYSFINQGCLTVDNIDDVEEFKLCDEAFDILGFTKEEKQSMFKCTASILHMGEMKFKQRPREEQAESDGTAEAEKVAFLCGINAGDLLKALLKPKVKVGTEMVTKGQNMNQVVNSVGALAKSLYDRMFNWLVRRVNKTLDTKAKRNYYIGVLDIAGFEIFDFNSFEQLCINYTNERLQQFFNHHMFILEQEEYKKEGIAWEFIDFGMDLQMCIDLIEKPMGILSILEEECMFPKADDKSFQDKLYQNHMGKNRMFTKPGKPTRPNQGPAHFELHHYAGNVPYSITGWLEKNKDPINENVVALLGASKEPLVAELFKAPEEPAGGGKKKKGKSSAFQTISAVHRESLNKLMKNLYSTHPHFVRCIIPNELKQPGLVDAELVLHQLQCNGVLEGIRICRKGFPSRLIYSEFKQRYSILAPNAIPQGFVDGKTVSEKILAGLQMDPAEYRLGTTKVFFKAGVLGNLEEMRDERLSKIISMFQAHIRGYLIRKAYKKLQDQRIGLSVIQRNIRKWLVLRNWQWWKLYSKVKPLLSIARQEEEMKEQLKQMDKMKEDLAKTERIKKELEEQNVTLLEQKNDLFLQLQTLEDSMGDQEERVEKLIMQKADFESQIKELEERLLDEEDAAADLEGIKKKMEADNANLKKDIGDLENTLQKAEQDKAHKDNQISTLQGEISQQDEHIGKLNKEKKALEEANKKTSDSLQAEEDKCNHLNKLKAKLEQALDELEDNLEREKKVRGDVEKAKRKVEQDLKSTQENVEDLERVKRELEENVRRKEAEISSLNSKLEDEQNLVSQLQRKIKELQARIEELEEELEAERNARAKVEKQRAELNRELEELGERLDEAGGATSAQIELNKKREAELLKIRRDLEEASLQHEAQISALRKKHQDAANEMADQVDQLQKVKSKLEKDKKDLKREMDDLESQMTHNMKNKGCSEKVMKQFESQMSDLNARLEDSQRSINELQSQKSRLQAENSDLTRQLEDAEHRVSVLSKEKSQLSSQLEDARRSLEEETRARSKLQNEVRNMHADMDAIREQLEEEQESKSDVQRQLSKANNEIQQWRSKFESEGANRTEELEDQKRKLLGKLSEAEQTTEAANAKCSALEKAKSRLQQELEDMSIEVDRANASVNQMEKKQRAFDKTTAEWQAKVNSLQSELENSQKESRGYSAELYRIKASIEEYQDSIGALRRENKNLADEIHDLTDQLSEGGRSTHELDKARRRLEMEKEELQAALEEAEGALEQEEAKVMRAQLEIATVRNEIDKRIQEKEEEFDNTRRNHQRALESMQASLEAEAKGKADAMRIKKKLEQDINELEVALDASNRGKAEMEKTVKRYQQQIREMQTSIEEEQRQRDEARESYNMAERRCTLMSGEVEELRAALEQAERARKASDNELADANDRVNELTSQVSSVQGQKRKLEGDINAMQTDLDEMHGELKGADERCKKAMADAARLADELRAEQDHSNQVEKVRKNLESQVKEFQIRLDEAEASSLKGGKKMIQKLESRVHELEAELDNEQRRHAETQKNMRKADRRLKELAFQADEDRKNQERLQELIDKLNAKIKTFKRQVEEAEEIAAINLAKYRKAQHELEEAEERADTADSTLQKFRAKSRSSVSVQRSSVSVSASN
