# Supplementary material for: Modular co-option of cardiopharyngeal genes during non-embryonic myogenesis
Source: EvoDevo. 2019 Mar 5;10:3. doi: 10.1186/s13227-019-0116-7 (PMC6399929; doi:10.1186/s13227-019-0116-7)
Supplement: Supplementary file 19 — Additional file 19. Table 1: Primer sequences. [file 13227_2019_116_MOESM19_ESM.docx]

| **Gene name** | **Primer sequence 5’-3’** |
| --- | --- |

| Myh III | Fw | TCGAAGTCCAAGCAATCCCT |
| --- | --- | --- |
|  | Rew | GCGCCTCGTACTTCTTCTTG |

| Nk4 | Fw | GTTGCGAGCGAGTAAAGAGG |
| --- | --- | --- |
|  | Rew | GCGACGACATAAAACCCGAA |

| Tbx1/10 | Fw | TGTCTTGAGAGTACAGCGCA |
| --- | --- | --- |
|  | Rew | CATCCAATGCGATCCCTTGG |

| FoxF | Fw | TCACCTCAACCATCTGGACA |
| --- | --- | --- |
|  | Rew | CCGGGTCGATAGTCCAGTAA |

| Islet | Fw | CTCGCACAAAGACCCGAAAA |
| --- | --- | --- |
|  | Rew | ATACGTTGATGTCTTGCGCC |

| Myh II | Fw | TGGTTGCTCTGTATCGCAAG |
| --- | --- | --- |
|  | Rew | TCCCATTTCCTTCTCGATTG |

| Mrf | Fw | AACACAGCACGCGATACATC |
| --- | --- | --- |
|  | Rew | TGGGATTGGAGCAGGAACAT |

| Myh E | Fw | TTCGACCAGACAAGGAGCTT |
| --- | --- | --- |
|  | Rew | TTGCGAAGAGTGGTCATCAG |

| Zic-r.a  (Macho-1) | Fw | CCAACTGCAGCAAAGTGTCT |
| --- | --- | --- |
|  | Rew | ATGGCCTTTCACCGGTATGA |

| ZicL | Fw | ATTTGGATGCATACGTGACTTG |
| --- | --- | --- |
|  | Rew | TCGGTATCCTGAACCTCTTGTT |

| Lim/ Lhx3 | Fw | TACATCTGCGCCGTATCAAT |
| --- | --- | --- |
|  | Rew | CATGTTTTCCCGGCTGTAAC |

| Tbx6 | Fw | TGCAAGTTCAACTCTGTGACA |
| --- | --- | --- |
|  | Rew | GCAGTCACAGTGATGAATACGGT |

| Mesp | Fw | ATGAGGAAACTGCGTGTCTACGAT |
| --- | --- | --- |
|  | Rew | TCAGCGAAATGCAGCAACTG |

| Ebf | Fw | TGCTGCGACCATAAAAGTTG |
| --- | --- | --- |
|  | Rew | GCCGACTGCAACTGACTGTA |

| Ets | Fw | TGCCAAAATCAACGAAGCCA |
| --- | --- | --- |
|  | Rew | TCTTCCTTGACCTGGCTCAG |
