# Supplementary material for: Modular co-option of cardiopharyngeal genes during non-embryonic myogenesis
Source: EvoDevo. 2019 Mar 5;10:3. doi: 10.1186/s13227-019-0116-7 (PMC6399929; doi:10.1186/s13227-019-0116-7)
Supplement: Supplementary file 21 — Additional file 21. Fig. 16: Examples of recurring background. [file 13227_2019_116_MOESM21_ESM.pdf]

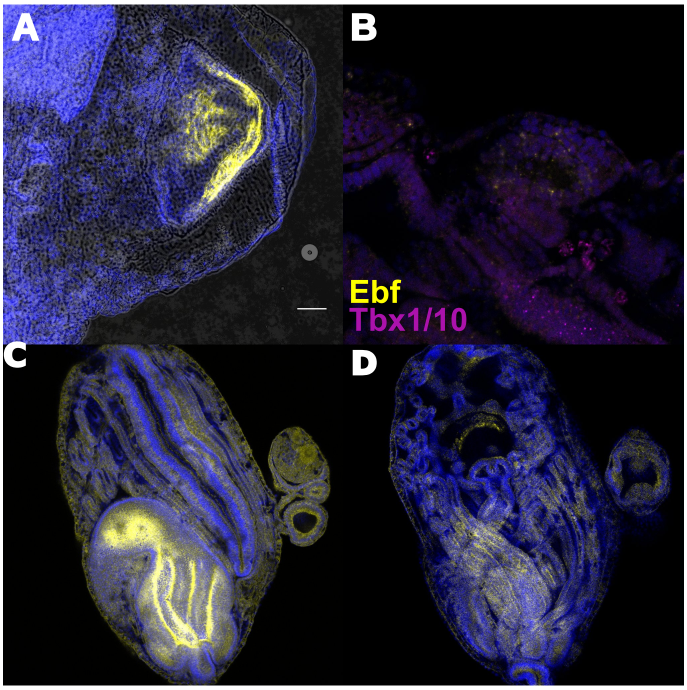

Supp. Fig. 16. Examples ouf background.The most recurring background is found in forming tunic in the (A) oral and atrial siphon. (B) TRITC coloration stains a specific celltype in the vasculature.
(C-D) The detunicated objects show staining in the gut, the gonads. Hoechst (blue).
